# CuCl-Promoted β‑Acylation of Cyclopropanols with Thioesters

**DOI:** 10.1021/acs.orglett.6c00432

**Published:** 2026-03-10

**Authors:** Savva Ponomarev, Sandra W. Papińska, Michael Stier, Johannes Kästner, Ivana Fleischer

**Affiliations:** † Institute of Organic Chemistry, Faculty of Science, 234487 Eberhard Karls Universität Tübingen, Auf der Morgenstelle 18, 72076 Tübingen, Germany; ‡ Institute for Theoretical Chemistry, 9149 University of Stuttgart, Pfaffenwaldring 55, Stuttgart 70569, Germany

## Abstract

CuCl-induced cyclopropyl alcohol ring-opening followed by β-acylation with thioester is reported. Cyclopropanols and thioesters with various substitution patterns were successfully subjected to the reaction, and a series of 1,4-dicarbonyl compounds were synthesized. The mechanistic investigation revealed the involvement of Cu­(I)-homoenolate, which reacts with the thioester via oxidative addition. Both experimental and computational evidence were found. Operational simplicity, high chemo- and regioselectivity, and good to excellent yields are the core features of the presented approach.

## Graphical abstract

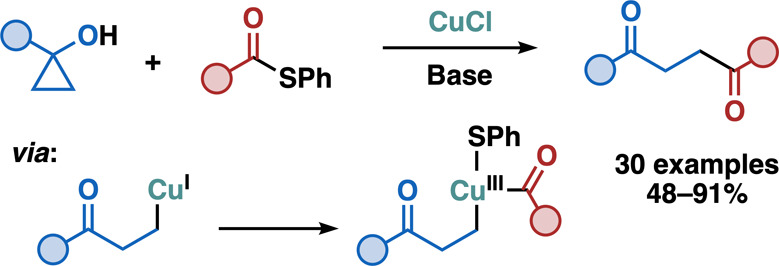

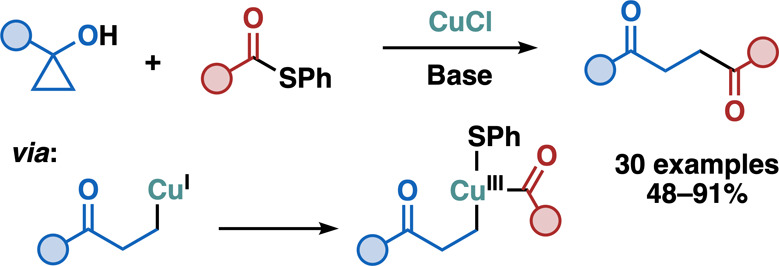

Cyclopropanols and their derivatives are synthetically useful compounds since they easily undergo various ring-opening processes due to ring strain. Their general advantage is that under certain conditions, they can generate nucleophilic as well as electrophilic intermediates or radicals (Scheme 1A), which opens the way to a wide spectrum of transformations, including couplings.^1^ An example of the generation of electrophilic intermediates is the Ni-catalyzed arylation of cyclopropyl tosylates with arylboronic acids, reported by Rousseaux and co-workers.^2^ Many valuable developments have been made in the field of radical reactions of cyclopropanols. Oxidation of the hydroxy group leads to an oxygen-centered radical, which is unstable and readily rearranges into a linear carbon-centered β-keto radical.^3^ The latter can directly attack radical acceptors^4^ or be coupled with another radical using transition metal catalysis.^5^

**Scheme 1:**
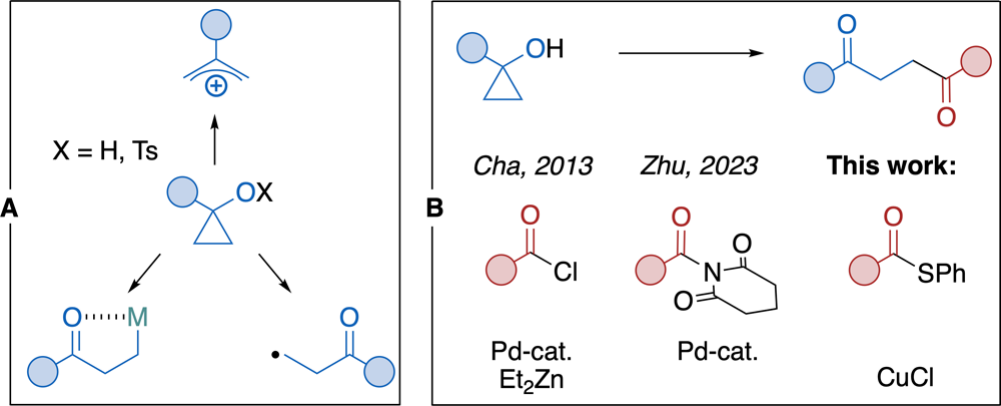
Cyclopropanol-Derived Intermediates (A), Synthesis of 1,4-Diones from Cyclopropanols (B)

The functionalization of cyclopropanols via formation of nucleophilic metal homoenolate is a rapidly advancing area of research.^6^ This activation gives access to modification of the β-carbon atom with a diverse range of electrophiles. Numerous reports disclose allylation, propargylation, and alkynylation procedures via sequential cyclopropanol activation with organozinc and -copper reagents.^7^ Benzyl chlorides as well as *N*-hydroxyphtalimides were found to be suitable for catalytic β-alkylation of cyclopropyl alcohols.^8^ Installation of a trifluoro-methyl group was achieved with usage of Togni reagent and Cu­(I) catalyst.^9^ However, the authors claimed that the reaction may proceed via both nucleophilic and radical intermediates. Employment of palladium as catalyst enabled β-arylation with aryl halides and triflates.^10^ Moreover, several techniques for metal homoenolate β-acylation have been developed (Scheme 1B).^11^ The generated 1,4-dicarbonyl functionality can be found in several bioactive molecules, and it represents a valuable building block in the synthesis of various heterocycles.^12^

In 2013, Cha and co-workers published Pd-catalyzed acylation of 1-alkyl- and 1-benzyl-substituted cyclopropanols.^13^ A stoichiometric amount of Et_2_Zn was used to generate the corresponding zinc homoenolates, which reacted with acyl chlorides in the presence of Pd­(PPh_3_)_4_. The authors did not comment on the mechanism, but without the Pd catalyst, only acylation of the cyclopropoxide took place. Interestingly, no organometallic reagent was needed in the more recently reported Pd-catalyzed acylation using activated amides.^14^ In this work, we report another convenient way to obtain 1,4-diones through copper homoenolate intermediates acylated by S-phenyl-substituted thioesters (Scheme 1B), which are easily accessible via known procedures starting from different compound classes.^15^ Thioesters are benchtop-stable alternative electrophiles to acyl chlorides, and they have been employed in various coupling reactions.^16^

Initially, we applied the reported conditions for acyl chlorides employing Pd­(II)-catalyst and Zn_2_Et to achieve the desired thioester coupling,^13^ but low regioselectivity and unsatisfactory yields were observed. Omitting Et_2_Zn proved completely inefficient, in contrast to coupling of activated amides.^14^ Considering the higher reactivity of copper homoenolate in comparison with zinc,^7a^ we decided to activate the substrate with a combination of CuCl and a base instead of Et_2_Zn, which turned out to be the key move in the optimization process (for more details, see the SI). The optimized conditions (1.5 equiv. CuCl, 1 equiv. K_2_CO_3_, in acetonitrile at 80–90 °C overnight) were applied to coupling of a broad scope of cyclopropanols and thioesters to obtain 30 products in 48 to 91% yields (Scheme 2A and 2B). 1-Alkyl-substituted and both electron-rich and -deficient 1-aryl-substituted cyclopropyl alcohols were successfully converted into corresponding 1,4-diones **3aa-3la**. Functional groups like ether, protected amines, and conjugated double bond tolerated the developed conditions. 1,2-Disubstituted cyclopropyl alcohols regioselectively formed products via cleavage of the less substituted bond of the three-membered ring (**3oa**-**sa**). Among the tested thioesters, aromatic compounds with diverse substitution patterns furnished products **3ab-ai** in good yields. Alkyl- and alkenyl-substituted thioesters also performed well, and the products **3aj-3al** were obtained in 75–88% yields. It should be mentioned that the system has limitations: aryl iodides are prone to dehalogenation, and *ortho*-substituted 1-aryl-cyclopropanols were not fully converted even at 90 °C (see SI, Scheme S1). Nonetheless, the reaction shows high versatility: almost 70% of products were obtained in 80% yield or higher. Additionally, other electrophiles were tested under designed conditions (SI, Scheme S2). More reactive acyl chlorides, S-pyridyl-thioesters, as well as less electrophilic S-alkyl-substituted thioesters showed noticeably lower efficiency. Cyclohexenone was completely unreactive, while allyl bromide gave 79% of the coupling product.

**Scheme 2:**
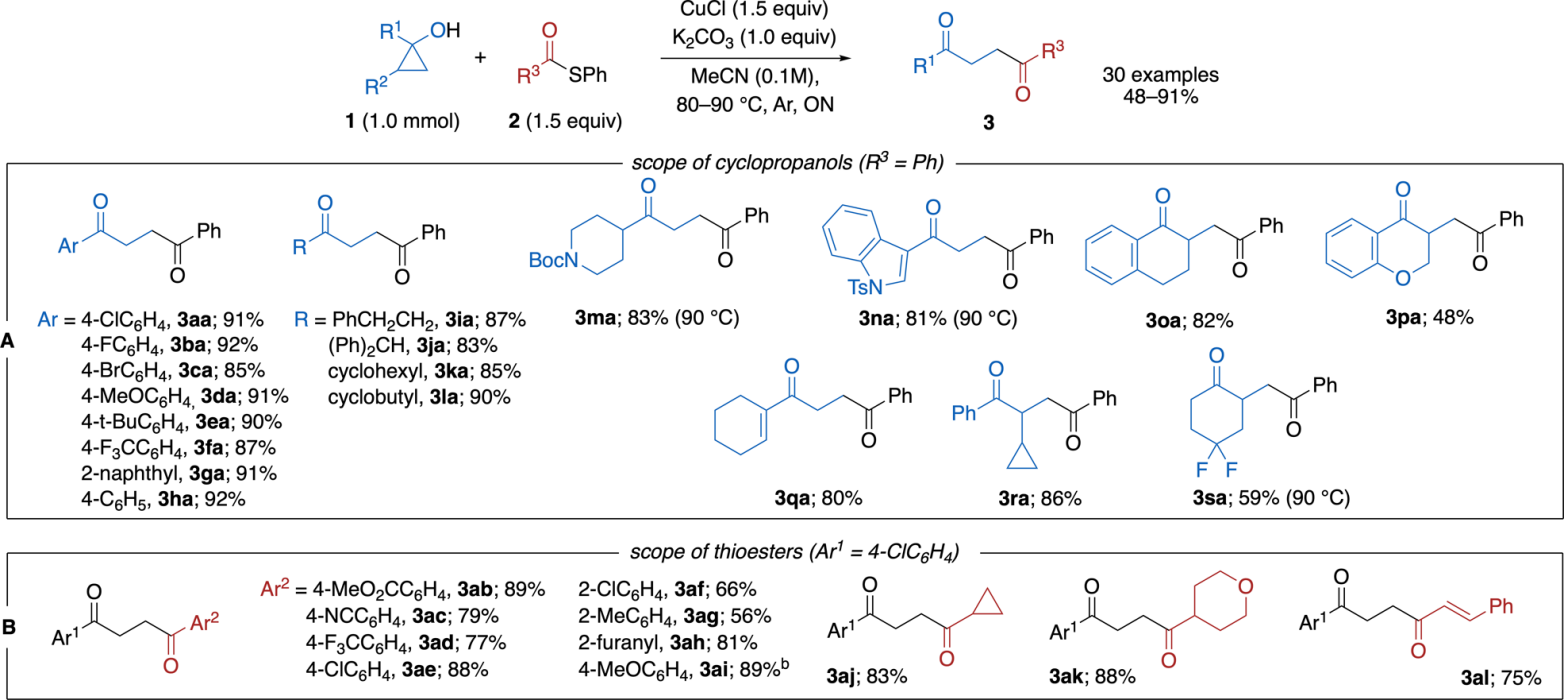
Scope of the Copper-Mediated Coupling of Cyclopropanols (A) and Thioesters (B)^a^

a Reaction conditions: **1** (1.0 mmol), **2** (1.5 mmol), CuCl (1.5 mmol), and K_2_CO_3_ (1.0 mmol) are mixed in MeCN (0.1 M) and heated overnight (ON) to 80 °C under Ar. Isolated yields.
b 90 °C.

Copper catalysis and mediation are broadly used tools in cyclopropyl alcohol chemistry, mainly due to the ability of copper to form several types of functional intermediates. Their nature, in general, depends on the oxidation state of copper (Scheme 3). Copper­(I) forms Cu­(I)-homoenolates **A** via β-C elimination, and copper­(II) furnishes β-keto radicals **C** via a redox process. Copper­(II) can also lead to Cu­(II)-homoenolates **B**, but they are usually unstable and readily decompose into the aforementioned β-keto radicals **C** and copper­(I).^6^ At the same time, copper­(I) can disproportionate under certain conditions and form copper­(II).^17^ Thus, regardless of the initial oxidation state of copper, copper species with a different oxidation state might be operational in the target reaction. Hypothetically, all three types of intermediates can react with the thioester, leading to 1,4-diketone product (see SI for more details). Cu­(I)- and Cu­(II)-homoenolates **A** and **B** can react with the thioester via conventional nucleophilic 1,2-addition. Cu­(I)-homoenolate **A** can also be engaged in the oxidative addition process. The β-keto radical **C** can attack the carbonyl group of the thioester (see SI), since thioesters are known as radical acceptors.^18^ A distinctive feature of the ionic vs radical pathway is the regioselectivity of the ring-opening of 1,2-disubstituted cyclopropyl alcohols (Scheme 3a). While the radical C–C bond cleavage occurs at the more substituted bond, the ionic β-C elimination leads to sterically less hindered metal homoenolate.^6^

**Scheme 3:**
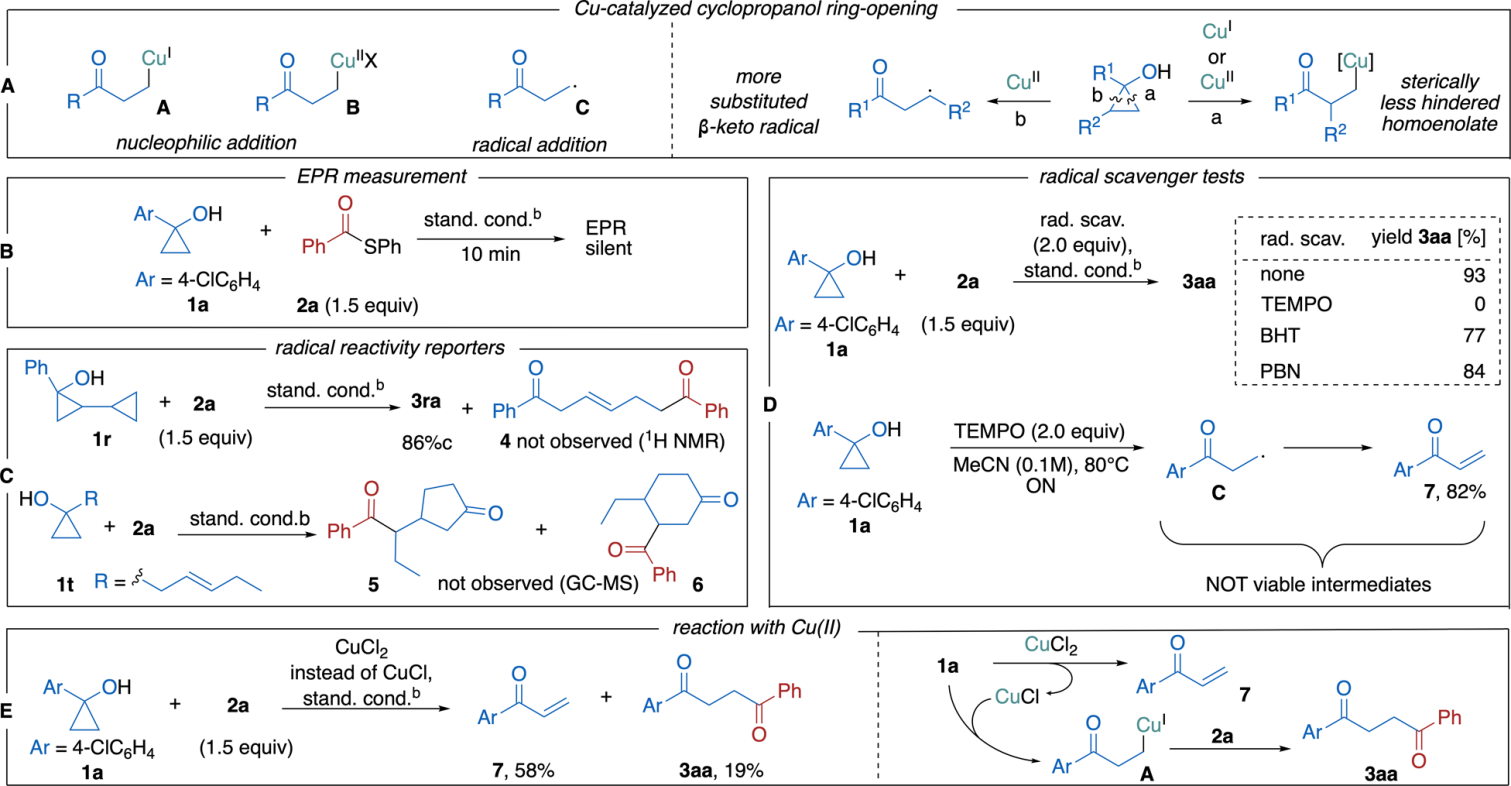
Possible Reaction Pathways of Cyclopropanol Ring-Opening (A) and Determination of Active Intermediate (B–E)^a^

a The yields are calculated via ^1^H NMR using 1,3,5-trimethoxybenzene as internal standard, unless otherwise specified.
b CuCl (1.5 equiv) and K_2_CO_3_ (1.0 equiv) in MeCN (0.1 M) at 80 °C overnight.
c Isolated yield.

A series of experiments were performed to differentiate between the radical and ionic mechanism. The EPR spectrum of the reaction mixture did not reveal any signals, which testifies to the absence of paramagnetic species in the reaction (Scheme 3B). The coupling of 2-cyclopropyl-substituted substrate **1r** with **2a** led to the diketone **3ra** with a retained cyclopropyl substituent in 86% yield. No product **4**, which would be expected in the case of radical ring-opening, was detected (Scheme 3C). Similarly, the allyl-substituted cyclopropyl alcohol **1t** did not furnish any of the possible products of radical cyclization **5** and/or **6** (Scheme 3C). Furthermore, reactions in the presence of radical scavengers were conducted. While BHT (2,6-di-*tert*-butyl-4-methylphenol) and PBN (*N*-*tert*-butyl-α-phenylnitrone) did not influence the product formation, no target diketone **3aa** was observed when TEMPO was used (Scheme 3D). However, it was shown that TEMPO oxidizes cyclopropanol **1a** into the enone **7** via **C** (Scheme 3D).^19^ Therefore, the total suppression of **3aa** formation in the presence of TEMPO shows that neither **C** nor **7** are viable intermediates in the reaction. When CuCl was replaced with CuCl_2_, the reaction showed significantly lower efficiency, as only 19% of **3aa** was obtained, and the vinyl ketone **7** was formed as the main product (Scheme 3E). Generally, CuCl_2_ could lead to intermediates **B** or **C**, but the low yield of **3aa** testifies against engagement of these intermediates in product formation. Cu­(I) species generated in the oxidation of **1a** by Cu­(II) could still be involved in the reaction via Cu­(I)-homoenolate **A** (Scheme 3A). To summarize, the results of all conducted tests point against the participation of any paramagnetic species (organic radicals and Cu­(II) species). Therefore, the reaction most likely proceeds via Cu­(I)-homoenolate **A** (Scheme 3A).

This intermediate can further react with the thioester via 1,2-addition or oxidative addition. DFT computations were performed using ORCA,^20^ version 6.0.1, with the ChemShell^21^ interface to differentiate between these pathways.^22^ Geometry optimizations and frequency calculations were performed with the ωB97X-3c^23^ composite method. Single point calculations were performed on the ωB97M-V/def2-QZVPP^24^ level of theory in combination with the SMD solvation model^25^ for acetonitrile. Gibbs free energies were computed within the rigid-rotor harmonic-oscillator approximation. More computational details and all geometries are given in the SI. Their Gibbs free energy profile along with the corresponding structures are depicted in Scheme 4A. The formation of the precomplex, as well as the release of the final product, were not considered, as these reactions might involve solvent molecules or counterions. It remains unclear what the exact species in the corresponding solvent are, making computation impossible. Both identified precomplexes exhibit similar stability. However, the activation barrier for the nucleophilic addition pathway is relatively high (119 kJ mol^–1^), making this pathway unlikely. Consequently, the nucleophilic addition route was not further considered. In contrast, the activation barrier for the oxidative addition pathway is 26 kJ mol^–1^, indicating that this is the preferred reaction mechanism. This barrier corresponds to the rate-determining step. The subsequent intermediate is slightly more stable than the precomplex. Formation of the final complex from this intermediate is barrierless and highly exergonic (155 kJ mol^–1^), effectively preventing back-reactions.

**Scheme 4:**
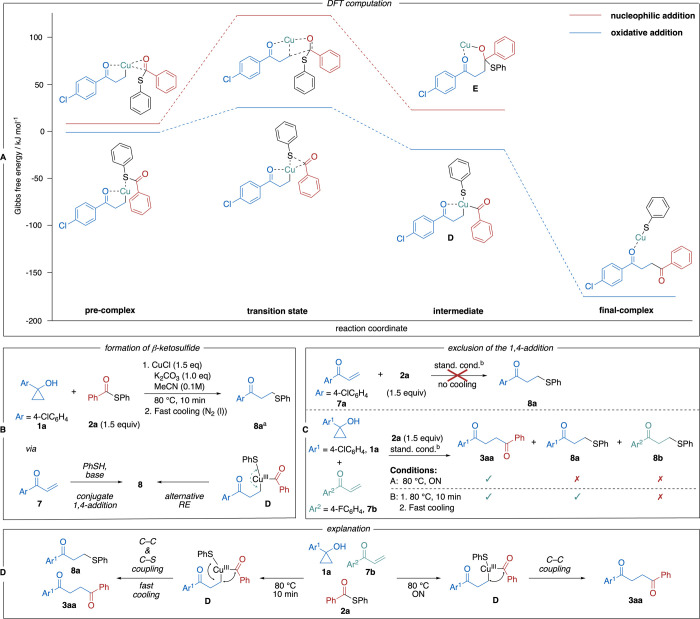
Investigations of Possible Pathways Including Cu­(I)-Homoenolate Intermediate^a^

a The yields are calculated via ^1^H NMR or GC FID using internal standards.
b CuCl (1.5 equiv) and K_2_CO_3_ (1.0 equiv) in MeCN (0.1 M) at 80 °C overnight.

The experimental confirmation of these results proved difficult, since it was not possible to detect any intermediates spectroscopically. Based on the kinetic investigation (see SI), which showed that the maximal reaction rate is reached between 2–20 min, we decided to find indirect evidence for intermediates within this time by freezing the standard reaction after 10 min using liquid N_2_ (Scheme 4B). The analysis with GCMS and ^1^H NMR revealed the formation of β-keto sulfide **8a**, which was not detected under standard conditions. This compound could be formed via conjugate 1,4-addition of a thiolate to enone **7a** (eventual product of β-H elimination from **1a**) or via alternative reductive elimination from Cu­(III)-complex **D** (Scheme 4B). To test the feasibility of 1,4-addition of a thiolate to **7a**, a series of control experiments was carried out (Scheme 4C). The direct reaction of enone **7a** with **2a** under standard conditions did not furnish product **8a**. Thus, the thioester is not a source of thiolate, which would react with **7a**. Furthermore, we performed a competition experiment using cyclopropyl alcohol **1a** and enone **7b** containing different aryl groups. Under standard conditions, only expected coupling product **3aa** formed. Upon cooling, also β-keto sulfide **8a** was observed, but no sulfide **8b** generated from the enone. This confirms that β-keto sulfide cannot be formed through 1,4-addition of thiolate to enone but rather through reductive elimination from Cu­(III)-complex **D** (Scheme 4D). This process seems to be suppressed at higher temperatures. Furthermore, we observed the formation of diphenylsulfide, which could be generated via a decarbonylation process from **D**, which additionally supports this pathway.

Based on the collected data, the following mechanism can be proposed (Scheme 5). Under basic conditions, cyclopropanol **1** is converted into cyclopropanolate **G**, which furnishes the homoenolate **A** through β-C elimination. Subsequently, **A** reacts with thioester **2** via oxidative addition providing **D**, which undergoes reductive elimination yielding product **3**. Cu­(I) turnover is not possible due to the precipitation of CuSPh.

**Scheme 5:**
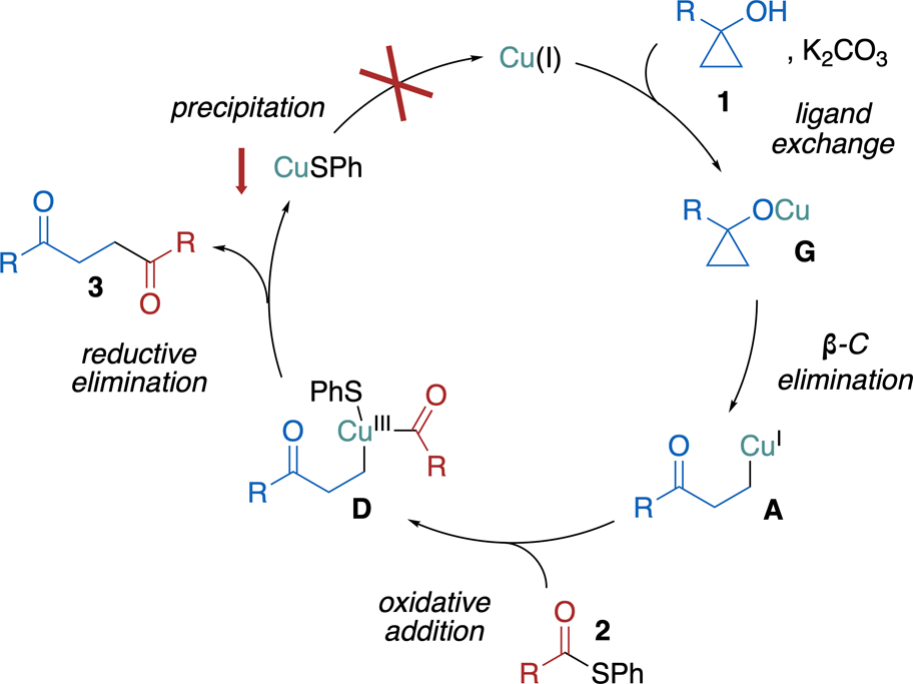
Plausible Reaction Mechanism

In summary, we developed a novel approach to β-acylation of cyclopropyl alcohols via *in situ* generated Cu­(I)-homoenolate using thioesters as the acyl source. Mechanistic investigations point toward the ionic pathway proceeding through the Cu­(I) homoenolate intermediate, which reacts with thioester via oxidative addition.

## Supplementary Material







## Data Availability

The data underlying this study are available in the published article and its Supporting Information.

## Notes

Text file of all computed molecule Cartesian coordinates in a format for convenient visualization (XYZ)
Experimental details, compound characterization data and NMR spectra (PDF)
Text file of all computed molecule Cartesian coordinates in a format for convenient visualization (XYZ)
