# Supplementary material for: CuCl-Promoted β‑Acylation of Cyclopropanols with Thioesters
Source: Org Lett. 2026 Mar 10;28(11):3553–8. doi: 10.1021/acs.orglett.6c00432 (PMC13010281; doi:10.1021/acs.orglett.6c00432)
Supplement: Supplementary file 2 [file ol6c00432_si_002.pdf]

CuCl-Promoted β-Acylation of Cyclopropanols with Thioesters

Savva Ponomarev,[a] Sandra W. Papińska,[a] Michael Stier,[b] Johannes Kästner,[b] and Ivana Fleischer [a]*

[a]Institute of Organic Chemistry, Faculty of Science, Eberhard Karls Universität Tübingen, Auf der Morgenstelle 18, 72076 Tübingen, Germany

[b]Institute for Theoretical Chemistry, University of Stuttgart, Pfaffenwaldring 55, Stuttgart 70569, Germany

[a]* Email: ivana.fleischer@uni-tuebingen.de

Supporting information

1. Table of contents

1. TABLE OF CONTENTS 2
2. GENERAL INFORMATION 3
2.1. CHEMICALS AND GENERAL TECHNIQUES 3
2.2. ANALYTICAL TECHNIQUES 3
2.3. COMPUTATIONAL DETAILS 3
3. CYCLOPROPANOL SYNTHESIS 4
3.1. GENERAL PROCEDURE 1. SIMMONS-SMITH REACTION 4
3.2. GENERAL PROCEDURE 2. KULINKOVICH REACTION 4
4. COUPLING OF CYCLOPROPANOLS WITH S-PHENYL-SUBSTITUTED THIOESTERS 5
4.1. POSSIBLE REACTION PATHWAYS 5
4.2. OPTIMIZATION 5
4.3. GENERAL PROCEDURE 3. SYNTHESIS OF 1,4-DIKETONES 7
5. REACTION PROGRESS 8
6. SYNTHESIZED CYCLOPROPANOLS 10
7. SYNTHESIZED 1,4-DIKETONES 34
8. REFERENCES 99

2. General information

2.1. Chemicals and general techniques

All reactions were carried out under argon employing standard Schlenk technique or a glovebox (GS MEGA E-Line, Glovebox Systemtechnik, <10 ppm O_2, <0.1ppm H_2O) with pre-dried glassware. Solvents were dried via a commercially available solvent purification system (MB SPS-5). Column chromatography was performed with silica (35 – 70 µm, 60A) from Thermo Fischer. Flash chromatography was carried out using the Interchim Puriflash XS420 system with FlashPure EcoFlex Silica 25 g columns (50 µm, irregular). Chemicals were purchased from abcr, BLDpharm, Sigma-Aldrich, Thermo Fischer, Acros or TCI and were used without further purification. Extra pure CuCl (Sigma-Aldrich, AnhydroBeads™, ≥99,99%) and K_2CO_3 (Thermo Scientific Chemicals, Puratronic™, ≥99,997%) were used and stored in glovebox. K_2CO_3 and $CuCl_2$ were dried at 110°C under *vacuo* over at least 2 hours. Syringe pump Chemyx Fusion 100 was used. Low temperature in cooling experiments was achieved using a dewar with liquid nitrogen. Compounds **2a-m**, **7**, **9** and **1r** were synthesized according to reported procedures, respectively.[1, 2, 3, 4]

2.2. Analytical techniques

NMR spectra were recorded using the Bruker Avance III HDX 700, Bruker Avance III HDX 600 or Bruker Avance 400 spectrometers. ^{13}C NMR experiments were performed in proton-decoupled mode. ^{1}H NMR spectra are reported in parts per million (ppm) downfield relative to $CDCl_3$ (7.26 ppm) or CD_3CN (1.94 ppm), ^{13}C NMR spectra are reported in ppm relative to $CDCl_3$ (77.16 ppm), ^{19}F NMR spectra are reported in ppm relative to α,α,α-trifluorotoluene (-63.72 ppm). J coupling constants are given in Hertz with the usual designations for splitting patterns (s = singlet, d = doublet, t = triplet, q = quartet, dd = doublet of doublets, tt = triplet of triplets, dt = doublet of triplets, m = multiplet). GC-LR-MS (EI) analysis was performed with an Agilent 7820A/5977B GC-system/MSD using an Agilent 190915-433UI column (30 m × 250 µm × 0.25 µm). Program: 50 °C – 300 °C over 16 minutes. HR-MS (ESI/APCI-TOF) measurements were performed by the mass spectrometry department of the Institute of Organic Chemistry, University of Tübingen. Measurements were made using maXis 4G from Bruker (ESI/APCI-TOF). The molecular ion [M+H/Na]+ is given in m/z units. FT-IR spectra were measured by a Cary 630 FTIR by applying the neat sample on a diamond ATR sampler. Melting points were determined by visual detection employing a Büchi B-540 (heating rate 5 °C/min). Thin layer chromatography (TLC) was performed on aluminum plates coated with 0.20 mm silica gel 60 with fluorescence indicator UV254 from Macherey-Nagel and compounds were detected under UV light (254 nm) or using potassium permanganate stain (1.5 g $KMnO_4$, 10 g K_2CO_3, 5 ml NaOH aqueous (10%), 200 ml H_2O).

2.3. Computational details

Density functional theory calculations were performed using ORCA, version 6.0.1.[5] The resolution-of-identity approximation [6] was applied, together with the def2/J auxiliary basis set.[7] The chain-of-spheres algorithm [8] was applied for the efficient computation of Hartree-Fock exchange. Molecular geometries for minima and transition states were optimized with the DL-FIND optimization library [9] in ChemShell.[10] Conformer search was performed using CREST [11], version 3.0.2, with GFN2-xTB.[12] Geometry optimizations and frequency calculations were performed with the ωB97X-3c composite method.[13] Single-point calculations were performed with the ωB97M-V functional [14] in the def2-QZVPP basis [15], in combination with the SMD implicit solvation model [16] for acetonitrile. Gibbs free energies were calculated at conditions of $1 molL^{-1}$ and 300K within the rigid rotor harmonic oscillator approximation. Vibrational frequencies below $100 cm^{-1}$ were adjusted to meet this threshold.[9]

3. Cyclopropanol synthesis

3.1. General procedure 1. Simmons-Smith reaction

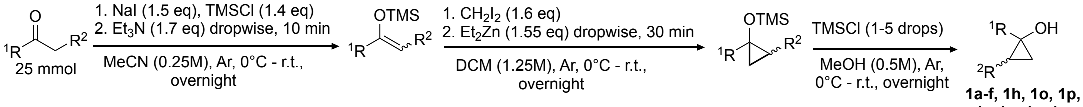

1a-f, 1h, 1o, 1p, 1r, 1s, 1v, 1w

1. A 250 ml Schlenk flask was charged with NaI (37.5 mmol, 1.5 eq), pre-dried under *vacuo* with a propane torch or a heat gun and backfilled with Ar. The flask was pre-dried under *vacuo* one additional time. After 100 ml of extra dry MeCN was added via syringe. NaI was let dissolve at room temperature while stirring. Next the parent ketone (25 mmol, 1.0 eq) was added. The solution was cooled down with an ice-water bath and TMSCl (35 mmol, 1.4 eq) was added, followed Et_3N (42.5 mmol, 1.7 eq, dropwise over 10 min.), which was by pre-dried over mol. sieves. The solution was let stir overnight at room temperature. After completion (monitored via ^1H NMR) the mixture was transferred into a 250 ml round-bottom flask with small amount of pentane or Et_2O. The solvent was evaporated under vacuo. The crude mixture was transferred onto a paper filter and washed into a 250 ml round-bottom flask with pentane or Et_2O. The solvent was evaporated under *vacuo*, the obtained liquid was directly submitted to the next step.
2. The silyl-enol ether (25 mmol, 1 eq) was transferred into a 100 ml Schlenk flask (pre-dried and backfilled with Ar three times) via syringe. The flask was evacuated until 1.0-2.0 mbar and backfilled with Ar three times. Next DCM (20 ml, 1.25M) was added, followed by CH_2I_2 (40.0 mmol, 1.6 eq). Upon cooling with an ice-water bath, Et_2Zn (38.75 mmol, 1.55 eq) was added dropwise within 30 min. The mixture was let warm up to room temperature and stirred overnight. In case of incomplete conversion (monitored via ^1H NMR), an additional portion (1.0 eq) of CH_2I_2 can be added. [The reaction can take multiple days and several additions of CH_2I_2 until completion.] The mixture was quenched at 0 °C with water. The precipitate was separated on Büchner funnel under *vacuo* and washed with DCM. The organic phase was separated using extraction funnel. The water phase was washed 2 times with DCM. Combined organic phases were dried over anh. $MgSO_4$ and concentrated under *vacuo*. The obtained liquid was directly submitted to the next step.
3. The silyl ether was transferred into a 100 ml Schlenk flask (pre-dried and backfilled with Ar three times) via syringe. Ar was evacuated until 1.0-2.0 mbar and backfilled three times. Next the substrate was dissolved in MeOH (50 ml, 0.5M). Upon cooling with an ice-water bath, 1-5 drops of TMSCl were added to the solution. The mixture was left stirring overnight at room temperature. After completion (monitored via ^1H NMR), the solvent was evaporated under *vacuo*. The product was isolated using column chromatography (SiO_2/hexane, hexane-EtOAc). In case of insufficient purity crystalline compounds can be recrystallized from hot hexane.

3.2. General procedure 2. Kulinkovich reaction

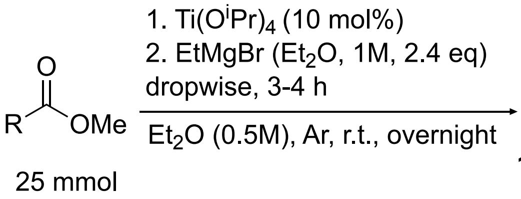

1g, 1i, 1j, 1l, 1m, 1n, 1q, 1t, 1u

A 250 ml Schlenk flask was pre-dried under *vacuo* with a propane torch/ a heat gun and backfilled with Ar (2-3 iterations). After cooling to r.t. the flask was charged with Et_2O (50 ml, 0.5M), the corresponding ester (25 mmol) and $Ti(OPr^i)_4$ (2.5 mmol, 10 mol%). To the obtained mixture 1M solution of EtMgBr in Et_2O (60 mmol, 2.4 eq) was added dropwise within 3 h at r.t. (water bath). The mixture was left stirring overnight. After reaction completion (^1H NMR), the mixture was quenched with water at 0°C (ice-water bath). The formed precipitate was filtered using Büchner funnel under vacuo and washed with Et_2O. The water phase was washed 2 times with Et_2O. Combined organic phases were washed once with brine and dried over anhydrous $MgSO_4$. After filtration the solvent was pulled out under vacuo. The obtained oil was purified using column chromatography (SiO_2/hexane, hexane-EtOAc). In case of insufficient purity crystalline compounds can be recrystallized from hot hexane.

4. Coupling of cyclopropanols with S-phenyl-substituted thioesters

4.1. Possible reaction pathways

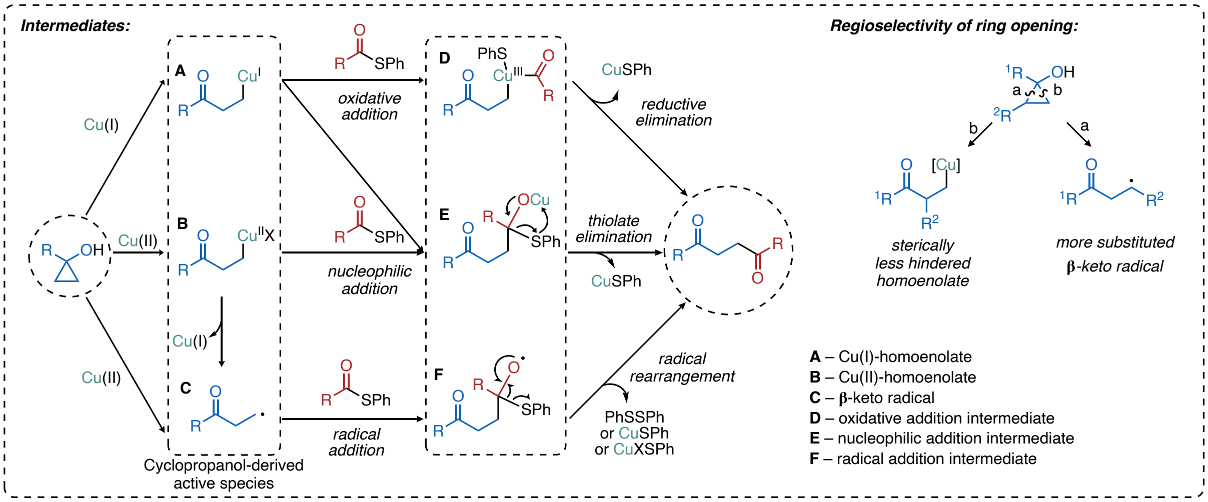

4.2. Optimization

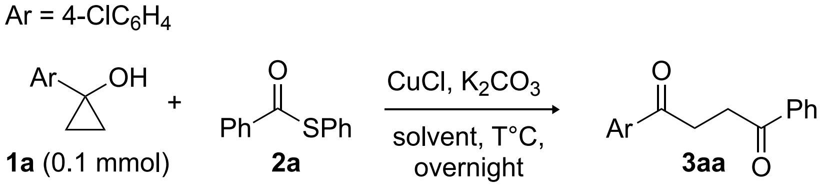

A regular Schlenk tube (or a pressure tube) was pre-dried with a heat gun/propane torch under *vacuo* and backfilled with argon (3 iterations). After cooling down to r.t. the tube was transferred into glovebox and charged with **1a** (0.1 mmol), **2a**, CuCl, K_2CO_3 and additional components, if stated. Next, the tube was transferred outside, and the solvent was added via syringe. The mixture was heated (aluminum heating block) overnight with vigorous stirring. The mixture was cooled down, quenched with 5-10 ml of H_2O, diluted with 1-3 ml of EtOAc and 50 µL of stock solution (1,3,5-trimethoxybenzene/EtOAc, ca. 0.62M). The mixture was stirred, and aliquot was taken from the organic phase. Solvent was evaporated under *vacuo* and NMR sample in $CDCl_3$ was prepared.

Table S1. Optimization of the developed reaction

Entry	2, eq	CuCl, eq	K_2CO_3, eq	T, °C	Solvent	C, M	3, %[a]
1[b]	1,5	1,5	1	60	MeCN	0,1	77
2	1,5	1,5	1	60	MeCN	0,1	81
3	1,5	1,5	1	60	MeCN	0,2	82
4	1,5	1,5	1	60	MeCN	0,05	88
5	2	2	1	60	MeCN	0,1	85
6	2	1,5	1	60	MeCN	0,1	87
7	1,5	2	1	60	MeCN	0,1	82
8[c]	2	2	1	60	MeCN	0,1	87
9	1,5	1,5	1	50	MeCN	0,1	71
10	1,5	1,5	1	80	MeCN	0,1	92
11	1,5	1,5	1	90	MeCN	0,1	93

12	2	2	2	60	MeCN	0,1	92
13	2	2	2	80	MeCN	0,1	96
14	1,5	1,5	2	80	MeCN	0,1	95
15	1,5	-	1	80	MeCN	0,1	5
16	1,5	0,2	1	100	MeCN	0,1	24
17	1,5	0,2	1	120	DMF	0,1	19

[a]The yields are calculated via ^{1}H NMR using 1,3,5-trimethoxybenzene as internal standard; [b]In presence of $Pd(OAc)_2$ (5 mol%) and 1,4-bis(diphenylphosphanyl)butane (5 mol%); [c]In presence of 1,4-bis(diphenylphosphanyl)butane (10 mol%).

Performing the reaction with 1.5 eq of CuCl and 1.0 eq of K_2CO_3 as a base in acetonitrile at 60°C led to product formation with 81% (Table S1, entry 2). Notably, usage of palladium catalyst did not exert a positive effect on the reaction yield (Table S1, entry 1). Concentration screening revealed that the system is not demanding towards this parameter (Table S1, entry 2, 3 and 4). Whereas, increasing the temperature to 80 °C helped to reach 92% (Table S1, entry 10). Since further increase was not beneficial (Table S1, entry 11) for the model substrate, we were satisfied with the obtained result and finished optimization at this point.

4.3. General procedure 3. Synthesis of 1,4-diketones

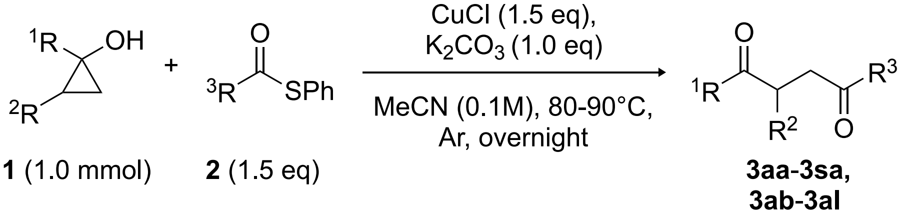

A pressure tube was pre-dried with a heat gun/propane torch under *vacuo* and backfilled with argon (3 iterations). After cooling down to r.t. the tube was transferred into glovebox and charged with **1** (1.0 mmol), **2** (1.5 eq), CuCl (1.5 eq), K_2CO_3 (1.0 eq). Next, the tube was transferred outside and MeCN (10 ml) was added via syringe. The mixture was heated at 80 °C or 90 °C (aluminum heating block) overnight with vigorous stirring. The mixture was cooled down, transferred into 250 ml round-bottom flask with EtOAc, 2.5 teaspoons of celite were added and solvent was evaporated under *vacuo*. Obtained powder was submitted to flash chromatography. The product was isolated using EtOAc/hexane mixtures as eluent.

Scheme S1. Limitations of the developed method

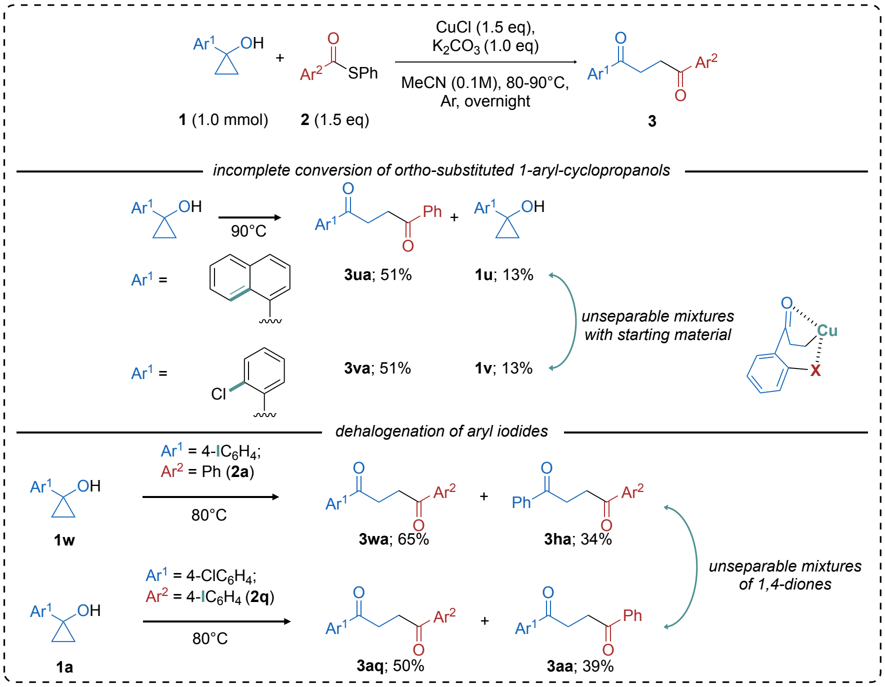

It was not possible to isolate compounds **3ua** and **3va** due to low conversion of the corresponding starting materials **1u** and **1v** (close Rf values). It can be assumed, that π-donating substituent in the *ortho*-position of the phenyl ring can coordinate at the Cu-center of the homoenolate leading to its lower reactivity due to increased steric hindrance. Diketones **3wa** and **3aq** were obtained as unseparable mixtures with the deiodinated derivatives **3ha** and **3aa**, respectively.

Scheme S2. Alternative electrophiles

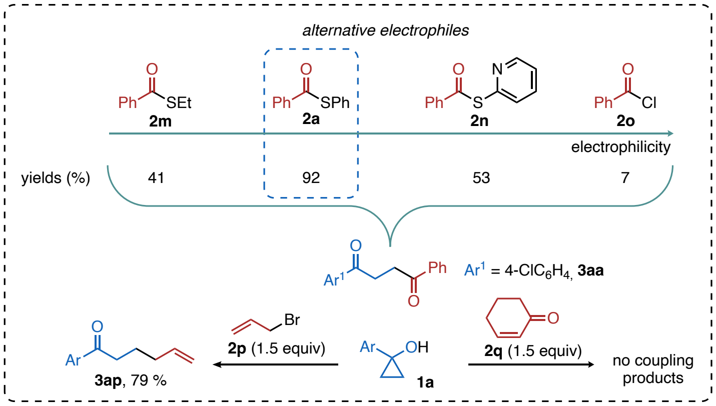

5. Reaction progress

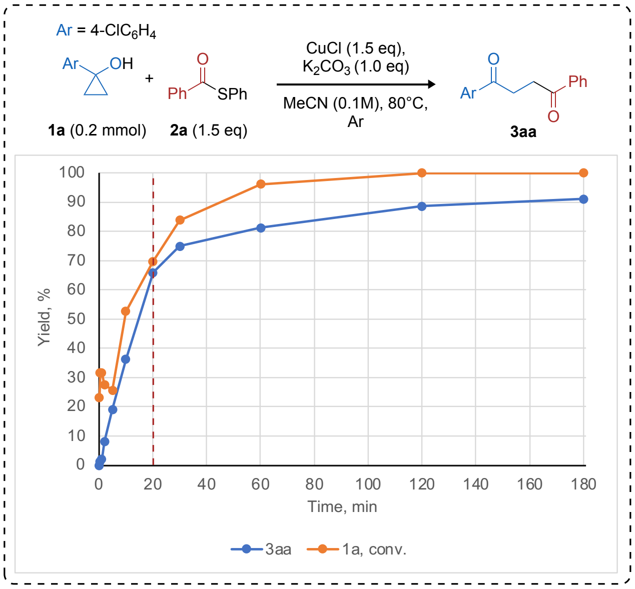

The reaction progress curve was obtained via a reaction series monitored with ^{1}H NMR using internal standard (1,3,5-trimethoxybenzene). To minimize errors a stock solution containing **1a**, **2a**, CuCl and 1,3,5-trimethoxybenzene was prepared in glovebox. The solution was distributed between Schlenk pressure tubes, which were next taken outside. Then K_2CO_3 was added as solid, and the tubes were immediately installed into heated at 80 °C aluminum block. At the very same moment the timer was set up. After certain time interval the tube was placed into water-ice bath and the mixture was quenched with cold water as quickly as possible. The corresponding time was noted down. An aliquot from organic phase was taken, filtered, concentrated under *vacuo* and submitted to ^{1}H NMR.

Table S3. Kinetic curve values

Entry	Time, min	Yield **3aa**, %	Conversion **1a**, %
1	0,22	0	23
2	0,53	1	31
3	1,05	2	31
4	2,08	8	28
5	5,07	19	26
6	10,07	36	53
7	20,08	66	70
8	30,10	75	84
9	60,08	81	96
10	120,07	89	100
11	180,08	91	100

6. Synthesized cyclopropanols

1-(4-chlorophenyl)cyclopropan-1-ol

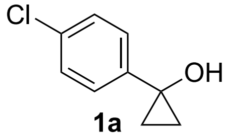

1a

1a was synthesized following the general procedure **1** and isolated via column chromatography (SiO_2/*n*-hexane, EtOAc/*n*-hexane 1/10, EtOAc/*n*-hexane 1/5) in 85% (3565 mg) yield as a colourless solid.

^{1}H NMR (300 MHz, $CDCl_3$): δ = 7.33 – 7.27 (m, 2H), 7.25 – 7.19 (m, 2H), 2.31 (d, *J* = 3.1 Hz, 1H), 1.33 – 1.23 (m, 2H), 1.07 – 0.97 (m, 2H). The spectroscopic data are consistent with those previously reported. [17]

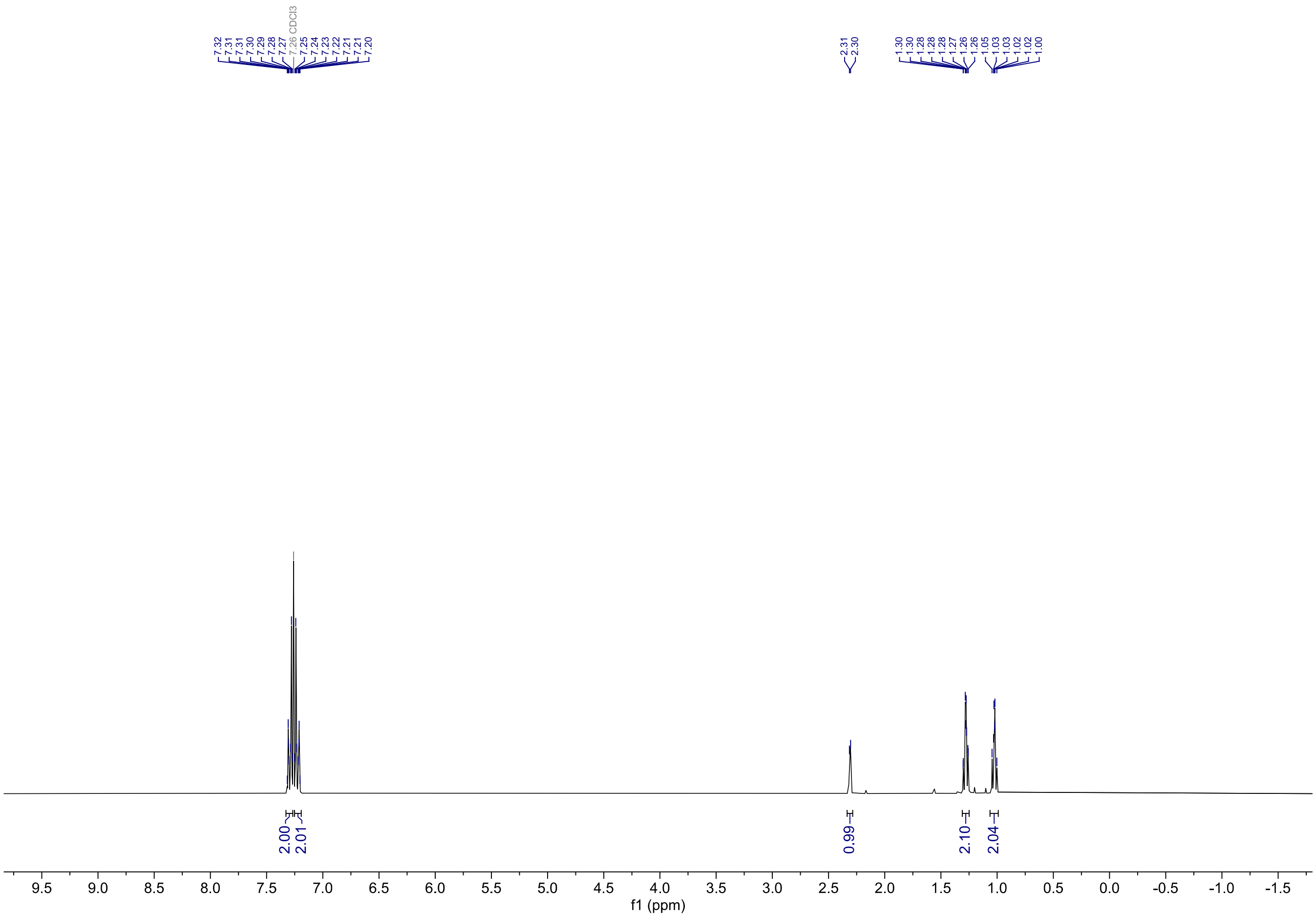

Figure S1. ^{1}H NMR of 1-(4-chlorophenyl)cyclopropan-1-ol (**1a**) in $CDCl_3$ measured at 300 MHz.

1-(4-fluorophenyl)cyclopropan-1-ol

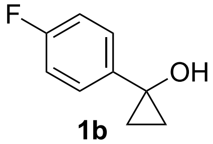

1b

1b was synthesized following the general procedure **1** and isolated via column chromatography (SiO_2/*n*-hexane, EtOAc/*n*-hexane 1/10, EtOAc/*n*-hexane 1/5) in 82% (3124 mg) yield as a colourless solid.

^{1}H NMR (400 MHz, $CDCl_3$): δ = 7.24 (dd, *J* = 8.8, 5.4 Hz, 2H), 6.97 (t, *J* = 8.7 Hz, 2H), 2.40 (s, 1H), 1.23 – 1.17 (m, 2H), 0.96 (q, *J* = 5.1 Hz, 2H). The spectroscopic data are consistent with those previously reported. [18]

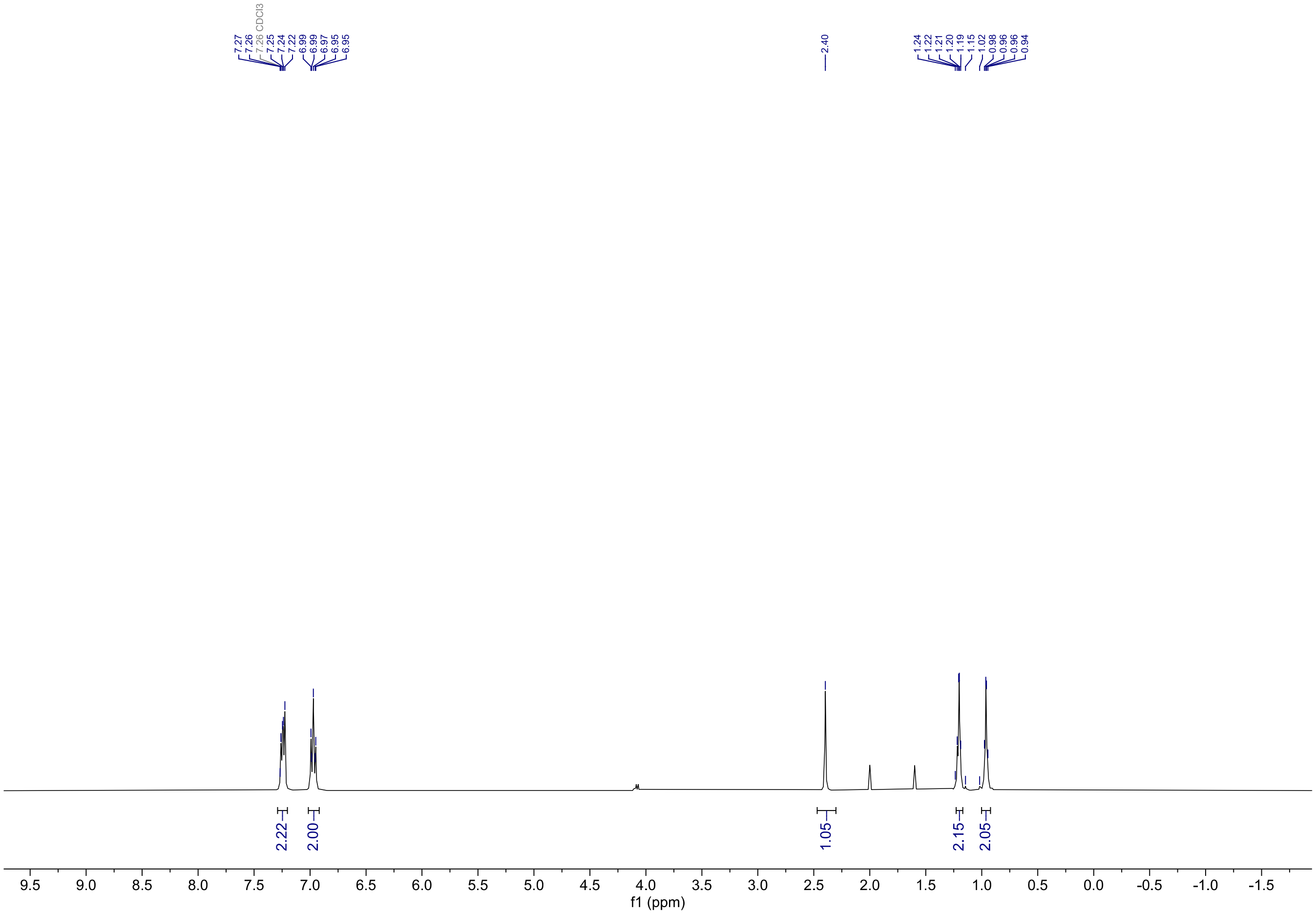

Figure S2. ^{1}H NMR of 1-(4-fluorophenyl)cyclopropan-1-ol (**1b**) in $CDCl_3$ measured at 400 MHz.

1-(4-bromophenyl)cyclopropan-1-ol

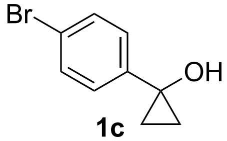

1c

1c was synthesized following the general procedure **1** and isolated via column chromatography (SiO_2/*n*-hexane, EtOAc/*n*-hexane 1/10, EtOAc/*n*-hexane 1/5) in 85% (4528 mg) yield as a colourless solid.

^{1}H NMR (400 MHz, $CDCl_3$): δ = 7.43 (d, *J* = 8.5 Hz, 2H), 7.16 (d, *J* = 8.6 Hz, 2H), 2.46 (s, 1H), 1.31 – 1.25 (m, 2H), 1.02 (t, *J* = 3.7 Hz, 2H). The spectroscopic data are consistent with those previously reported. [18]

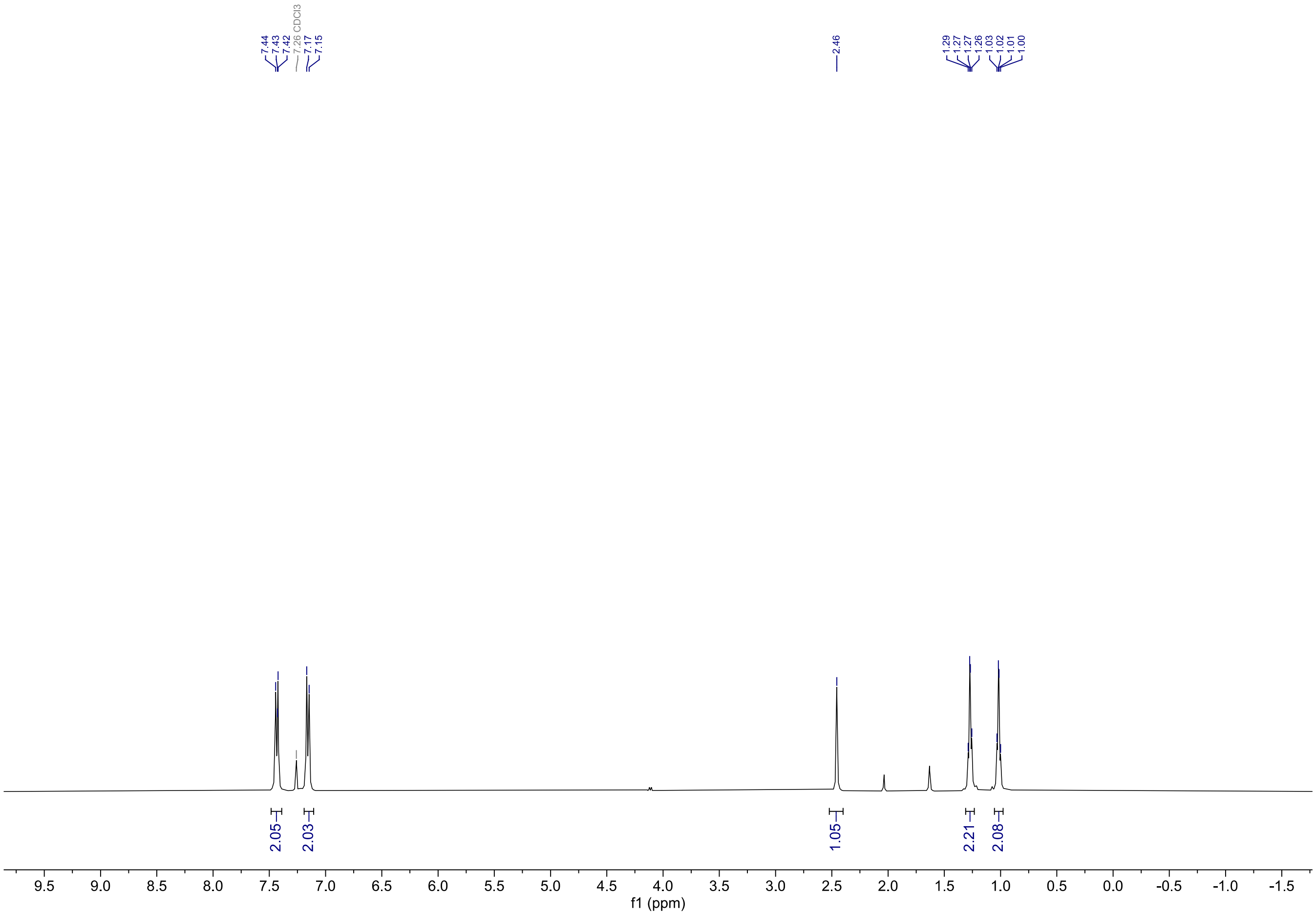

Figure S3. ^{1}H NMR of 1-(4-bromophenyl)cyclopropan-1-ol (**1c**) in $CDCl_3$ measured at 400 MHz.

1-(4-methoxyphenyl)cyclopropan-1-ol

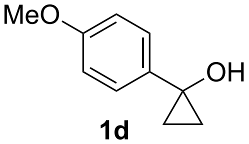

1d

1d was synthesized following the general procedure **1** and isolated via column chromatography (SiO_2/*n*-hexane, EtOAc/*n*-hexane 1/10, EtOAc/*n*-hexane 1/5) in 56% (2316 mg) yield as a pale yellow solid.

^{1}H NMR (400 MHz, $CDCl_3$): δ = 7.30 – 7.25 (m, 2H), 6.91 – 6.84 (m, 2H), 3.80 (s, 3H), 2.26 (s, 1H), 1.24 – 1.16 (m, 2H), 1.01 – 0.94 (m, 2H). The spectroscopic data are consistent with those previously reported. [17]

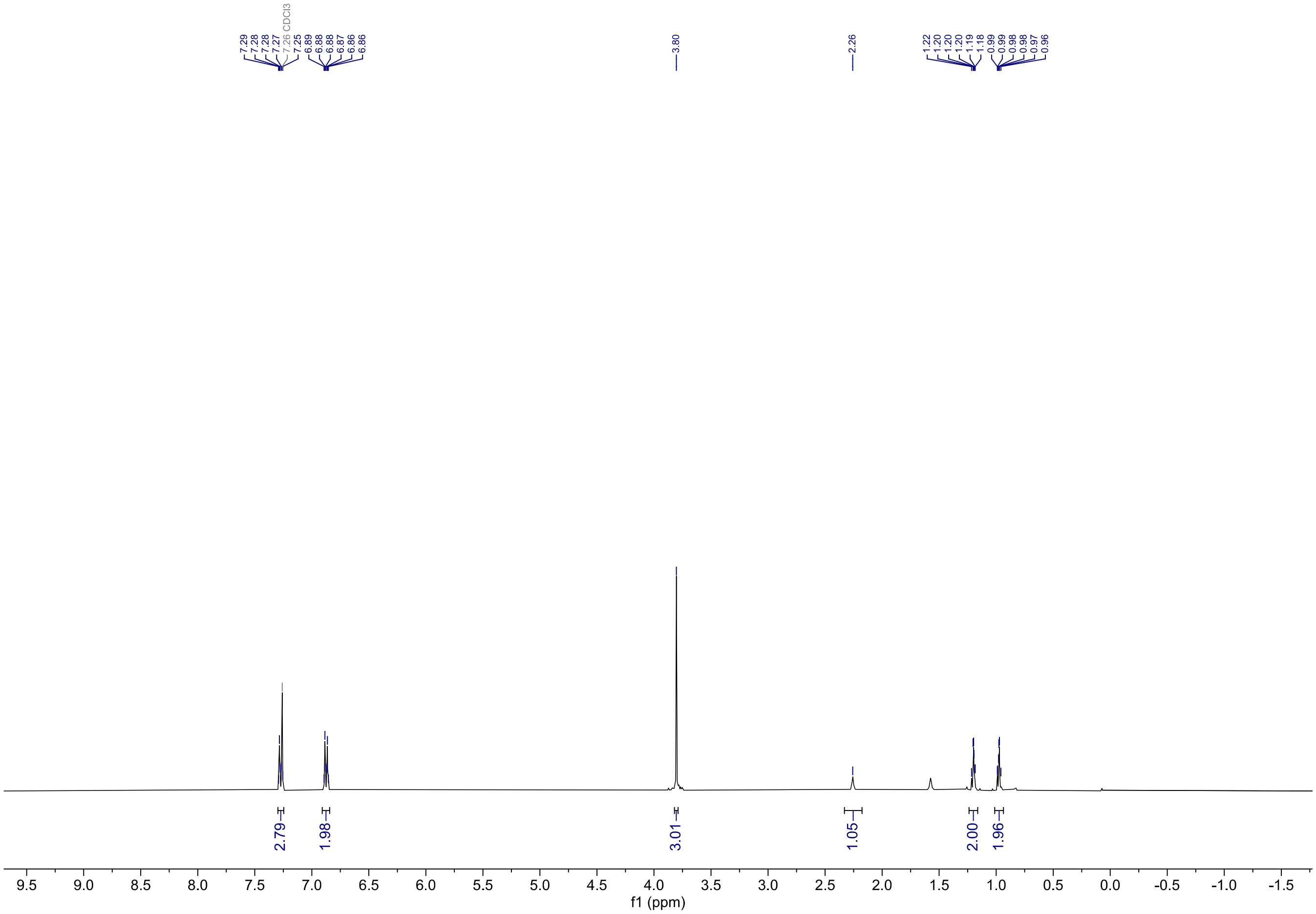

Figure S4. ^{1}H NMR of 1-(4-methoxyphenyl)cyclopropan-1-ol (**1d**) in $CDCl_3$ measured at 400 MHz.

1-(4-(*tert*-butyl)phenyl)cyclopropan-1-ol

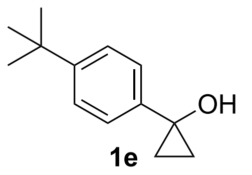

1e was synthesized following the general procedure **1** and isolated via column chromatography (SiO_2/*n*-hexane, EtOAc/*n*-hexane 1/10, EtOAc/*n*-hexane 1/5) in 96% (4556 mg) yield as a colourless solid.

^{1}H NMR (400 MHz, $CDCl_3$): δ = 7.40 – 7.34 (m, 2H), 7.29 – 7.23 (m, 2H), 2.31 (d, J = 5.5 Hz, 1H), 1.33 (s, 9H), 1.26 – 1.21 (m, 2H), 1.07 – 1.00 (m, 2H). The spectroscopic data are consistent with those previously reported. [17]

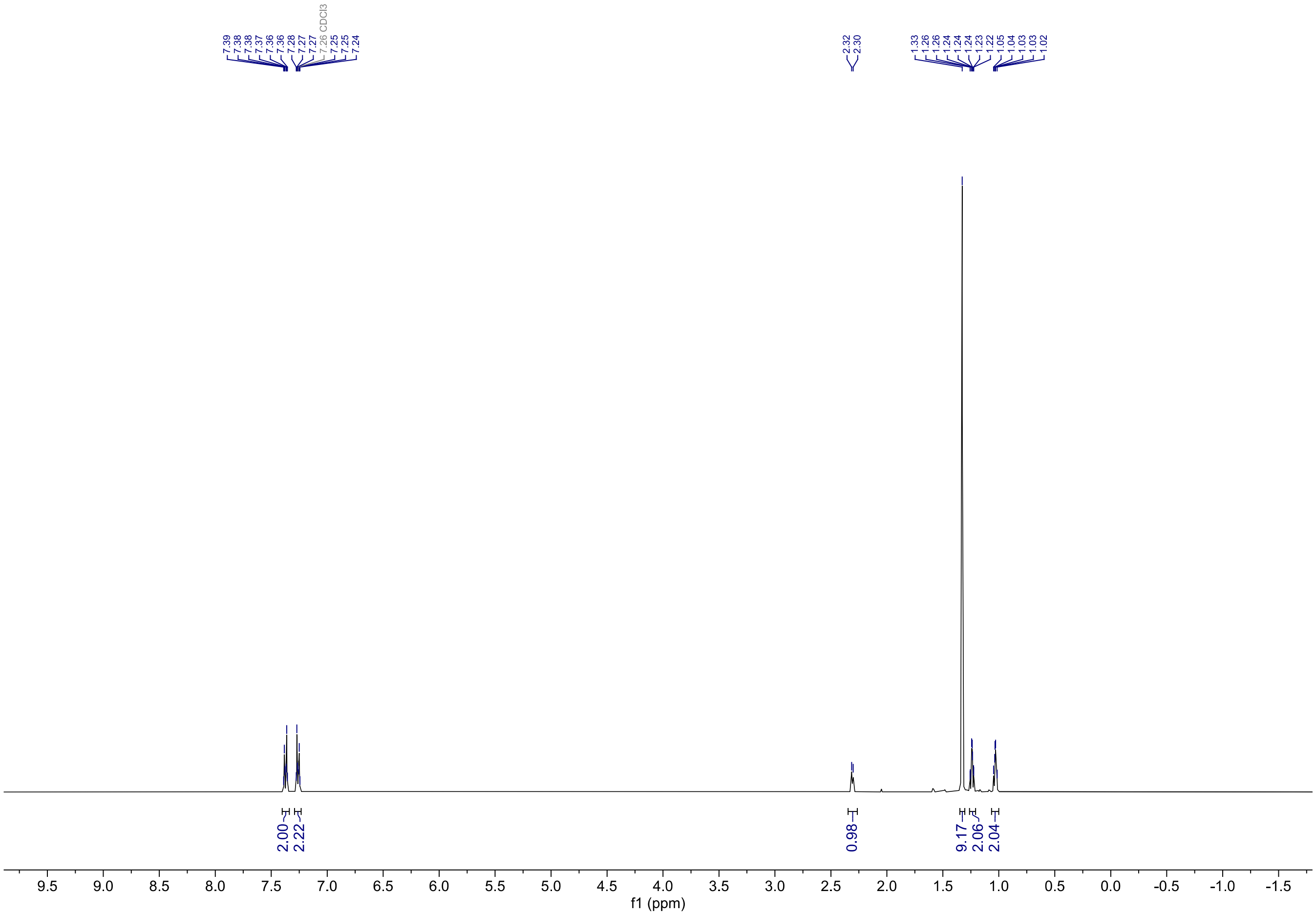

Figure S5. ^{1}H NMR of 1-(4-methoxyphenyl)cyclopropan-1-ol (**1e**) in $CDCl_3$ measured at 400 MHz.

1-(4-(trifluoromethyl)phenyl)cyclopropan-1-ol

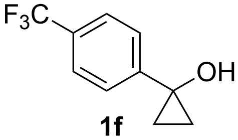

1f was synthesized following the general procedure **1** and isolated via column chromatography (SiO_2/*n*-hexane, EtOAc/*n*-hexane 1/10, EtOAc/*n*-hexane 1/5) in 73% (3684 mg) yield as a yellowish solid.

^{1}H NMR (400 MHz, $CDCl_3$): δ = 7.62 – 7.55 (m, 2H), 7.41 – 7.34 (m, 2H), 2.35 (s, 1H), 1.40 – 1.32 (m, 2H), 1.15 – 1.07 (m, 2H). The spectroscopic data are consistent with those previously reported. [17]

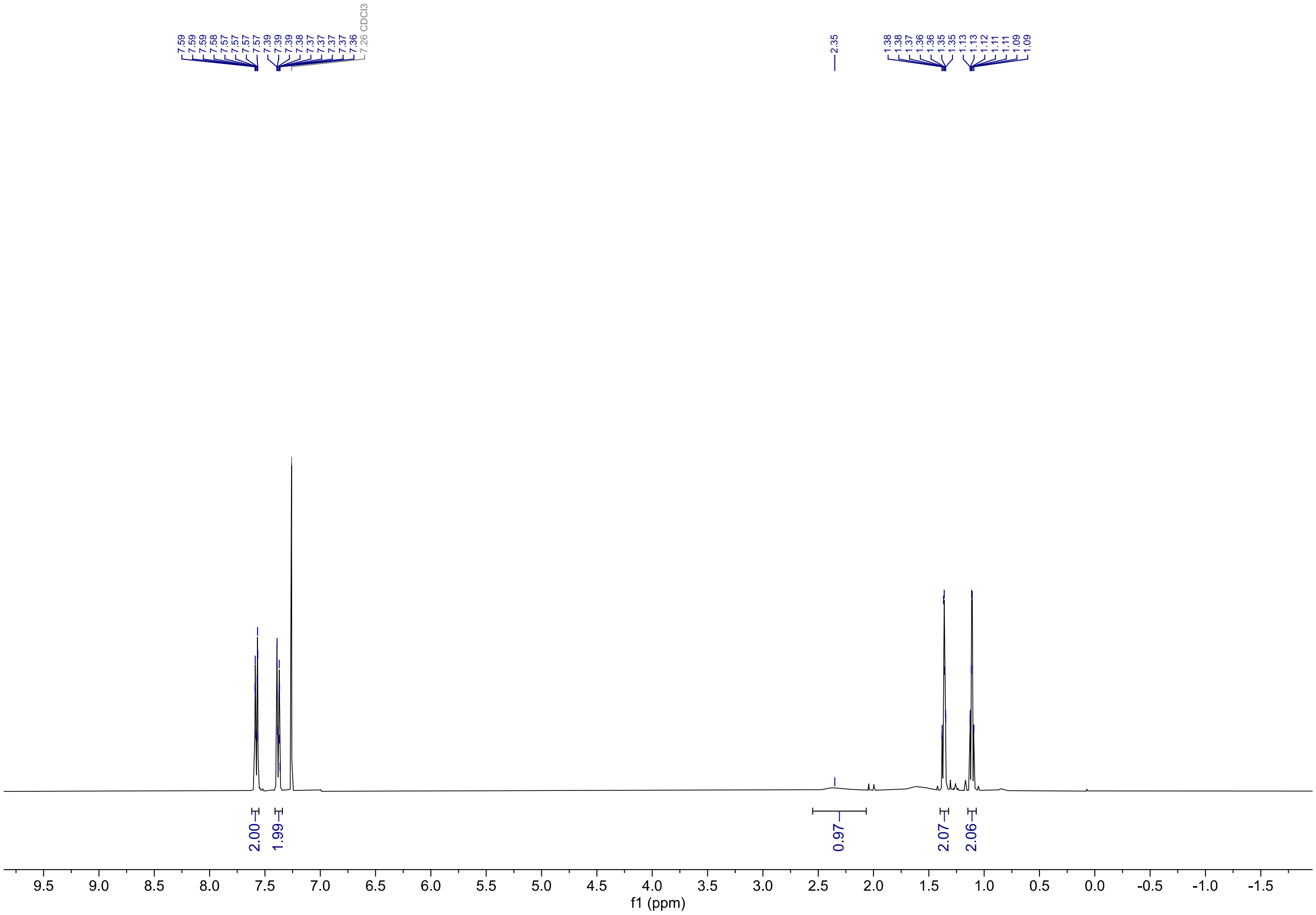

Figure S6. ^{1}H NMR of 1-(4-(trifluoromethyl)phenyl)cyclopropan-1-ol (**1f**) in $CDCl_3$ measured at 400 MHz.

1-(naphthalen-2-yl)cyclopropan-1-ol

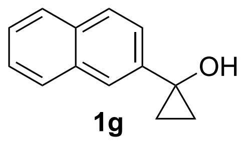

1g

1g was synthesized following the general procedure **1** and isolated via column chromatography (SiO_2/*n*-hexane, EtOAc/*n*-hexane 1/10, EtOAc/*n*-hexane 1/6) in 74% (3408 mg) yield as a yellowish solid.

^{1}H NMR (400 MHz, $CDCl_3$): δ = 7.91 – 7.72 (m, 4H), 7.52 – 7.41 (m, 2H), 7.31 (dd, *J* = 8.7, 1.9 Hz, 1H), 2.48 (d, *J* = 10.3 Hz, 1H), 1.38 – 1.31 (m, 2H), 1.20 – 1.13 (m, 2H). The spectroscopic data are consistent with those previously reported. [17]

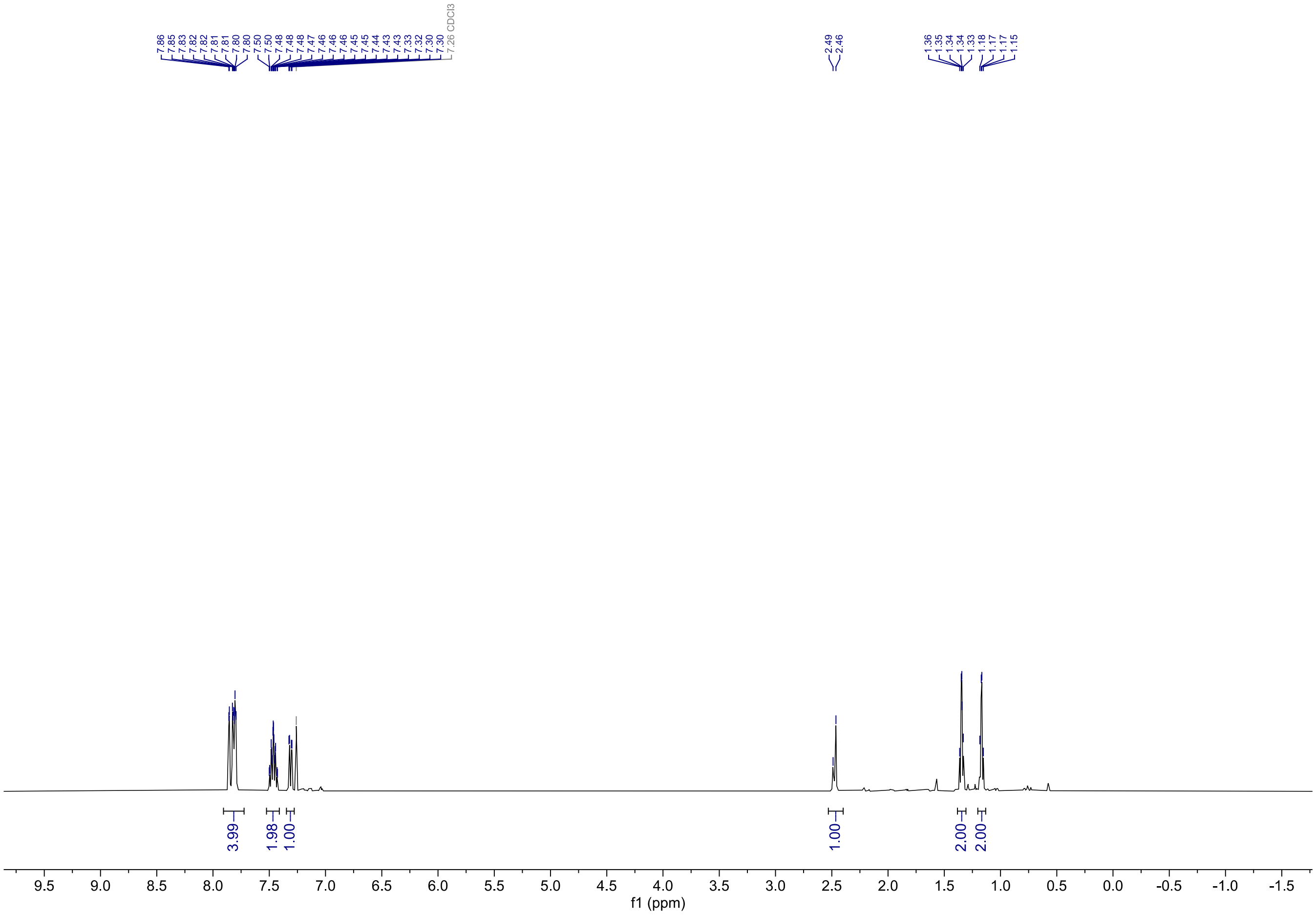

Figure S7. ^{1}H NMR of 1-(naphthalen-2-yl)cyclopropan-1-ol (**1g**) in $CDCl_3$ measured at 400 MHz.

1-phenylcyclopropan-1-ol

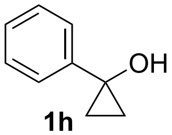

1h

1h was synthesized following the general procedure **1** and isolated via column chromatography (SiO_2/*n*-hexane, EtOAc/*n*-hexane 1/10, EtOAc/*n*-hexane 1/5) in 81% (2723 mg) yield as a yellowish solid.

^{1}H NMR (400 MHz, $CDCl_3$): δ = 7.38 – 7.29 (m, 4H), 7.23 (m, 1H), 2.32 (s, 1H), 1.29 – 1.25 (m, 2H), 1.09 – 1.02 (m, 2H). The spectroscopic data are consistent with those previously reported. [17]

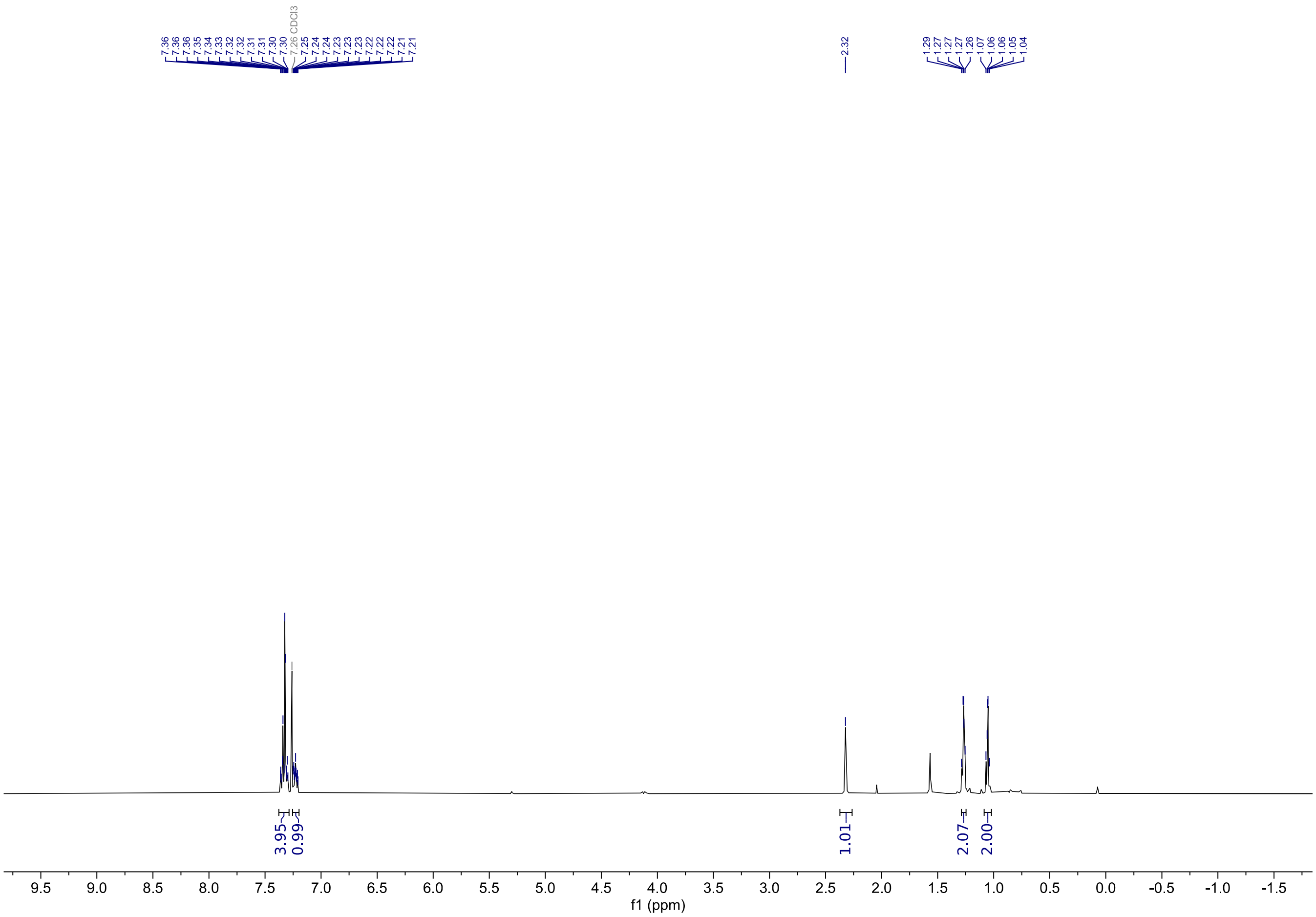

Figure S8. ^{1}H NMR of 1-phenylcyclopropan-1-ol (**1h**) in $CDCl_3$ measured at 400 MHz.

1-phenethylcyclopropan-1-ol

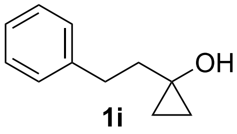

1i was synthesized following the general procedure **2** and isolated via column chromatography (SiO_2/*n*-hexane, EtOAc/*n*-hexane 1/10, EtOAc/*n*-hexane 1/5) in 77% (3108 mg) yield as a colourless solid.

^{1}H NMR (400 MHz, $CDCl_3$): δ = 7.30 (dd, *J* = 8.0, 6.7 Hz, 2H), 7.26 – 7.16 (m, 3H), 2.92 – 2.83 (m, 2H), 2.01 (s, 1H), 1.93 – 1.85 (m, 2H), 0.82 – 0.74 (m, 2H), 0.52 – 0.44 (m, 2H). The spectroscopic data are consistent with those previously reported.[17]

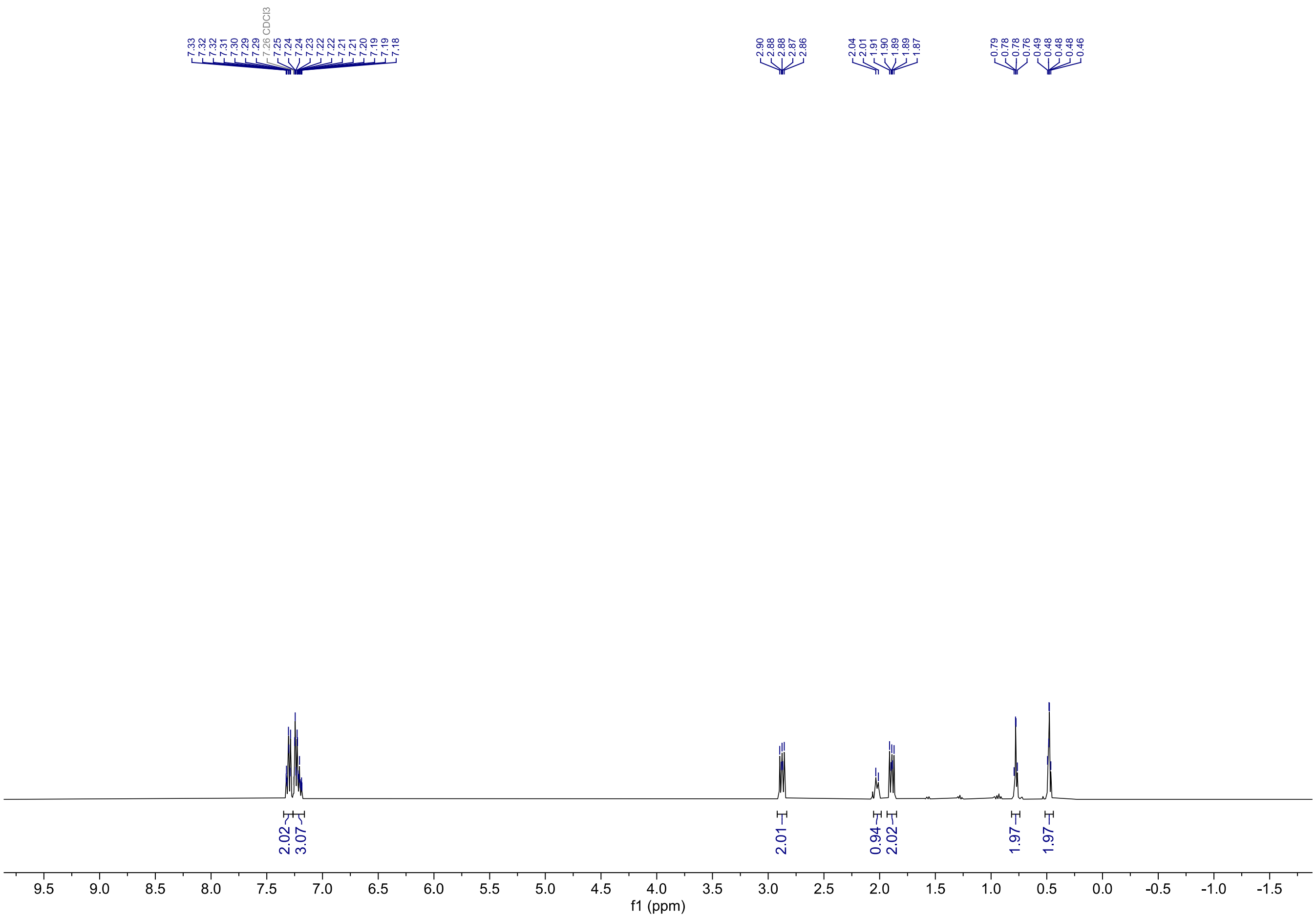

Figure S9. ^{1}H NMR of 1-phenethylcyclopropan-1-ol (**1i**) in $CDCl_3$ measured at 400 MHz.

1-benzhydrylcyclopropan-1-ol

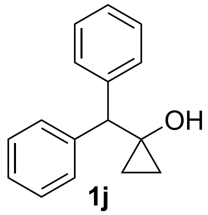

1j was synthesized following the general procedure **2** and isolated via column chromatography (SiO_2/*n*-hexane, EtOAc/*n*-hexane 1/10, EtOAc/*n*-hexane 1/5). Recrystallized from hot hexane in 72% (4018 mg) yield (colourless solid).

^{1}H NMR (400 MHz, $CDCl_3$): δ = 7.40 – 7.30 (m, 8H), 7.28 – 7.22 (m, 2H), 3.89 (s, 1H), 2.04 (s, 1H), 0.97 – 0.91 (m, 2H), 0.73 – 0.66 (m, 2H). The spectroscopic data are consistent with those previously reported. [17]

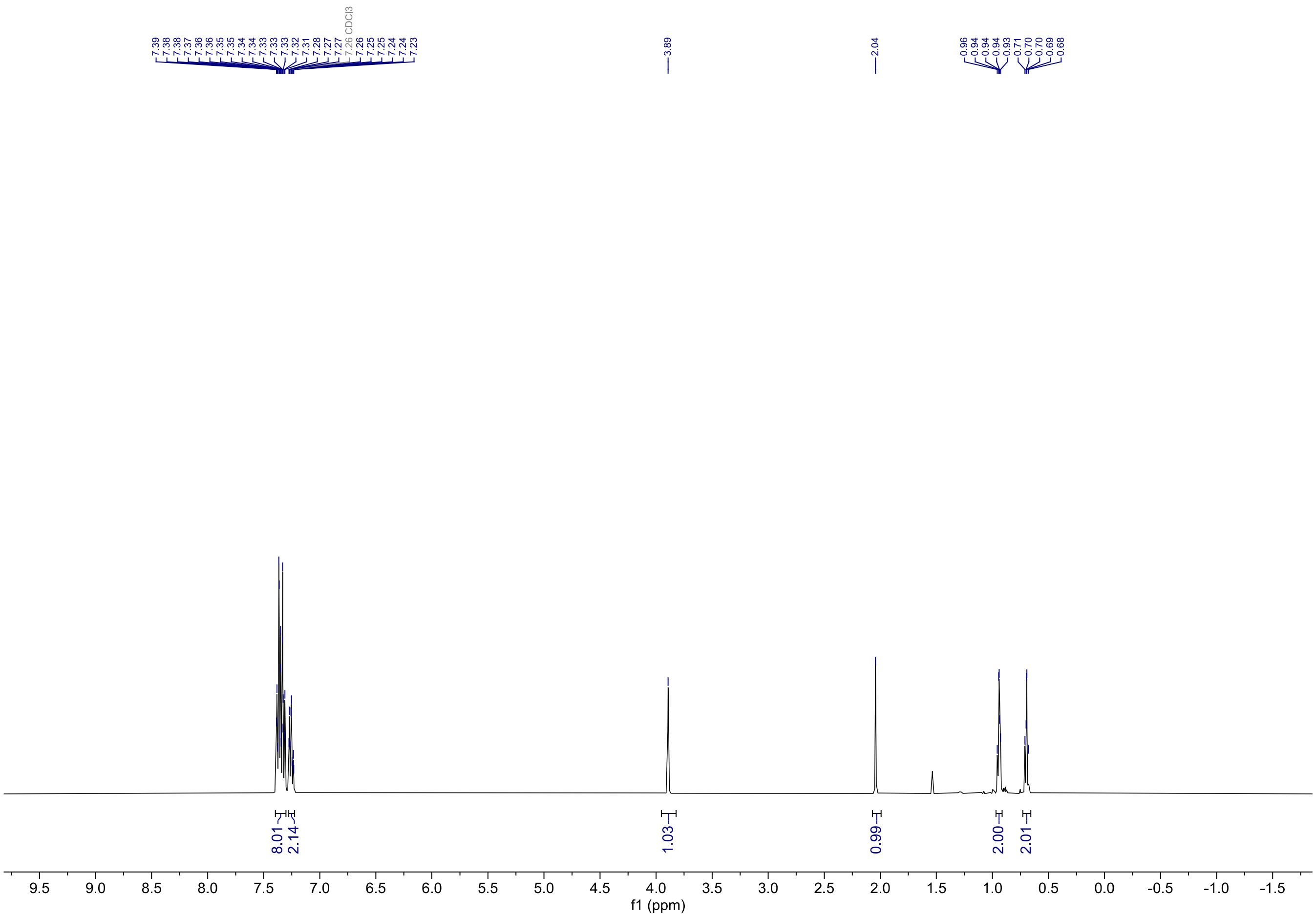

Figure S10. ^{1}H NMR of 1-benzhydrylcyclopropan-1-ol (**1j**) in $CDCl_3$ measured at 400 MHz.

1-cyclohexylcyclopropan-1-ol

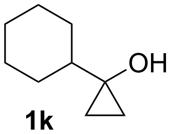

1k

1k was synthesized following the general procedure **2** and isolated via column chromatography (SiO_2/*n*-hexane, EtOAc/*n*-hexane 1/10, EtOAc/*n*-hexane 1/5) in 79% (2756 mg) yield as a colourless oil.

^{1}H NMR (400 MHz, $CDCl_3$): δ = 1.84 – 1.70 (m, 5H), 1.67 (m, 1H), 1.30 – 1.11 (m, 5H), 0.99 – 0.87 (m, 1H), 0.72 – 0.65 (m, 2H), 0.47 – 0.39 (m, 2H). The spectroscopic data are consistent with those previously reported. [18]

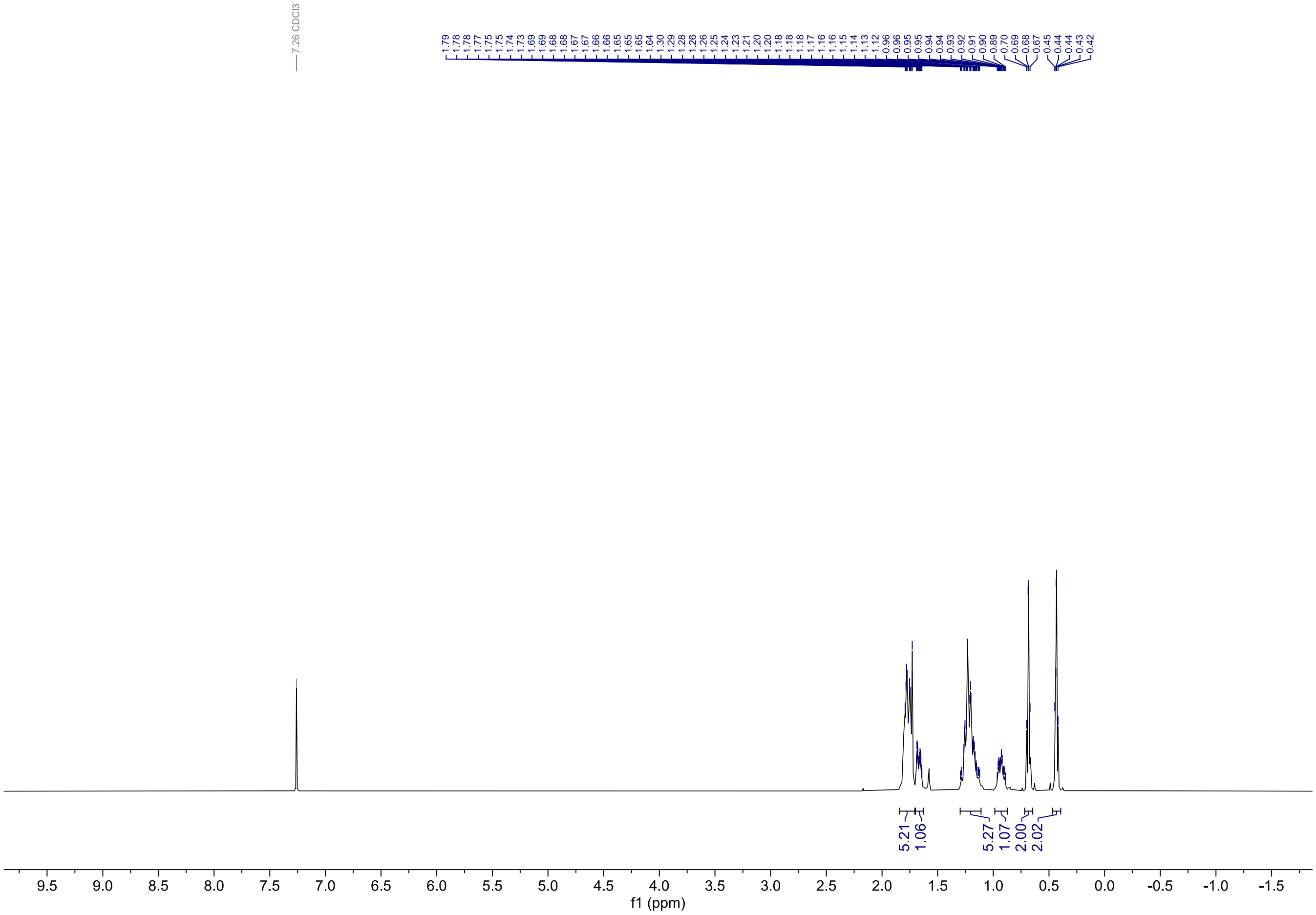

Figure S11. ^{1}H NMR of 1-cyclohexylcyclopropan-1-ol (**1k**) in $CDCl_3$ measured at 400 MHz.

1-cyclobutylcyclopropan-1-ol

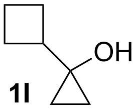

1l

1l was synthesized following the general procedure **2** and isolated via column chromatography (SiO_2/*n*-hexane, EtOAc/*n*-hexane 1/10, EtOAc/*n*-hexane 1/8) in 57% (1598 mg) yield as a colourless oil.

^{1}H NMR (400 MHz, $CDCl_3$): δ = 2.50 (tt, *J* = 10.7, 7.3 Hz, 1H), 2.01 – 1.71 (m, 7H), 0.73 – 0.66 (m, 2H), 0.53 – 0.46 (m, 2H). The spectroscopic data are consistent with those previously reported. [19]

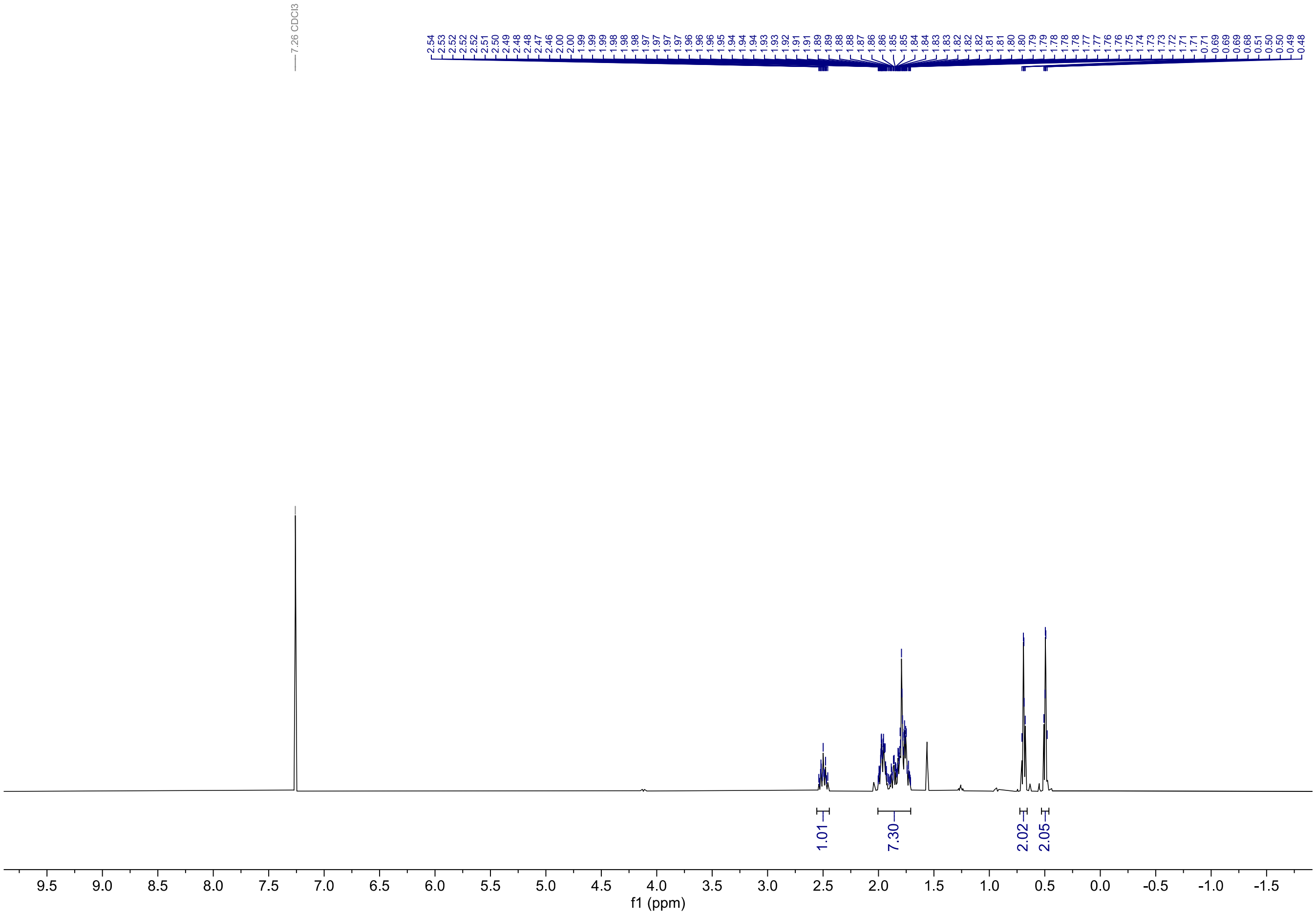

Figure S12. ^{1}H NMR of 1-cyclobutylcyclopropan-1-ol (**1l**) in $CDCl_3$ measured at 400 MHz.

***tert*-butyl 4-(1-hydroxycyclopropyl)piperidine-1-carboxylate**

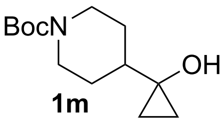

1m was synthesized following the general procedure **2** and isolated via column chromatography (SiO_2/*n*-hexane, EtOAc/*n*-hexane 1/5, EtOAc/*n*-hexane 1/3, EtOAc/*n*-hexane 1/3). Recrystallized from hot hexane in 21% (1263 mg) yield (colourless solid).

^{1}H NMR (400 MHz, $CDCl_3$): δ = 4.19 (s, 2H), 2.64 (t, *J* = 12.6 Hz, 2H), 1.82 – 1.75 (m, 1H), 1.68 (dd, *J* = 13.9, 3.2 Hz, 2H), 1.46 (s, 9H), 1.44 (m, 2H), 1.13 (tt, *J* = 12.1, 3.6 Hz, 1H), 0.77 – 0.71 (m, 2H), 0.51 – 0.44 (m, 2H). The spectroscopic data are consistent with those previously reported. [20]

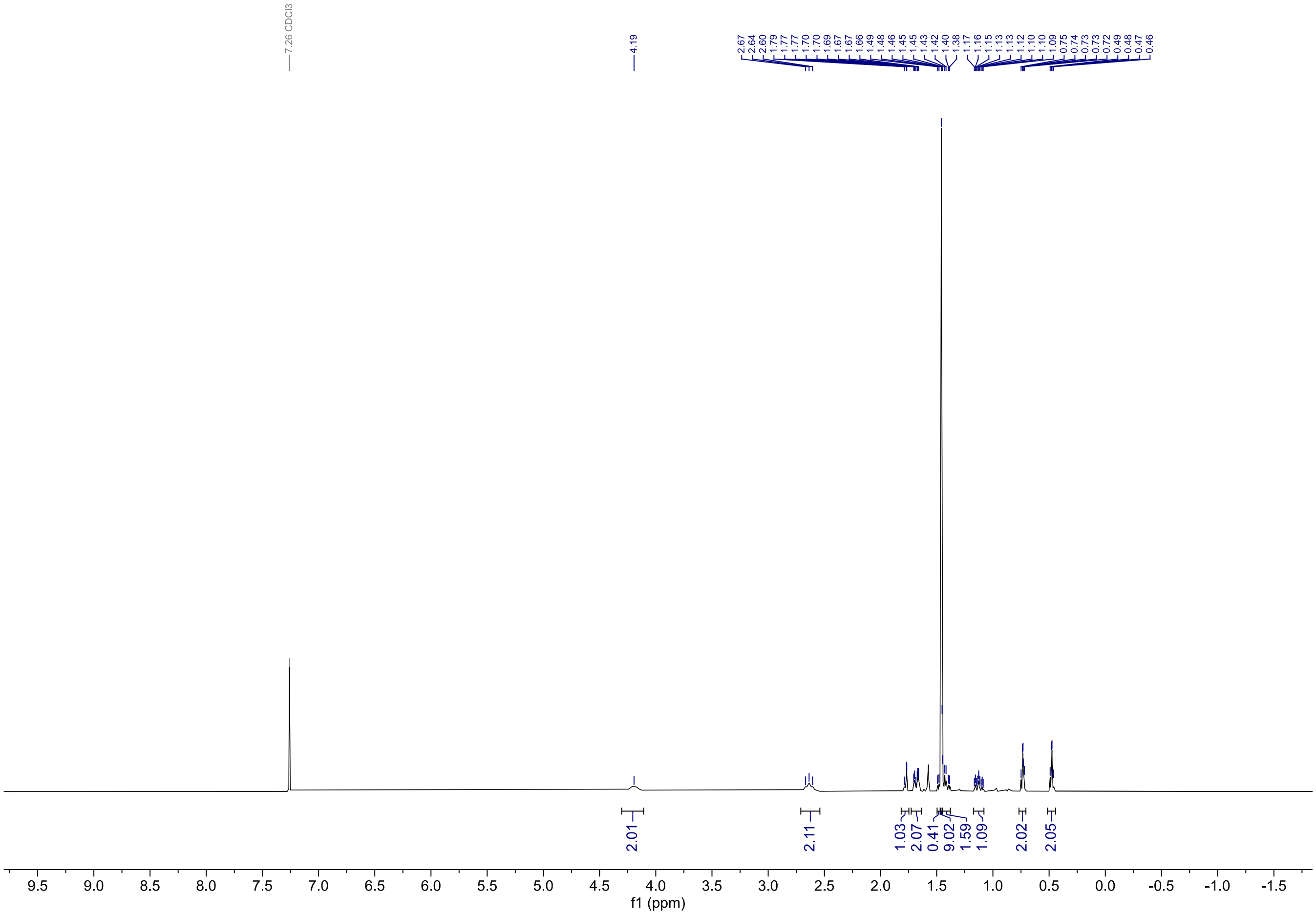

Figure S13. ^{1}H NMR of *tert*-butyl 4-(1-hydroxycyclopropyl)piperidine-1-carboxylate (**1m**) in $CDCl_3$ measured at 400 MHz.

1-(1-tosyl-1*H*-indol-2-yl)cyclopropan-1-ol

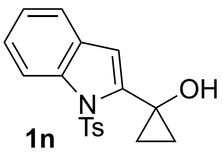

1n

1n was synthesized following the general procedure **2** and isolated via column chromatography (SiO_2/*n*-hexane, EtOAc/*n*-hexane 1/10, EtOAc/*n*-hexane 1/5) in 19% (1538 mg) yield as a pale ochre solid.

^{1}H NMR (400 MHz, $CDCl_3$): δ = 7.98 (d, *J* = 8.4 Hz, 1H), 7.76 (d, *J* = 8.1 Hz, 2H), 7.68 (dd, *J* = 7.9, 1.3 Hz, 1H), 7.47 (s, 1H), 7.32 (m, 1H), 7.26 – 7.16 (m, 3H), 2.48 – 2.37 (m, 1H), 2.33 (d, *J* = 1.8 Hz, 3H), 1.23 – 1.15 (m, 2H), 1.08 – 1.00 (m, 2H). The spectroscopic data are consistent with those previously reported. [21]

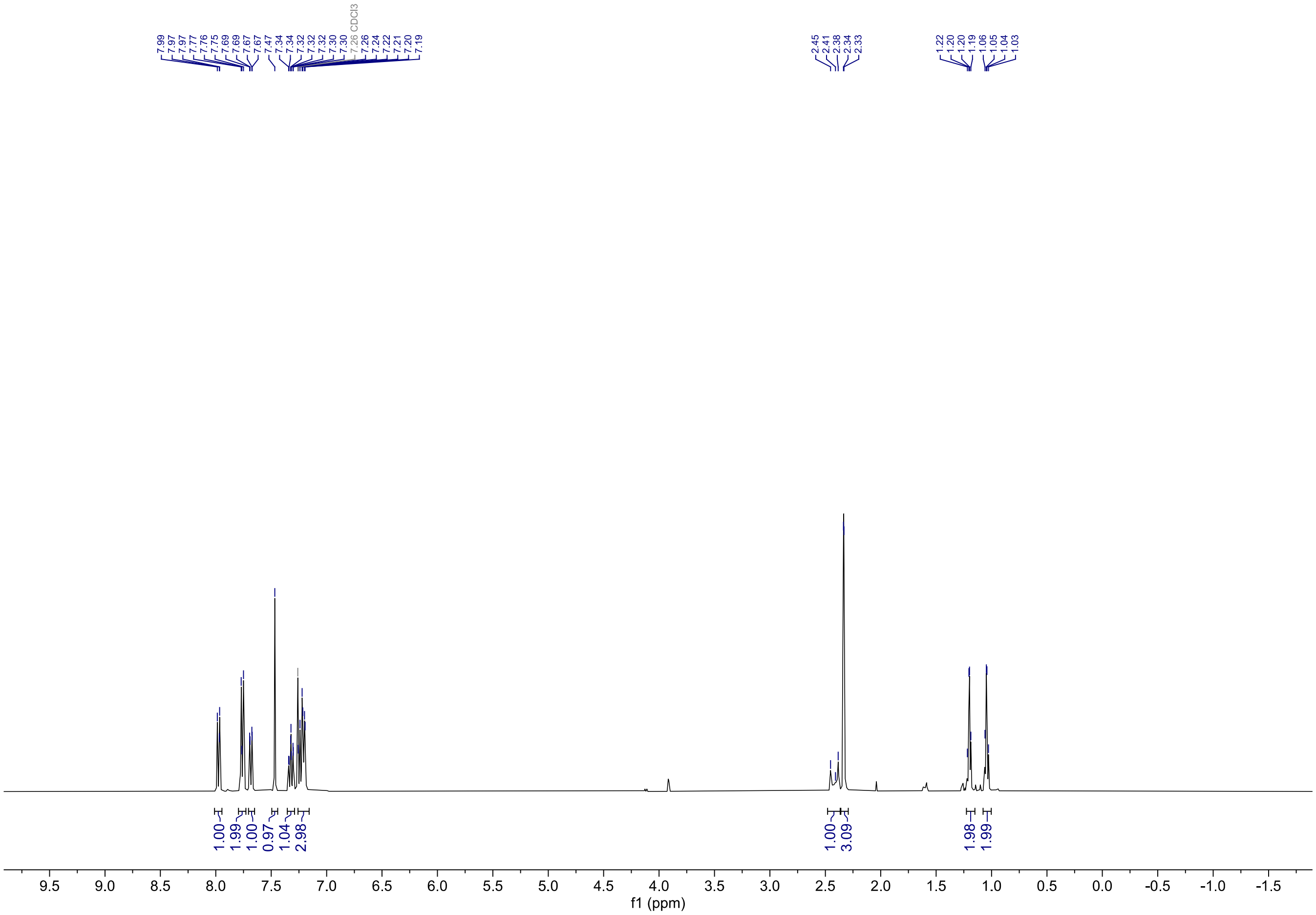

Figure S14. ^{1}H NMR of 1-(1-tosyl-1*H*-indol-2-yl)cyclopropan-1-ol (**1n**) in $CDCl_3$ measured at 400 MHz.

1,1a,2,3-tetrahydro-7b*H*-cyclopropa[*a*]naphthalen-7b-ol

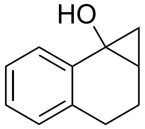

1o

1o was synthesized following the general procedure **1** and isolated via column chromatography (SiO_2/*n*-hexane, EtOAc/*n*-hexane 1/10, EtOAc/*n*-hexane 1/3) in 85% (3388 mg) yield as a pale yellow solid.

^{1}H NMR (400 MHz, $CDCl_3$): δ = 7.72 (dd, *J* = 7.7, 1.4 Hz, 1H), 7.27 (tt, *J* = 7.7, 1.3 Hz, 1H), 7.14 (td, *J* = 7.4, 1.4 Hz, 1H), 7.06 (dt, *J* = 7.4, 1.4 Hz, 1H), 2.71 – 2.55 (m, 1H), 2.53 – 2.22 (m, 2H), 2.06 – 1.95 (m, 1H), 1.82 – 1.70 (m, 2H), 1.23 (dd, *J* = 9.7, 5.8 Hz, 1H), 1.07 (t, *J* = 5.8 Hz, 1H). The spectroscopic data are consistent with those previously reported. [22]

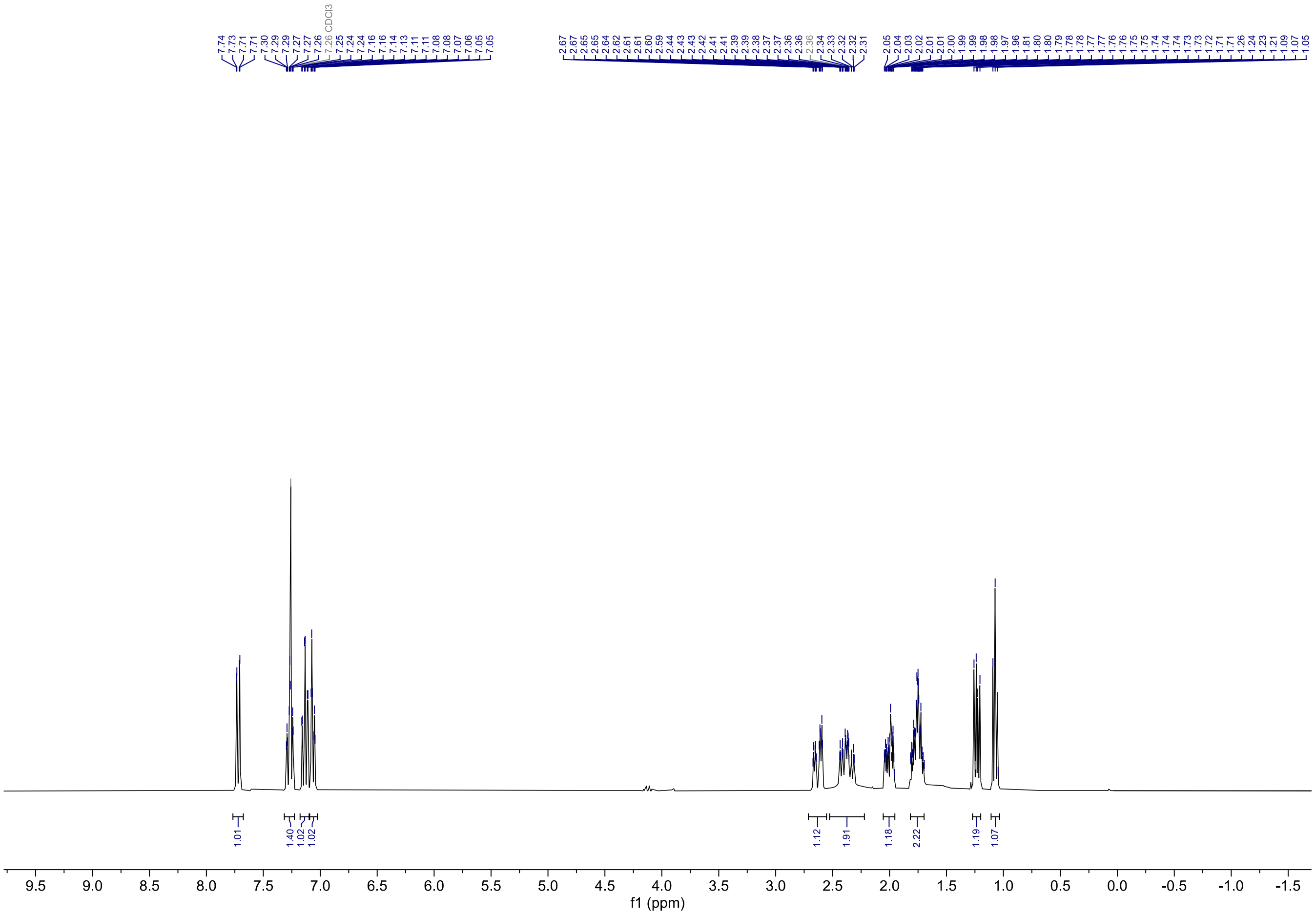

Figure S15. ^{1}H NMR of 1,1a,2,3-tetrahydro-7b*H*-cyclopropa[*a*]naphthalen-7b-ol (**1o**) in $CDCl_3$ measured at 400 MHz.

1a,2-dihydrocyclopropa[*c*]chromen-7b(1*H*)-ol

1p 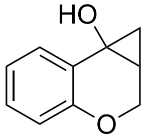

1p was synthesized following the general procedure **1** and isolated via column chromatography (SiO_2/*n*-hexane, EtOAc/*n*-hexane 1/10, EtOAc/*n*-hexane 1/3) in 20% (822 mg) yield as a pale yellow solid.

^{1}H NMR (400 MHz, $CDCl_3$): δ = 7.58 (dd, *J* = 7.6, 1.7 Hz, 1H), 7.16 – 7.09 (m, 1H), 7.00 (td, *J* = 7.5, 1.2 Hz, 1H), 6.83 (dd, *J* = 8.0, 1.3 Hz, 1H), 4.16 (dd, *J* = 10.5, 1.3 Hz, 1H), 3.82 – 3.72 (m, 1H), 3.41 (dd, *J* = 8.5, 4.9 Hz, 1H), 1.85 (m, 1H), 1.35 (dd, *J* = 9.8, 5.5 Hz, 1H), 1.18 (m, 1H). The spectroscopic data are consistent with those previously reported. [23]

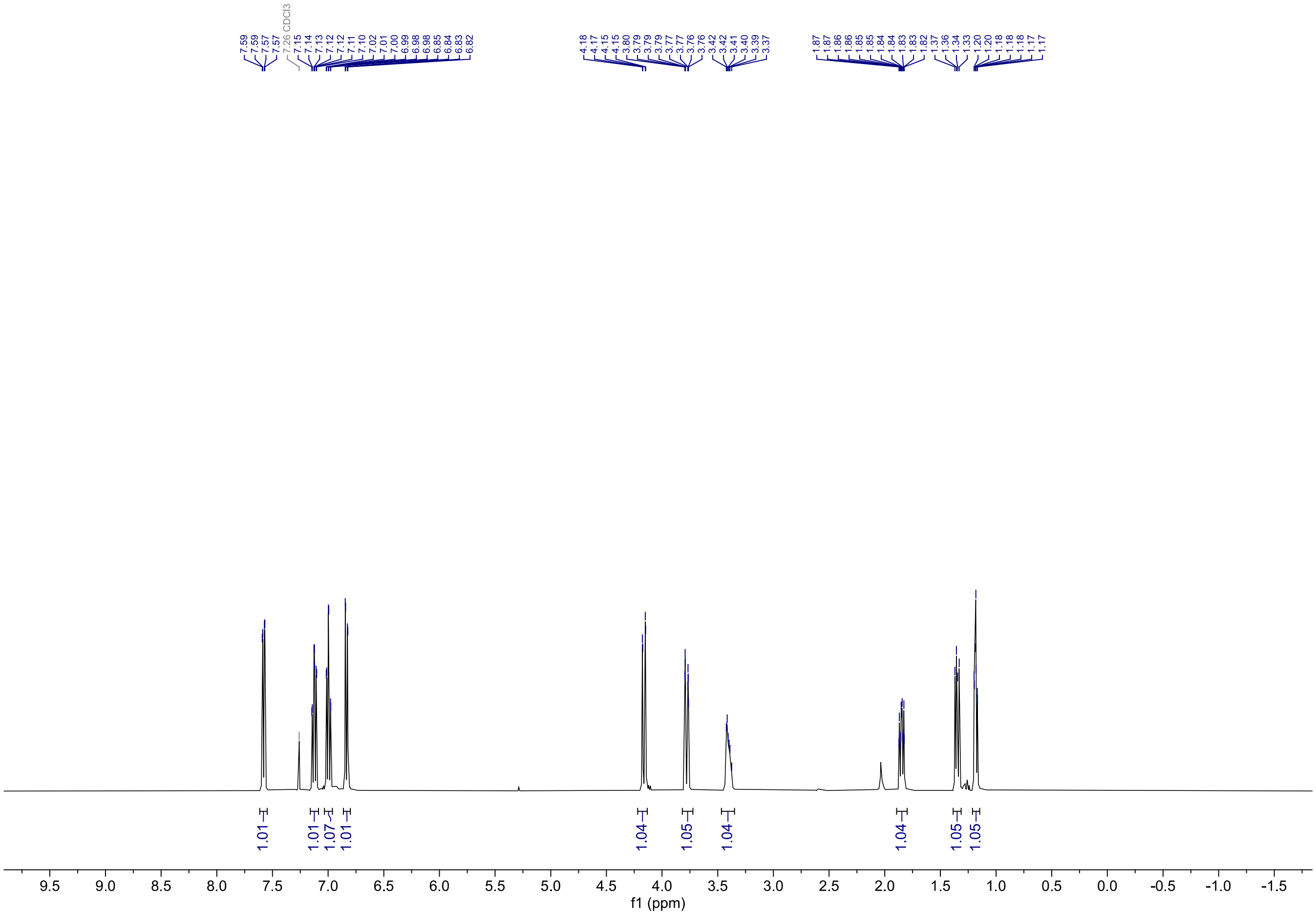

Figure S16. ^{1}H NMR of 1a,2-dihydrocyclopropa[*c*]chromen-7b(1*H*)-ol (**1p**) in $CDCl_3$ measured at 400 MHz.

1-(cyclohex-1-en-1-yl)cyclopropan-1-ol

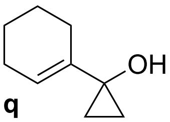

1q

1q was synthesized following the general procedure **2** and isolated via column chromatography (Al_2O_3/*n*-hexane, EtOAc/*n*-hexane 1/20, EtOAc/*n*-hexane 1/10) in 32% (1090 mg) yield as a yellowish oil.

^{1}H NMR (400 MHz, $CDCl_3$): δ = 5.76 (tt, *J* = 3.7, 1.7 Hz, 1H), 2.04 (m, 3H), 1.94 (m, 2H), 1.63 (m, 2H), 1.59 – 1.52 (m, 2H), 0.86 – 0.80 (m, 2H), 0.79 – 0.73 (m, 2H). The spectroscopic data are consistent with those previously reported. [24]

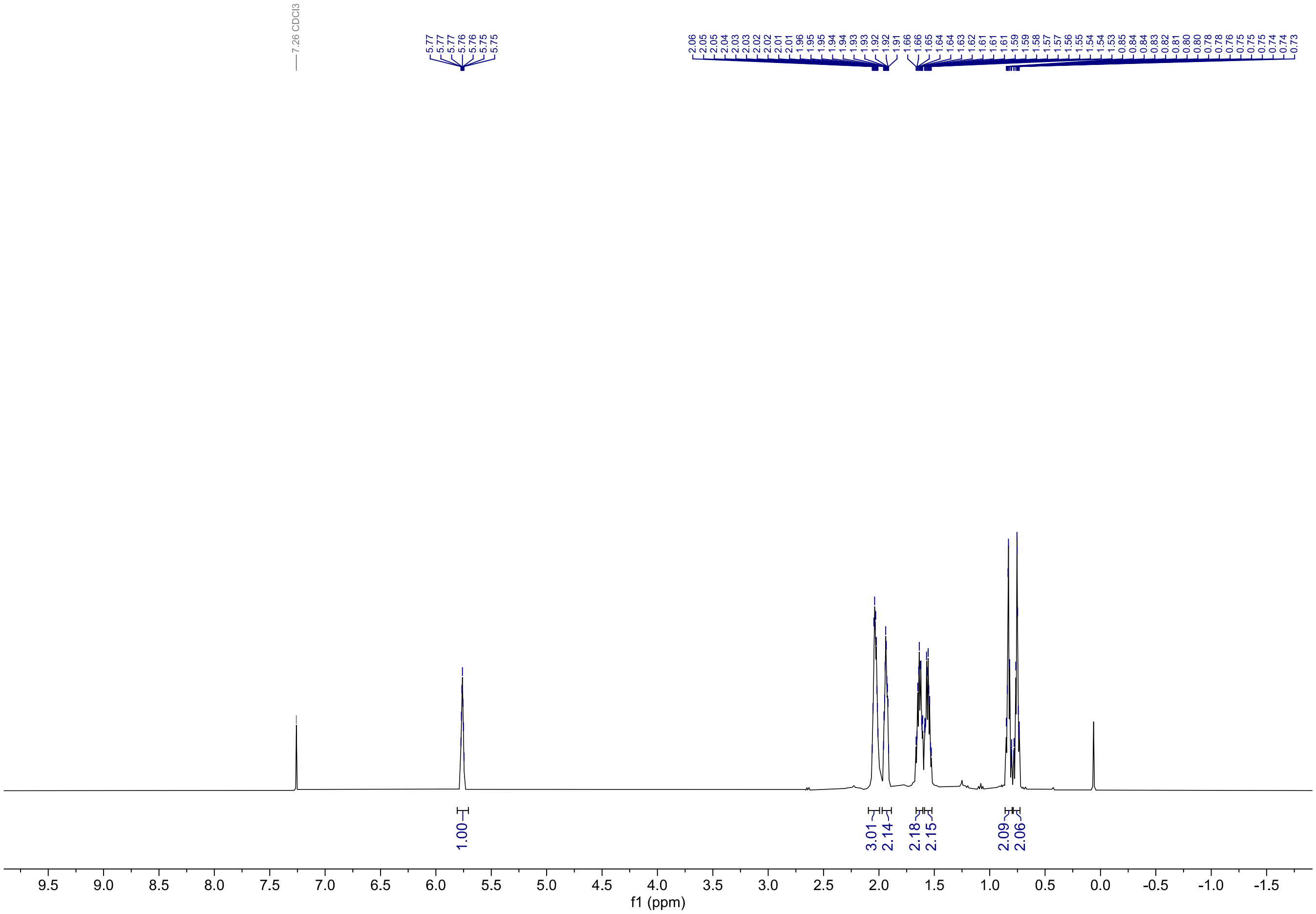

Figure S17. ^{1}H NMR of 1-(cyclohex-1-en-1-yl)cyclopropan-1-ol (**1q**) in $CDCl_3$ measured at 400 MHz.

2-phenyl-[1,1'-bi(cyclopropan)]-2-ol

1r 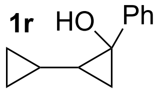

1r was synthesized following the general procedure **1** and isolated via column chromatography (SiO_2/*n*-hexane, EtOAc/*n*-hexane 1/20, EtOAc/*n*-hexane 1/10) in 61% (2112 mg) yield as a yellowish solid.

^{1}H NMR (400 MHz, $CDCl_3$): δ = 7.33 – 7.25 (m, 3H), 7.24 (d, *J* = 2.2 Hz, 1H), 7.22 – 7.15 (m, 1H), 2.38 (s, 1H), 1.20 – 1.12 (m, 1H), 1.04 – 0.94 (m, 2H), 0.94 – 0.85 (m, 1H), 0.66 – 0.52 (m, 2H), 0.35 (m, 1H), 0.29 – 0.20 (m, 1H). The spectroscopic data are consistent with those previously reported. [4]

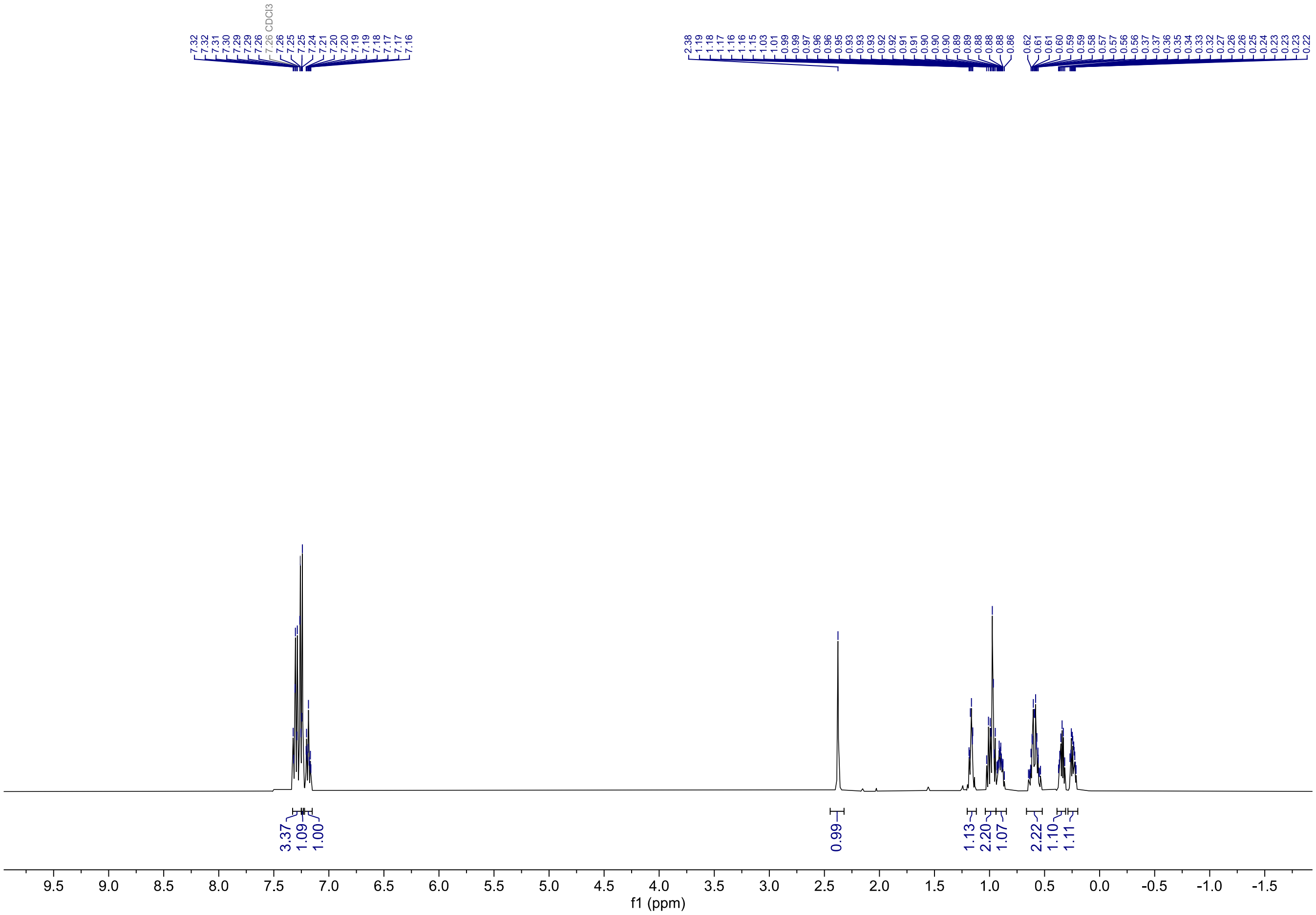

Figure S18. ^{1}H NMR of 2-phenyl-[1,1'-bi(cyclopropan)]-2-ol (**1r**) in $CDCl_3$ measured at 400 MHz.

4,4-difluorobicyclo[4.1.0]heptan-1-ol

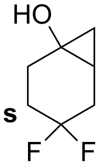

1s

1s was synthesized following the general procedure **1** and isolated via column chromatography (SiO_2/*n*-hexane, EtOAc/*n*-hexane 1/4) in 44% (1642 mg) yield as an off-white solid.

¹H NMR (400 MHz, $CDCl_3$): δ = 2.52 – 2.37 (m, 1H), 2.36 – 2.15 (m, 3H), 2.05 – 1.91 (m, 1H), 1.76 (m, 1H), 1.61 – 1.42 (m, 1H), 1.26 – 1.16 (m, 1H), 1.06 (m, 1H), 0.49 (t, *J* = 6.0 Hz, 1H). The spectroscopic data are consistent with those previously reported. [25]

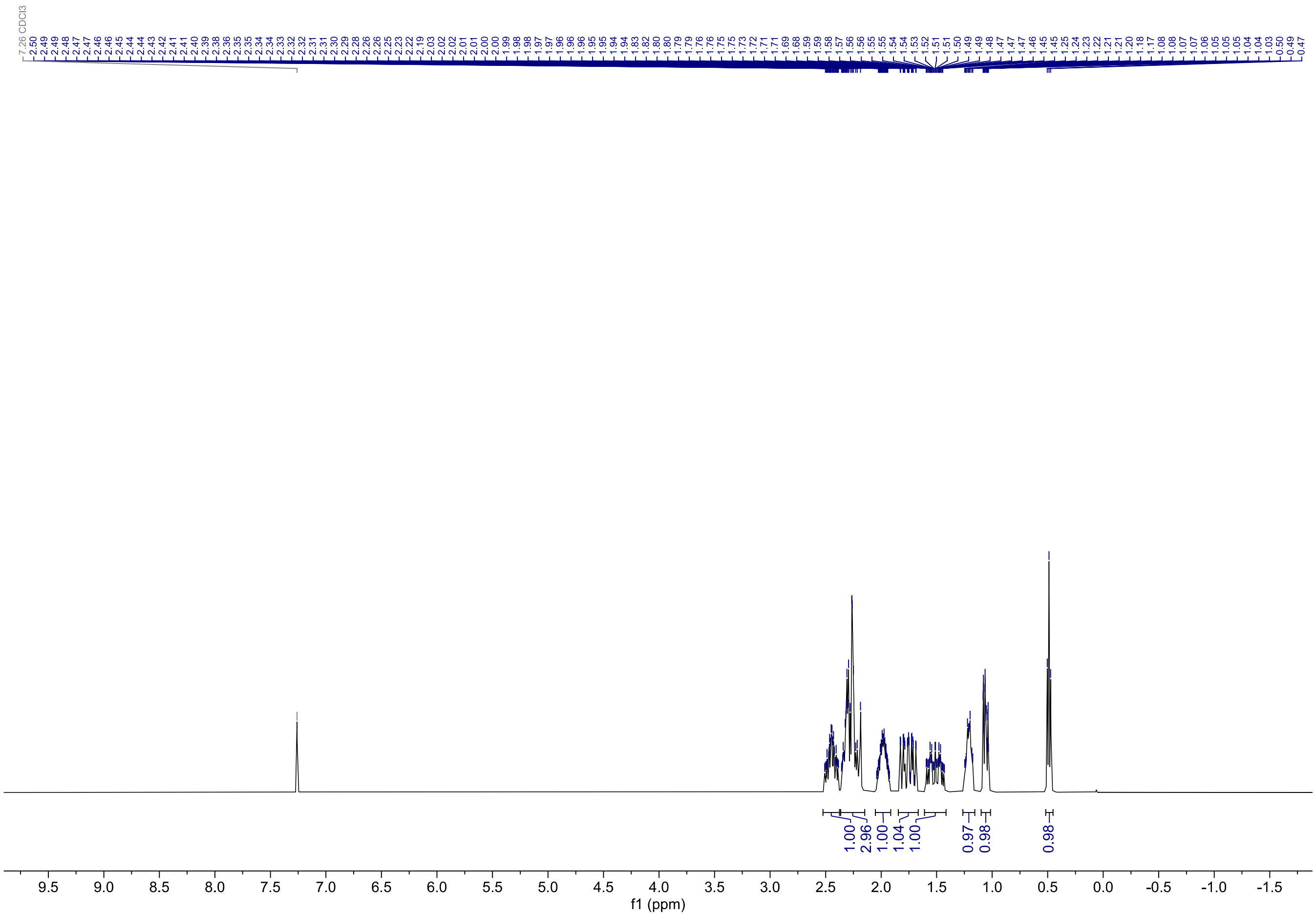

Figure S19. ¹H NMR of 4,4-difluorobicyclo[4.1.0]heptan-1-ol (**1s**) in $CDCl_3$ measured at 400 MHz.

(*E*)-1-(pent-2-en-1-yl)cyclopropan-1-ol

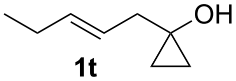

1t

1t was synthesized following the general procedure **2** and isolated via column chromatography (SiO_2/*n*-hexane, *n*-hexane, EtOAc/*n*-hexane 1/20) in 22% (709 mg) yield as a yellowish oil.

^{1}H NMR (400 MHz, $CDCl_3$): δ = 5.65 (m, 1H), 5.49 (m, 1H), 2.27 (dd, *J* = 7.0, 1.3 Hz, 2H), 2.06 (m, 2H), 1.98 (s, 1H), 1.00 (t, *J* = 7.5 Hz, 3H), 0.77 – 0.73 (m, 2H), 0.50 – 0.46 (m, 2H). The spectroscopic data are consistent with those previously reported. [26]

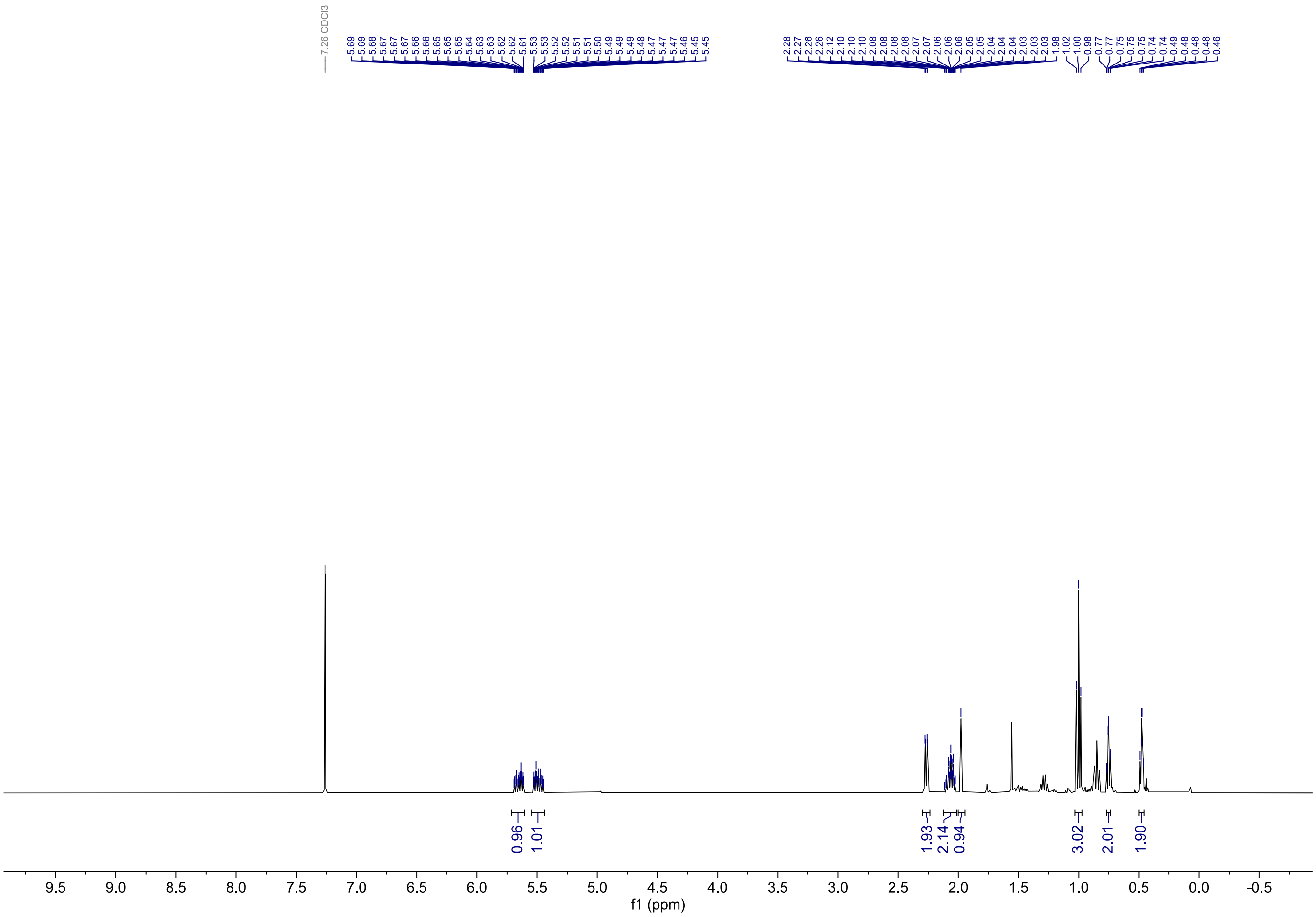

Figure S20. ^{1}H NMR of (*E*)-1-(pent-2-en-1-yl)cyclopropan-1-ol (**1t**) in $CDCl_3$ measured at 400 MHz.

1-(naphthalen-1-yl)cyclopropan-1-ol

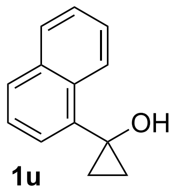

1u

1u was synthesized following the general procedure **2** and isolated via column chromatography (SiO_2/*n*-hexane, EtOAc/*n*-hexane 1/10, EtOAc/*n*-hexane 1/6) in 38% (1771 mg) yield as a colourless solid.

^{1}H NMR (400 MHz, $CDCl_3$): δ = 8.59 (dd, *J* = 8.4, 1.3 Hz, 1H), 7.89 (dd, *J* = 8.1, 1.4 Hz, 1H), 7.82 (d, *J* = 8.2 Hz, 1H), 7.60 (m, 1H), 7.56 – 7.49 (m, 2H), 7.42 (dd, *J* = 8.2, 7.0 Hz, 1H), 2.38 (d, *J* = 8.4 Hz, 1H), 1.37 – 1.32 (m, 2H), 1.10 – 1.06 (m, 2H). The spectroscopic data are consistent with those previously reported. [27]

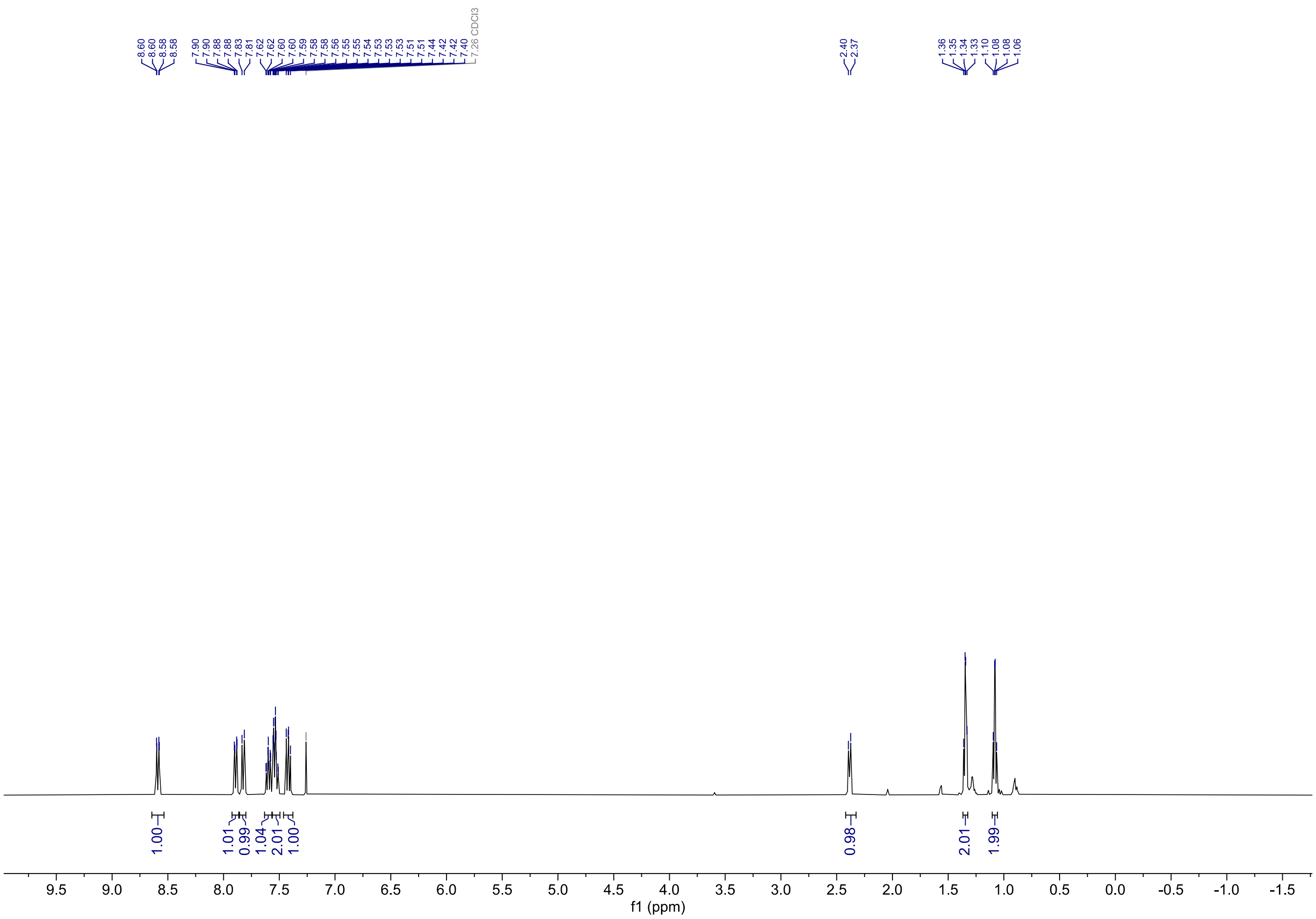

Figure S21. ^{1}H NMR of 1-(naphthalen-1-yl)cyclopropan-1-ol (**1u**) in $CDCl_3$ measured at 400 MHz.

1-(2-chlorophenyl)cyclopropan-1-ol

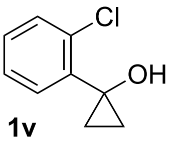

1v

1v was synthesized following the general procedure **1** and isolated via column chromatography (SiO_2/*n*-hexane, EtOAc/*n*-hexane 1/10, EtOAc/*n*-hexane 1/5) in 83% (3509 mg) yield as a colourless solid.

^{1}H NMR (400 MHz, $CDCl_3$): δ = 7.46 – 7.34 (m, 2H), 7.29 – 7.20 (m, 2H), 2.95 (s, 1H), 1.25 – 1.19 (m, 2H), 1.02 – 0.94 (m, 2H).

^{13}C NMR (101 MHz, $CDCl_3$): δ = 139.5, 135.7, 130.3, 129.9, 129.4, 127.0, 56.9, 14.5.

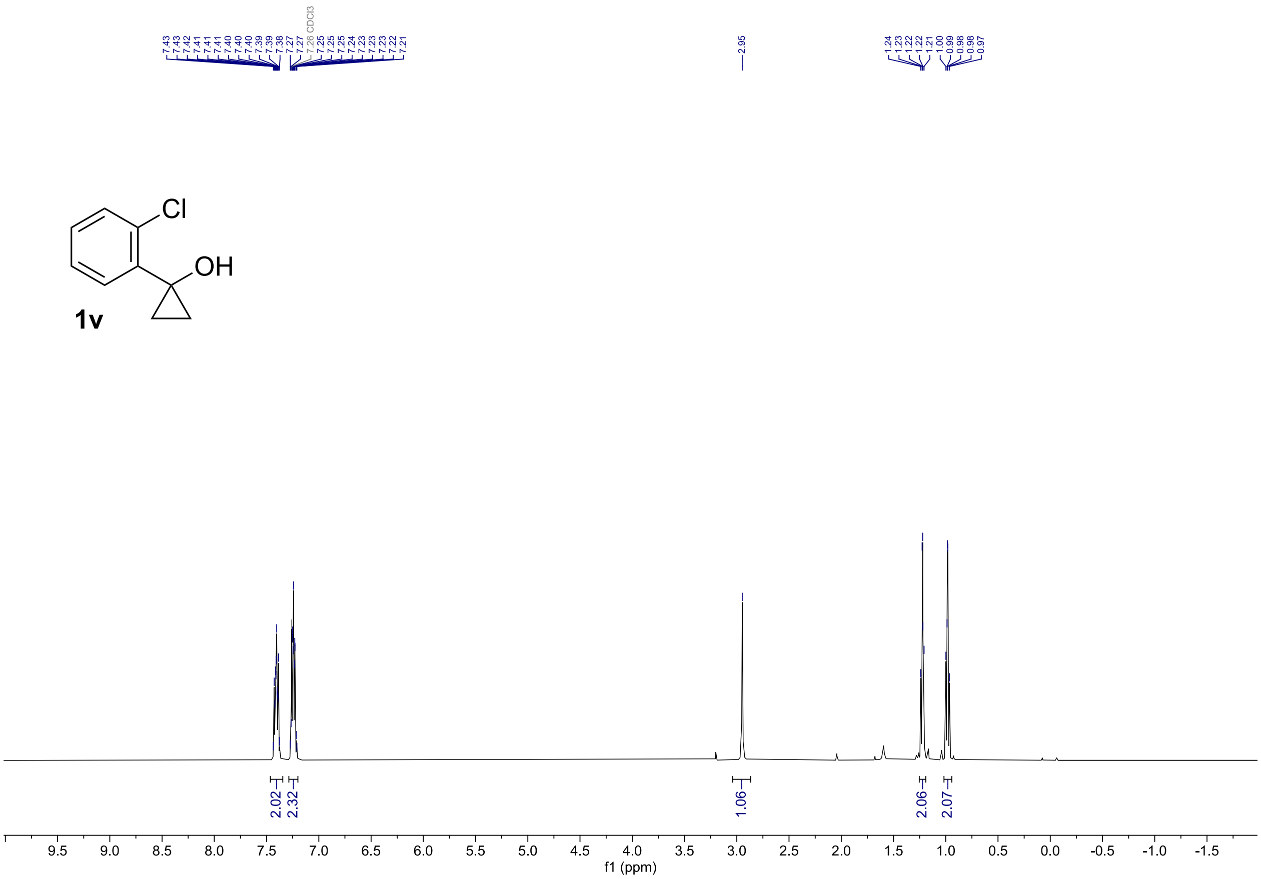

Figure S22. ^{1}H NMR of 1-(2-chlorophenyl)cyclopropan-1-ol (**1v**) in $CDCl_3$ measured at 400 MHz.

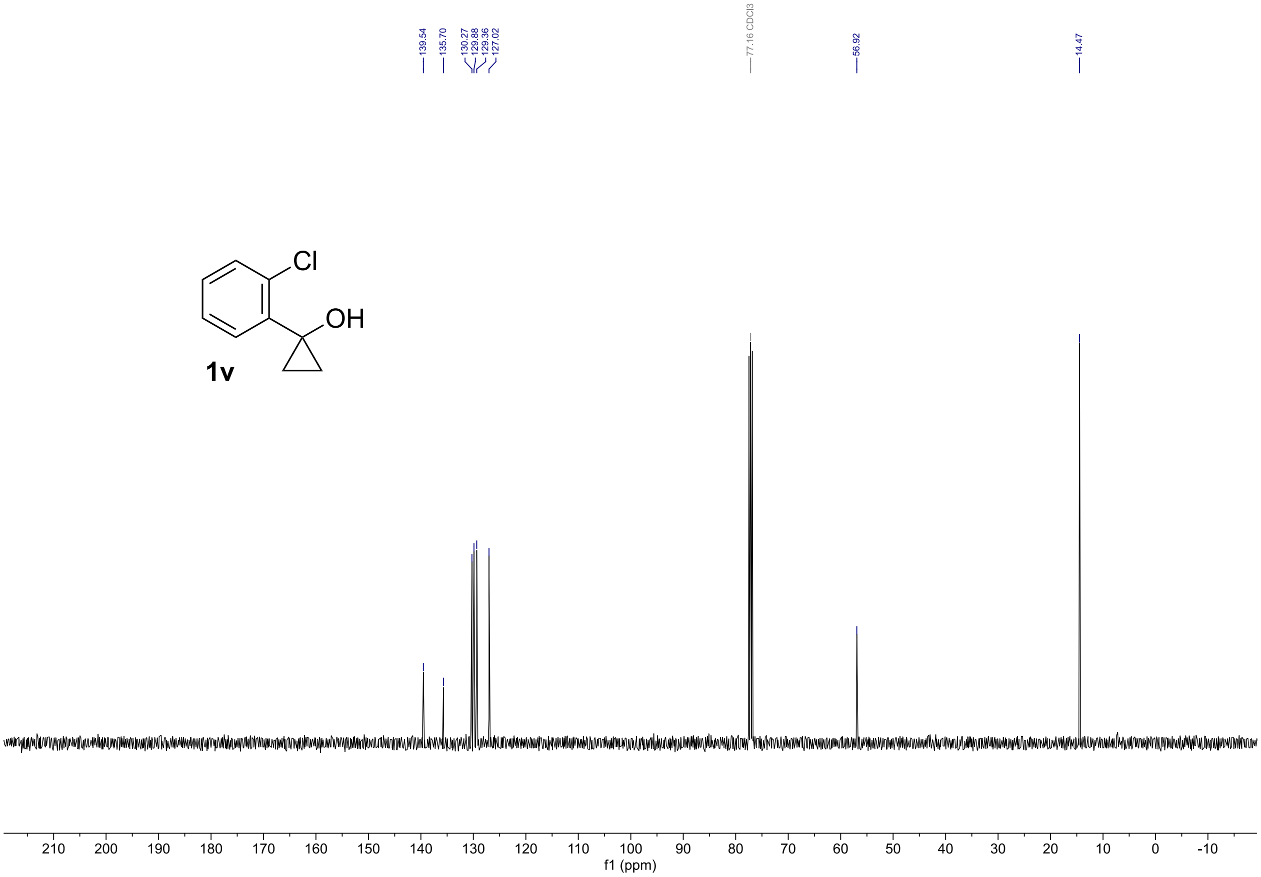

Figure S23. ^{1}3C NMR of 1-(2-chlorophenyl)cyclopropan-1-ol (**1v**) in $CDCl_3$ measured at 101 MHz.

1-(4-iodophenyl)cyclopropan-1-ol

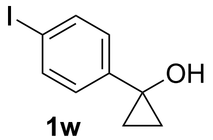

1w

1w was synthesized following the general procedure **1** and isolated via column chromatography (SiO_2/*n*-hexane, EtOAc/*n*-hexane 1/10, EtOAc/*n*-hexane 1/5) in 82% (5301 mg) yield as a pale yellow solid.

^{1}H NMR (400 MHz, $CDCl_3$): δ = 7.64 (d, *J* = 8.1 Hz, 2H), 7.03 (d, *J* = 8.0 Hz, 2H), 2.40 (s, 1H), 1.27 (d, *J* = 6.1 Hz, 2H), 1.03 (d, *J* = 6.5 Hz, 2H). The spectroscopic data are consistent with those previously reported. [21]

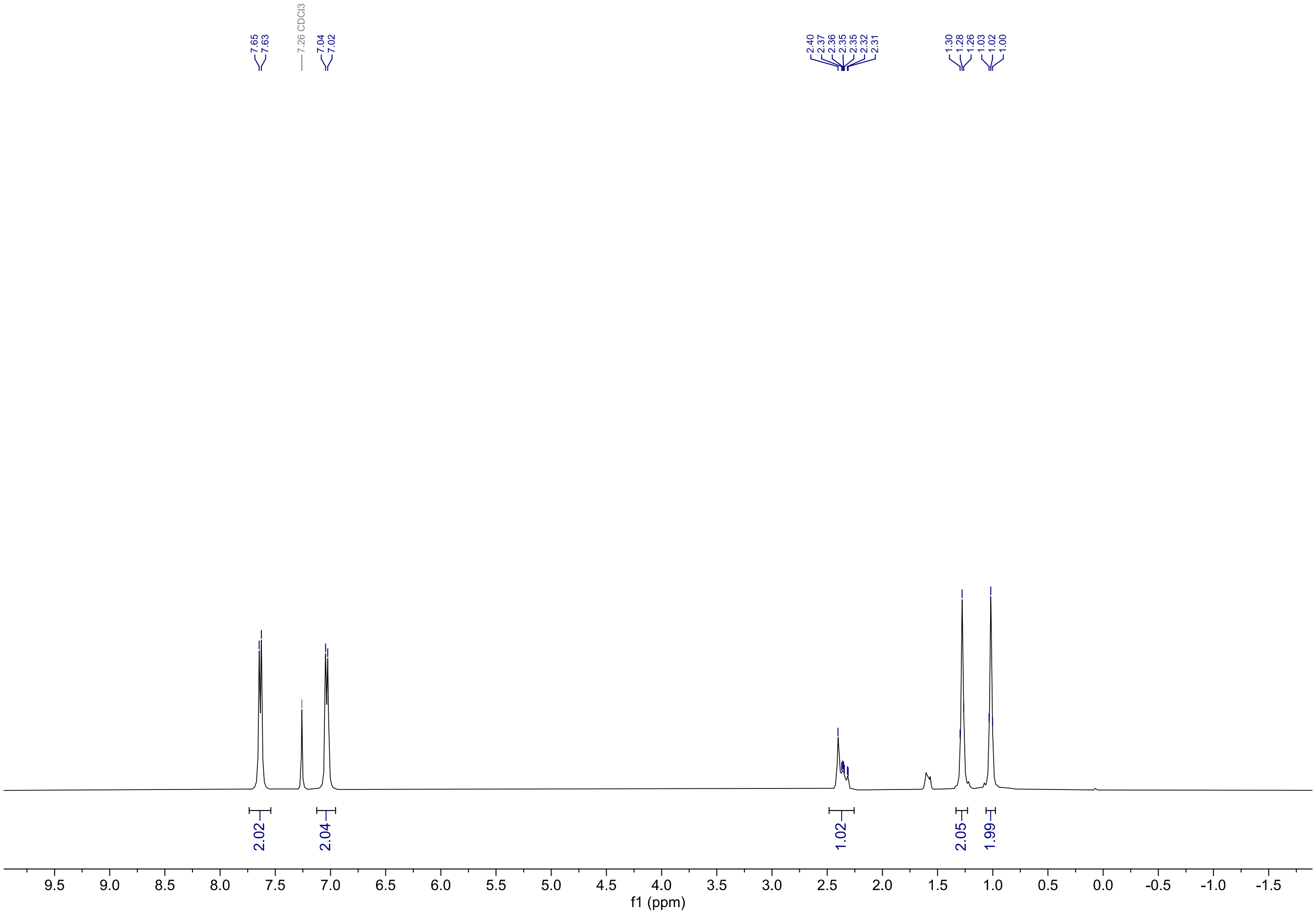

Figure S24. ^{1}H NMR of 1-(4-iodophenyl)cyclopropan-1-ol (**1w**) in $CDCl_3$ measured at 400 MHz.

7. Synthesized 1,4-diketones

1-(4-chlorophenyl)-4-phenylbutane-1,4-dione

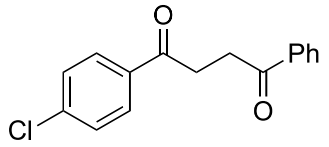

3aa

3aa was synthesized following the general procedure **4**, employing **1a** (169 mg, 1.00 mmol, 1.00 eq), **2a** (321 mg, 1.50 mmol, 1.50 eq), CuCl (149 mg, 1.50 mmol, 1.50 eq), K_2CO_3 (138 mg, 1.00 mmol, 1.00 eq) at 80°C, and isolated via flash chromatography (23 g SiO_2, gradient from 10:90 to 20:80 EtOAc/*n*-hexane over 10 CV, gradient from 20:80 to 50:50 EtOAc/*n*-hexane over 5 CV) as a pale ochre solid (249 mg, 91%).

$C_{16}H_{13}ClO_2$ (272,73 g/mol)

mp: 110-112°C

Rf: 0.31 (EtOAc/n-hexane = 1:10) [UV]

^{1}H NMR (400 MHz, $CDCl_3$): δ = 8.08 – 7.93 (m, 4H), 7.62 – 7.54 (m, 1H), 7.52 – 7.43 (m, 4H), 3.55 – 3.35 (m, 4H).

^{13}C NMR (101 MHz, $CDCl_3$): δ = 198.6, 197.6, 139.7, 136.8, 135.3, 133.4, 129.7, 129.1, 128.8, 128.3, 32.7, 32.7.

HRMS (ESI) m/z: $[M+H]^+$ Calcd for $C_{16}H_{13}ClO_2H$ 273.0677; Found 273.0675

IR (ATR, ṽ): 1672 (s, CO).

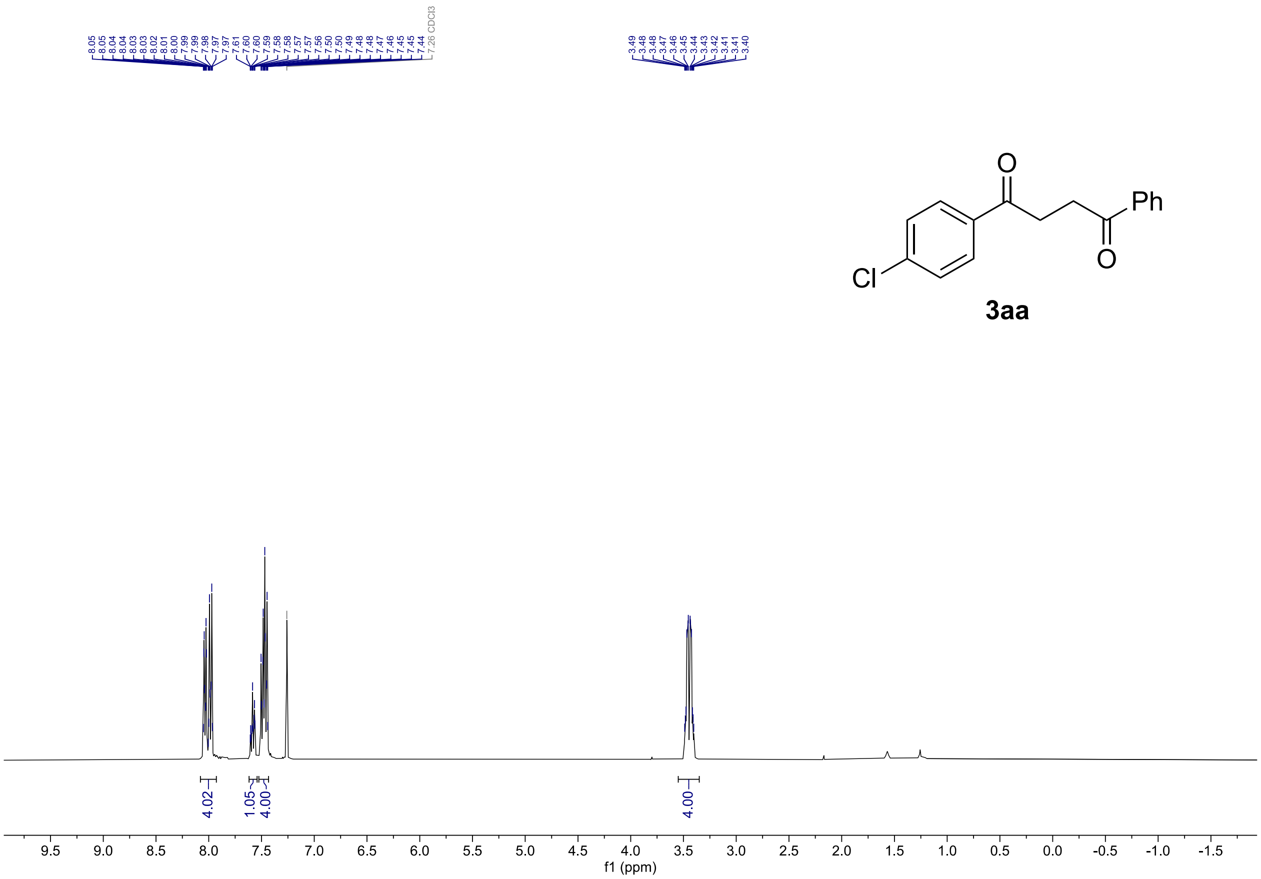

Figure S25. ^{1}H NMR of 1-(4-chlorophenyl)-4-phenylbutane-1,4-dione (**3aa)** in $CDCl_3$ measured at 400 MHz.

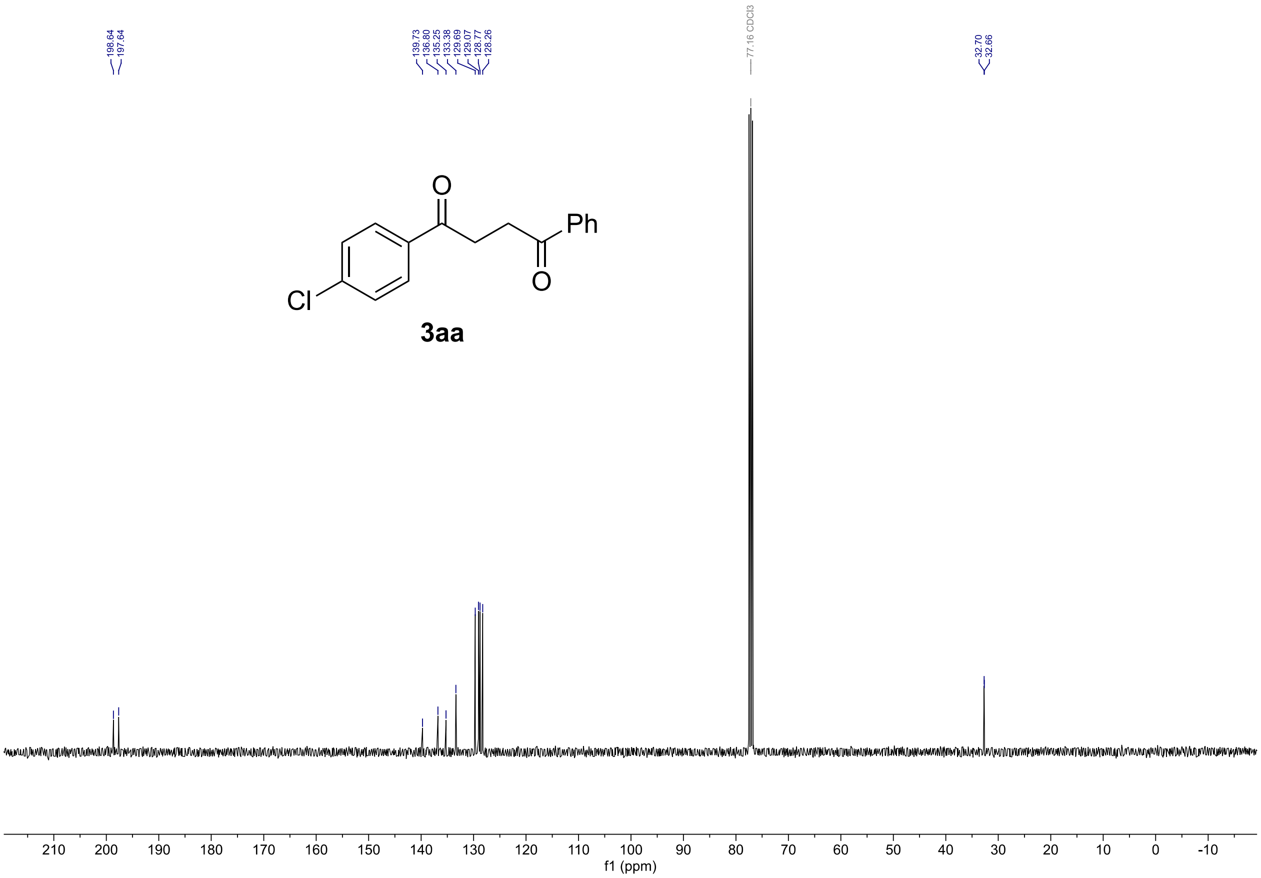

Figure S26. ^{13}C NMR of 1-(4-chlorophenyl)-4-phenylbutane-1,4-dione (**3aa)** in $CDCl_3$ measured at 101 MHz.

1-(4-fluorophenyl)-4-phenylbutane-1,4-dione

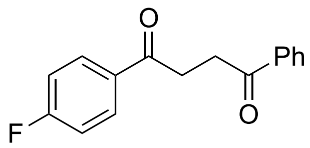

3ba

3ba was synthesized following the general procedure **4**, employing **1b** (152 mg, 1.00 mmol, 1.00 eq), **2a** (321 mg, 1.50 mmol, 1.50 eq), CuCl (149 mg, 1.50 mmol, 1.50 eq), K_2CO_3 (138 mg, 1.00 mmol, 1.00 eq) at 80°C, and isolated via flash chromatography (23 g SiO_2, gradient from 10:90 to 20:80 EtOAc/*n*-hexane over 10 CV, gradient from 20:80 to 50:50 EtOAc/*n*-hexane over 5 CV) as a pale ochre solid (237 mg, 92%).

$C_{16}H_{13}FO_2$ (256,28 g/mol)

mp: 106-107°C

Rf: 0.24 (EtOAc/n-hexane = 1:10) [UV]

^{1}H NMR (400 MHz, $CDCl_3$): δ = 8.16 – 7.95 (m, 4H), 7.64 – 7.54 (m, 1H), 7.49 (m, 2H), 7.21 – 7.07 (m, 2H), 3.51 – 3.39 (m, 4H).

^{13}C NMR (101 MHz, $CDCl_3$): δ = 198.4, 197.0, 165.7 (d, *J* = 254.6 Hz), 136.6, 133.2 (d, *J* = 2.9 Hz), 133.1, 130.7 (d, *J* = 9.2 Hz), 128.5, 128.0, 115.6 (d, *J* = 22.0 Hz), 32.5, 32.4.

^{19}F NMR (376 MHz, $CDCl_3$): δ = -106.29 (td, *J* = 8.8, 4.4 Hz, 1F).

HRMS (ESI) m/z: $[M+Na]^+$ Calcd for $C_{16}H_{13}FO_2Na$ 279.0792; Found 279.0790.

IR (ATR, ṽ): 1675 (s, CO).

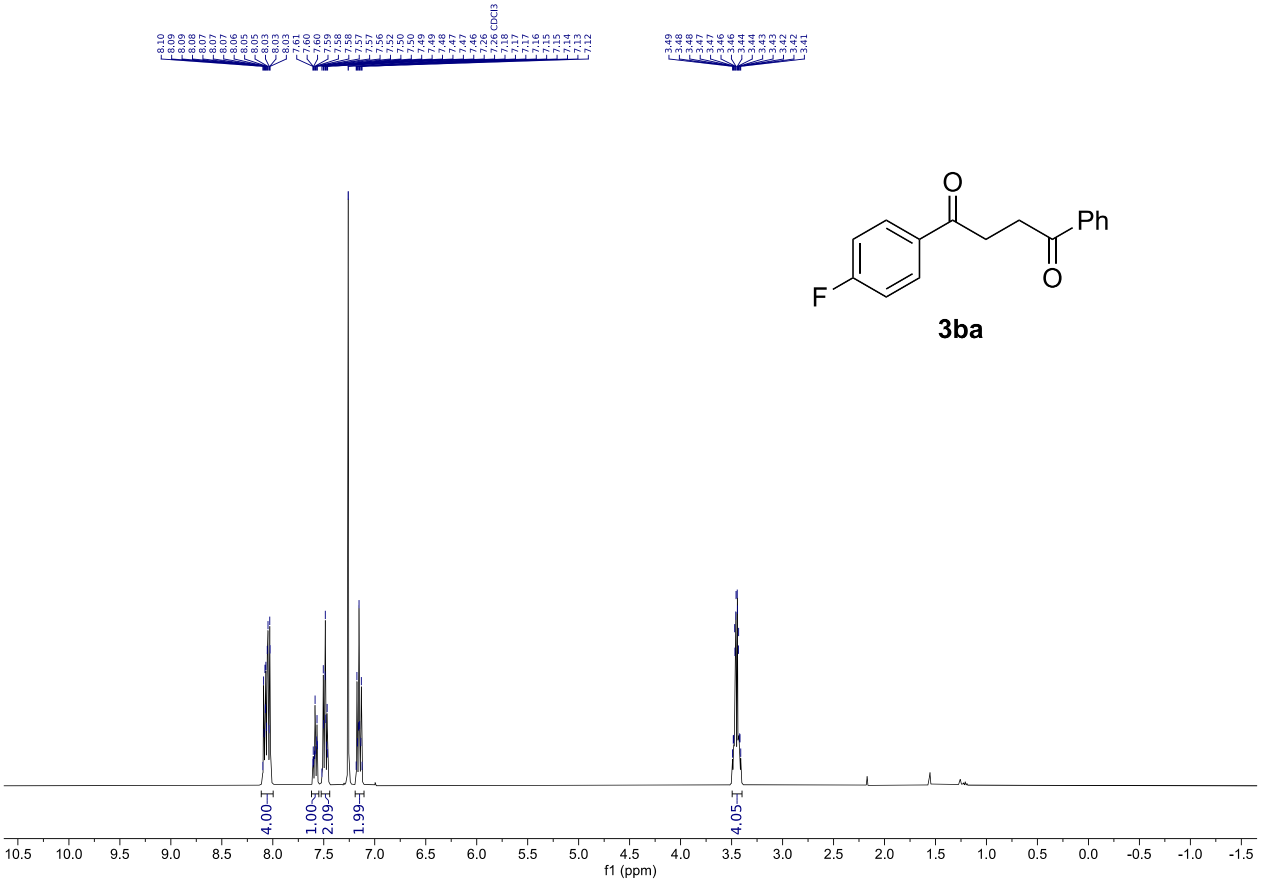

Figure S27. ^{1}H NMR of 1-(4-fluorophenyl)-4-phenylbutane-1,4-dione (**3ba**) in $CDCl_3$ measured at 400 MHz.

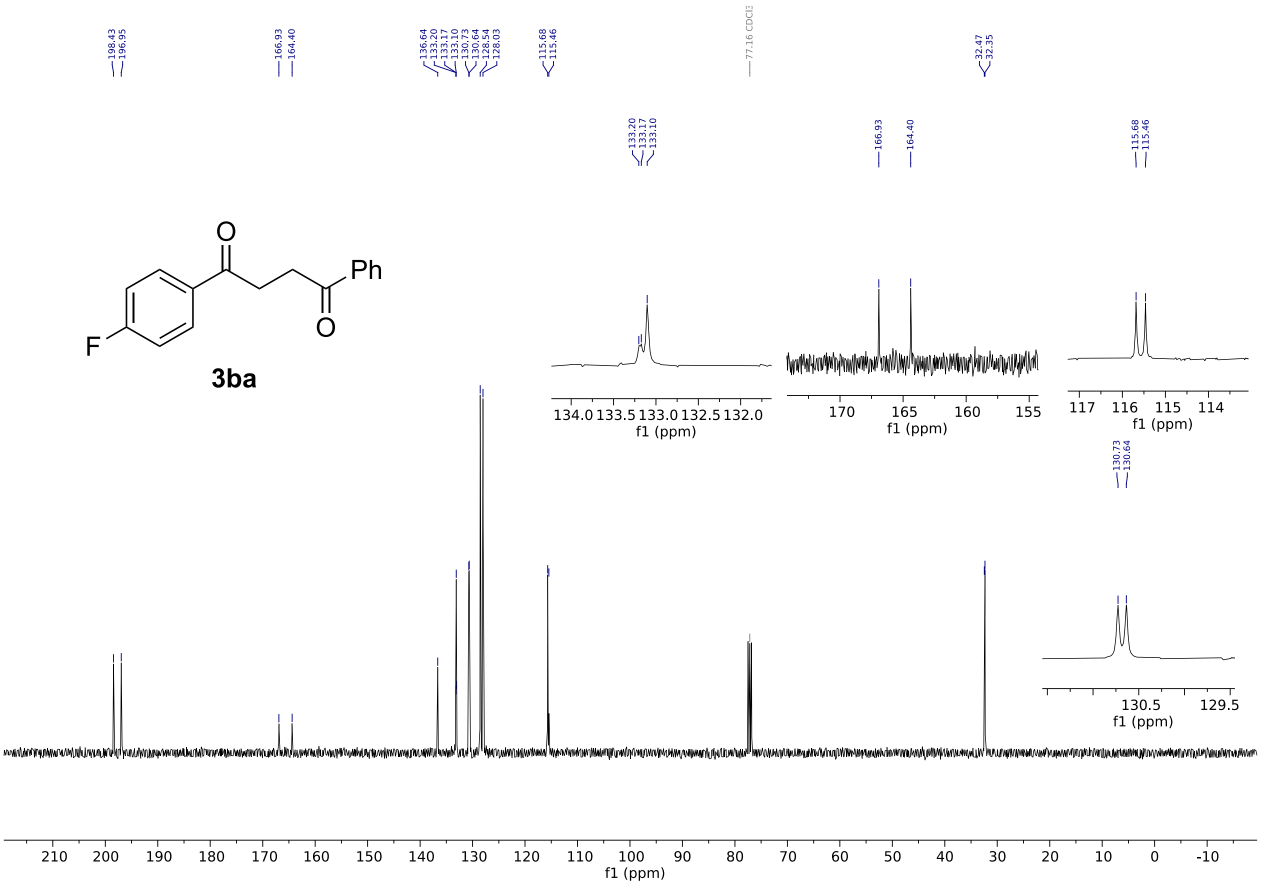

Figure S28. ^{13}C NMR of 1-(4-fluorophenyl)-4-phenylbutane-1,4-dione (**3ba**) in $CDCl_3$ measured at 101 MHz.

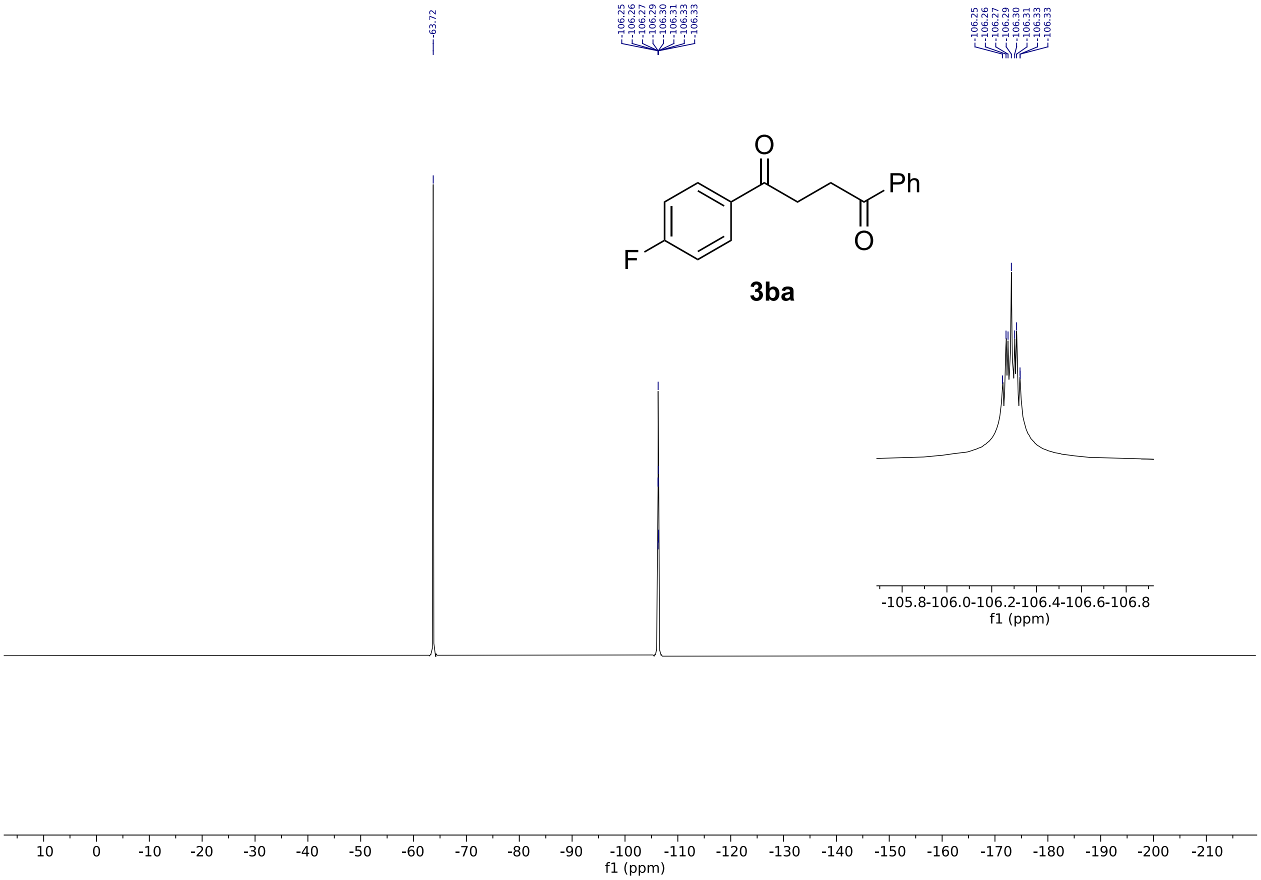

Figure S29. ^{19}F NMR of 1-(4-fluorophenyl)-4-phenylbutane-1,4-dione (**3ba**) in $CDCl_3$ measured at 376 MHz.

1-(4-bromophenyl)-4-phenylbutane-1,4-dione

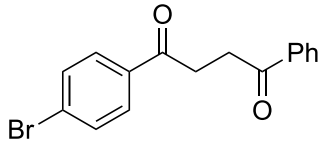

3ca

3ca was synthesized following the general procedure **4**, employing **1c** (213 mg, 1.00 mmol, 1.00 eq), **2a** (321 mg, 1.50 mmol, 1.50 eq), CuCl (149 mg, 1.50 mmol, 1.50 eq), K_2CO_3 (138 mg, 1.00 mmol, 1.00 eq) at 80°C, and isolated via flash chromatography (23 g SiO_2, gradient from 10:90 to 20:80 EtOAc/*n*-hexane over 10 CV, gradient from 20:80 to 50:50 EtOAc/*n*-hexane over 5 CV) as a pale yellow solid (271 mg, 85%).

$C_{16}H_{13}BrO_2$ (317,18 g/mol)

mp: 115°C

Rf: 0.27 (EtOAc/n-hexane = 1:10) [UV]

^{1}H NMR (400 MHz, $CDCl_3$): δ = 8.03 (dt, *J* = 7.0, 1.4 Hz, 2H), 7.93 – 7.87 (m, 2H), 7.65 – 7.59 (m, 2H), 7.59 – 7.56 (m, 1H), 7.52 – 7.45 (m, 2H), 3.51 – 3.38 (m, 4H).

^{13}C NMR (101 MHz, $CDCl_3$): δ = 198.6, 197.8, 136.8, 135.6, 133.4, 132.0, 129.8, 128.7, 128.4, 128.2, 32.7, 32.6.

HRMS (ESI) m/z: $[M+Na]^+$ Calcd for $C_{16}H_{13}BrO_2Na$ 338.9991; Found 338.9992.

IR (ATR, ṽ): 1668 (s, CO).

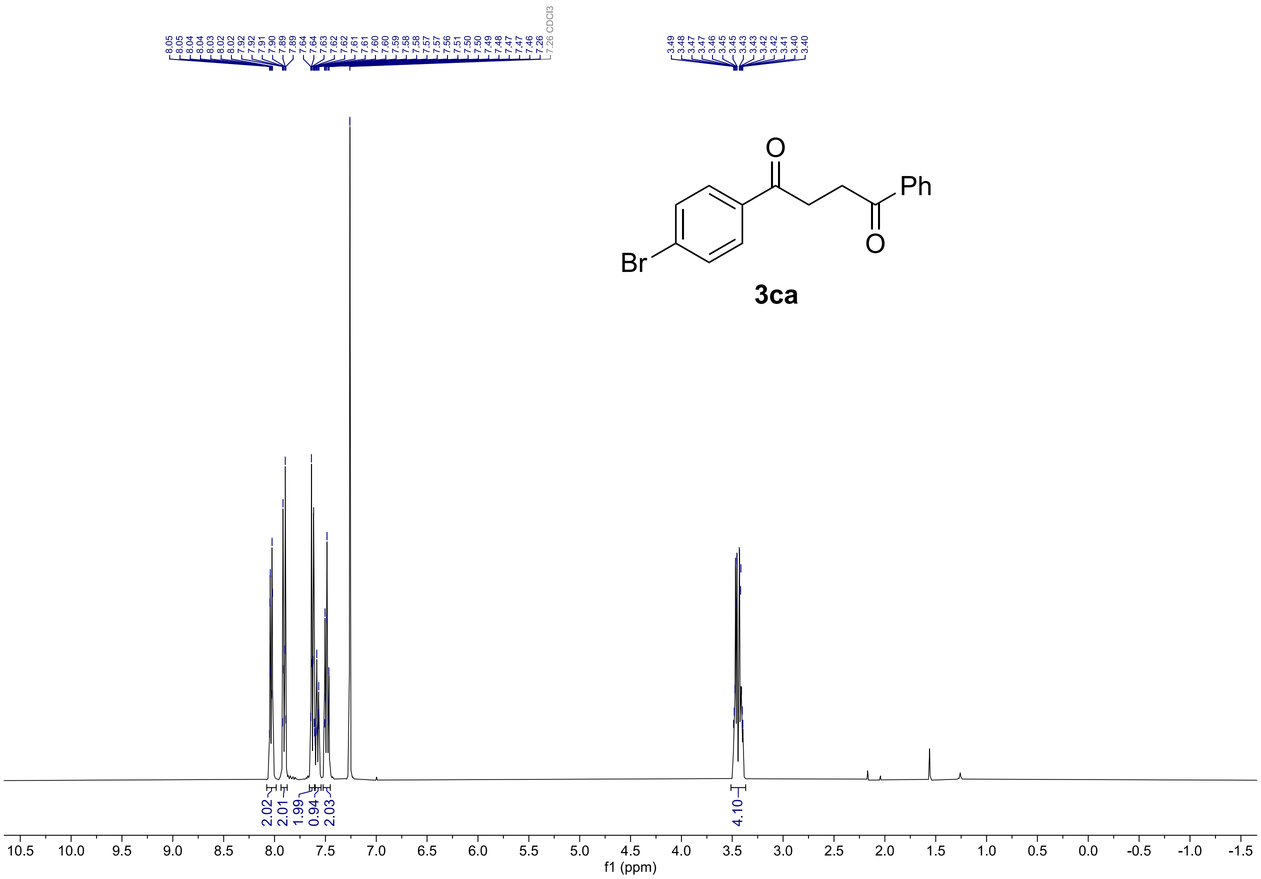

Figure S30. ^{1}H NMR of 1-(4-bromophenyl)-4-phenylbutane-1,4-dione (**3ca**) in $CDCl_3$ measured at 400 MHz.

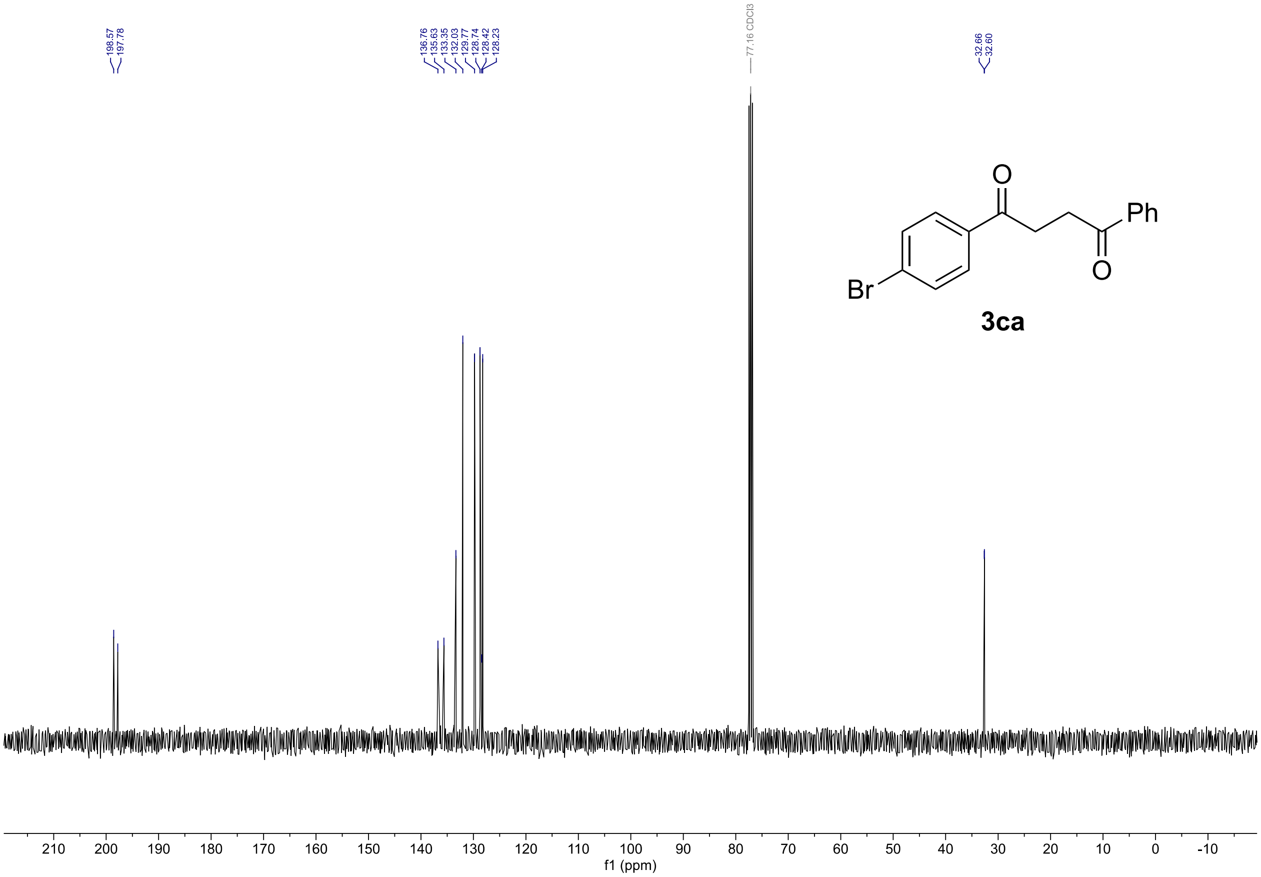

Figure S31. ^{1}3C NMR of 1-(4-bromophenyl)-4-phenylbutane-1,4-dione (**3ca**) in $CDCl_3$ measured at 101 MHz.

1-(4-methoxyphenyl)-4-phenylbutane-1,4-dione

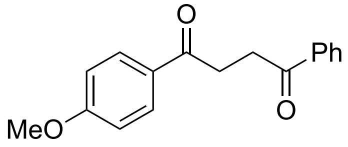

3da

3da was synthesized following the general procedure **4**, employing **1d** (164 mg, 1.00 mmol, 1.00 eq), **2a** (321 mg, 1.50 mmol, 1.50 eq), CuCl (149 mg, 1.50 mmol, 1.50 eq), K_2CO_3 (138 mg, 1.00 mmol, 1.00 eq) at 80°C, and isolated via flash chromatography (23 g SiO_2, gradient from 10:90 to 20:80 EtOAc/*n*-hexane over 10 CV, gradient from 20:80 to 50:50 EtOAc/*n*-hexane over 5 CV) as a pale yellow solid (245 mg, 91%).

$C_{17}H_{16}O_3$ (268,31 g/mol)

mp: 103-104°C

Rf: 0.15 (EtOAc/n-hexane = 1:10) [UV]

^{1}H NMR (400 MHz, $CDCl_3$): δ = 8.03 (t, *J* = 7.6 Hz, 4H), 7.57 (t, *J* = 7.2 Hz, 1H), 7.47 (t, *J* = 7.5 Hz, 2H), 6.95 (d, *J* = 8.6 Hz, 2H), 3.87 (s, 3H), 3.48 – 3.37 (m, 4H).

^{13}C NMR (101 MHz, $CDCl_3$): δ = 199.0, 197.3, 163.7, 137.0, 133.2, 130.5, 130.0, 128.7, 128.3, 113.9, 55.6, 32.8, 32.4.

HRMS (ESI) m/z: $[M+Na]^+$ Calcd for $C_{17}H_{16}O_3Na$ 291.0992; Found 291.0991.

IR (ATR, ṽ): 1675 (s, CO).

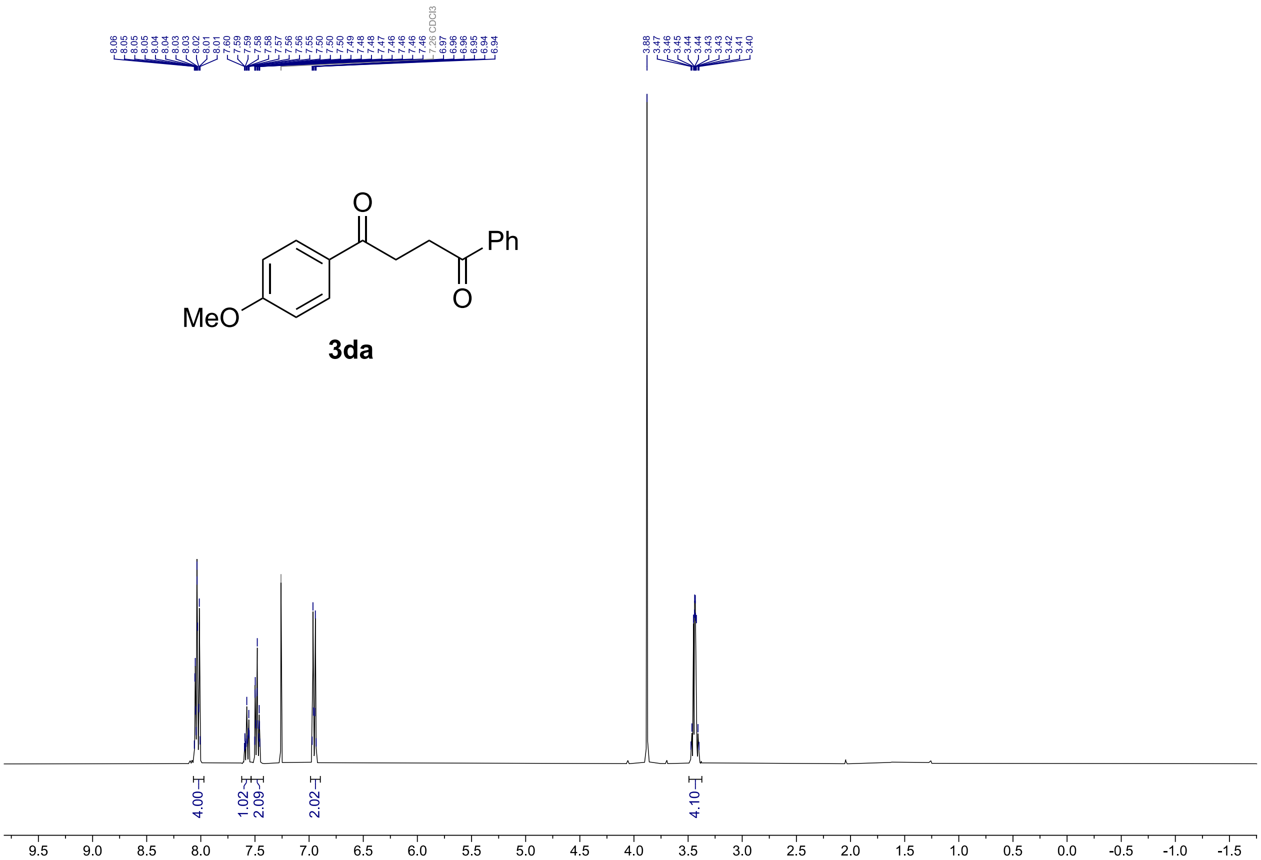

Figure S32. ^{1}H NMR of 1-(4-methoxyphenyl)-4-phenylbutane-1,4-dione (**3da**) in $CDCl_3$ measured at 400 MHz.

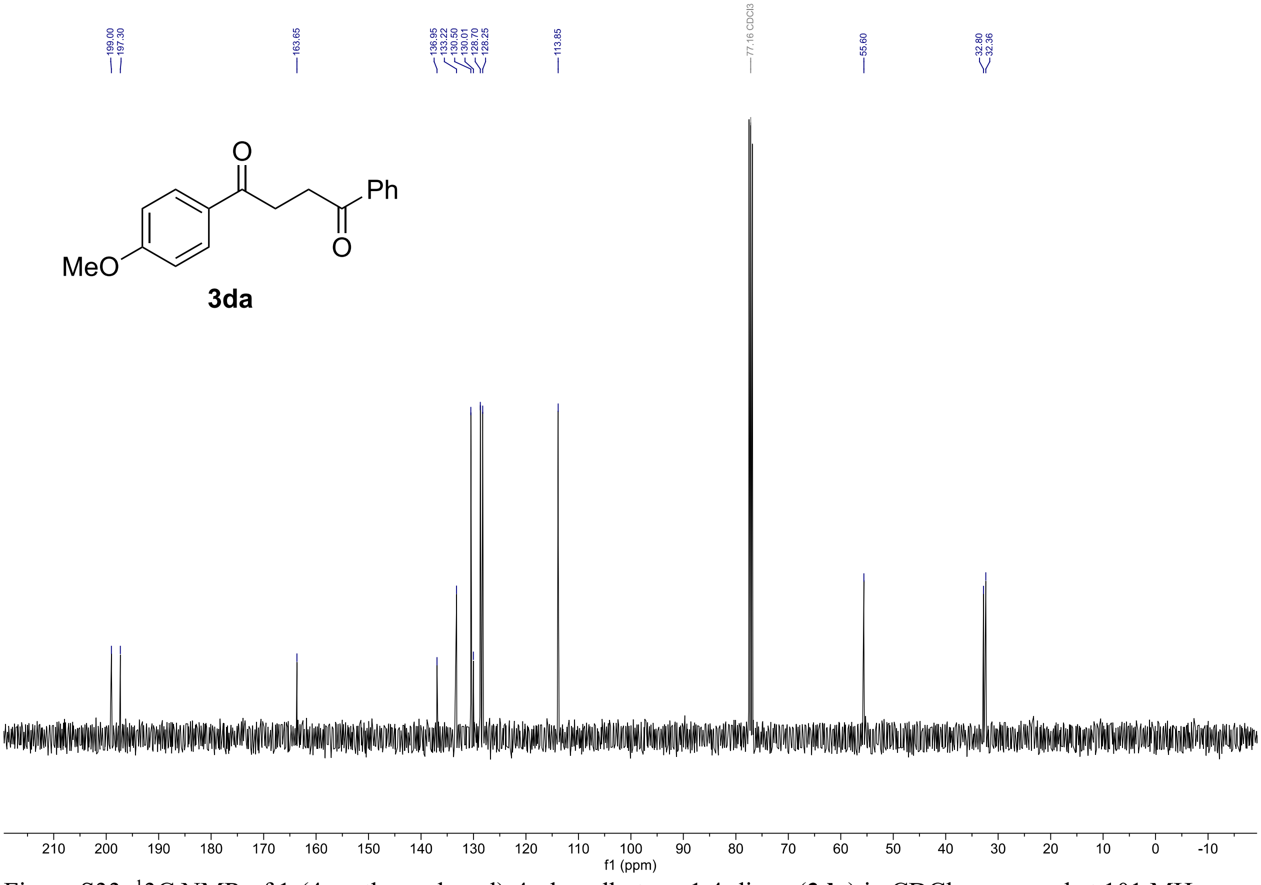

Figure S33. ^{13}C NMR of 1-(4-methoxyphenyl)-4-phenylbutane-1,4-dione (**3da**) in $CDCl_3$ measured at 101 MHz.

1-(4-(*tert*-butyl)phenyl)-4-phenylbutane-1,4-dione

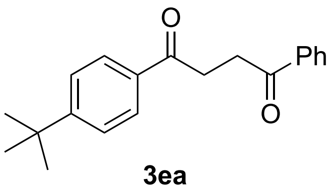

3ea

3ea was synthesized following the general procedure **4**, employing **1e** (190 mg, 1.00 mmol, 1.00 eq), **2a** (321 mg, 1.50 mmol, 1.50 eq), CuCl (149 mg, 1.50 mmol, 1.50 eq), K_2CO_3 (138 mg, 1.00 mmol, 1.00 eq) at 80°C, and isolated via flash chromatography (23 g SiO_2, gradient from 10:90 to 20:80 EtOAc/*n*-hexane over 10 CV, gradient from 20:80 to 50:50 EtOAc/*n*-hexane over 5 CV) as a colourless solid (265 mg, 90%).

$C_{20}H_{22}O_2$ (294,39 g/mol)

mp: 80 °C

Rf: 0.39 (EtOAc/n-hexane = 1:10) [UV]

^{1}H NMR (400 MHz, $CDCl_3$): δ = 8.04 (dt, *J* = 7.0, 1.4 Hz, 2H), 8.01 – 7.95 (m, 2H), 7.61 – 7.54 (m, 1H), 7.53 – 7.44 (m, 4H), 3.46 (m, 4H), 1.35 (s, 9H).

^{13}C NMR (101 MHz, $CDCl_3$): δ = 198.9, 198.4, 157.0, 136.9, 134.3, 133.2, 128.7, 128.2, 128.2, 125.7, 35.2, 32.8, 32.6, 31.2.

HRMS (ESI) m/z: $[M+Na]^+$ Calcd for $C_{20}H_{22}O_2Na$ 317.1512; Found 317.1510.

IR (ATR, ṽ): 1671 (s, CO).

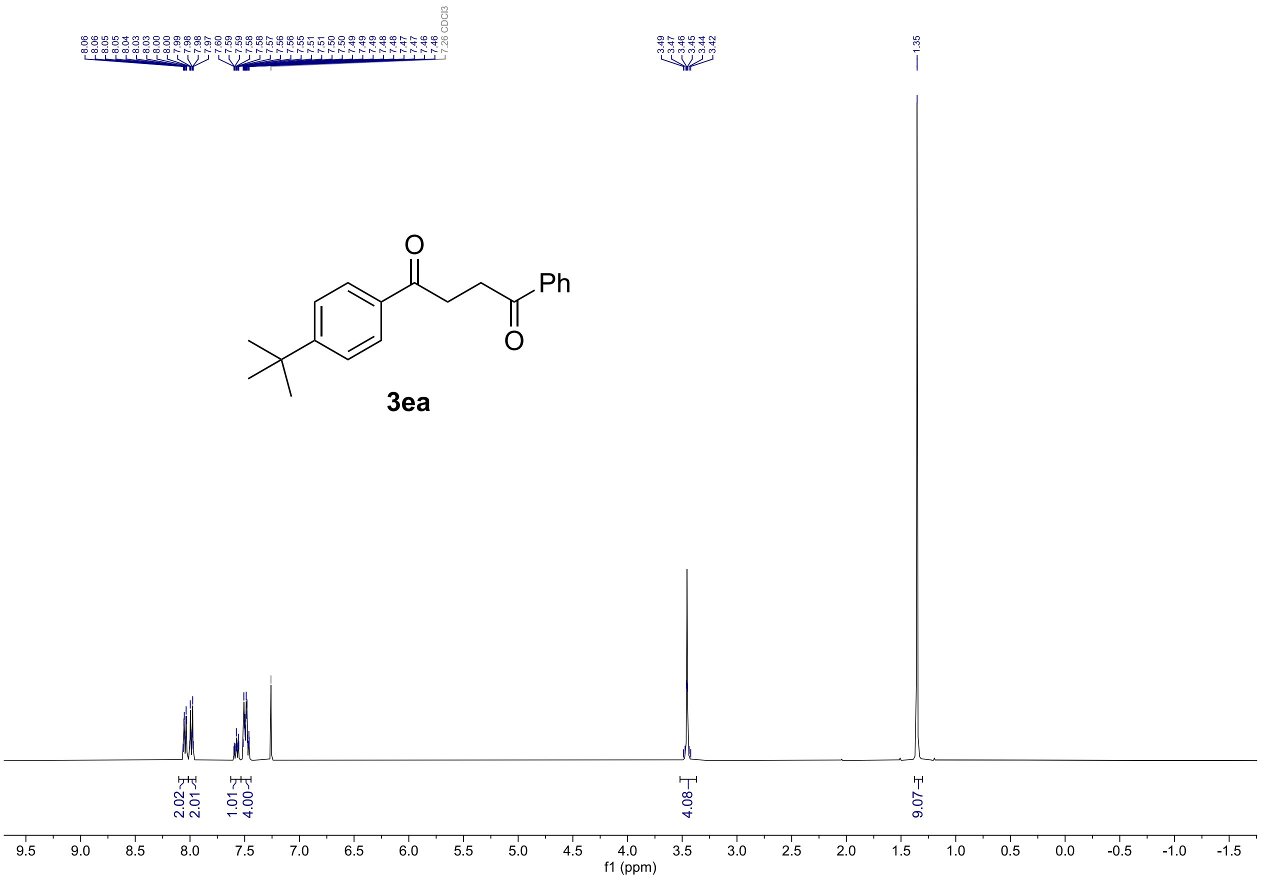

Figure S34. ^{1}H NMR of 1-(4-(*tert*-butyl)phenyl)-4-phenylbutane-1,4-dione (**3ea**) in $CDCl_3$ measured at 400 MHz.

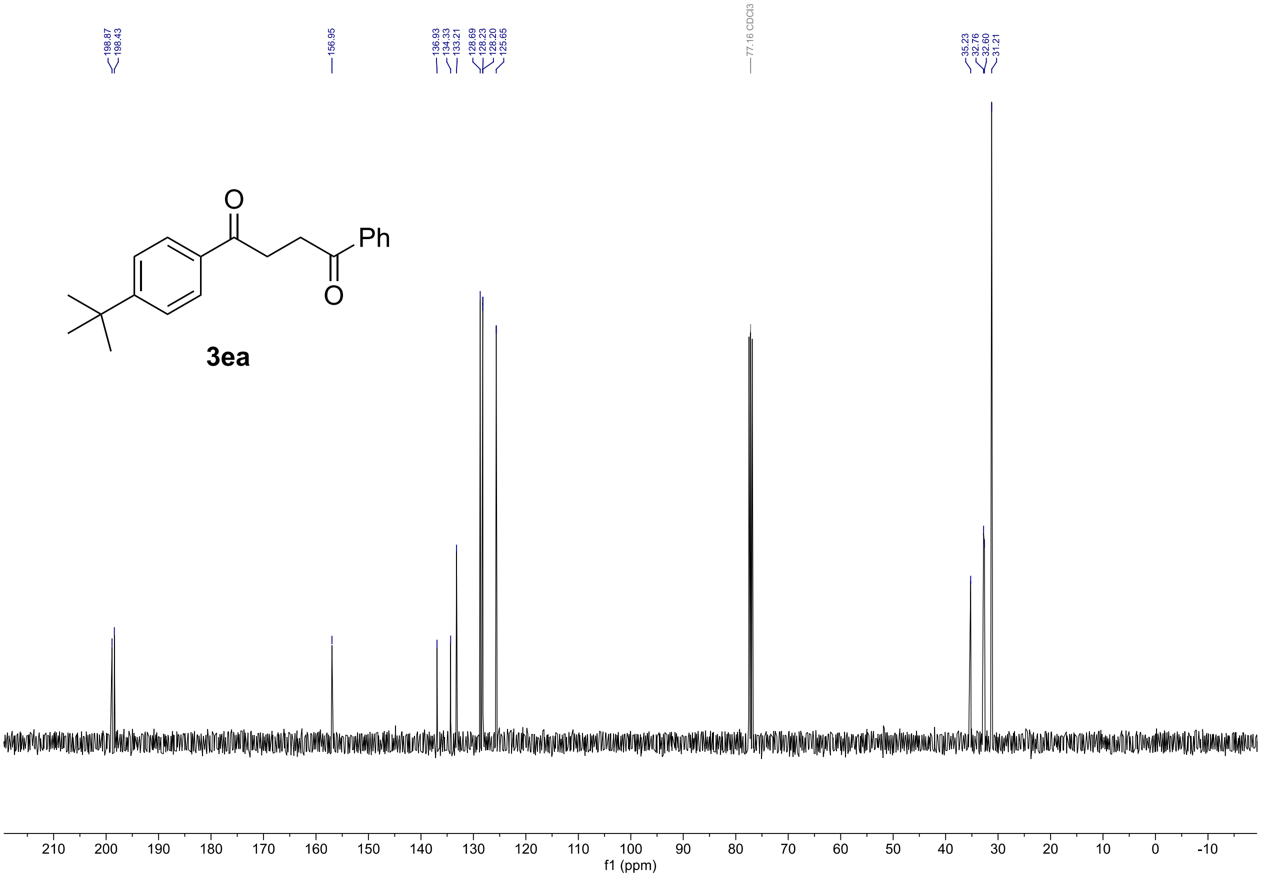

Figure S35. ^{13}C NMR of 1-(4-(*tert*-butyl)phenyl)-4-phenylbutane-1,4-dione (**3ea**) in $CDCl_3$ measured at 101 MHz.

1-phenyl-4-(4-(trifluoromethyl)phenyl)butane-1,4-dione

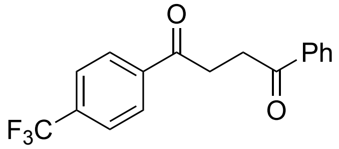

3fa

3fa was synthesized following the general procedure **4**, employing **1f** (202 mg, 1.00 mmol, 1.00 eq), **2a** (321 mg, 1.50 mmol, 1.50 eq), CuCl (149 mg, 1.50 mmol, 1.50 eq), K_2CO_3 (138 mg, 1.00 mmol, 1.00 eq) at 80°C, and isolated via flash chromatography (23 g SiO_2, gradient from 10:90 to 20:80 EtOAc/*n*-hexane over 10 CV, gradient from 20:80 to 50:50 EtOAc/*n*-hexane over 5 CV) as a yellow solid (267 mg, 87%).

$C_{17}H_{13}F_3O_2$ (306,28 g/mol)

mp: 129-130°C

Rf: 0.24 (EtOAc/n-hexane = 1:10) [UV]

^{1}H NMR (400 MHz, $CDCl_3$): δ = 8.15 (d, *J* = 8.1 Hz, 2H), 8.04 (m, 2H), 7.76 (d, *J* = 8.1 Hz, 2H), 7.62 – 7.56 (m, 1H), 7.49 (m, 2H), 3.49 (m, 4H).

^{13}C NMR (101 MHz, $CDCl_3$): δ = 198.4, 197.9, 139.6, 136.7, 34.5 (q, *J* = 32.6 Hz), 133.4, 128.8, 128.6, 128.2, 125.8 (q, *J* = 3.7 Hz), 123.8 (q, *J* = 272.7 Hz), 32.9, 32.7.

^{19}F NMR (376 MHz, $CDCl_3$): δ = -63.34 (s, 3F).

HRMS (ESI) m/z: $[M+Na]^+$ Calcd for $C_{17}H_{13}F_3O_2Na$ 329.0760; Found 329.0761.

IR (ATR, ṽ): 1672 (m, CO).

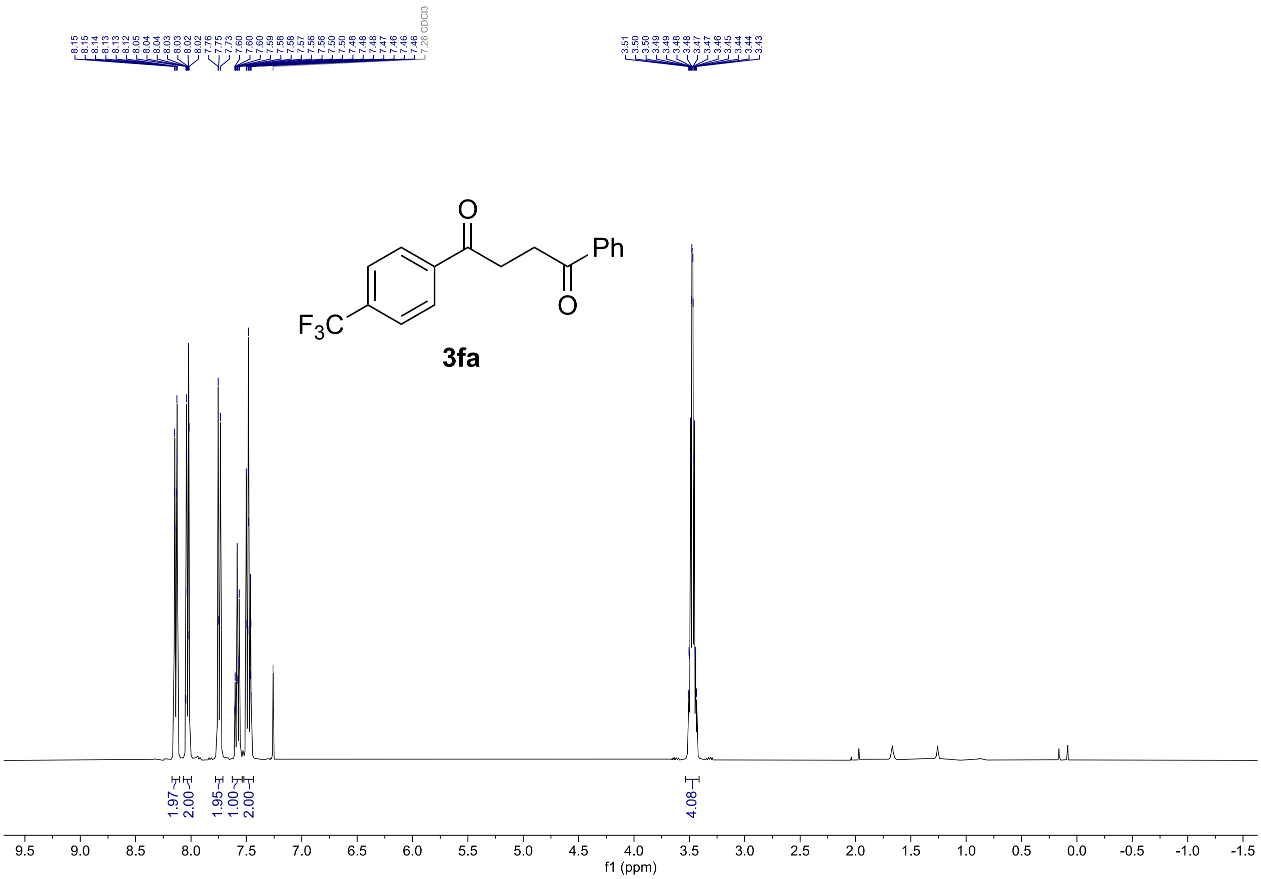

Figure S36. ^{1}H NMR of 1-phenyl-4-(4-(trifluoromethyl)phenyl)butane-1,4-dione (**3fa**) in $CDCl_3$ measured at 400 MHz.

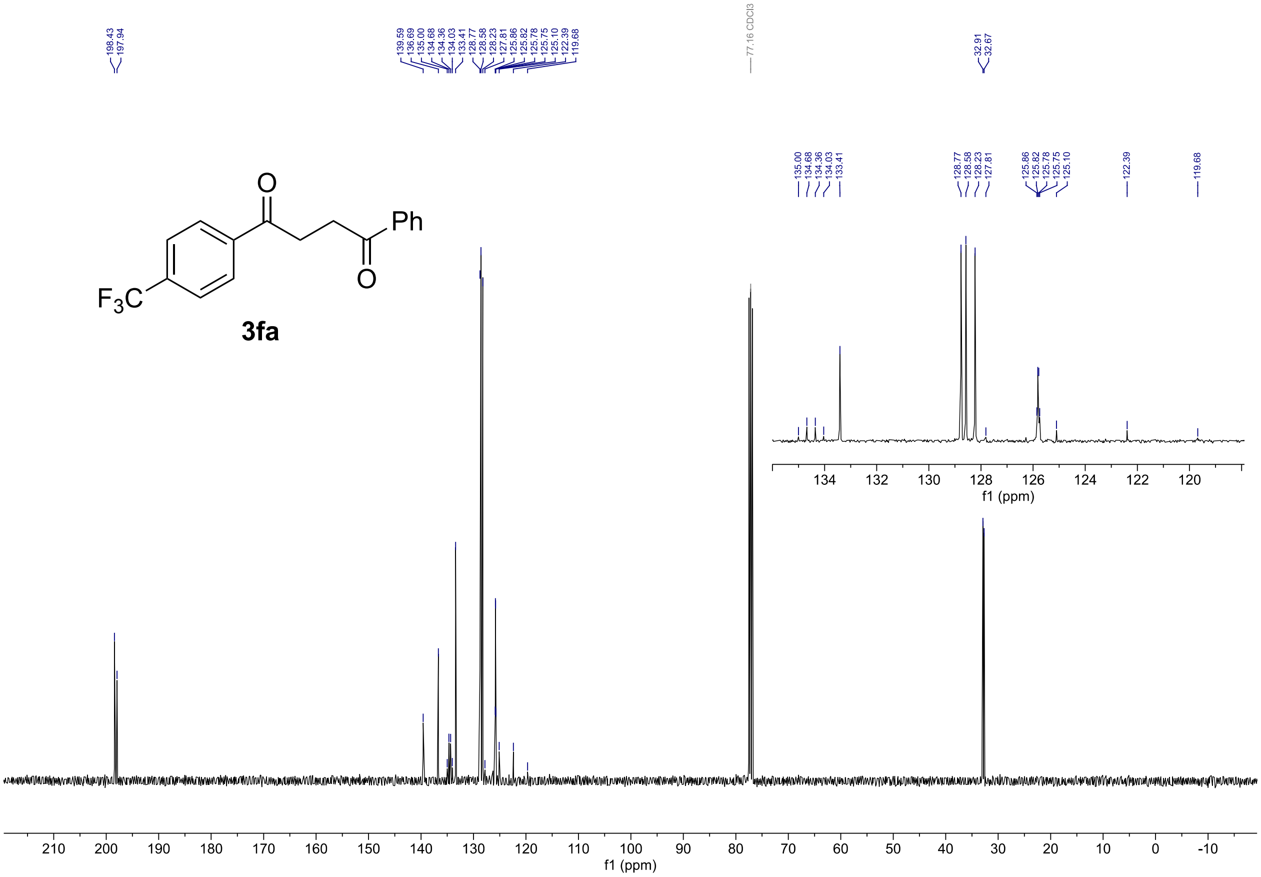

Figure S37. $^{1}3C$ NMR of 1-phenyl-4-(4-(trifluoromethyl)phenyl)butane-1,4-dione (**3fa**) in $CDCl_3$ measured at 101 MHz.

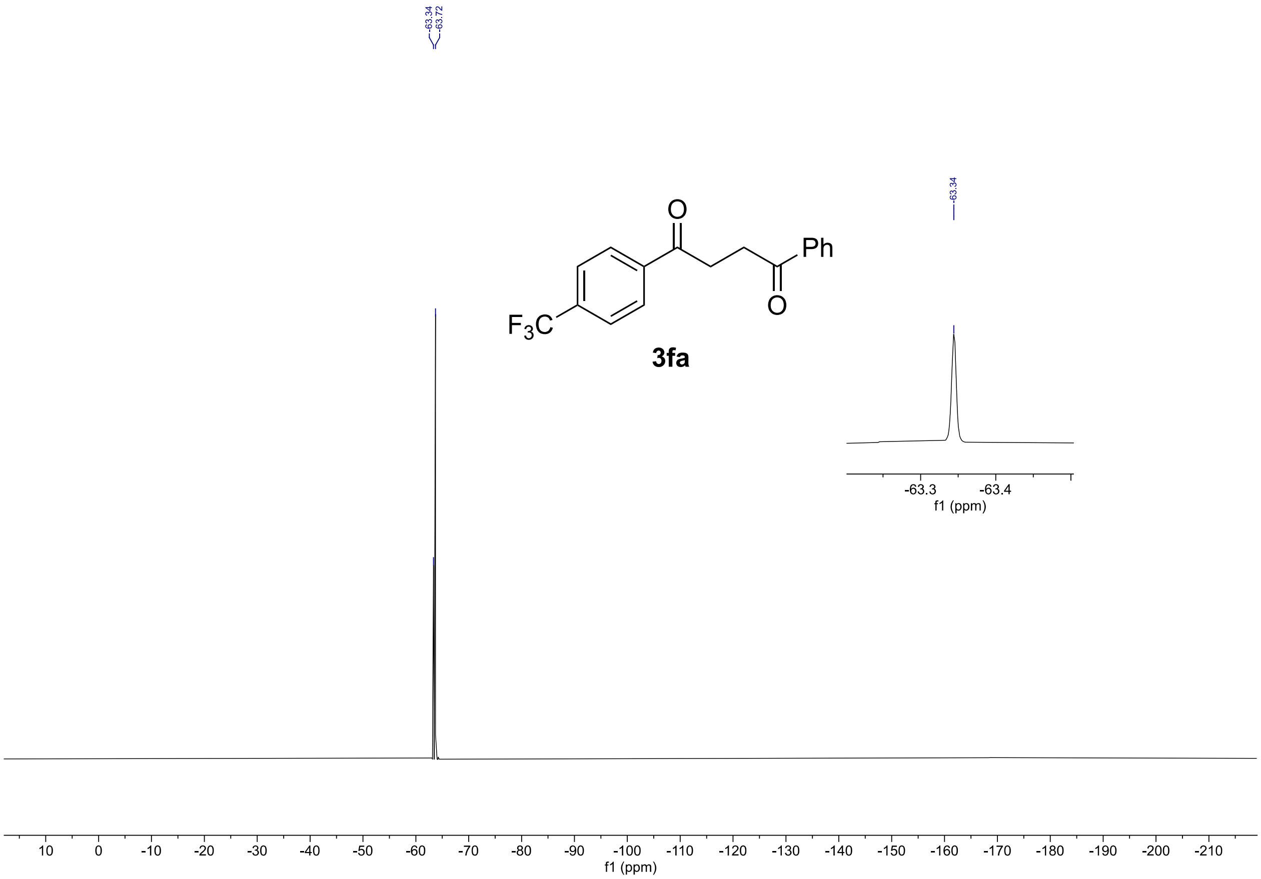

Figure S38. ^{19}F NMR of 1-phenyl-4-(4-(trifluoromethyl)phenyl)butane-1,4-dione (**3fa**) in $CDCl_3$ measured at 376 MHz.

1-(naphthalen-2-yl)-4-phenylbutane-1,4-dione

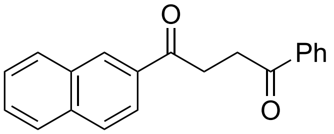

3ga

3ga was synthesized following the general procedure **4**, employing **1g** (184 mg, 1.00 mmol, 1.00 eq), **2a** (321 mg, 1.50 mmol, 1.50 eq), CuCl (149 mg, 1.50 mmol, 1.50 eq), K_2CO_3 (138 mg, 1.00 mmol, 1.00 eq) at 80°C, and isolated via flash chromatography (23 g SiO_2, gradient from 10:90 to 20:80 EtOAc/*n*-hexane over 10 CV, gradient from 20:80 to 50:50 EtOAc/*n*-hexane over 5 CV) as a pale yellow solid (263 mg, 91%).

$C_{20}H_{16}O_2$ (288,35 g/mol)

mp: 152-153°C

Rf: 0.28 (EtOAc/n-hexane = 1:10) [UV]

^{1}H NMR (400 MHz, $CDCl_3$): δ = 8.59 (d, *J* = 2.0 Hz, 1H), 8.08 (td, *J* = 7.9, 1.7 Hz, 3H), 7.99 (dd, *J* = 8.1, 1.2 Hz, 1H), 7.94 – 7.85 (m, 2H), 7.65 – 7.53 (m, 3H), 7.53 – 7.46 (m, 2H), 3.67 – 3.47 (m, 4H).

^{13}C NMR (101 MHz, $CDCl_3$): δ = 198.9, 198.8, 136.9, 135.8, 134.2, 133.3, 132.7, 130.0, 129.8, 128.8, 128.6, 128.3, 127.9, 126.9, 124.0, 32.9, 32.8.

HRMS (ESI) m/z: $[M+Na]^+$ Calcd for $C_{20}H_{16}O_2Na$ 311.1043; Found 311.1042.

IR (ATR, ṽ): 1672 (s, CO).

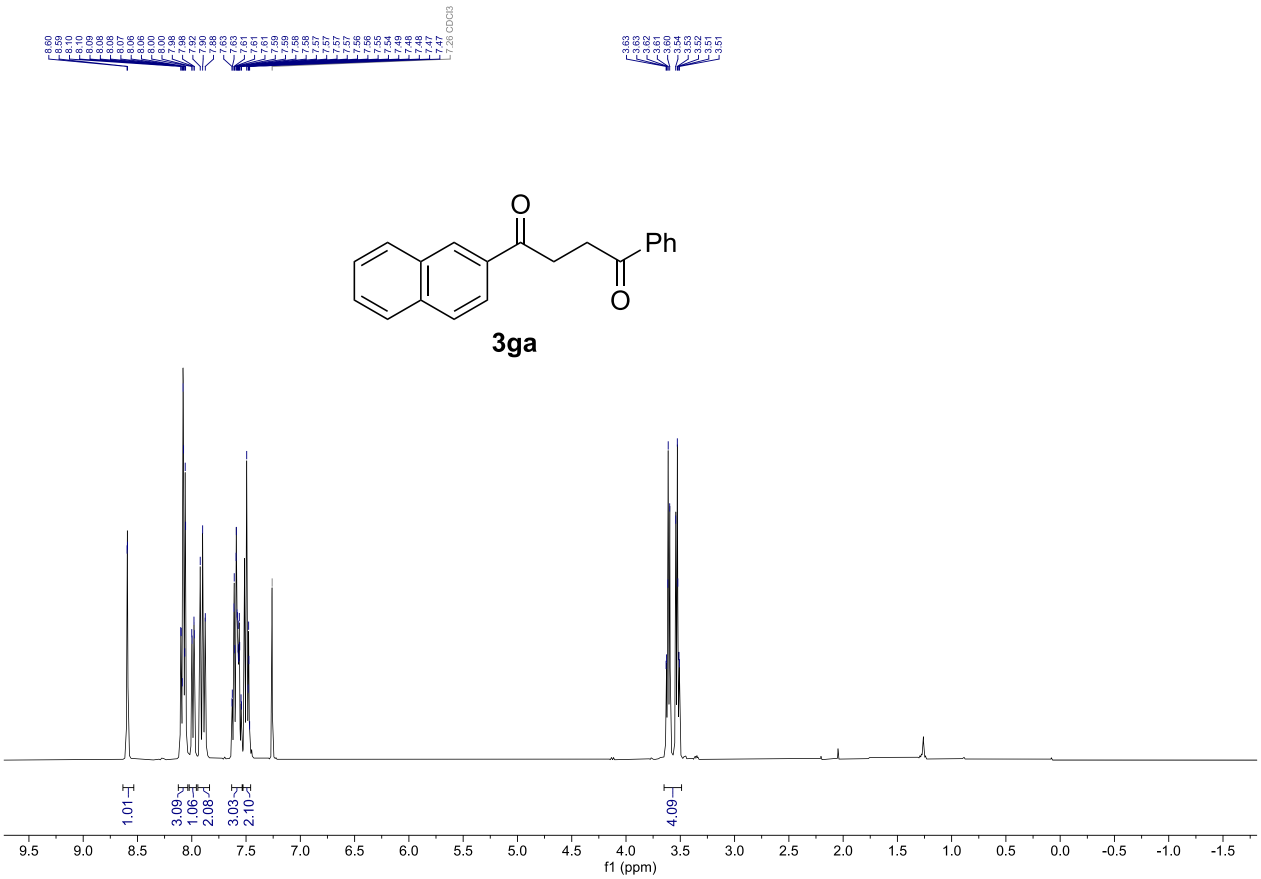

Figure S39. ^{1}H NMR of 1-(naphthalen-2-yl)-4-phenylbutane-1,4-dione (**3ga**) in $CDCl_3$ measured at 400 MHz.

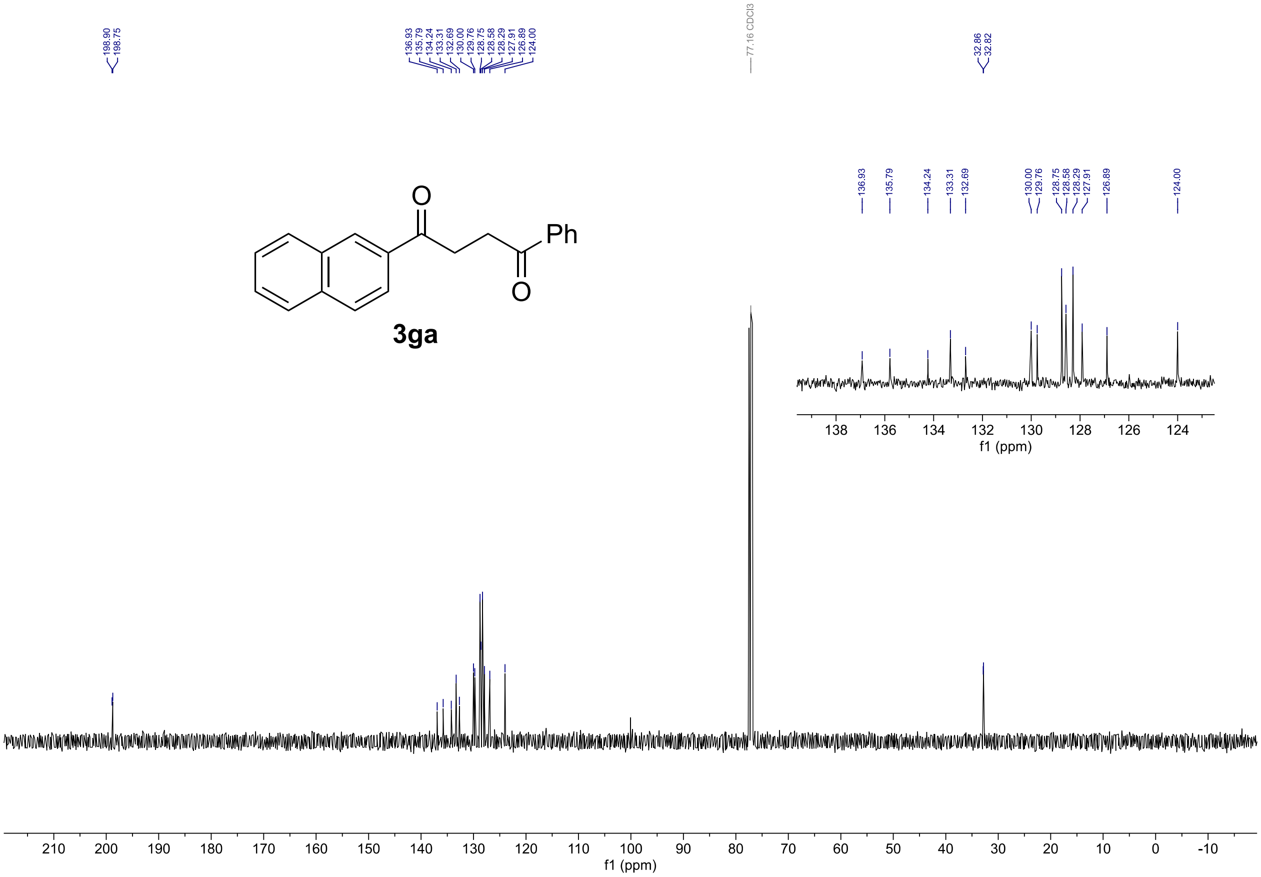

Figure S40. ^{13}C NMR of 1-(naphthalen-2-yl)-4-phenylbutane-1,4-dione (**3ga**) in $CDCl_3$ measured at 101 MHz.

1,4-diphenylbutane-1,4-dione

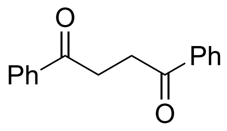

3ha

3ha was synthesized following the general procedure **4**, employing **1h** (134 mg, 1.00 mmol, 1.00 eq), **2a** (321 mg, 1.50 mmol, 1.50 eq), CuCl (149 mg, 1.50 mmol, 1.50 eq), K_2CO_3 (138 mg, 1.00 mmol, 1.00 eq) at 80°C, and isolated via flash chromatography (23 g SiO_2, gradient from 10:90 to 20:80 EtOAc/*n*-hexane over 10 CV, gradient from 20:80 to 50:50 EtOAc/*n*-hexane over 5 CV) as a pale yellow solid (219 mg, 92%).

$C_{16}H_{14}O_2$ (238,29 g/mol)

mp: 145°C

Rf: 0.27 (EtOAc/n-hexane = 1:10) [UV]

^{1}H NMR (400 MHz, $CDCl_3$): δ = 8.08 – 8.02 (m, 4H), 7.61 – 7.55 (m, 2H), 7.49 (tt, *J* = 6.6, 1.4 Hz, 4H), 3.47 (s, 4H).

^{13}C NMR (101 MHz, $CDCl_3$): δ = 198.8, 136.9, 133.3, 128.7, 128.3, 32.7.

HRMS (ESI) m/z: $[M+Na]^+$ Calcd for $C_{16}H_{14}O_2Na$ 261.0886; Found 261.0890.

IR (ATR, ṽ): 1672 (s, CO)

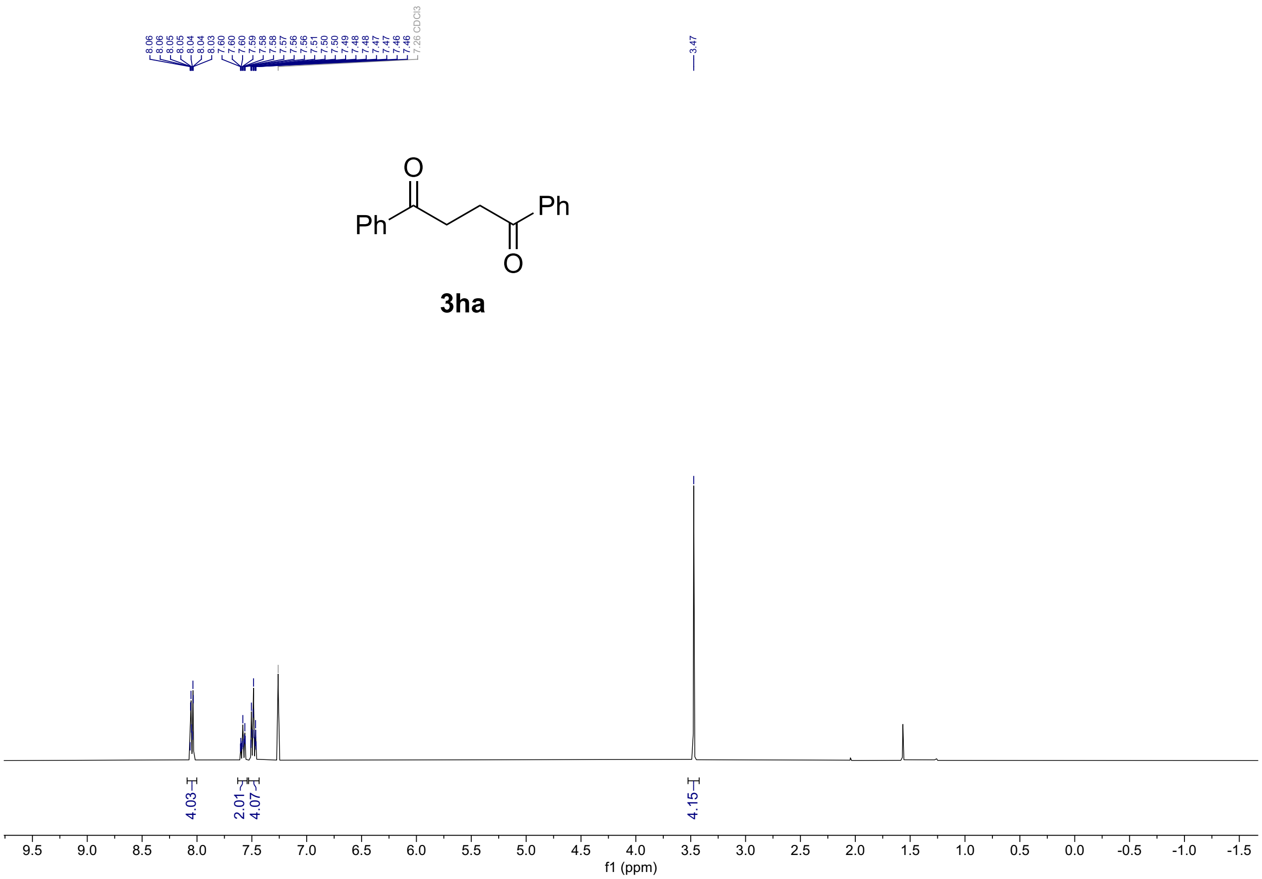

Figure S41. ^{1}H NMR of 1,4-diphenylbutane-1,4-dione (**3ha**) in $CDCl_3$ measured at 400 MHz.

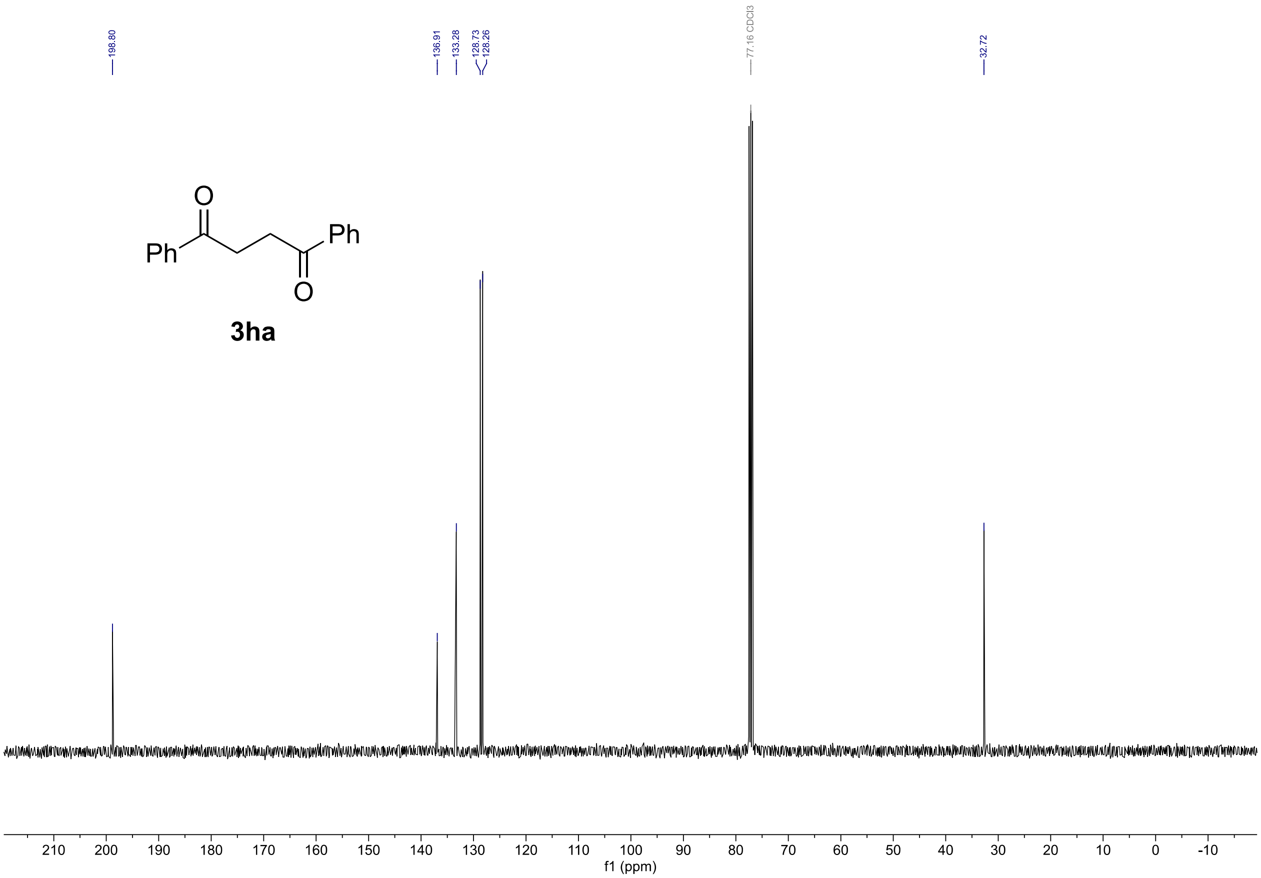

Figure S42. ^{13}C NMR of 1,4-diphenylbutane-1,4-dione (**3ha**) in $CDCl_3$ measured at 101 MHz.

1,6-diphenylhexane-1,4-dione

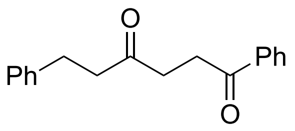

3ia

3ia was synthesized following the general procedure **4**, employing **1i** (162 mg, 1.00 mmol, 1.00 eq), **2a** (321 mg, 1.50 mmol, 1.50 eq), CuCl (149 mg, 1.50 mmol, 1.50 eq), K_2CO_3 (138 mg, 1.00 mmol, 1.00 eq) at 80°C, and isolated via flash chromatography (23 g SiO_2, gradient from 10:90 to 20:80 EtOAc/*n*-hexane over 10 CV, gradient from 20:80 to 50:50 EtOAc/*n*-hexane over 5 CV) as an off-white solid (231 mg, 87%)

$C_{18}H_{18}O_2$ (266,34 g/mol)

mp: 87-88°C

Rf: 0.21 (EtOAc/n-hexane = 1:10) [UV]

^{1}H NMR (400 MHz, $CDCl_3$): δ = 8.01 – 7.95 (m, 2H), 7.60 – 7.54 (m, 1H), 7.46 (m, 2H), 7.32 – 7.27 (m, 2H), 7.24 – 7.17 (m, 3H), 3.32 – 3.25 (m, 2H), 2.95 (m, 2H), 2.91 – 2.82 (m, 4H).

^{13}C NMR (101 MHz, $CDCl_3$): δ = 208.7, 198.7, 141.2, 136.8, 133.3, 128.7, 128.6, 128.5, 128.2, 126.2, 44.6, 36.5, 32.5, 29.9.

HRMS (ESI) m/z: $[M+Na]^+$ Calcd for $C_{18}H_{18}O_2Na$ 289.1199; Found 289.1201.

IR (ATR, ṽ): 1705 (m, CO), 1679 (s, CO).

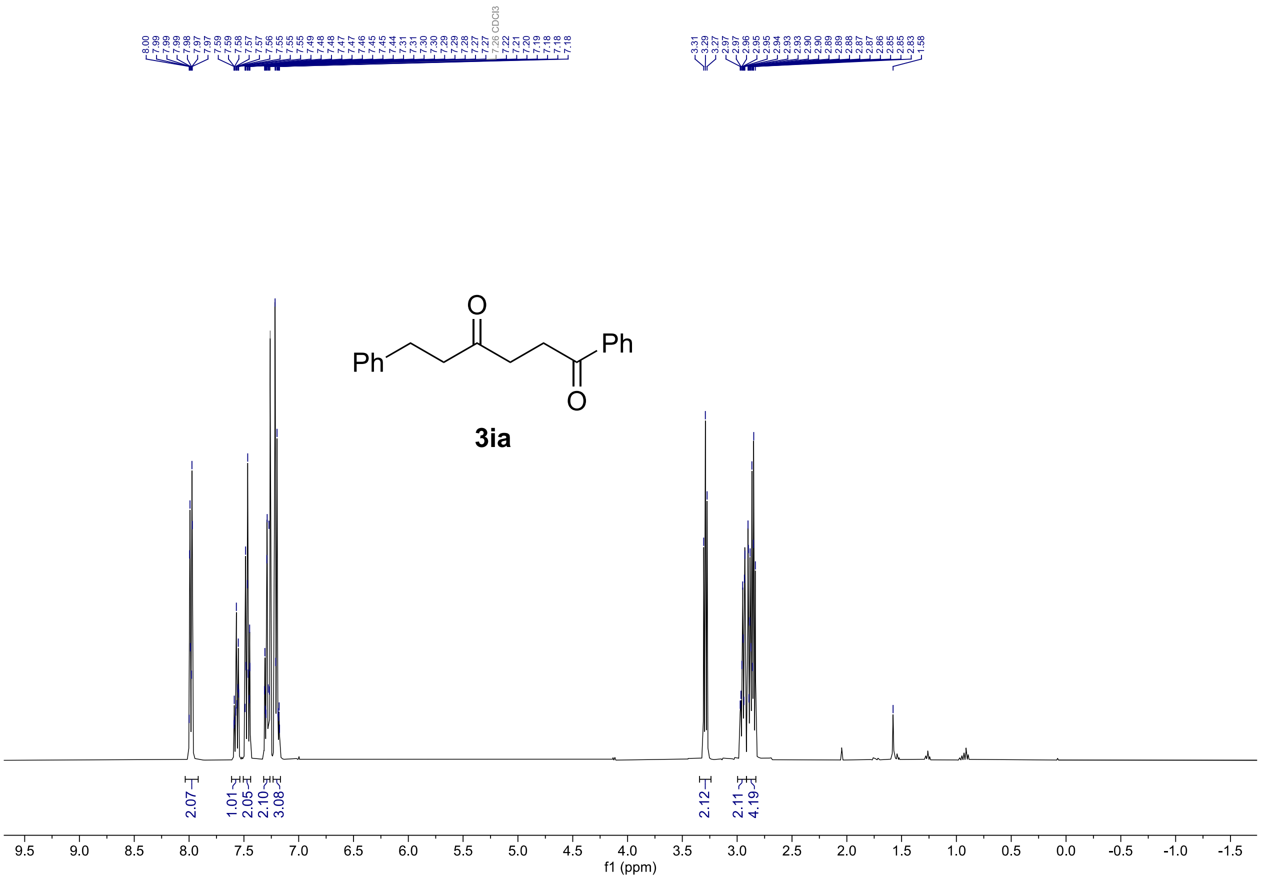

Figure S43. ^{1}H NMR of 1,6-diphenylhexane-1,4-dione (**3ia**) in $CDCl_3$ measured at 400 MHz.

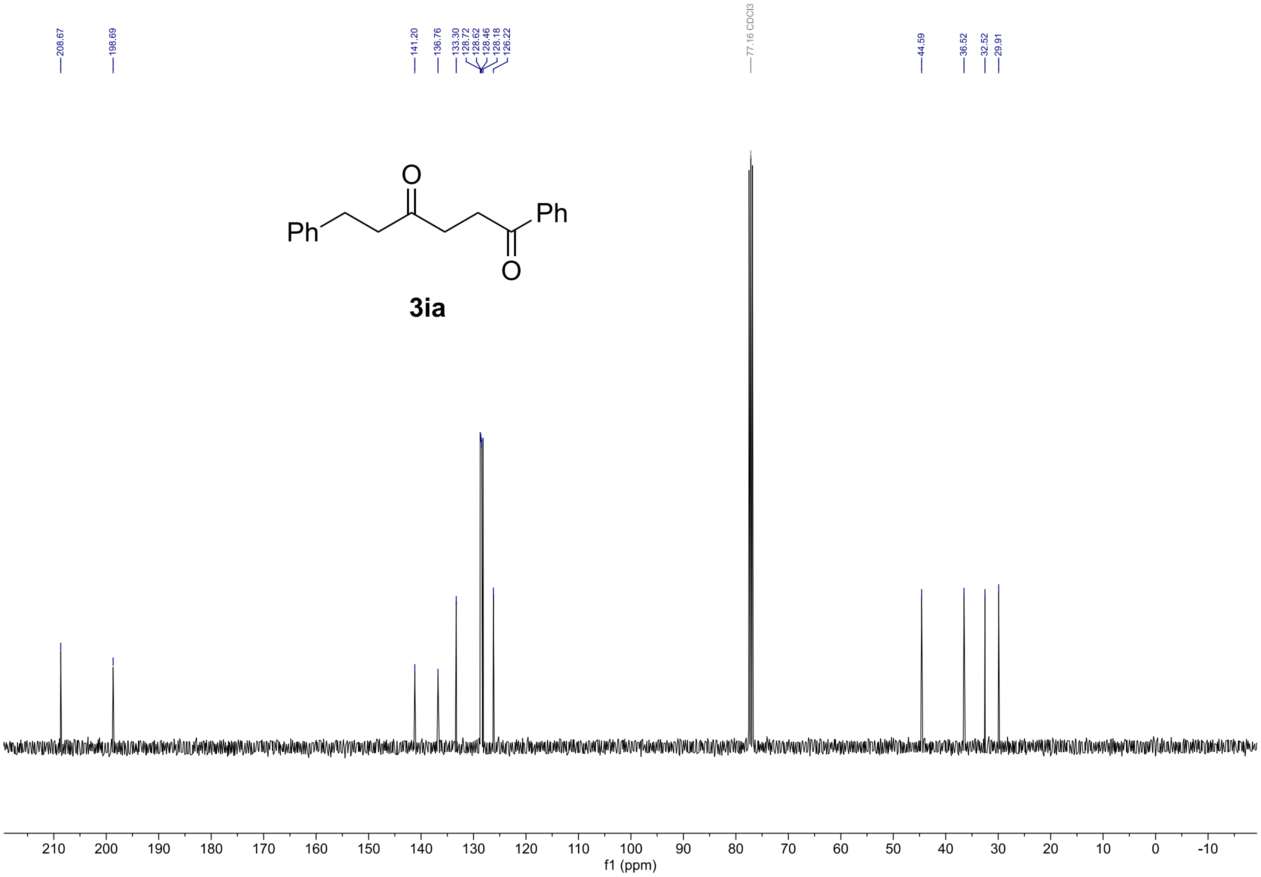

Figure S44. ^{13}C NMR of 1,6-diphenylhexane-1,4-dione (**3ia**) in $CDCl_3$ measured at 101 MHz.

1,5,5-triphenylpentane-1,4-dione

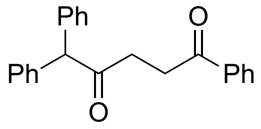

3ja

3ja was synthesized following the general procedure **4**, employing **1j** (224 mg, 1.00 mmol, 1.00 eq), **2a** (321 mg, 1.50 mmol, 1.50 eq), CuCl (149 mg, 1.50 mmol, 1.50 eq), K_2CO_3 (138 mg, 1.00 mmol, 1.00 eq) at 80°C, and isolated via flash chromatography (23 g SiO_2, gradient from 10:90 to 20:80 EtOAc/*n*-hexane over 10 CV, gradient from 20:80 to 50:50 EtOAc/*n*-hexane over 5 CV) as a yellowish solid (271 mg, 83%).

$C_{23}H_{20}O_2$ (328,41 g/mol)

mp: 60-61°C

Rf: 0.24 (EtOAc/n-hexane = 1:10) [UV]

^{1}H NMR (400 MHz, $CDCl_3$): δ = 7.97 – 7.92 (m, 2H), 7.56 – 7.50 (m, 1H), 7.42 (m, 2H), 7.32 (m, 4H), 7.25 – 7.21 (m, 6H), 5.29 (s, 1H), 3.27 (dd, *J* = 6.8, 5.9 Hz, 2H), 2.97 (dd, *J* = 6.9, 5.8 Hz, 2H).

^{13}C NMR (101 MHz, $CDCl_3$): δ = 207.7, 198.6, 138.5, 136.7, 133.2, 129.2, 128.8, 128.7, 128.1, 127.3, 64.5, 36.7, 33.0.

HRMS (ESI) m/z: $[M+Na]^+$ Calcd for $C_{23}H_{20}O_2Na$ 351.1356; Found 351.1354.

IR (ATR, ṽ): 1713 (m, CO), 1683 (s, CO).

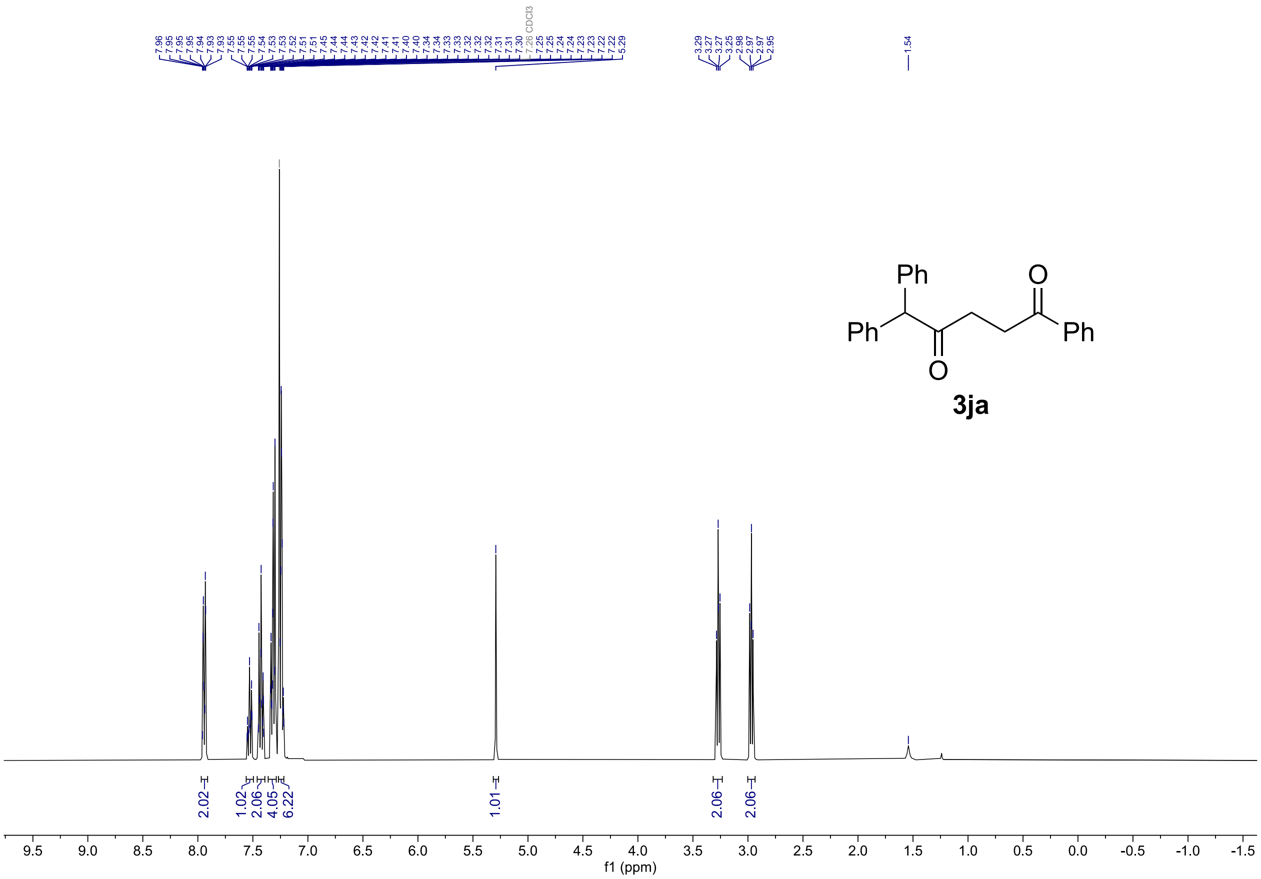

Figure S45. ^{1}H NMR of 1,5,5-triphenylpentane-1,4-dione (**3ja**) in $CDCl_3$ measured at 400 MHz.

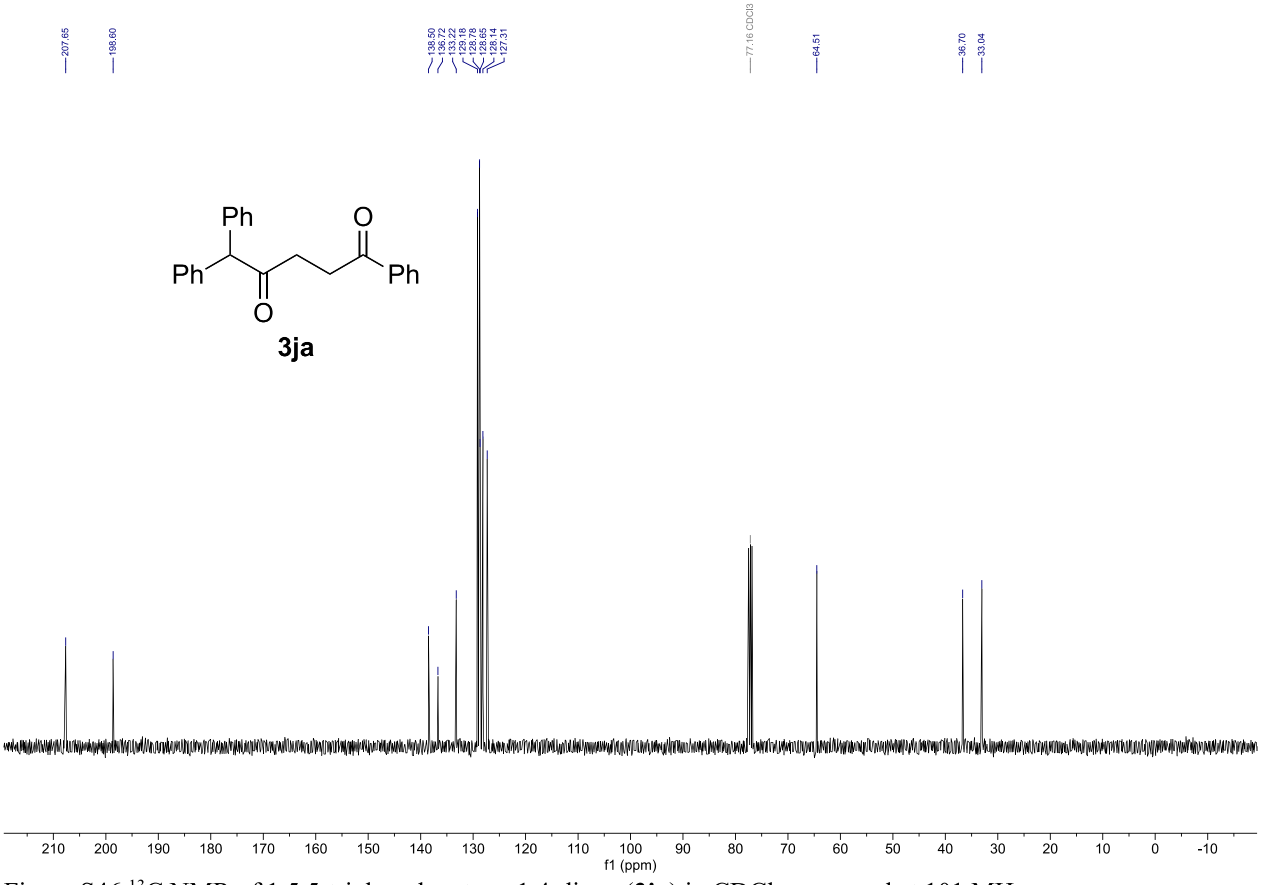

Figure S46 ^{13}C NMR of 1,5,5-triphenylpentane-1,4-dione (**3ja**) in $CDCl_3$ measured at 101 MHz.

1-cyclohexyl-4-phenylbutane-1,4-dione

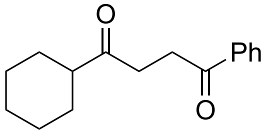

3ka

3ka was synthesized following the general procedure **4**, employing **1k** (140 mg, 1.00 mmol, 1.00 eq), **2a** (321 mg, 1.50 mmol, 1.50 eq), CuCl (149 mg, 1.50 mmol, 1.50 eq), K_2CO_3 (138 mg, 1.00 mmol, 1.00 eq) at 80°C, and isolated via flash chromatography (23 g SiO_2, gradient from 10:90 to 20:80 EtOAc/*n*-hexane over 10 CV, gradient from 20:80 to 50:50 EtOAc/*n*-hexane over 5 CV) as a yellowish solid (207 mg, 85%).

$C_{16}H_{20}O_2$ (244,33 g/mol)

mp: 51-52°C

Rf: 0.36 (EtOAc/n-hexane = 1:10) [UV]

^{1}H NMR (400 MHz, $CDCl_3$): δ = 8.01 – 7.96 (m, 2H), 7.59 – 7.52 (m, 1H), 7.49 – 7.42 (m, 2H), 3.27 (dd, *J* = 6.8, 5.7 Hz, 2H), 2.90 (dd, *J* = 6.9, 5.8 Hz, 2H), 2.47 (tt, *J* = 11.2, 3.5 Hz, 1H), 1.97 – 1.89 (m, 2H), 1.84 – 1.76 (m, 2H), 1.72 – 1.64 (m, 1H), 1.45 – 1.15 (m, 5H).

^{13}C NMR (101 MHz, $CDCl_3$): δ = 212.7, 198.9, 136.9, 133.2, 128.7, 128.2, 51.0, 34.3, 32.4, 28.7, 26.0, 25.8.

HRMS (ESI) m/z: $[M+Na]^+$ Calcd for $C_{16}H_{20}O_2Na$ 267.1356; Found 267.1359.

IR (ATR, ṽ): 1672 (s, CO).

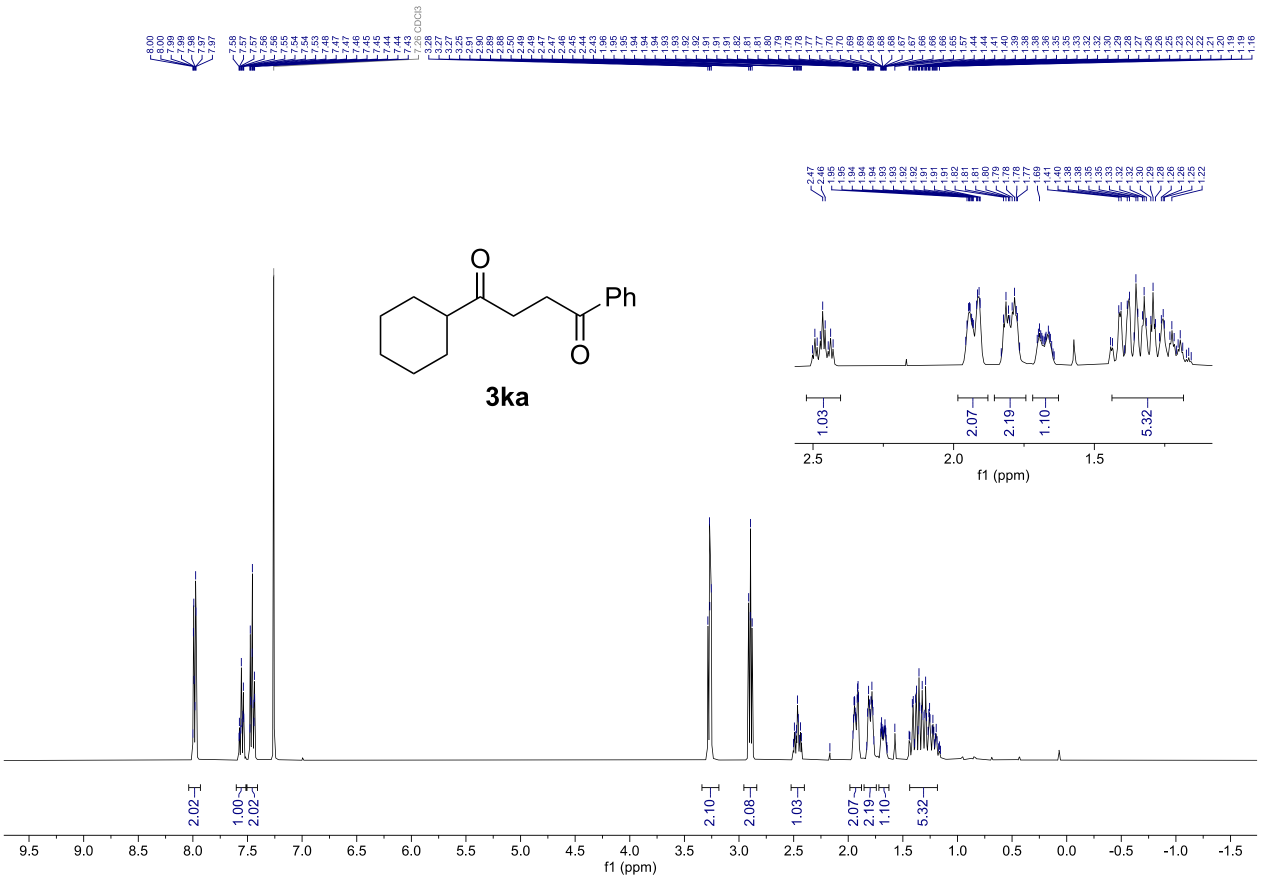

Figure S47. ^{1}H NMR of 1-cyclohexyl-4-phenylbutane-1,4-dione (**3ka**) in $CDCl_3$ measured at 400 MHz.

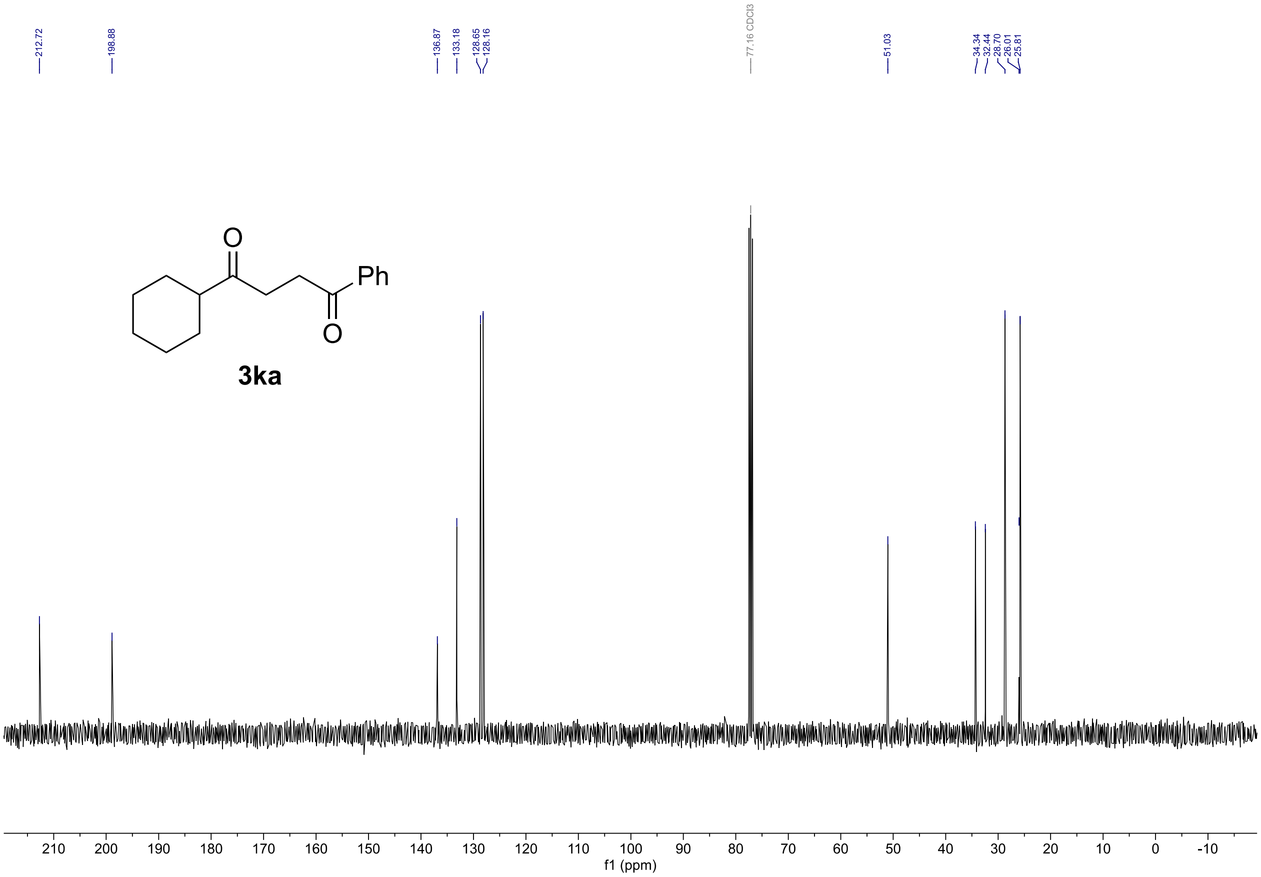

Figure S48. ^{13}C NMR of 1-cyclohexyl-4-phenylbutane-1,4-dione (**3ka**) in $CDCl_3$ measured at 101 MHz.

1-cyclobutyl-4-phenylbutane-1,4-dione

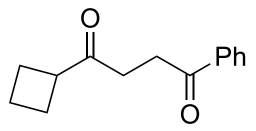

3la

3la was synthesized following the general procedure **4**, employing **1l** (112 mg, 1.00 mmol, 1.00 eq), **2a** (321 mg, 1.50 mmol, 1.50 eq), CuCl (149 mg, 1.50 mmol, 1.50 eq), K_2CO_3 (138 mg, 1.00 mmol, 1.00 eq) at 80°C, isolated via flash chromatography (23 g SiO_2, gradient from 10:90 to 20:80 EtOAc/*n*-hexane over 10 CV, gradient from 20:80 to 50:50 EtOAc/*n*-hexane over 5 CV) as a colourless solid (194 mg, 90%).

$C_{14}H_{16}O_2$ (216,28 g/mol)

mp: 28-29°C

Rf: 0.31 (EtOAc/n-hexane = 1:10) [UV]

^{1}H NMR (400 MHz, $CDCl_3$): δ = 8.01 – 7.96 (m, 2H), 7.59 – 7.53 (m, 1H), 7.46 (tt, *J* = 6.6, 1.5 Hz, 2H), 3.37 (m, 1H), 3.28 (dd, *J* = 6.9, 5.8 Hz, 2H), 2.81 (dd, *J* = 6.8, 5.8 Hz, 2H), 2.37 – 2.25 (m, 2H), 2.25 – 2.14 (m, 2H), 2.06 – 1.93 (m, 1H), 1.90 – 1.78 (m, 1H).

^{13}C NMR (101 MHz, $CDCl_3$): δ = 210.3, 198.6, 136.8, 133.1, 128.6, 128.0, 65.8, 45.5, 33.4, 32.2, 24.4, 17.8, 15.3.

HRMS (ESI) m/z: $[M+Na]^+$ Calcd for $C_{14}H_{16}O_2Na$ 239.1043; Found 239.1046.

IR (ATR, ṽ): 1682 (vs, CO).

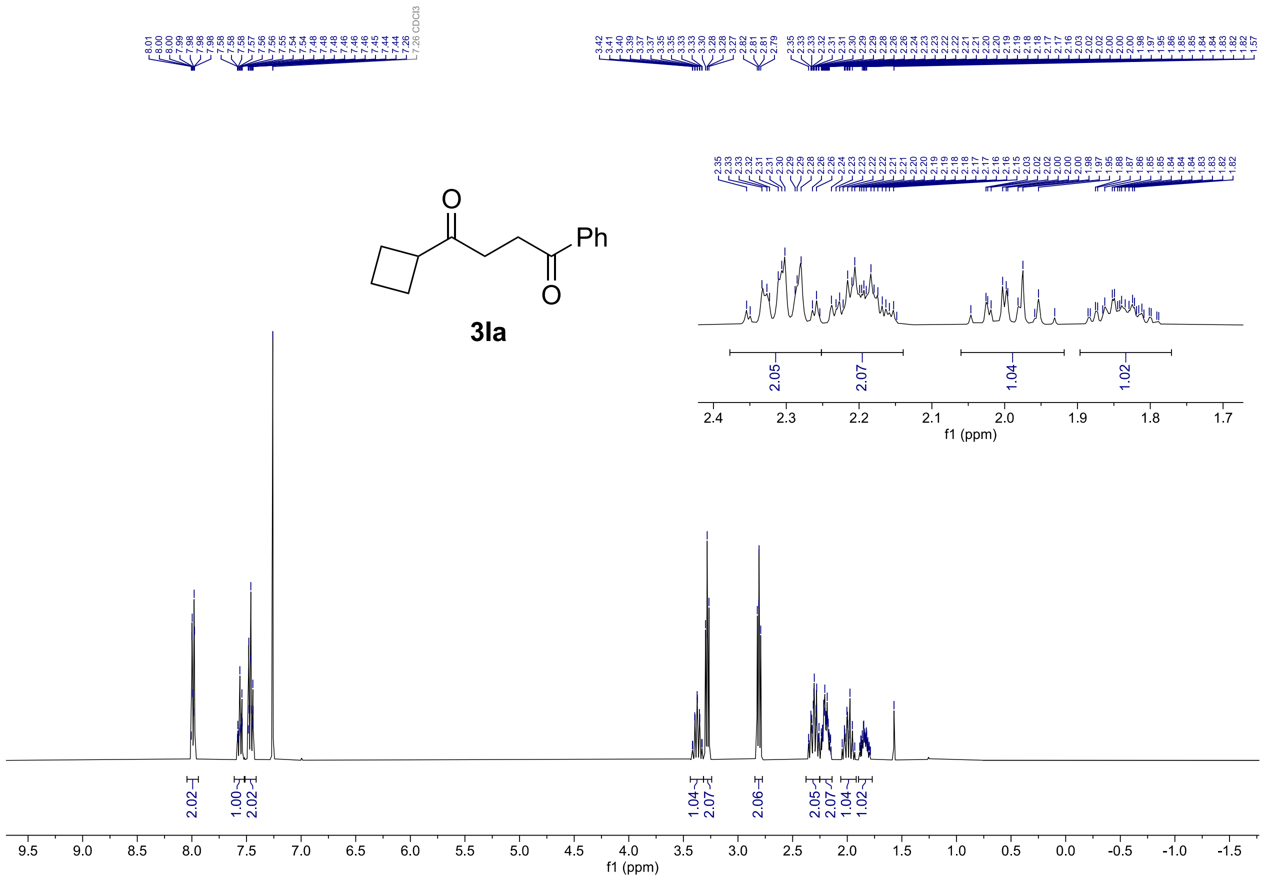

Figure S49. ^{13}C NMR of 1-cyclobutyl-4-phenylbutane-1,4-dione (**3la**) in $CDCl_3$ measured at 400 MHz.

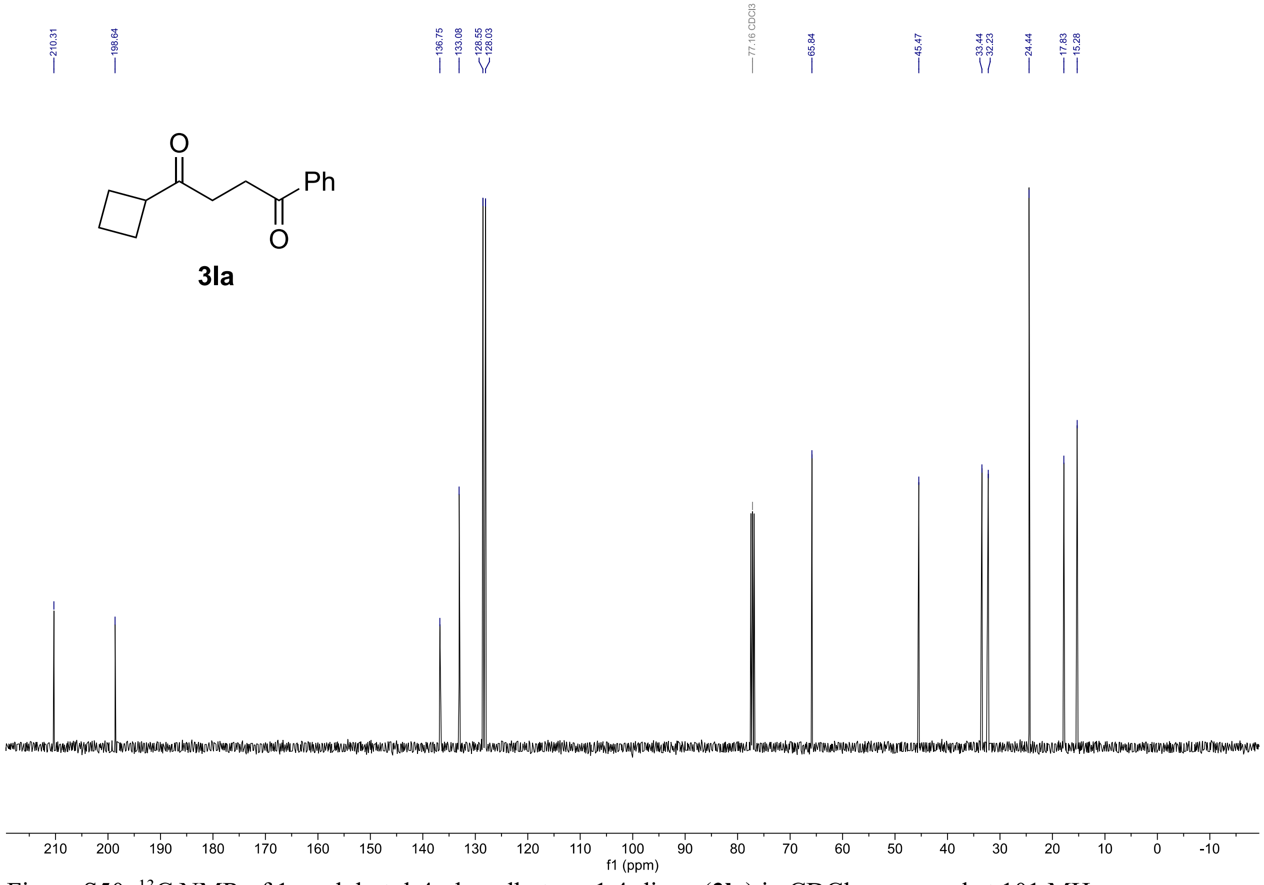

Figure S50. ^{13}C NMR of 1-cyclobutyl-4-phenylbutane-1,4-dione (**3la**) in $CDCl_3$ measured at 101 MHz.

***tert*-butyl 4-(4-oxo-4-phenylbutanoyl)piperidine-1-carboxylate**

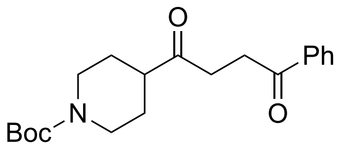

3ma

3ma was synthesized following the general procedure **4**, employing **1m** (241 mg, 1.00 mmol, 1.00 eq), **2a** (321 mg, 1.50 mmol, 1.50 eq), CuCl (149 mg, 1.50 mmol, 1.50 eq), K_2CO_3 (138 mg, 1.00 mmol, 1.00 eq) at 80°C, and isolated via flash chromatography (23 g SiO_2, gradient from 10:90 to 50:50 EtOAc/*n*-hexane over 10 CV, gradient from 50:50 to 100:0 EtOAc/*n*-hexane over 5 CV) as a yellow liquid (283 mg, 83%).

$C_{20}H_{27}NO_4$ (345,44 g/mol)

mp: liquid

Rf: 0.09 (EtOAc/n-hexane = 1:5) [UV]

^{1}H NMR (400 MHz, $CDCl_3$): δ = 8.02 – 7.94 (m, 2H), 7.61 – 7.53 (m, 1H), 7.46 (tt, *J* = 6.6, 1.5 Hz, 2H), 4.12 (d, *J* = 7.1 Hz, 2H), 3.31 (t, *J* = 6.2 Hz, 2H), 2.89 (t, *J* = 6.2 Hz, 2H), 2.82 (m, 2H), 2.62 (m, 1H), 1.90 (m, 2H), 1.66 – 1.53 (m, 2H), 1.46 (s, 9H).

^{13}C NMR (101 MHz, $CDCl_3$): δ = 210.7, 198.4, 154.6, 136.5, 133.1, 128.5, 128.0, 79.5, 48.6, 43.2, 34.1, 32.3, 28.4, 27.5.

HRMS (ESI) m/z: $[M+Na]^+$ Calcd for $C_{20}H_{27}NO_4Na$ 368.1832; Found 368.1833.

IR (ATR, ṽ): 1680 (vs, CO)

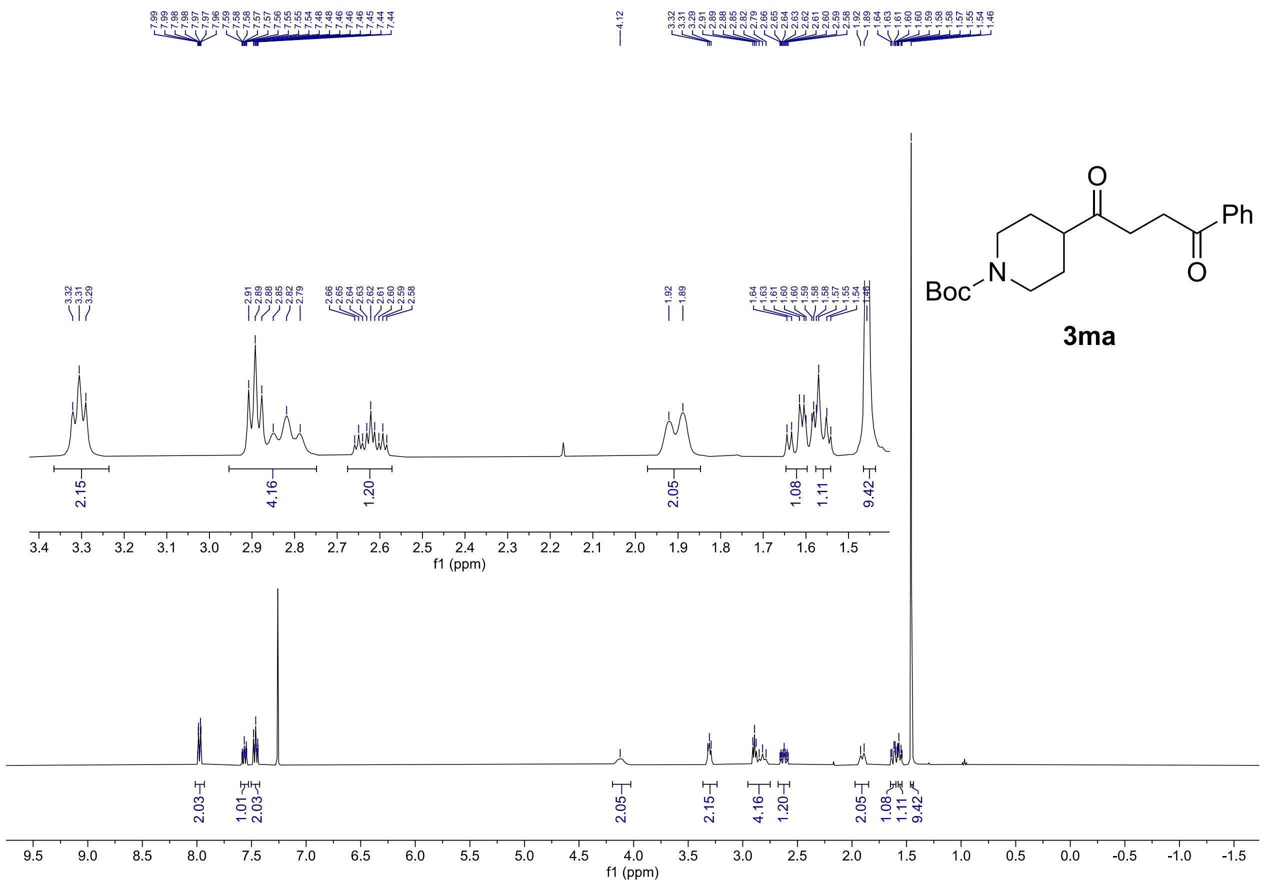

Figure S51. ^{1}H NMR of *tert*-butyl 4-(4-oxo-4-phenylbutanoyl)piperidine-1-carboxylate (**3ma**) in $CDCl_3$ measured at 400 MHz.

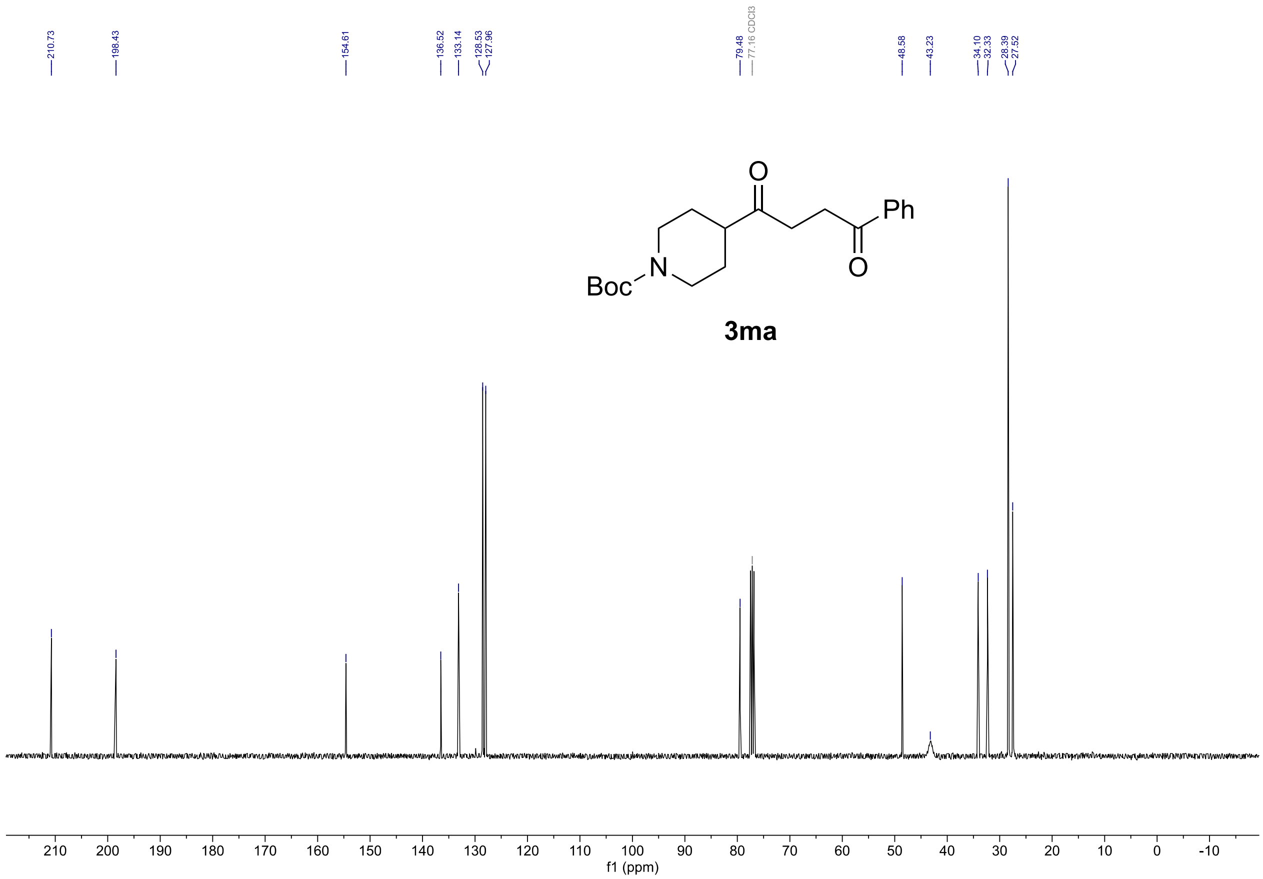

Figure S52. ^{13}C NMR of *tert*-butyl 4-(4-oxo-4-phenylbutanoyl)piperidine-1-carboxylate (**3ma**) in $CDCl_3$ measured at 101 MHz.

1-phenyl-4-(1-tosyl-1*H*-indol-3-yl)butane-1,4-dione

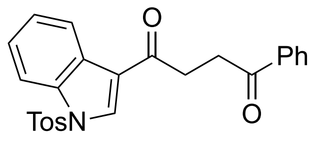

3na

3na was synthesized following the general procedure **4**, employing **1n** (327 mg, 1.00 mmol, 1.00 eq), **2a** (321 mg, 1.50 mmol, 1.50 eq), CuCl (149 mg, 1.50 mmol, 1.50 eq), K_2CO_3 (138 mg, 1.00 mmol, 1.00 eq) at 90°C, and isolated via flash chromatography (23 g SiO_2, gradient from 10:90 to 50:50 EtOAc/*n*-hexane over 10 CV, gradient from 50:50 to 100:0 EtOAc/*n*-hexane over 5 CV) as a pale ochre solid (351 mg, 81%).

$C_{25}H_{21}NO_4S$ (431,51 g/mol)

mp: 136-138°C

Rf: 0.18 (EtOAc/n-hexane = 1:5) [UV]

^{1}H NMR (400 MHz, $CDCl_3$): δ = 8.39 (s, 1H), 8.31 (d, *J* = 7.7 Hz, 1H), 8.04 (d, *J* = 7.7 Hz, 2H), 7.95 (d, *J* = 8.1 Hz, 1H), 7.86 (d, *J* = 8.0 Hz, 2H), 7.58 (t, *J* = 7.3 Hz, 1H), 7.48 (t, *J* = 7.6 Hz, 2H), 7.40 – 7.26 (m, 4H), 3.54 – 3.35 (m, 4H), 2.36 (s, 3H).

^{13}C NMR (101 MHz, $CDCl_3$): δ = 198.8, 194.5, 146.1, 136.8, 135.0, 134.7, 133.4, 132.1, 130.4, 128.8, 128.3, 127.7, 127.3, 125.8, 124.9, 123.2, 121.1, 113.2, 33.9, 32.5, 21.8.

HRMS (ESI) m/z: $[M+Na]^+$ Calcd for $C_{25}H_{21}NO_4SNa$ 454.1084; Found 454.1090.

IR (ATR, ṽ): 1668 (s, CO).

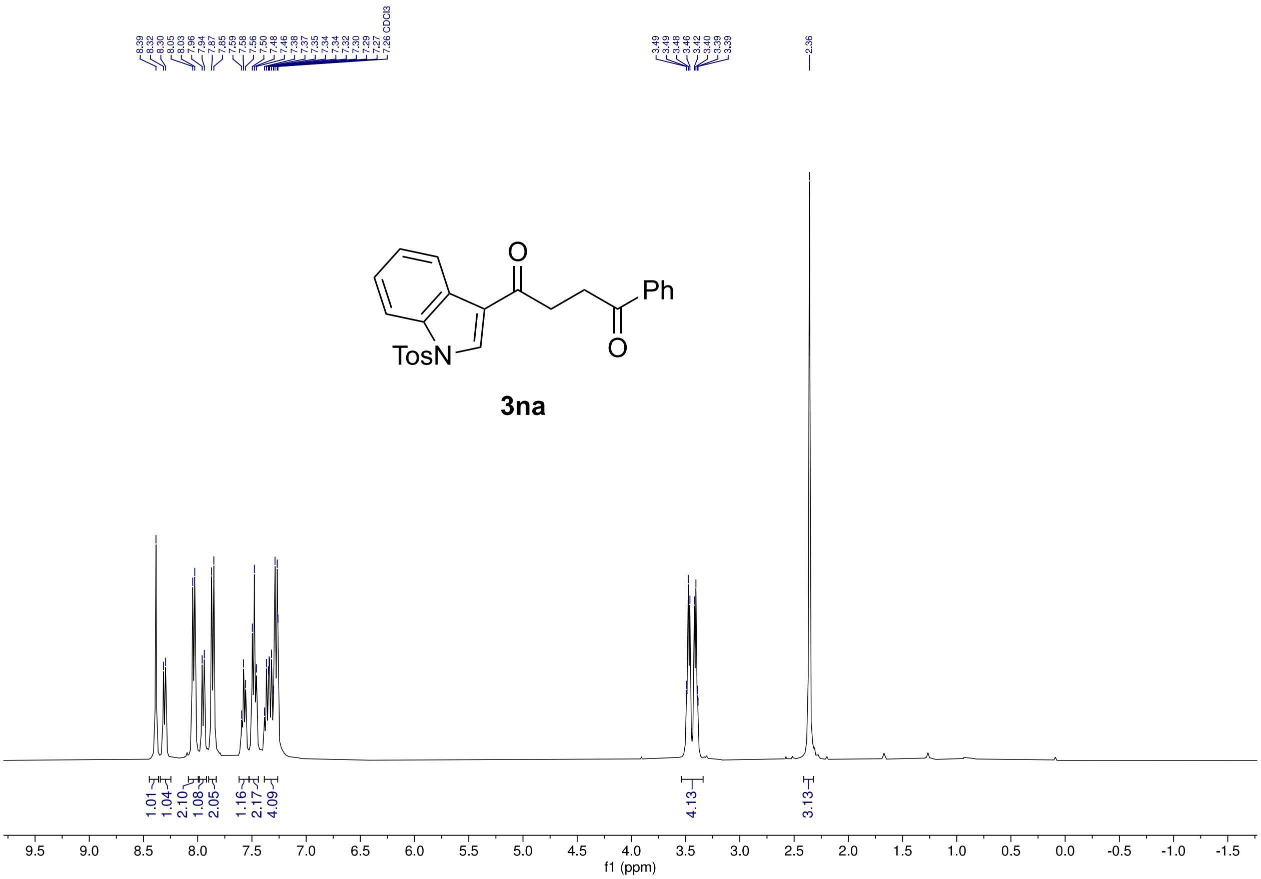

Figure S53. ^{13}C NMR of 1-phenyl-4-(1-tosyl-1*H*-indol-3-yl)butane-1,4-dione (**3na**) in $CDCl_3$ measured at 400 MHz.

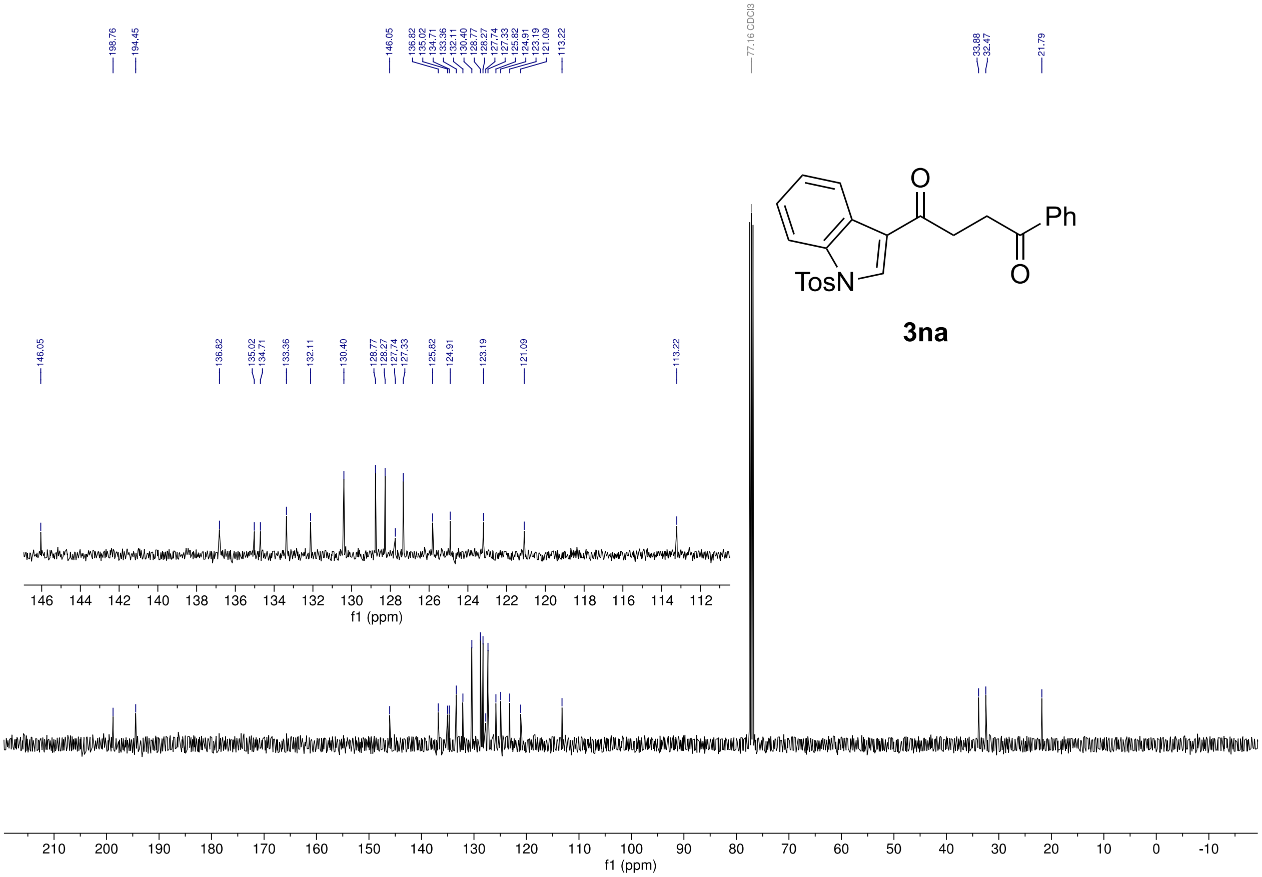

Figure S54. ^{13}C NMR of 1-phenyl-4-(1-tosyl-1*H*-indol-3-yl)butane-1,4-dione (**3na**) in $CDCl_3$ measured at 101 MHz.

2-(2-oxo-2-phenylethyl)-3,4-dihydronaphthalen-1(2*H*)-one

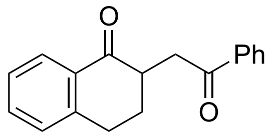

3oa

3oa was synthesized following the general procedure **4**, employing **1o** (160 mg, 1.00 mmol, 1.00 eq), **2a** (321 mg, 1.50 mmol, 1.50 eq), CuCl (149 mg, 1.50 mmol, 1.50 eq), K_2CO_3 (138 mg, 1.00 mmol, 1.00 eq) at 80°C, and isolated via flash chromatography (23 g SiO_2, gradient from 10:90 to 20:80 EtOAc/*n*-hexane over 10 CV, gradient from 20:80 to 50:50 EtOAc/*n*-hexane over 5 CV) as a light yellow solid (217 mg, 82%).

$C_{18}H_{16}O_2$ (264,32 g/mol)

mp: 90 °C

Rf: 0.30 (EtOAc/n-hexane = 1:10) [UV]

^{1}H NMR (400 MHz, $CDCl_3$): δ = 8.04 (dt, *J* = 8.4, 1.5 Hz, 3H), 7.61 – 7.54 (m, 1H), 7.48 (tt, *J* = 7.5, 1.4 Hz, 3H), 7.35 – 7.28 (m, 1H), 7.28 – 7.23 (m, 1H), 3.87 (dd, *J* = 17.5, 4.7 Hz, 1H), 3.33 (m, 1H), 3.25 – 3.13 (m, 1H), 3.04 – 2.93 (m, 2H), 2.30 (m, 1H), 1.99 (m, 1H).

^{13}C NMR (101 MHz, $CDCl_3$): δ = 199.0, 198.6, 144.2, 137.1, 133.4, 133.2, 132.4, 128.8, 128.7, 128.2, 127.5, 126.7, 44.3, 39.1, 29.6, 29.5.

HRMS (ESI) m/z: $[M+Na]^+$ Calcd for $C_{18}H_{16}O_2Na$ 287.1043; Found 287.1045.

IR (ATR, ṽ): 1675 (m, CO).

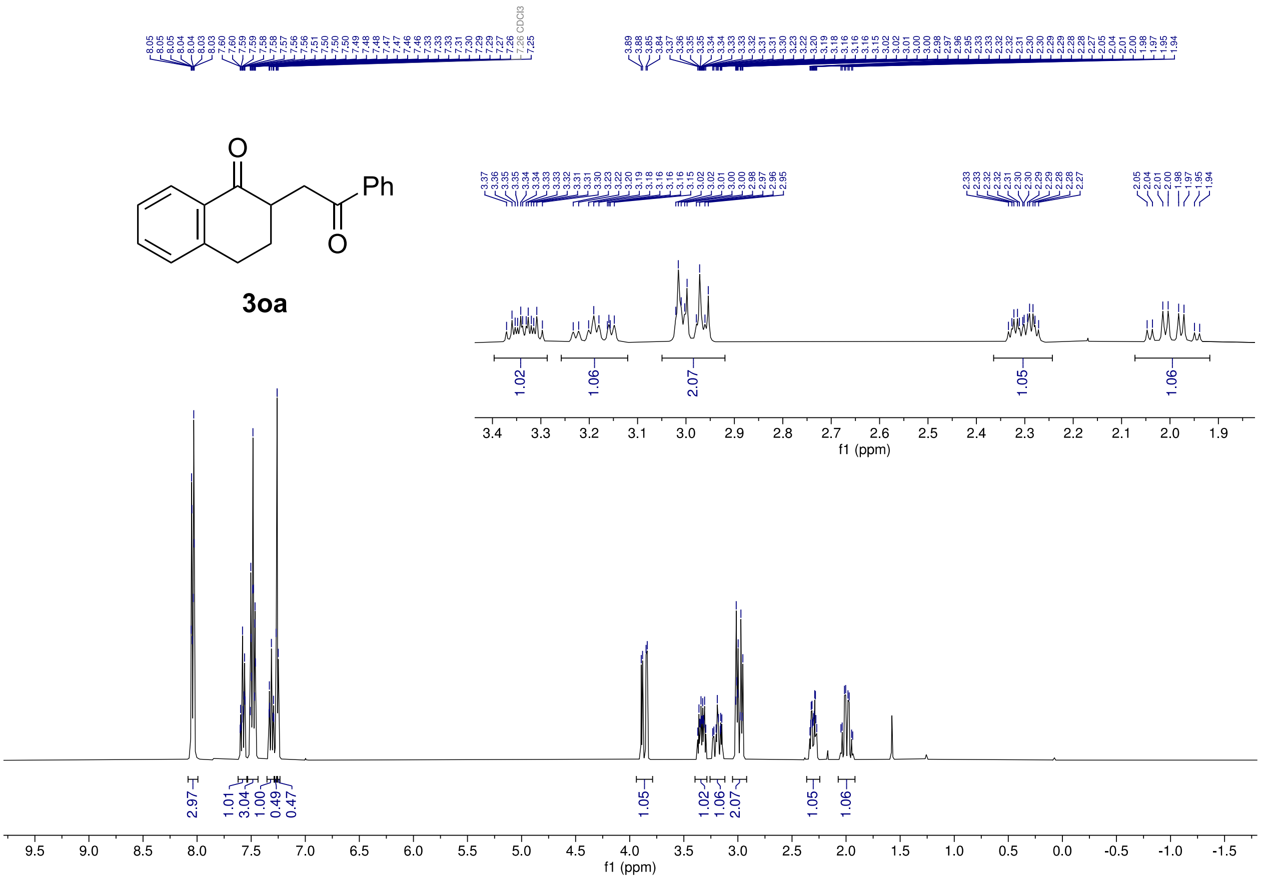

Figure S55. ^{1}H NMR of 2-(2-oxo-2-phenylethyl)-3,4-dihydronaphthalen-1(2*H*)-one (**3oa**) in $CDCl_3$ measured at 400 MHz.

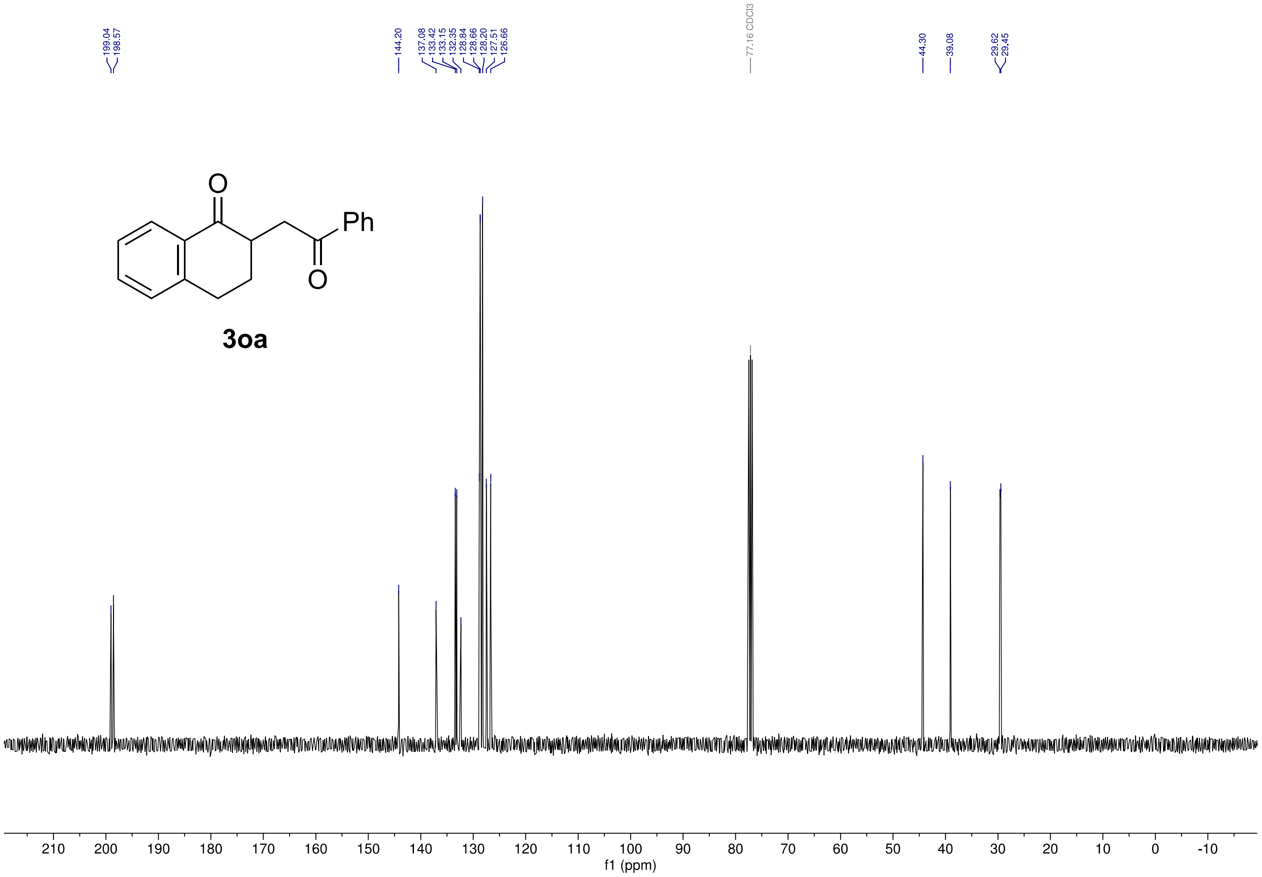

Figure S56. ^{13}C NMR of 2-(2-oxo-2-phenylethyl)-3,4-dihydronaphthalen-1(2*H*)-one (**3oa**) in $CDCl_3$ measured at 101 MHz.

3-(2-oxo-2-phenylethyl)chroman-4-one

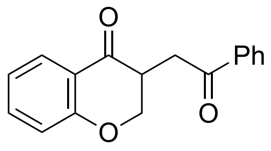

3pa

3pa was synthesized following the general procedure **4**, employing **1p** (162 mg, 1.00 mmol, 1.00 eq), **2a** (321 mg, 1.50 mmol, 1.50 eq), CuCl (149 mg, 1.50 mmol, 1.50 eq), K_2CO_3 (138 mg, 1.00 mmol, 1.00 eq) at 80°C, and isolated via flash chromatography (23 g SiO_2, gradient from 10:90 to 20:80 EtOAc/*n*-hexane over 10 CV, gradient from 20:80 to 50:50 EtOAc/*n*-hexane over 5 CV) as a pale yellow solid (128 mg, 48%).

$C_{17}H_{14}O_3$ (266,30 g/mol)

mp: 93-94°C

Rf: 0.29 (EtOAc/n-hexane = 1:10) [UV]

^{1}H NMR (400 MHz, $CDCl_3$): δ = 8.04 – 7.99 (m, 2H), 7.92 (dd, *J* = 7.8, 1.8 Hz, 1H), 7.62 – 7.56 (m, 1H), 7.49 (m, 3H), 7.07 – 6.97 (m, 2H), 4.66 (m, 1H), 4.32 (m, 1H), 3.72 (m, 1H), 3.65 – 3.56 (m, 1H), 3.03 (m, 1H).

^{13}C NMR (101 MHz, $CDCl_3$): δ = 197.1, 193.5, 161.9, 136.5, 136.1, 133.6, 128.8, 128.2, 127.5, 121.5, 120.8, 118.0, 70.5, 42.0, 34.4.

HRMS (ESI) m/z: $[M+Na]^+$ Calcd for $C_{17}H_{14}O_3Na$ 289.0835; Found 289.0838.

IR (ATR, ṽ): 1679 (m, CO).

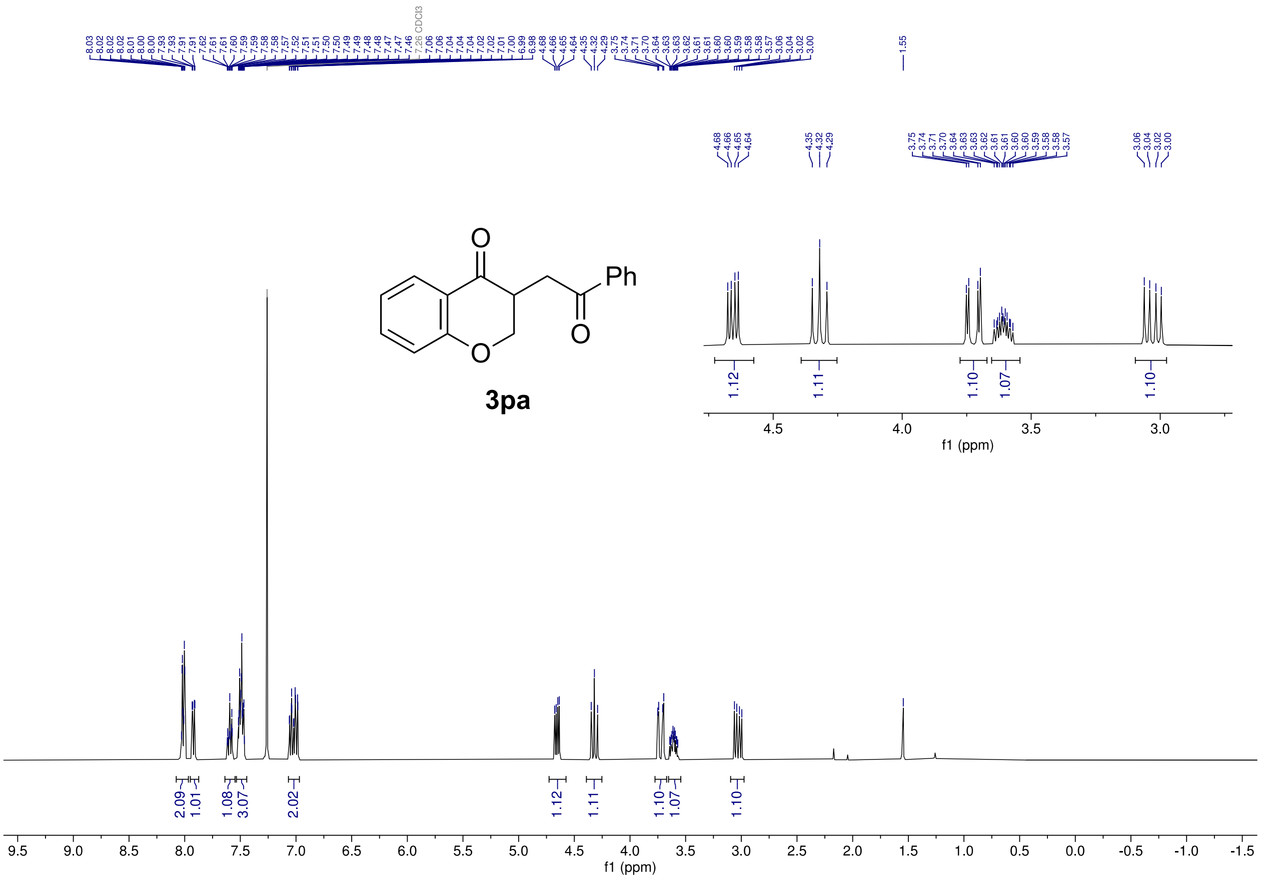

Figure S57. ^{1}H NMR of 3-(2-oxo-2-phenylethyl)chroman-4-one (**3pa**) in $CDCl_3$ measured at 400 MHz.

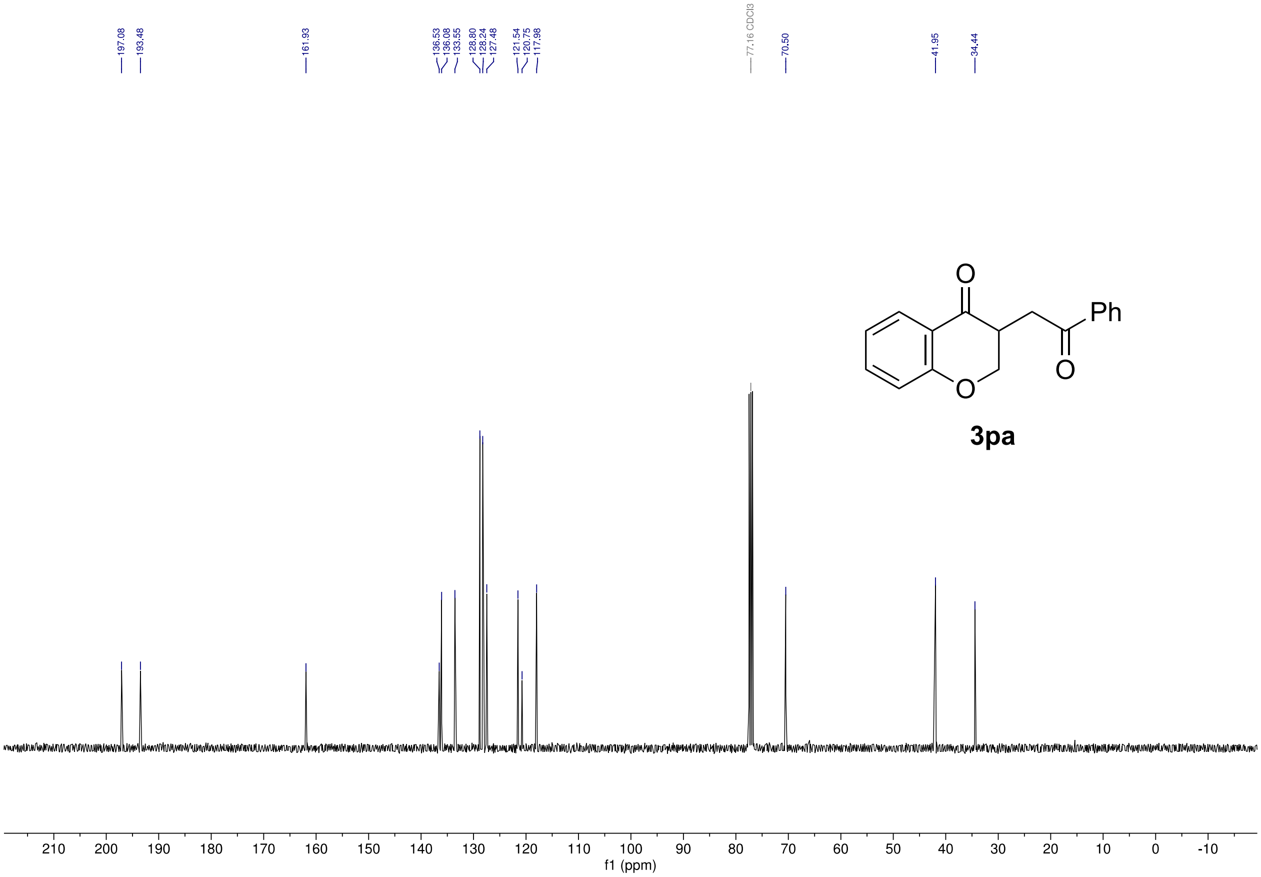

Figure S58. ^{13}C NMR of 3-(2-oxo-2-phenylethyl)chroman-4-one (**3pa**) in $CDCl_3$ measured at 101 MHz.

1-(cyclohex-1-en-1-yl)-4-phenylbutane-1,4-dione

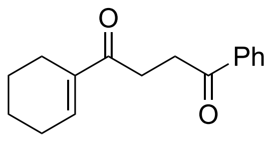

3qa

3qa was synthesized following the general procedure **4**, employing **1q** (138 mg, 1.00 mmol, 1.00 eq), **2a** (321 mg, 1.50 mmol, 1.50 eq), CuCl (149 mg, 1.50 mmol, 1.50 eq), K_2CO_3 (138 mg, 1.00 mmol, 1.00 eq) at 80°C, and isolated via flash chromatography (23 g SiO_2, gradient from 10:90 to 20:80 EtOAc/*n*-hexane over 10 CV, gradient from 20:80 to 50:50 EtOAc/*n*-hexane over 5 CV) as an off-white solid (194 mg, 80%).

$C_{16}H_{18}O_2$ (242,32 g/mol)

mp: 70-71°C

Rf: 0.33 (EtOAc/n-hexane = 1:10) [UV]

^{1}H NMR (400 MHz, $CDCl_3$): δ = 8.01 (dt, *J* = 6.8, 1.4 Hz, 2H), 7.59 – 7.52 (m, 1H), 7.46 (tt, *J* = 6.5, 1.4 Hz, 2H), 7.04 (m, 1H), 3.31 (t, *J* = 6.4 Hz, 2H), 3.12 (t, *J* = 6.4 Hz, 2H), 2.26 (m, 4H), 1.70 – 1.56 (m, 4H).

^{13}C NMR (101 MHz, $CDCl_3$): δ = 199.6, 199.2, 140.2, 139.1, 137.0, 133.1, 128.6, 128.2, 32.7, 31.1, 26.2, 23.3, 22.1, 21.7.

HRMS (ESI) m/z: $[M+Na]^+$ Calcd for $C_{16}H_{18}O_2Na$ 265.1199; Found 265.1199.

IR (ATR, ṽ): 1657 (s, CO).

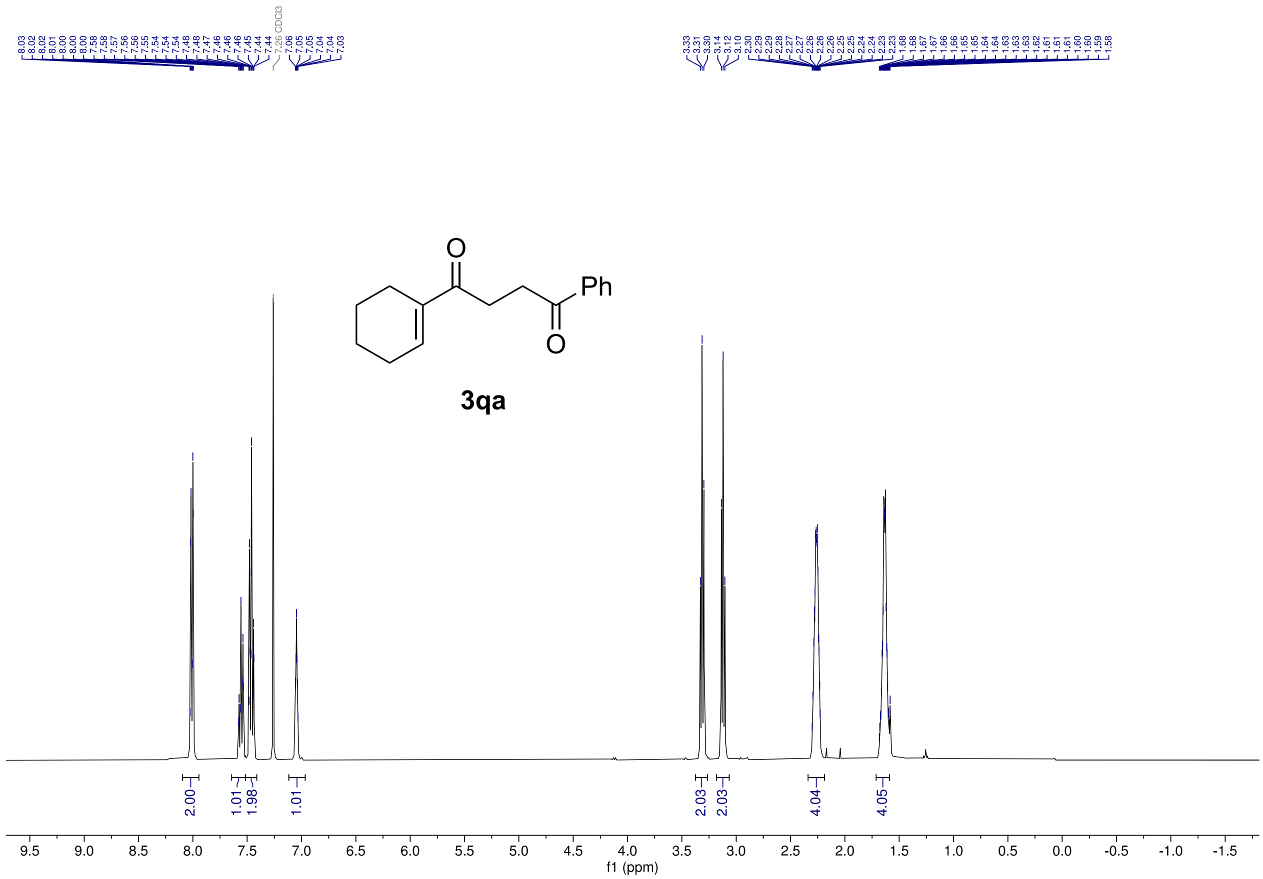

Figure S59. ^{1}H NMR of 1-(cyclohex-1-en-1-yl)-4-phenylbutane-1,4-dione (**3qa**) in $CDCl_3$ measured at 400 MHz.

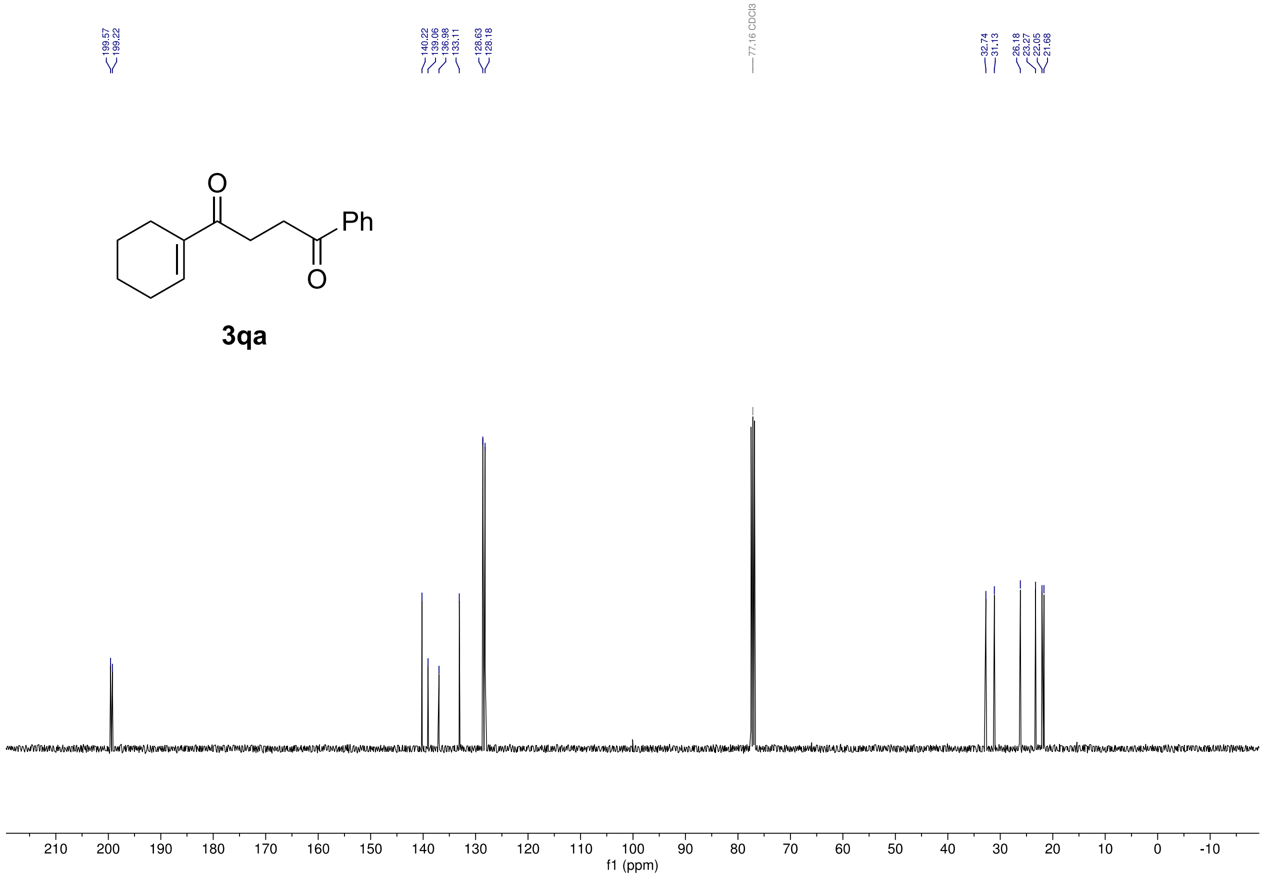

Figure S60. ^{13}C NMR of 1-(cyclohex-1-en-1-yl)-4-phenylbutane-1,4-dione (**3qa**) in $CDCl_3$ measured at 101 MHz.

2-cyclopropyl-1,4-diphenylbutane-1,4-dione

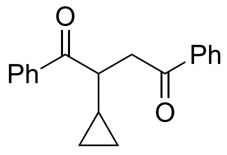

3ra

3ra was synthesized following the general procedure **4**, employing **1r** (174 mg, 1.00 mmol, 1.00 eq), **2a** (321 mg, 1.50 mmol, 1.50 eq), CuCl (149 mg, 1.50 mmol, 1.50 eq), K_2CO_3 (138 mg, 1.00 mmol, 1.00 eq) at 80°C, and isolated via flash chromatography (23 g SiO_2, gradient from 10:90 to 20:80 EtOAc/*n*-hexane over 10 CV, gradient from 20:80 to 50:50 EtOAc/*n*-hexane over 5 CV) as a colourless solid (239 mg, 86%).

$C_{19}H_{18}O_2$ (278,35 g/mol)

mp: 83-85°C

Rf: 0.36 (EtOAc/n-hexane = 1:10) [UV]

^{1}H NMR (400 MHz, $CDCl_3$): δ = 8.07 – 8.02 (m, 2H), 7.99 (dt, *J* = 7.0, 1.4 Hz, 2H), 7.61 – 7.53 (m, 2H), 7.53 – 7.41 (m, 4H), 3.92 (dd, *J* = 18.0, 9.8 Hz, 1H), 3.46 (td, *J* = 9.7, 3.7 Hz, 1H), 3.32 (dd, *J* = 18.0, 3.7 Hz, 1H), 0.94 (m, 1H), 0.61 – 0.53 (m, 1H), 0.50 – 0.41 (m, 1H), 0.33 – 0.19 (m, 2H).

^{13}C NMR (101 MHz, $CDCl_3$): δ = 202.7, 198.7, 137.7, 136.5, 133.2, 132.8, 128.6, 128.5, 128.1, 45.2, 42.0, 13.9, 4.7, 4.2.

HRMS (ESI) m/z: $[M+Na]^+$ Calcd for $C_{19}H_{18}O_2Na$ 301.1199; Found 301.1202.

IR (ATR, ṽ): 1746 (w, CO), 1671 (s, CO).

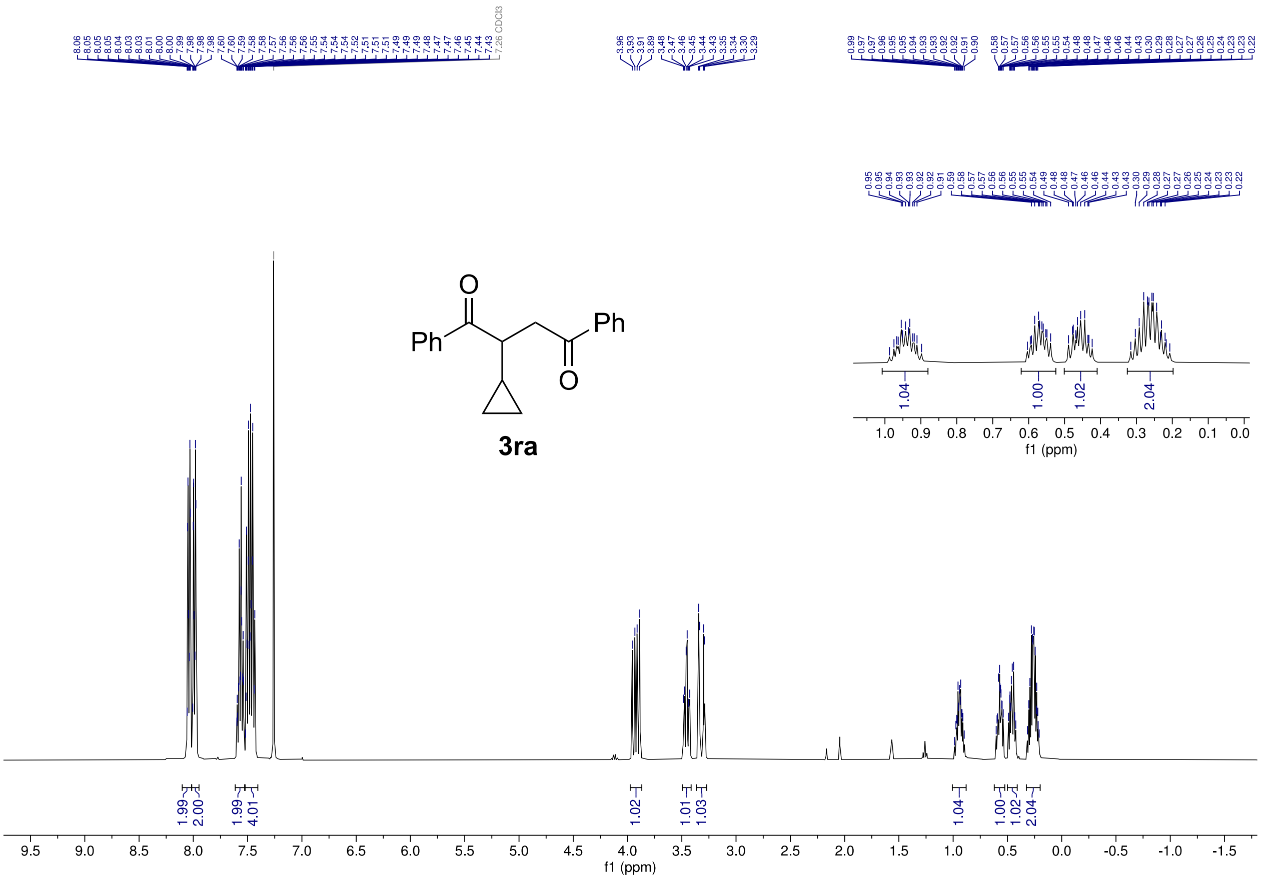

Figure S61. ^{1}H NMR of 2-cyclopropyl-1,4-diphenylbutane-1,4-dione (**3ra**) in $CDCl_3$ measured at 400 MHz.

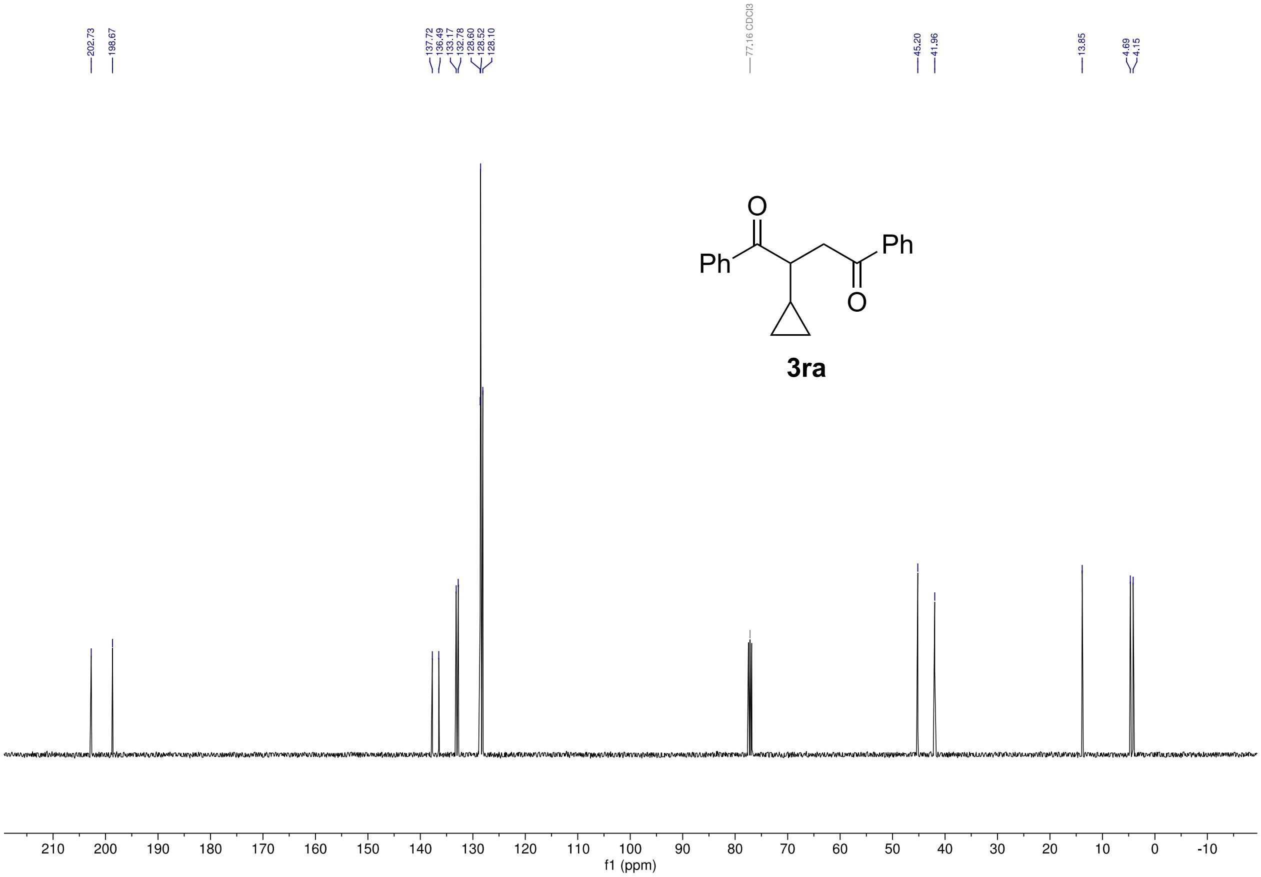

Figure S62. ^{13}C NMR of 2-cyclopropyl-1,4-diphenylbutane-1,4-dione (**3ra**) in $CDCl_3$ measured at 101 MHz.

4,4-difluoro-2-(2-oxo-2-phenylethyl)cyclohexan-1-one

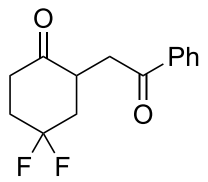

3sa

3sa was synthesized following the general procedure **4**, employing **1b** (148 mg, 1.00 mmol, 1.00 eq), **2a** (321 mg, 1.50 mmol, 1.50 eq), CuCl (149 mg, 1.50 mmol, 1.50 eq), K_2CO_3 (138 mg, 1.00 mmol, 1.00 eq) at 90°C, and isolated via flash chromatography (23 g SiO_2, gradient from 10:90 to 20:80 EtOAc/*n*-hexane over 10 CV, gradient from 20:80 to 50:50 EtOAc/*n*-hexane over 5 CV) as a yellowish solid (148 mg, 59%).

$C_{14}H_{14}F_2O_2$ (252,26 g/mol)

mp: 84-85°C

Rf: 0.16 (EtOAc/n-hexane = 1:10) [UV]

^{1}H NMR (400 MHz, $CDCl_3$): δ = 8.01 – 7.93 (m, 2H), 7.62 – 7.54 (m, 1H), 7.47 (m, 2H), 3.52 (dd, *J* = 17.7, 6.2 Hz, 1H), 3.46 – 3.34 (m, 1H), 2.92 (dd, *J* = 17.9, 5.1 Hz, 1H), 2.85 – 2.70 (m, 1H), 2.65 – 2.42 (m, 3H), 2.35 – 2.03 (m, 2H).

^{13}C NMR (101 MHz, $CDCl_3$): δ = 207.4, 197.2, 136.7, 133.4, 128.7, 128.1, 121.6 (dd, *J* = 243.8, 240.2 Hz), 41.2 (d, *J* = 10.2 Hz), 38.8 (t, *J* = 25.6 Hz), 37.6, 36.4 (d, *J* = 10.1 Hz), 33.3 (t, *J* = 26.1 Hz).

^{19}F NMR (376 MHz, $CDCl_3$): δ = -95.51 – -96.67 (m, 1F), -103.16 (dtt, *J* = 243.2, 32.6, 10.1 Hz, 1F).

HRMS (ESI) m/z: $[M+Na]^+$ Calcd for $C_{14}H_{14}F_2O_2Na$ 275.0854; Found 275.0855.

IR (ATR, ṽ): 1720 (m, CO), 1676 (s, CO).

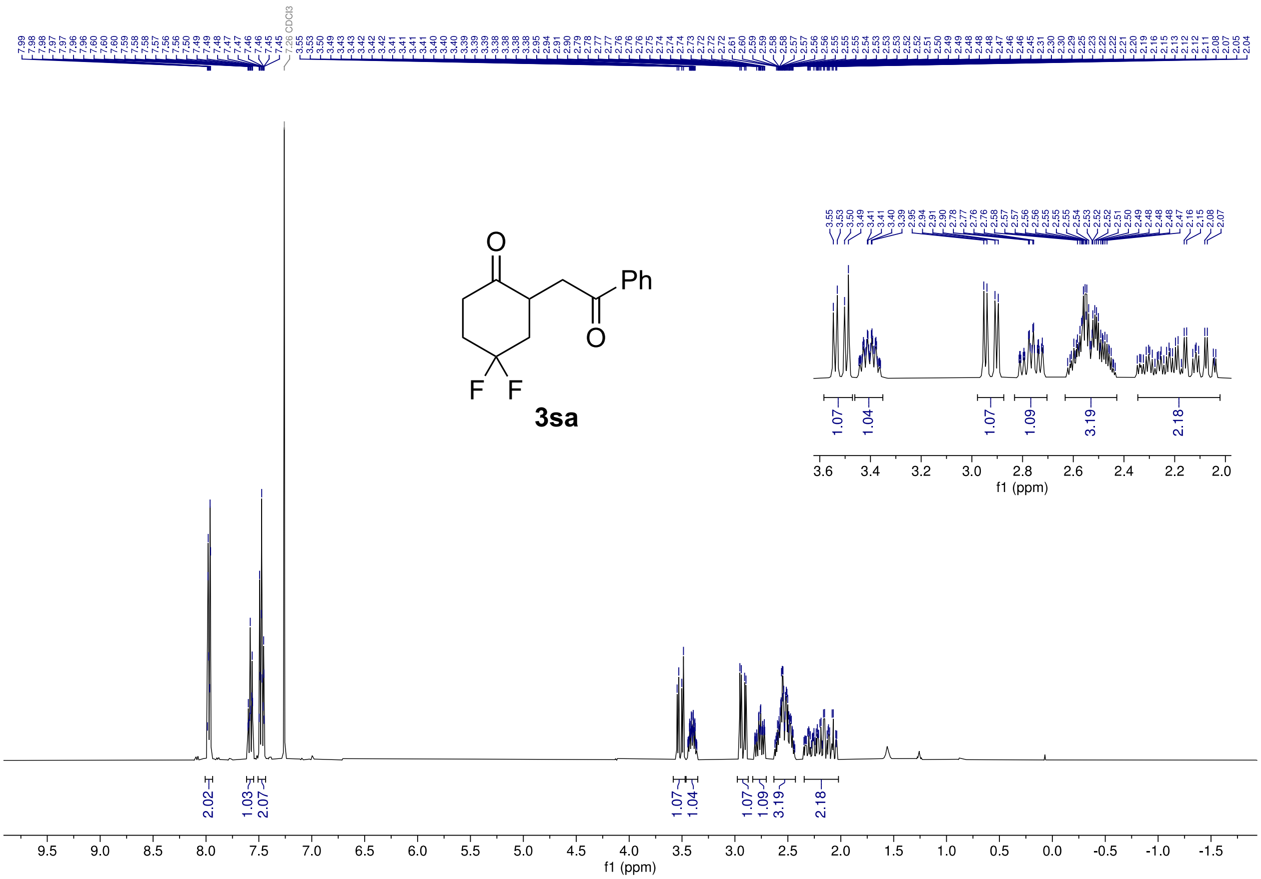

Figure S63. ^{1}H NMR of 4,4-difluoro-2-(2-oxo-2-phenylethyl)cyclohexan-1-one (**3sa**) in $CDCl_3$ measured at 400 MHz.

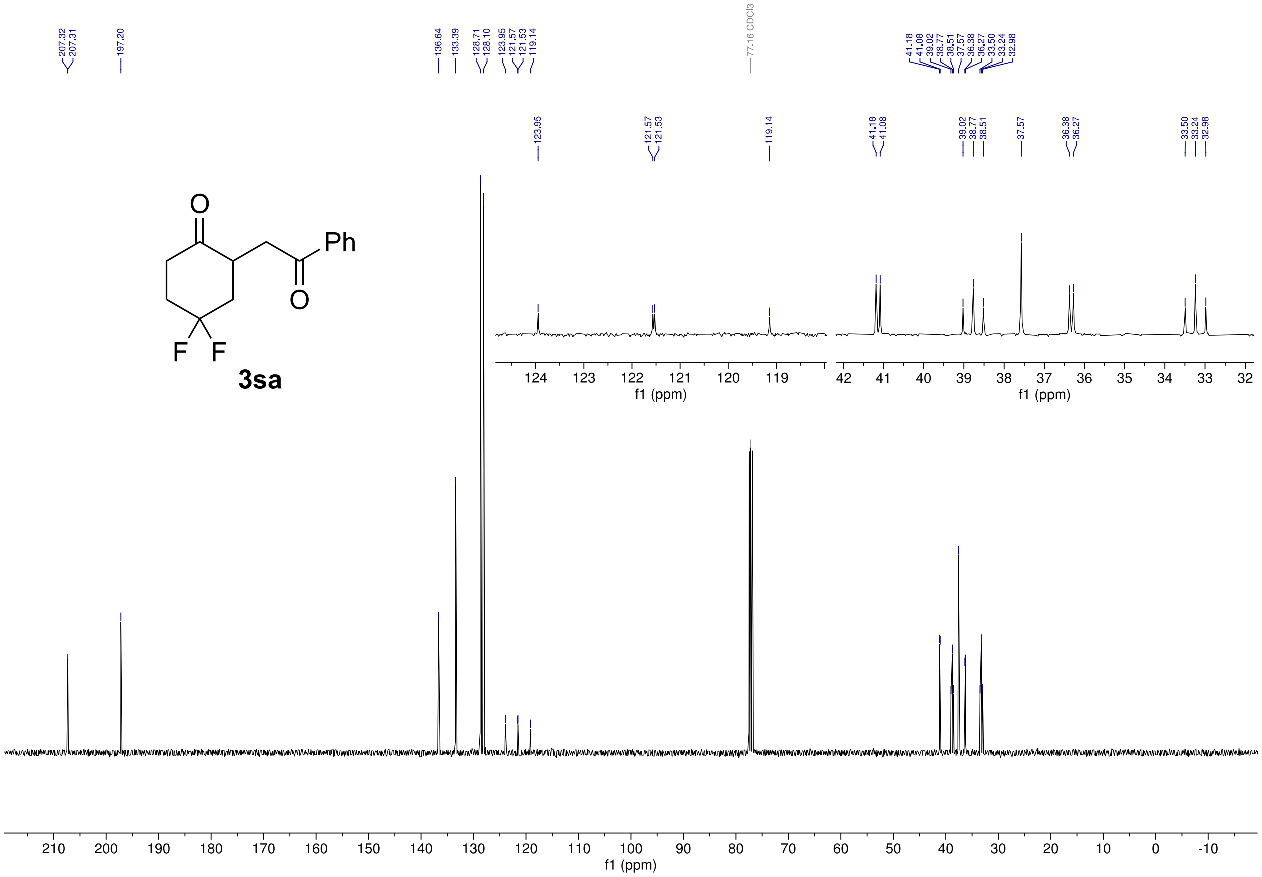

Figure S64. ^{13}C NMR of 4,4-difluoro-2-(2-oxo-2-phenylethyl)cyclohexan-1-one (**3sa**) in $CDCl_3$ measured at 101 MHz.

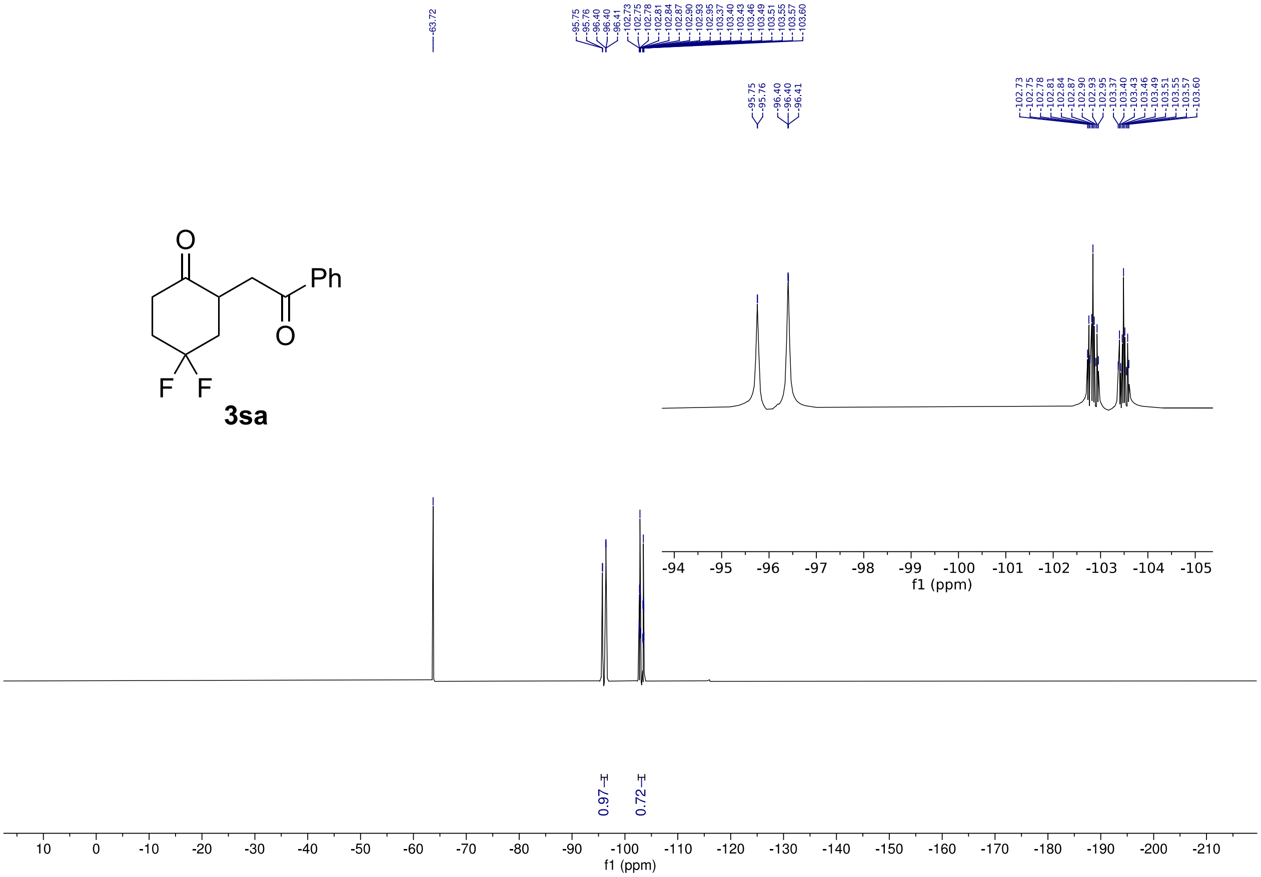

Figure S65. ^{19}F NMR of 4,4-difluoro-2-(2-oxo-2-phenylethyl)cyclohexan-1-one (**3sa**) in $CDCl_3$ measured at 376 MHz.

methyl 4-(4-(4-chlorophenyl)-4-oxobutanoyl)benzoate

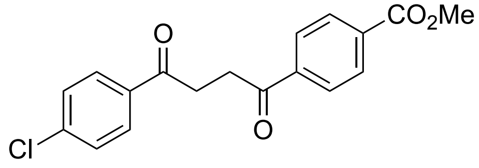

3ab

3ab was synthesized following the general procedure **4**, employing **1a** (169 mg, 1.00 mmol, 1.00 eq), **2b** (**409** mg, 1.50 mmol, 1.50 eq), CuCl (149 mg, 1.50 mmol, 1.50 eq), K_2CO_3 (138 mg, 1.00 mmol, 1.00 eq) at 80°C, and isolated via flash chromatography (23 g SiO_2, gradient from 10:90 to 20:80 EtOAc/*n*-hexane over 10 CV, gradient from 20:80 to 50:50 EtOAc/*n*-hexane over 5 CV) as a pale yellow solid (296 mg, 89%).

$C_{18}H_{15}ClO_4$ (330,76 g/mol)

mp: 174-175°C

Rf: 0.12 (EtOAc/n-hexane = 1:10) [UV]

^{1}H NMR (400 MHz, $CDCl_3$): δ = 8.19 – 8.11 (m, 2H), 8.11 – 8.05 (m, 2H), 8.01 – 7.94 (m, 2H), 7.50 – 7.41 (m, 2H), 3.96 (s, 3H), 3.45 (m, 4H).

^{13}C NMR (101 MHz, $CDCl_3$): δ = 198.2, 197.4, 166.4, 140.0, 139.8, 135.1, 134.1, 130.0, 129.7, 129.1, 128.2, 52.6, 33.0, 32.6.

HRMS (ESI) m/z: $[M+Na]^+$ Calcd for $C_{18}H_{15}ClO_4Na$ 353.0551; Found 353.0553.

IR (ATR, ṽ): 1720 (m, CO), 1668 (m, CO).

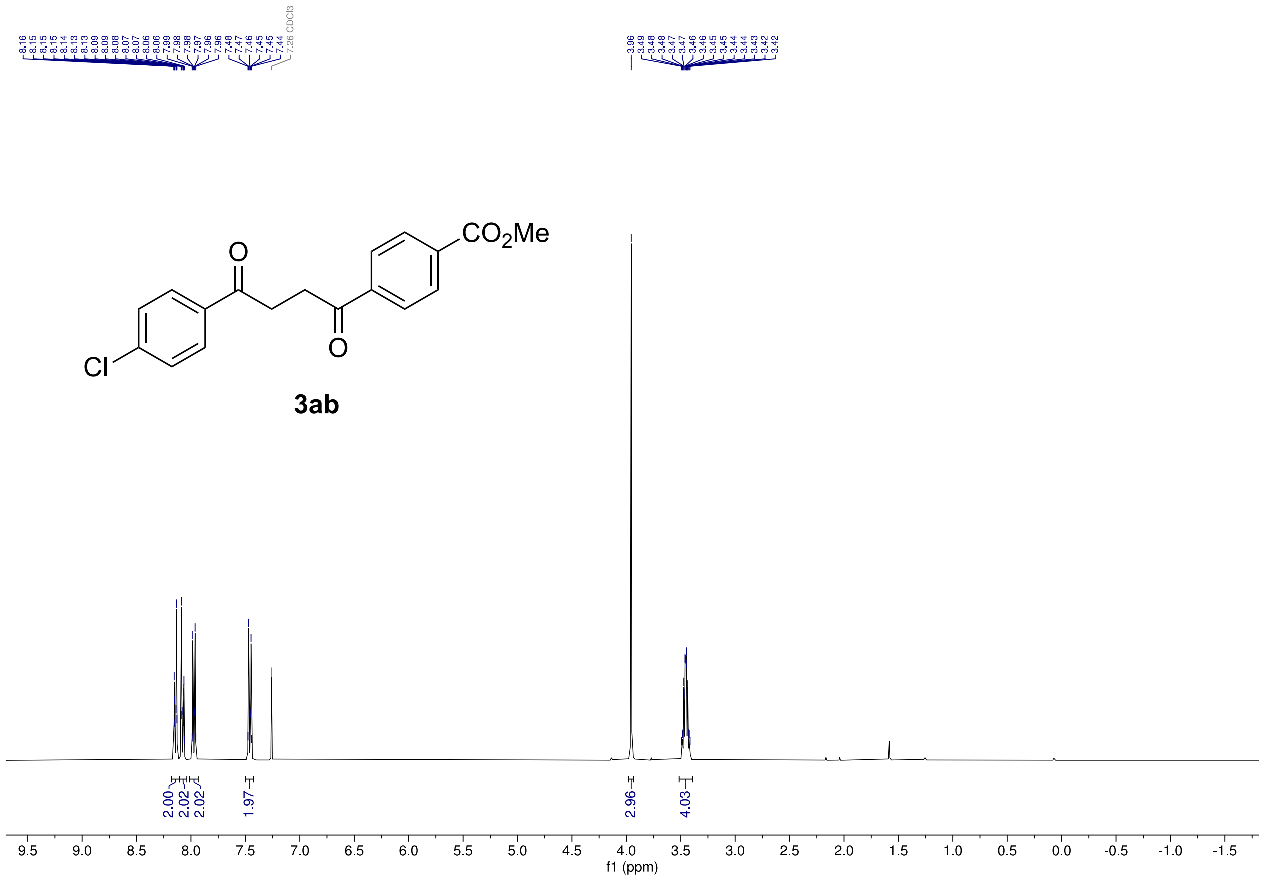

Figure S66. ^{1}H NMR of methyl 4-(4-(4-chlorophenyl)-4-oxobutanoyl)benzoate (**3ab**) in $CDCl_3$ measured at 400 MHz.

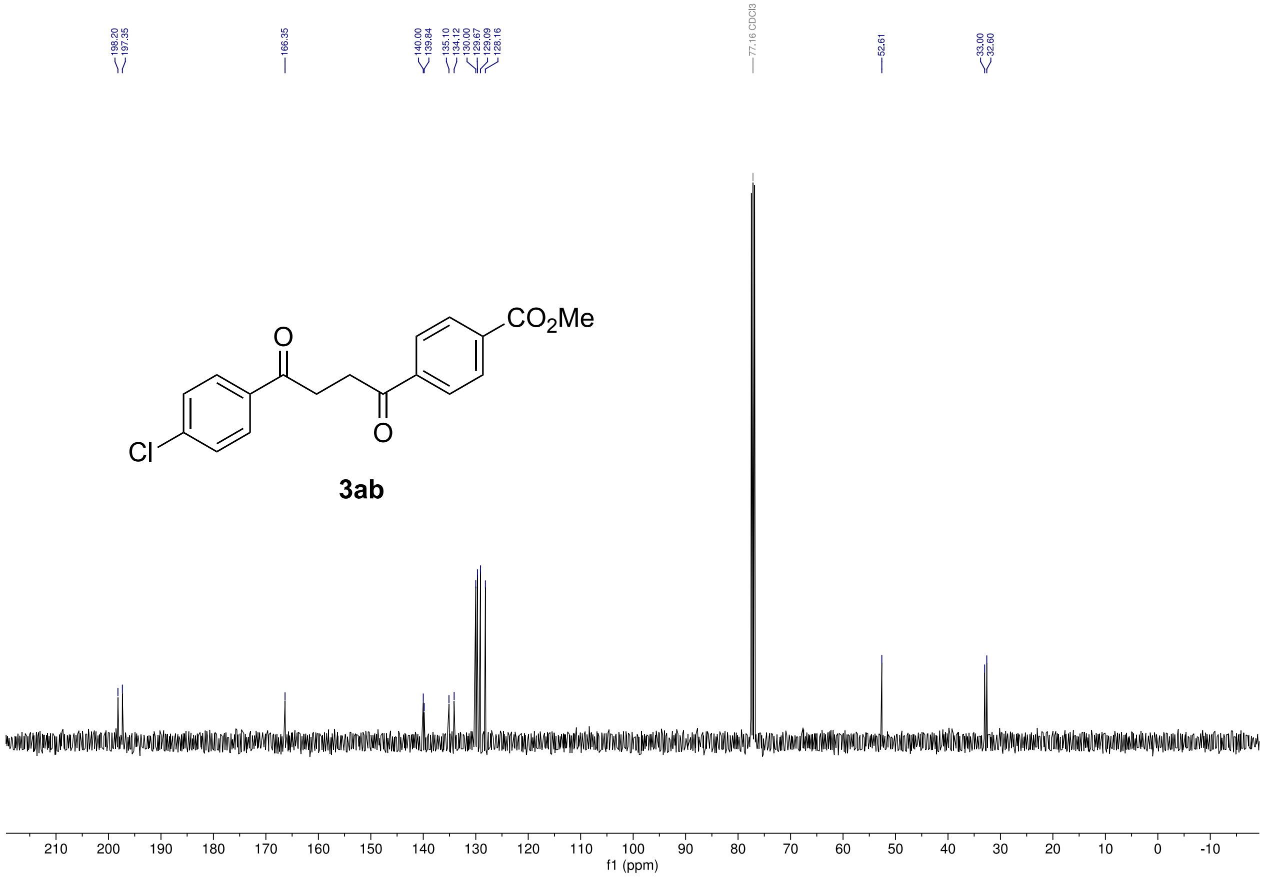

Figure S67. ^{13}C NMR of methyl 4-(4-(4-chlorophenyl)-4-oxobutanoyl)benzoate (**3ab**) in $CDCl_3$ measured at 101 MHz.

4-(4-(4-chlorophenyl)-4-oxobutanoyl)benzonitrile

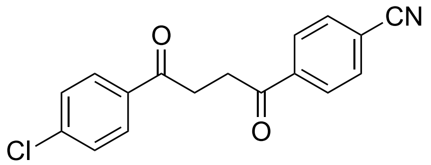

3ac

3ac was synthesized following the general procedure **4**, employing **1a** (169 mg, 1.00 mmol, 1.00 eq), **2c** (**359** mg, 1.50 mmol, 1.50 eq), CuCl (149 mg, 1.50 mmol, 1.50 eq), K_2CO_3 (138 mg, 1.00 mmol, 1.00 eq) at 80 °C and isolated via flash chromatography (23 g SiO_2, gradient from 10:90 to 20:80 EtOAc/*n*-hexane over 10 CV, gradient from 20:80 to 50:50 EtOAc/*n*-hexane over 5 CV) as an ochre solid (235 mg, 79%).

$C_{17}H_{12}ClNO_2$ (297,74 g/mol)

mp: 169-170°C

Rf: 0.06 (EtOAc/n-hexane = 1:10) [UV]

^{1}H NMR (400 MHz, $CDCl_3$): δ = 8.15 – 8.09 (m, 2H), 7.99 – 7.93 (m, 2H), 7.82 – 7.77 (m, 2H), 7.49 – 7.44 (m, 2H), 3.44 (m, 4H).

^{13}C NMR (101 MHz, $CDCl_3$): δ = 197.4, 197.1, 139.9, 139.7, 134.9, 132.6, 129.6, 129.1, 128.6, 118.0, 116.5, 32.8, 32.6.

HRMS (ESI) m/z: $[M+Na]^+$ Calcd for $C_{17}H_{12}ClNO_2Na$ 320.0449; Found 320.0450.

IR (ATR, ṽ): 1676 (s, CO).

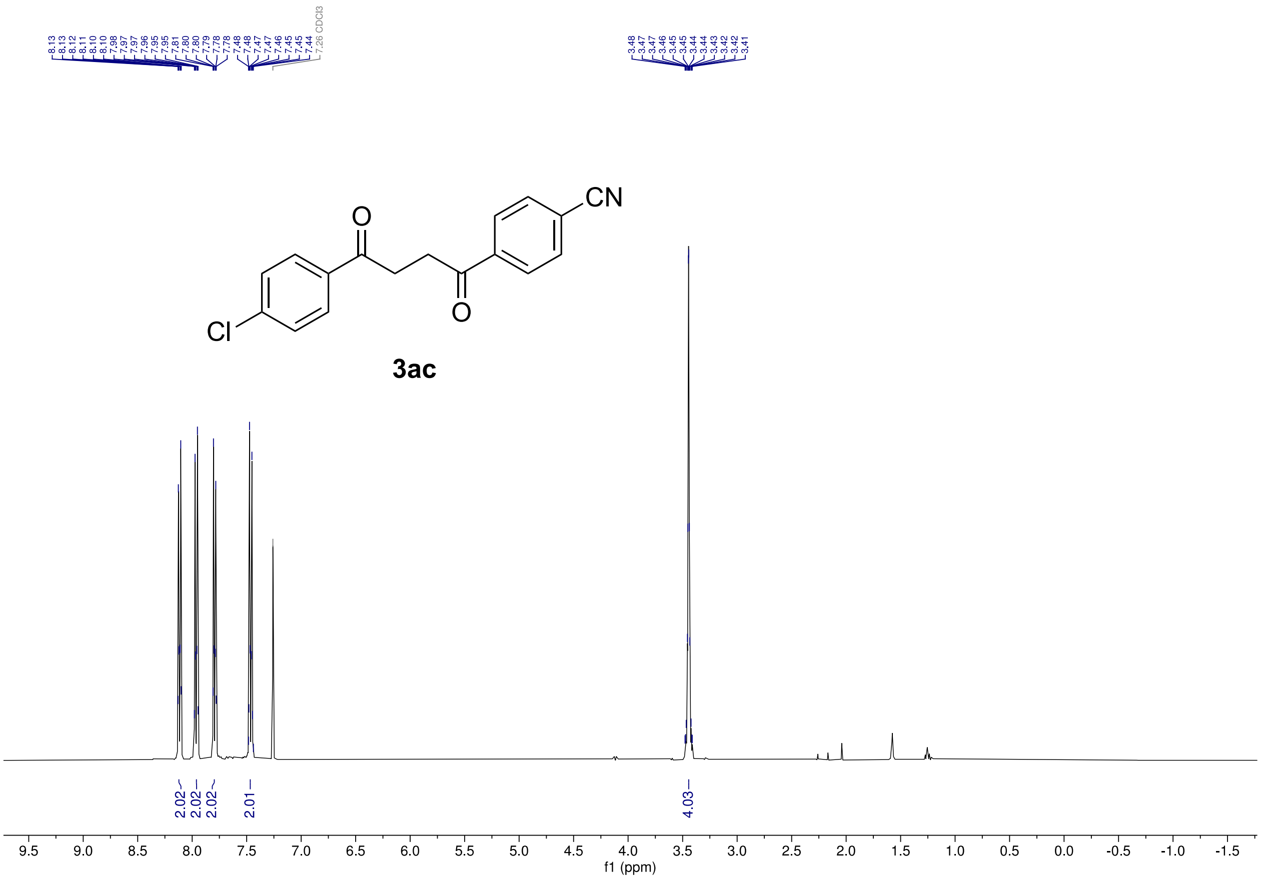

Figure S68. ^{1}H NMR of 4-(4-(4-chlorophenyl)-4-oxobutanoyl)benzonitrile (**3ac**) in $CDCl_3$ measured at 400 MHz.

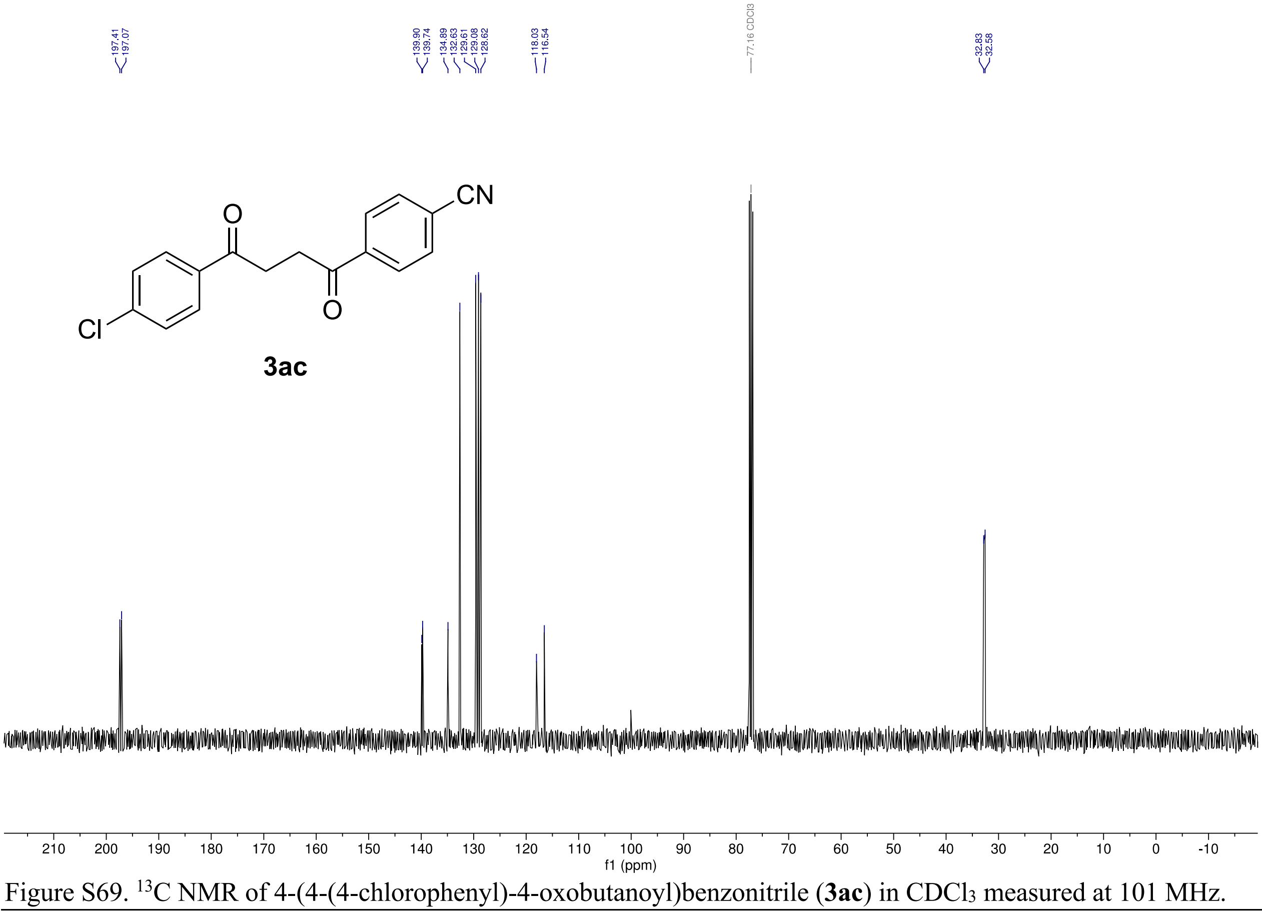

Figure S69. ^{13}C NMR of 4-(4-(4-chlorophenyl)-4-oxobutanoyl)benzonitrile (**3ac**) in $CDCl_3$ measured at 101 MHz.

1-(4-chlorophenyl)-4-(4-(trifluoromethyl)phenyl)butane-1,4-dione

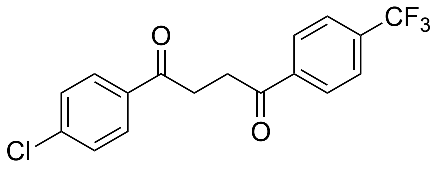

3ad

3ad was synthesized following the general procedure **4**, employing **1a** (169 mg, 1.00 mmol, 1.00 eq), **2d** (**423** mg, 1.50 mmol, 1.50 eq), CuCl (149 mg, 1.50 mmol, 1.50 eq), K_2CO_3 (138 mg, 1.00 mmol, 1.00 eq) at 80 °C and isolated via flash chromatography (23 g SiO_2, gradient from 10:90 to 20:80 EtOAc/*n*-hexane over 10 CV, gradient from 20:80 to 50:50 EtOAc/*n*-hexane over 5 CV) as a light yellow solid (263 mg, 77%).

$C_{17}H_{12}ClF_3O_2$ (340,73 g/mol)

mp: 121-122°C

Rf: 0.24 (EtOAc/n-hexane = 1:10) [UV]

^{1}H NMR (400 MHz, $CDCl_3$): δ = 8.14 (d, *J* = 8.1 Hz, 2H), 8.03 – 7.92 (m, 2H), 7.76 (d, *J* = 8.4 Hz, 2H), 7.53 – 7.40 (m, 2H), 3.45 (q, *J* = 1.6 Hz, 4H).

^{13}C NMR (101 MHz, $CDCl_3$): δ = 198.4, 197.9, 139.6, 136.7, 134.5 (q, *J* = 32.6 Hz), 133.4, 128.8, 128.6, 128.2, 125.8 (q, *J* = 3.7 Hz), 123.8 (q, *J* = 272.7 Hz), 32.9, 32.7.

^{19}F NMR (376 MHz, $CDCl_3$): δ = -63.33 (s, 3F).

HRMS (ESI) m/z: $[M+Na]^+$ Calcd for $C_{17}H_{12}ClF_3O_2Na$ 363.0370; Found 363.0376.

IR (ATR, ṽ): 1671 (s, CO).

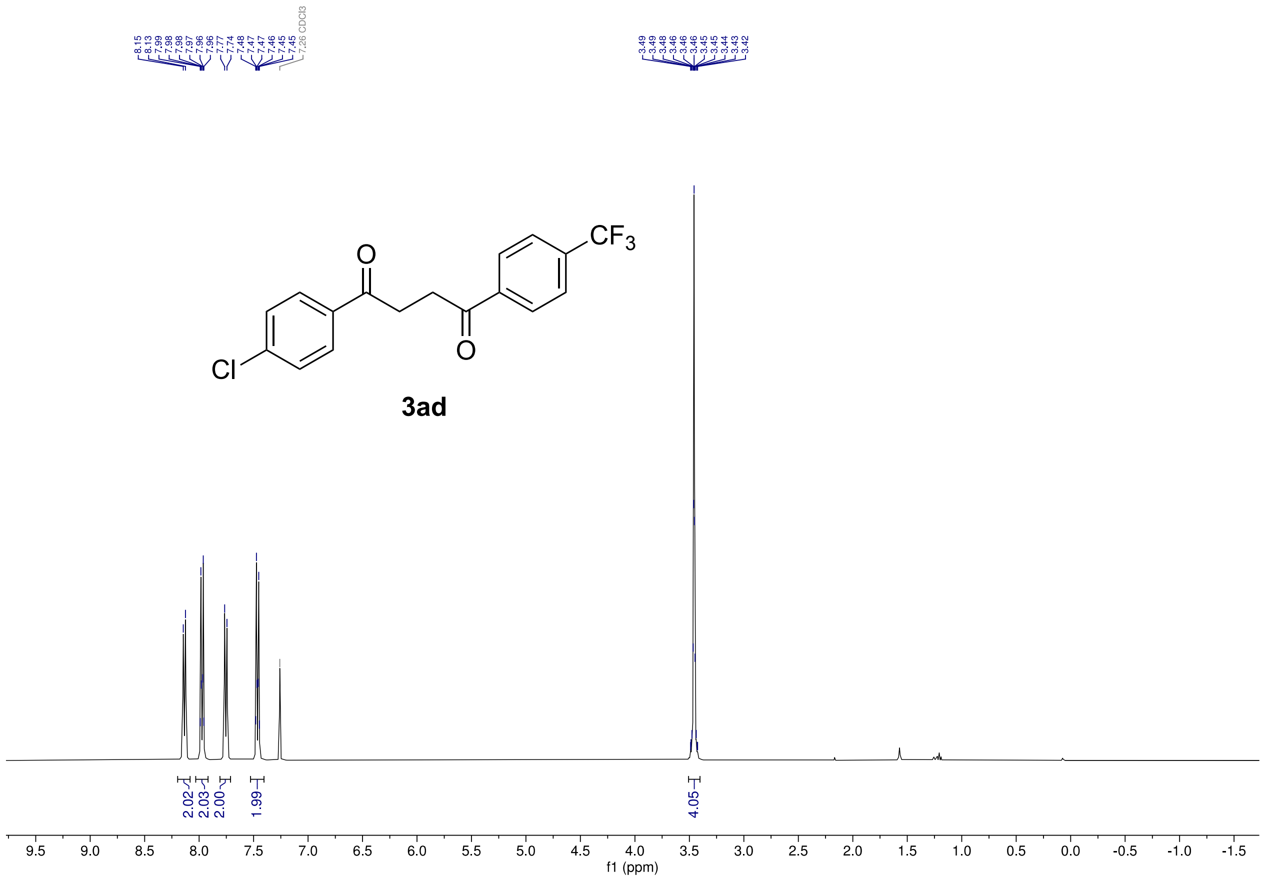

Figure S70. ^{1}H NMR of 1-(4-chlorophenyl)-4-(4-(trifluoromethyl)phenyl)butane-1,4-dione (**3ad**) in $CDCl_3$ measured at 400 MHz.

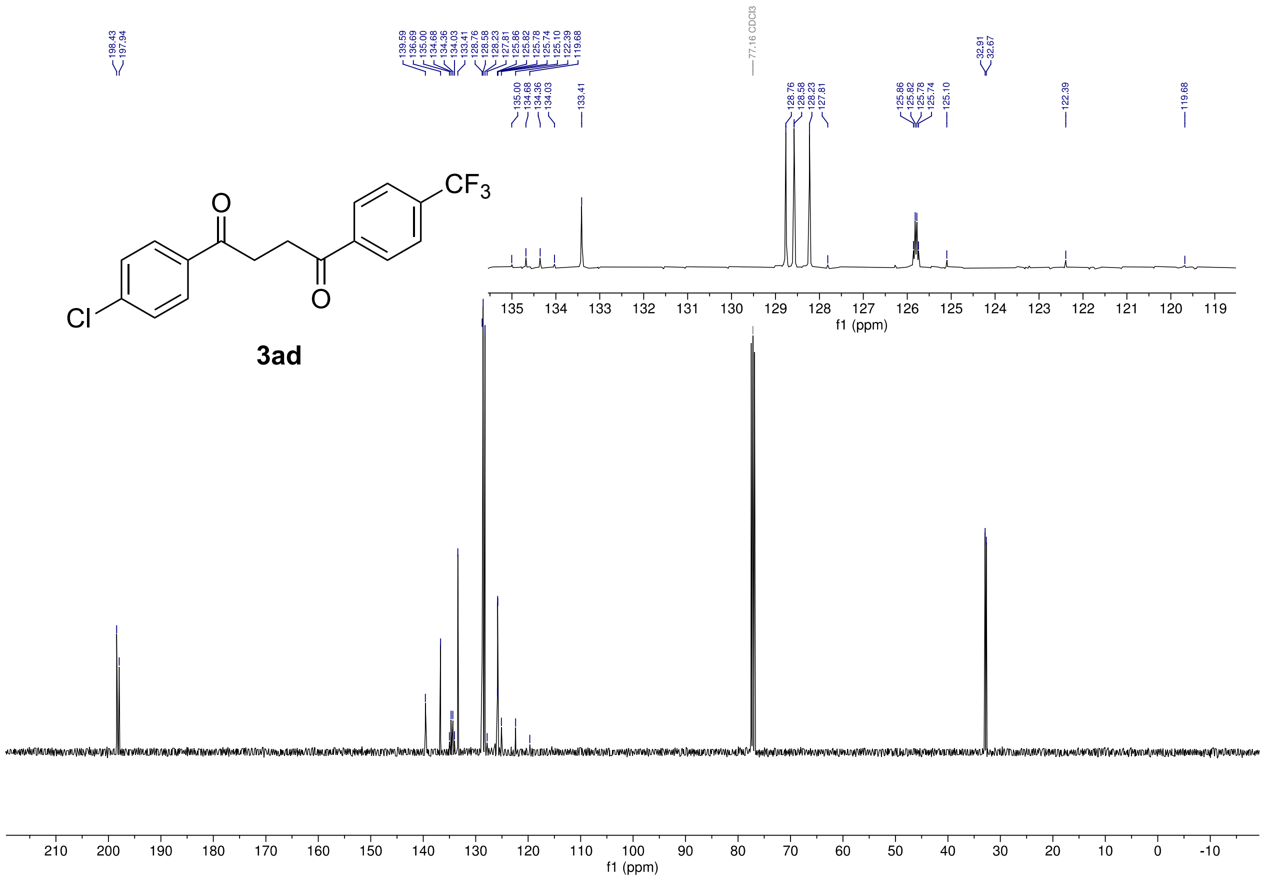

Figure S71. ^{13}C NMR of 1-(4-chlorophenyl)-4-(4-(trifluoromethyl)phenyl)butane-1,4-dione (**3ad**) in $CDCl_3$ measured at 101 MHz.

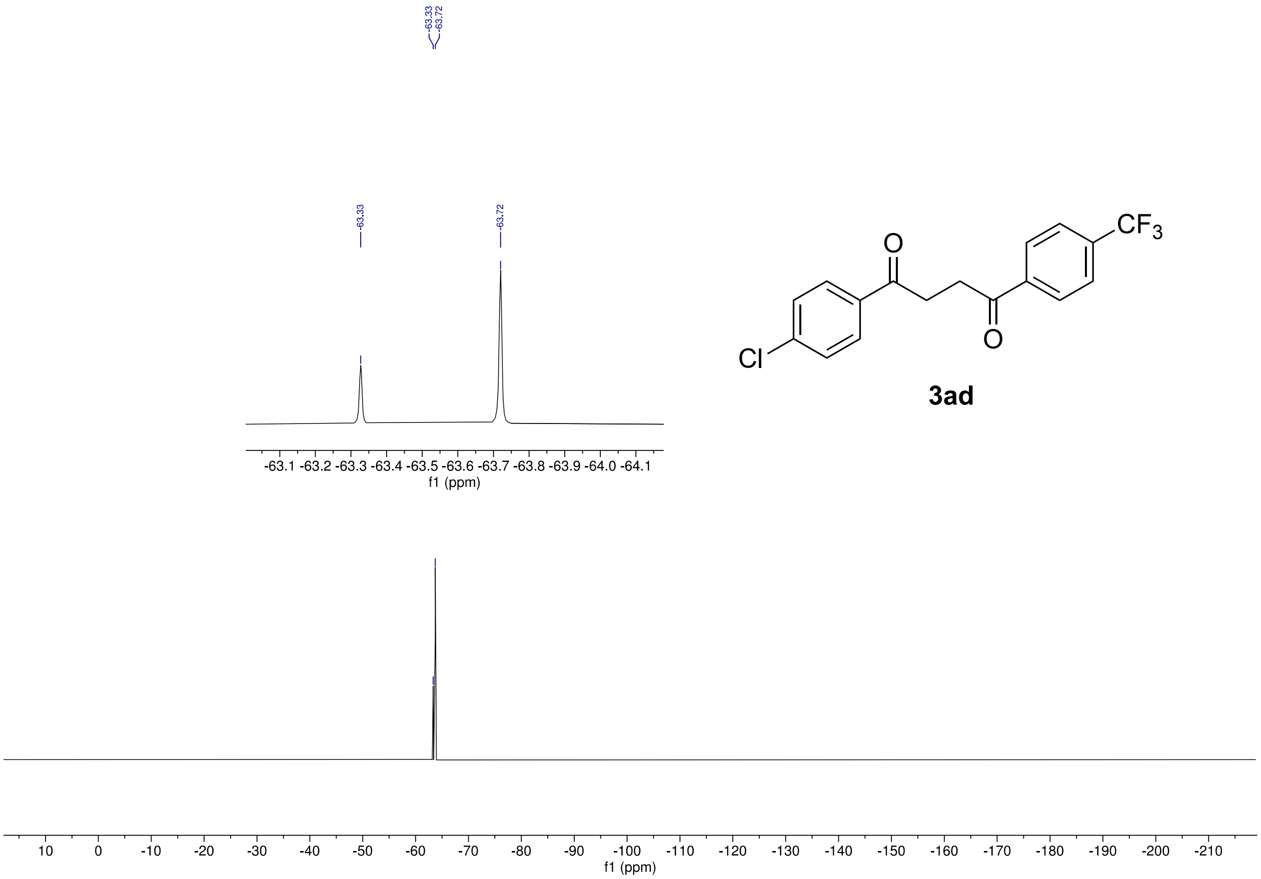

3ad

Figure S72. ^{19}F NMR of 1-(4-chlorophenyl)-4-(4-(trifluoromethyl)phenyl)butane-1,4-dione (**3ad**) in $CDCl_3$ measured at 376 MHz.

1,4-bis(4-chlorophenyl)butane-1,4-dione

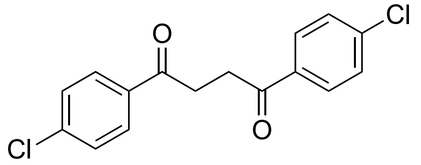

3ae

3ae was synthesized following the general procedure **4**, employing **1a** (169 mg, 1.00 mmol, 1.00 eq), **2e** (**373** mg, 1.50 mmol, 1.50 eq), CuCl (149 mg, 1.50 mmol, 1.50 eq), K_2CO_3 (138 mg, 1.00 mmol, 1.00 eq) at 80 °C and isolated via flash chromatography (23 g SiO_2, gradient from 10:90 to 20:80 EtOAc/*n*-hexane over 10 CV, gradient from 20:80 to 50:50 EtOAc/*n*-hexane over 5 CV) as a yellowish solid (272 mg, 88%).

$C_{16}H_{12}Cl_2O_2$ (307,17 g/mol)

mp: 148-149°C

Rf: 0.24 (EtOAc/n-hexane = 1:10) [UV]

^{1}H NMR (400 MHz, $CDCl_3$): δ = 8.01 – 7.93 (m, 4H), 7.49 – 7.42 (m, 4H), 3.42 (s, 4H).

^{13}C NMR (101 MHz, $CDCl_3$): δ = 197.4, 139.8, 135.1, 129.6, 129.1, 32.6.

HRMS (ESI) m/z: $[M+Na]^+$ Calcd for $C_{16}H_{12}Cl_2O_2Na$ 329.0107; Found 329.0104.

IR (ATR, ṽ): 1668 (s, CO).

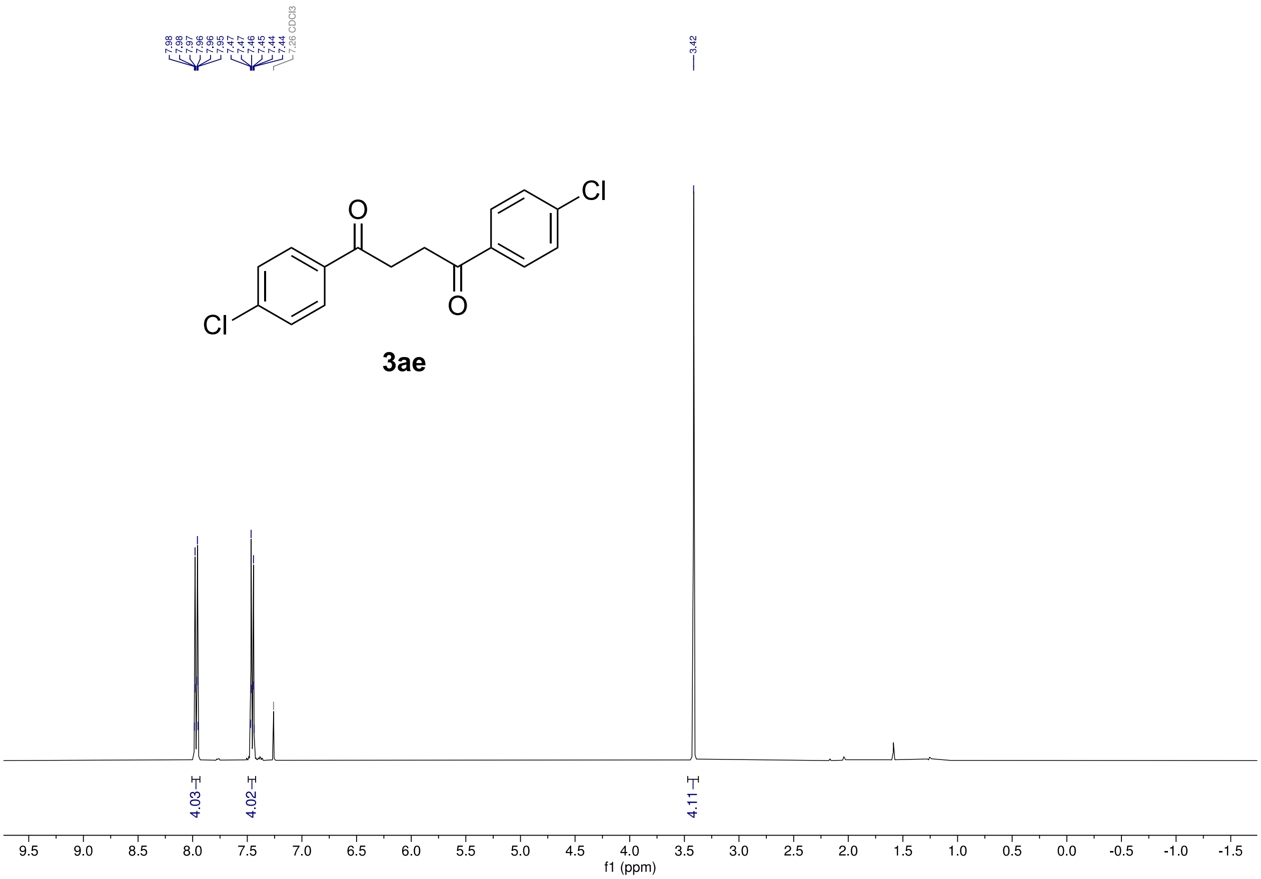

Figure S73. ^{1}H NMR of 1,4-bis(4-chlorophenyl)butane-1,4-dione (**3ae**) in $CDCl_3$ measured at 400 MHz.

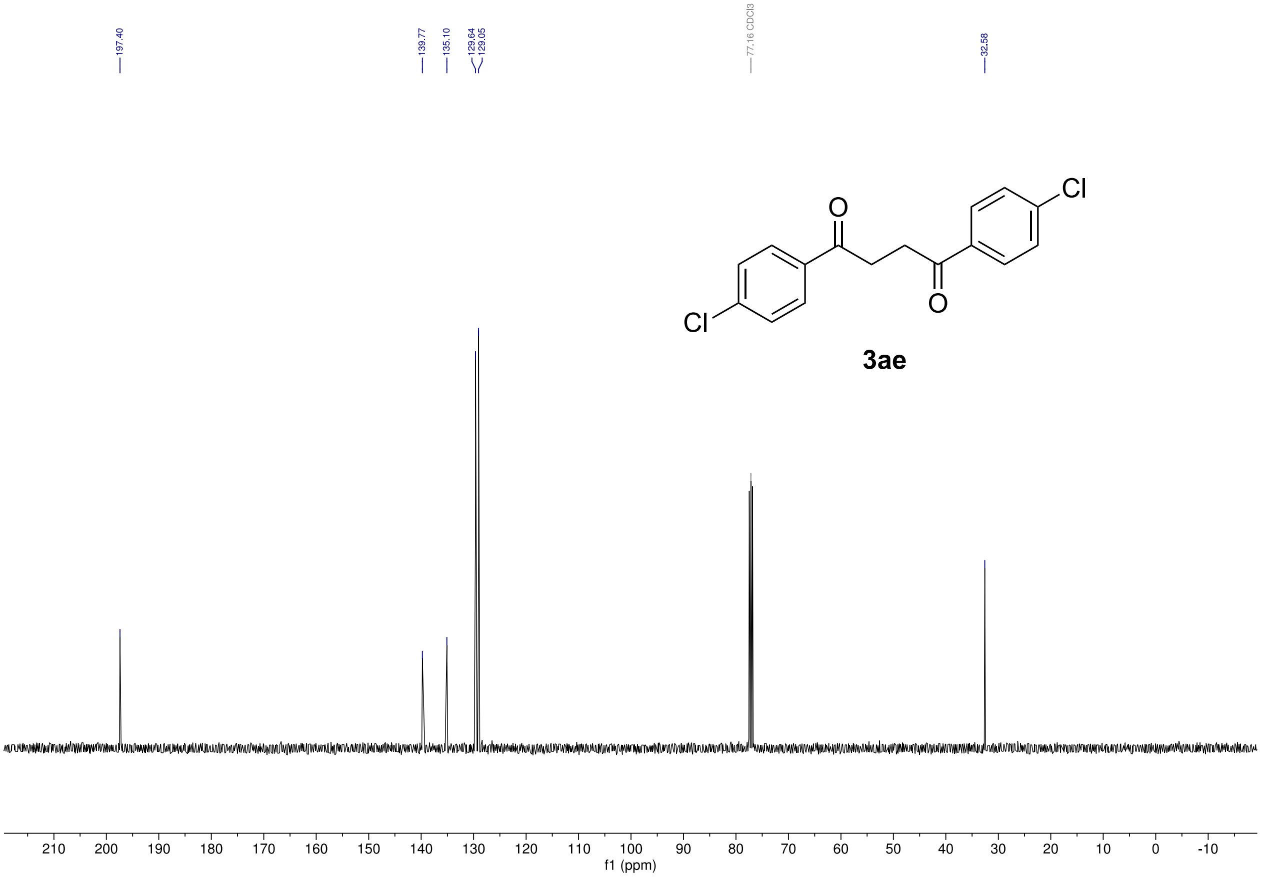

Figure S74. ^{13}C NMR of 1,4-bis(4-chlorophenyl)butane-1,4-dione (**3ae**) in $CDCl_3$ measured at 101 MHz.

1-(2-chlorophenyl)-4-(4-chlorophenyl)butane-1,4-dione

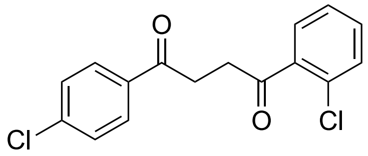

3af

3af was synthesized following the general procedure **4**, employing **1a** (169 mg, 1.00 mmol, 1.00 eq), **2f** (**373** mg, 1.50 mmol, 1.50 eq), CuCl (149 mg, 1.50 mmol, 1.50 eq), K_2CO_3 (138 mg, 1.00 mmol, 1.00 eq) at 80 °C and isolated via flash chromatography (23 g SiO_2, gradient from 10:90 to 20:80 EtOAc/*n*-hexane over 10 CV, gradient from 20:80 to 50:50 EtOAc/*n*-hexane over 5 CV) as a yellow solid (204 mg, 66%).

$C_{16}H_{12}Cl_2O_2$ (307,17 g/mol)

mp: 67-70°C

Rf: 0.30 (EtOAc/n-hexane = 1:10) [UV]

^{1}H NMR (400 MHz, $CDCl_3$): δ = 8.00 – 7.92 (m, 2H), 7.67 – 7.61 (m, 1H), 7.48 – 7.33 (m, 5H), 3.44 (m, 2H), 3.41 – 3.35 (m, 2H).

^{13}C NMR (101 MHz, $CDCl_3$): δ = 201.5, 197.1, 139.6, 139.0, 134.9, 131.8, 130.9, 130.5, 129.5, 129.3, 128.9, 127.0, 36.7, 33.0.

HRMS (ESI) m/z: $[M+Na]^+$ Calcd for $C_{16}H_{12}Cl_2O_2Na$ 329.0107; Found 329.0103.

IR (ATR, ṽ): 1675 (s, CO).

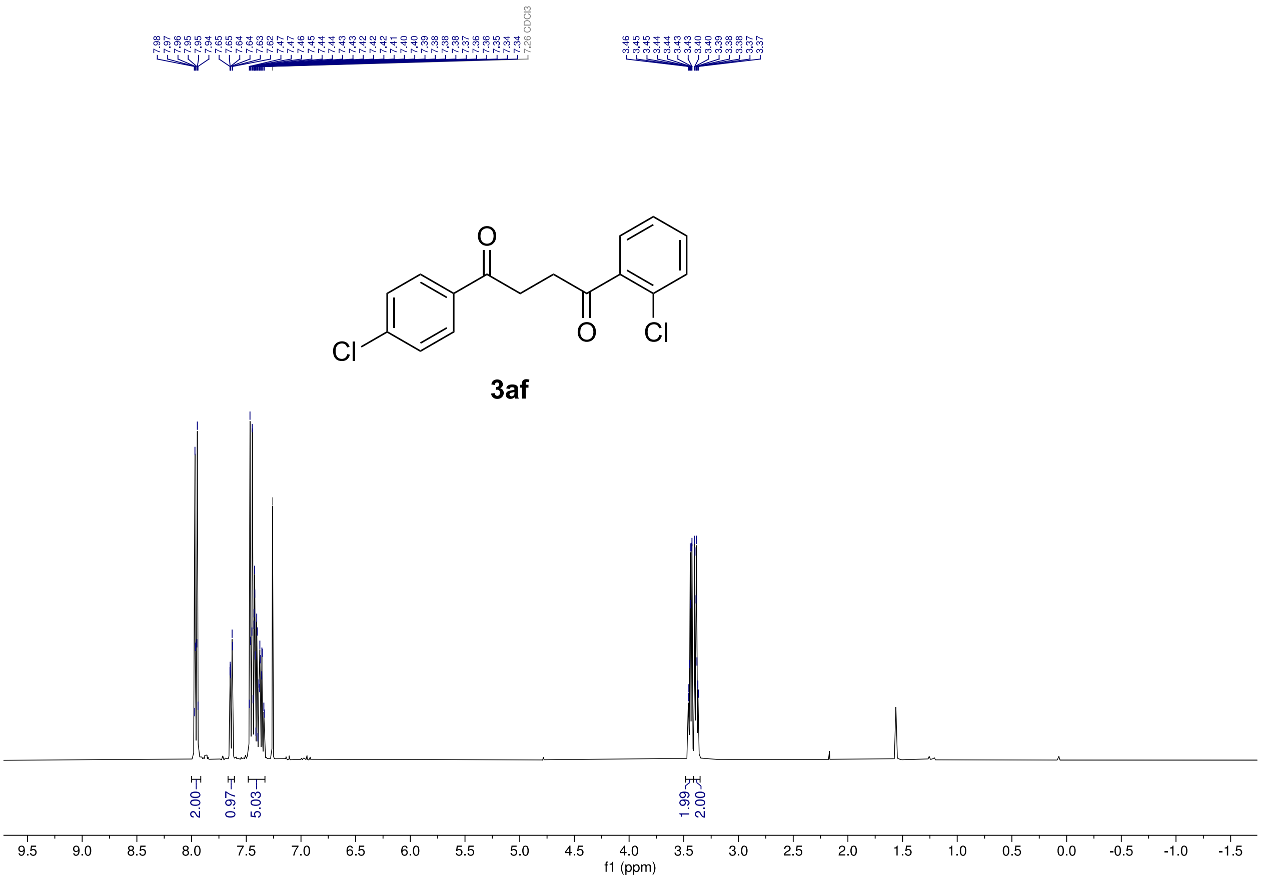

Figure S75. ^{1}H NMR of 1-(2-chlorophenyl)-4-(4-chlorophenyl)butane-1,4-dione (**3af**) in $CDCl_3$ measured at 400 MHz.

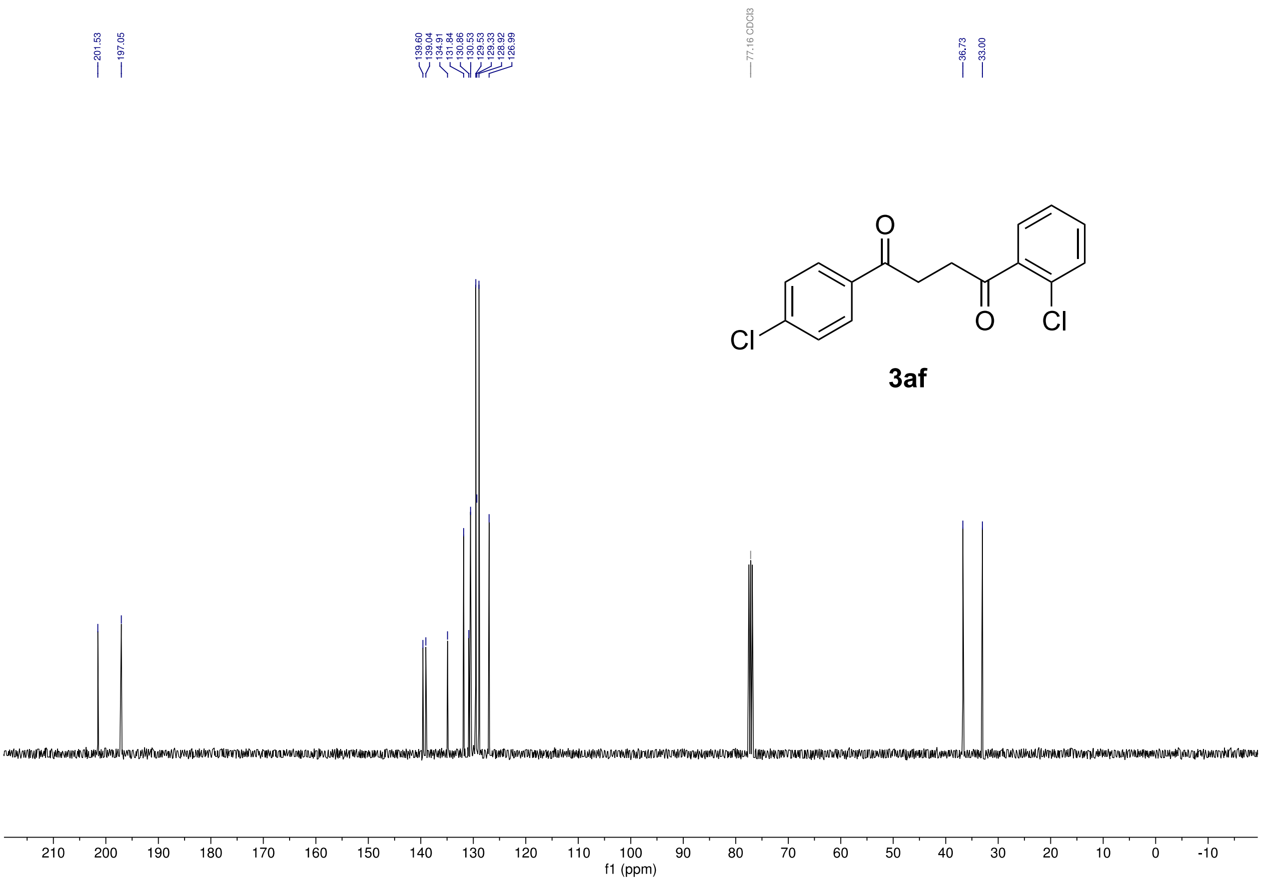

Figure S76. ^{13}C NMR of 1-(2-chlorophenyl)-4-(4-chlorophenyl)butane-1,4-dione (**3af**) in $CDCl_3$ measured at 101 MHz.

1-(4-chlorophenyl)-4-(*o*-tolyl)butane-1,4-dione

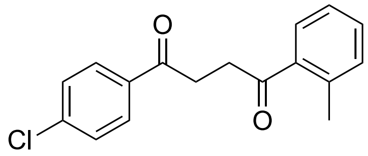

3ag

3ag was synthesized following the general procedure **4**, employing **1a** (169 mg, 1.00 mmol, 1.00 eq), **2g** (**342** mg, 1.50 mmol, 1.50 eq), CuCl (149 mg, 1.50 mmol, 1.50 eq), K_2CO_3 (138 mg, 1.00 mmol, 1.00 eq) at 80 °C and isolated via flash chromatography (23 g SiO_2, gradient from 10:90 to 20:80 EtOAc/*n*-hexane over 10 CV, gradient from 20:80 to 50:50 EtOAc/*n*-hexane over 5 CV) as a pale yellow solid (161 mg, 56%).

$C_{17}H_{15}ClO_2$ (286,76 g/mol)

mp: 82-83°C

Rf: 0.33 (EtOAc/n-hexane = 1:10) [UV]

^{1}H NMR (400 MHz, $CDCl_3$): δ = 8.00 – 7.95 (m, 2H), 7.81 (dd, *J* = 7.8, 1.4 Hz, 1H), 7.49 – 7.42 (m, 2H), 7.39 (td, *J* = 7.5, 1.5 Hz, 1H), 7.32 – 7.24 (m, 2H), 3.41 (m, 2H), 3.38 – 3.33 (m, 2H), 2.50 (s, 3H).

^{13}C NMR (101 MHz, $CDCl_3$): δ = 202.5, 197.5, 139.7, 138.3, 137.8, 135.2, 132.0, 131.5, 129.6, 129.0, 128.7, 125.8, 35.3, 32.9, 21.4.

HRMS (ESI) m/z: $[M+Na]^+$ Calcd for $C_{17}H_{15}ClO_2Na$ 309.0653; Found 309.0653.

IR (ATR, ṽ): 1672 (s, CO).

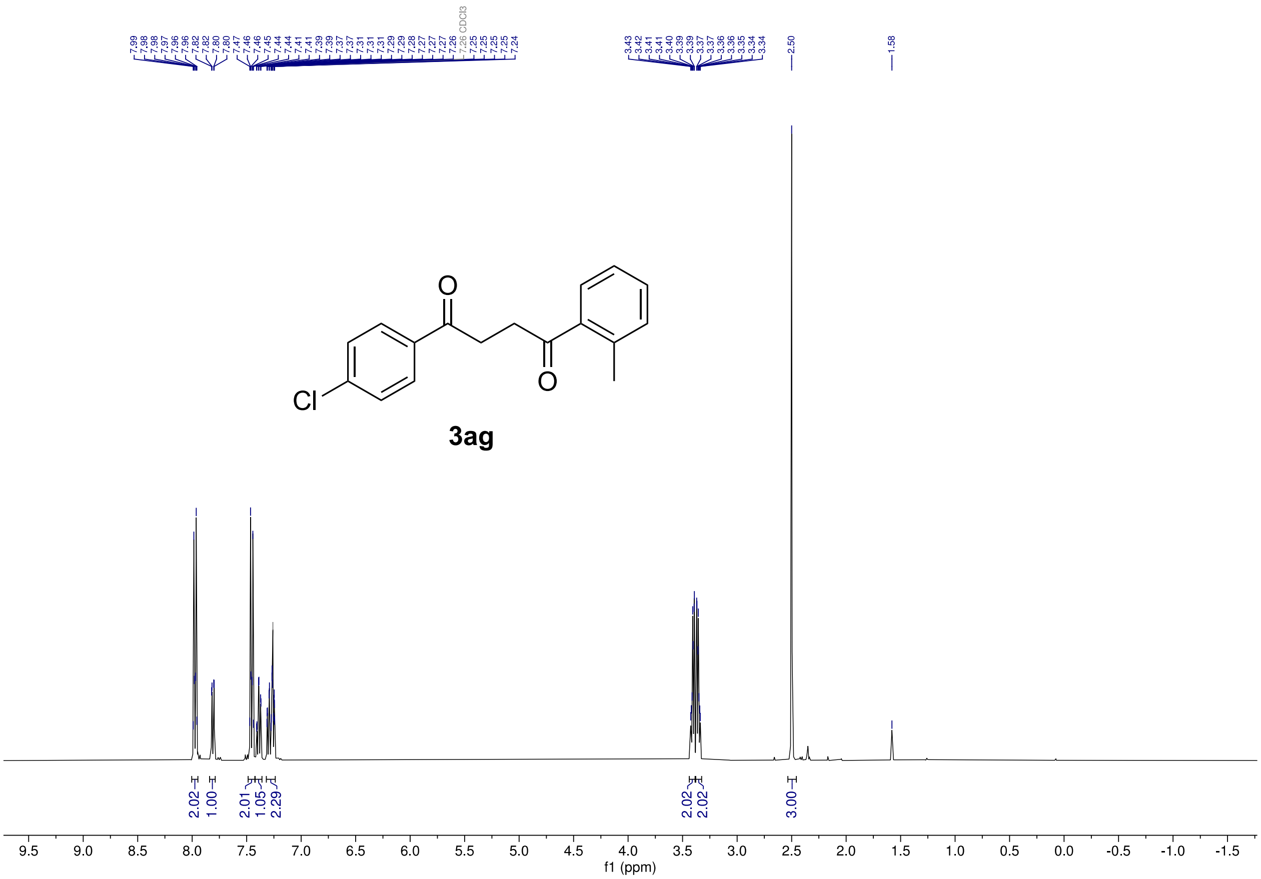

Figure S77. ^{1}H NMR of 1-(4-chlorophenyl)-4-(*o*-tolyl)butane-1,4-dione (**3ag**) in $CDCl_3$ measured at 400 MHz.

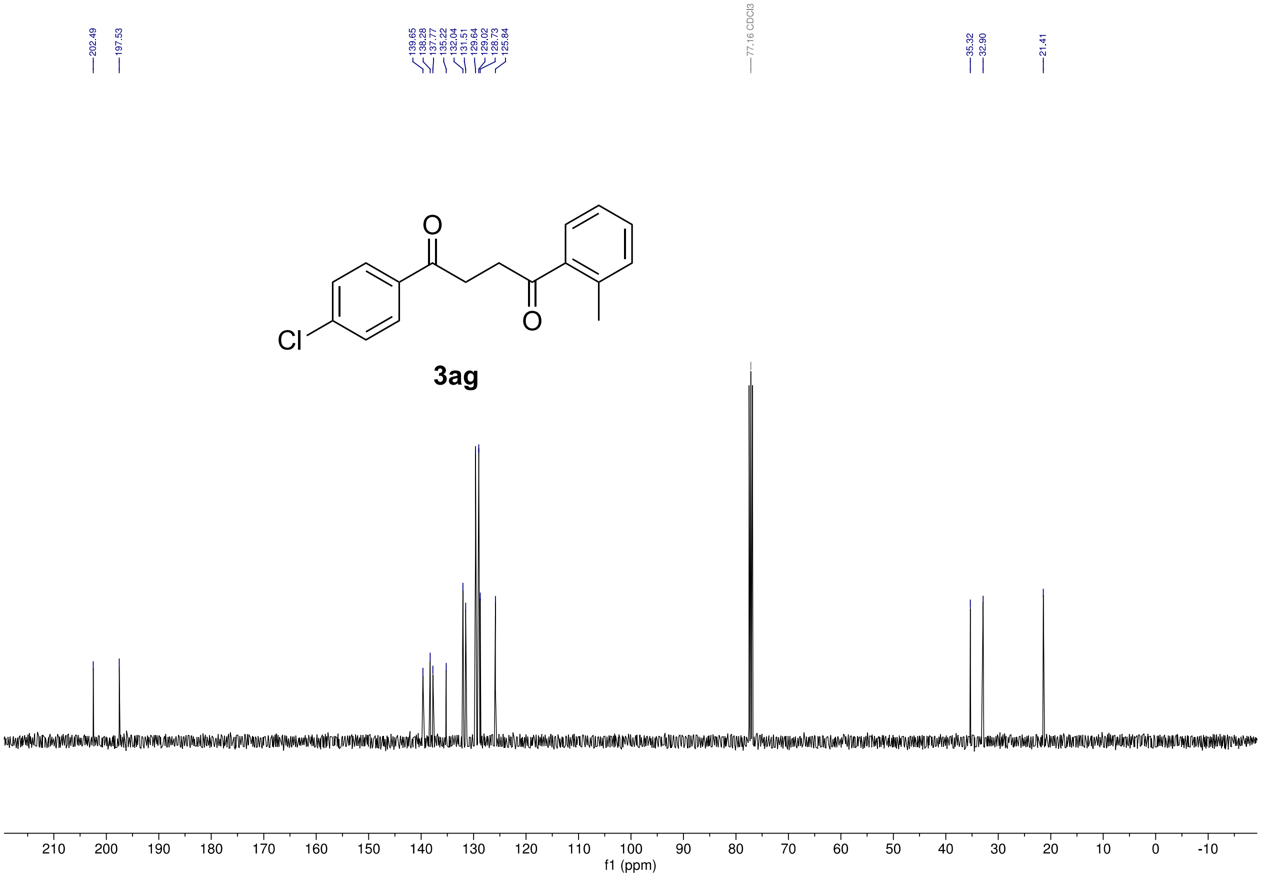

Figure S78. ^{13}C NMR of 1-(4-chlorophenyl)-4-(*o*-tolyl)butane-1,4-dione (**3ag**) in $CDCl_3$ measured at 101 MHz.

1-(4-chlorophenyl)-4-(furan-2-yl)butane-1,4-dione

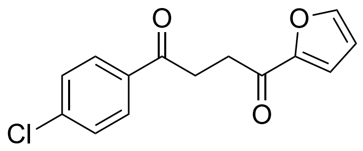

3ah

3ah was synthesized following the general procedure **4**, employing **1a** (169 mg, 1.00 mmol, 1.00 eq), **2h** (**306** mg, 1.50 mmol, 1.50 eq), CuCl (149 mg, 1.50 mmol, 1.50 eq), K_2CO_3 (138 mg, 1.00 mmol, 1.00 eq) at 80 °C and isolated via flash chromatography (23 g SiO_2, gradient from 10:90 to 20:80 EtOAc/*n*-hexane over 10 CV, gradient from 20:80 to 50:50 EtOAc/*n*-hexane over 5 CV) as a beige solid (212 mg, 81%).

$C_{14}H_{11}ClO_3$ (262,69 g/mol)

mp: 104-105°C

Rf: 0.12 (EtOAc/n-hexane = 1:10) [UV]

^{1}H NMR (400 MHz, $CDCl_3$): δ = 8.00 – 7.91 (m, 2H), 7.60 (dd, *J* = 1.7, 0.8 Hz, 1H), 7.49 – 7.41 (m, 2H), 7.27 – 7.25 (m, 1H), 6.55 (dd, *J* = 3.6, 1.7 Hz, 1H), 3.40 (m, 2H), 3.31 (m, 2H).

^{13}C NMR (101 MHz, $CDCl_3$): δ = 197.3, 187.8, 152.6, 146.5, 139.7, 135.1, 129.6, 129.0, 117.3, 112.4, 32.3, 32.3.

HRMS (ESI) m/z: $[M+Na]^+$ Calcd for $C_{14}H_{11}ClO_3Na$ 285.0289; Found 285.0290.

IR (ATR, ṽ): 1742 (s, CO), 1671 (s, CO).

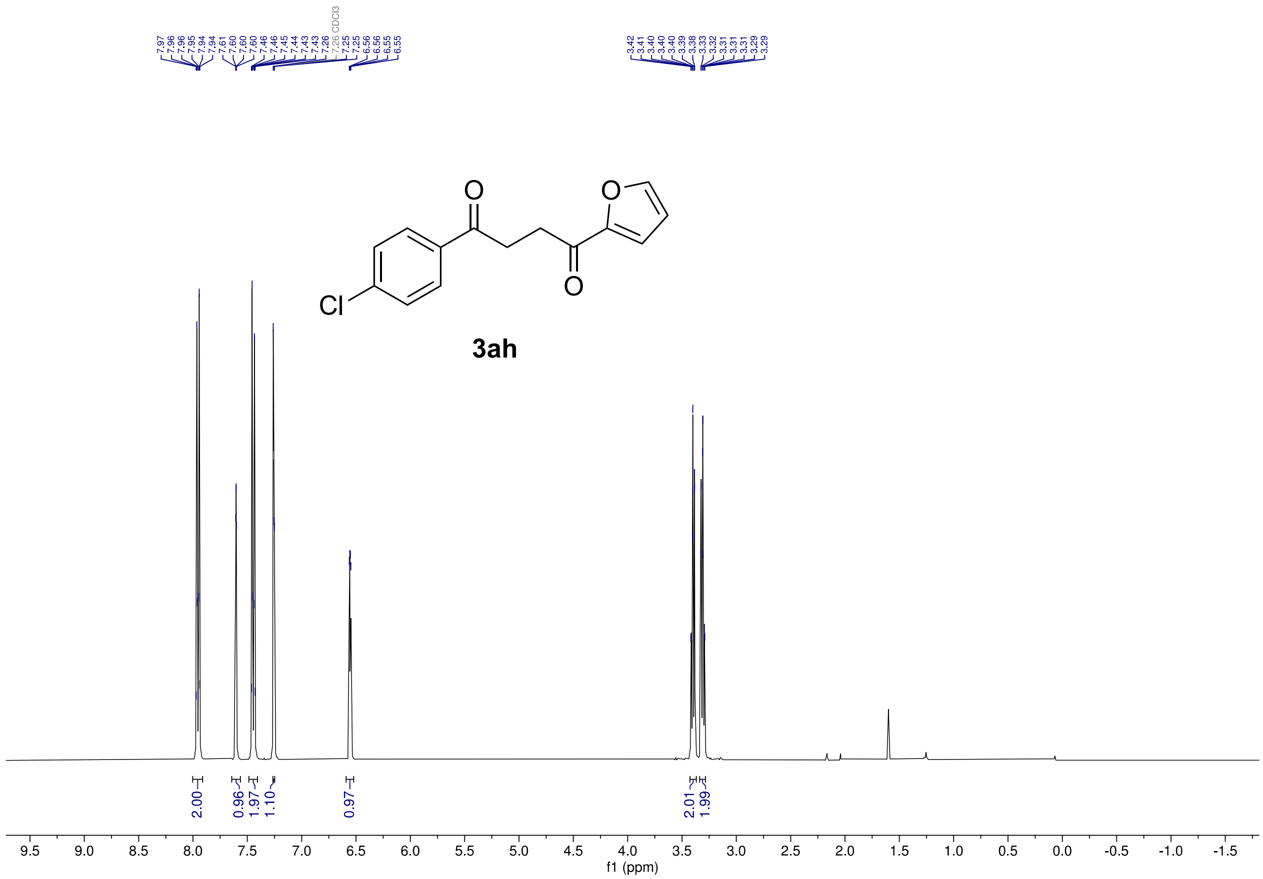

Figure S79. ^{1}H NMR of 1-(4-chlorophenyl)-4-(furan-2-yl)butane-1,4-dione (**3ah**) in $CDCl_3$ measured at 400 MHz.

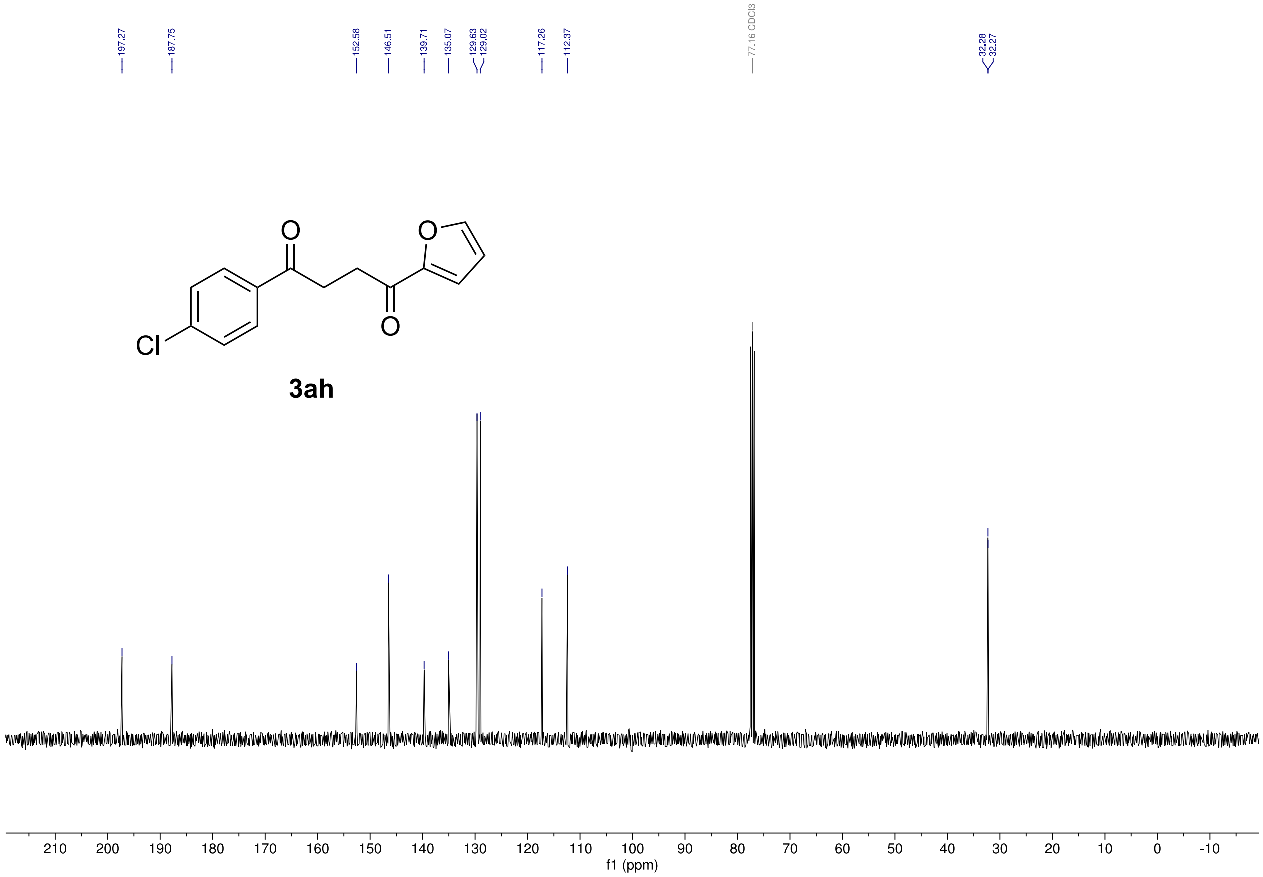

Figure S80. ^{13}C NMR of 1-(4-chlorophenyl)-4-(furan-2-yl)butane-1,4-dione (**3ah**) in $CDCl_3$ measured at 101 MHz.

1-(4-chlorophenyl)-4-(4-methoxyphenyl)butane-1,4-dione

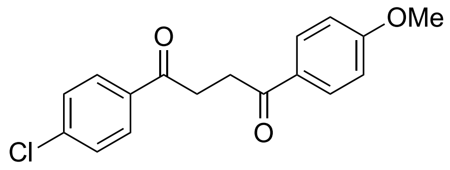

3ai

3ai was synthesized following the general procedure **4**, employing **1a** (169 mg, 1.00 mmol, 1.00 eq), **2i** (**366** mg, 1.50 mmol, 1.50 eq), CuCl (149 mg, 1.50 mmol, 1.50 eq), K_2CO_3 (138 mg, 1.00 mmol, 1.00 eq) at 90 °C and isolated via flash chromatography (23 g SiO_2, gradient from 10:90 to 20:80 EtOAc/*n*-hexane over 10 CV, gradient from 20:80 to 50:50 EtOAc/*n*-hexane over 5 CV) as a yellowish solid (269 mg, 89%).

$C_{17}H_{15}ClO_3$ (302,75 g/mol)

mp: 153-155°C

Rf: 0.15 (EtOAc/n-hexane = 1:10) [UV]

^{1}H NMR (400 MHz, $CDCl_3$): δ = 8.05 – 7.94 (m, 4H), 7.50 – 7.39 (m, 2H), 7.00 – 6.92 (m, 2H), 3.88 (s, 3H), 3.45 – 3.36 (m, 4H).

^{13}C NMR (101 MHz, $CDCl_3$): δ = 197.7, 197.0, 163.6, 139.5, 135.2, 130.4, 129.8, 129.6, 128.9, 113.8, 55.5, 32.6, 32.3.

HRMS (APCI) m/z: $[M+H]^+$ Calcd for $C_{17}H_{16}ClO_3$ 303.0783; Found 303.0777.

IR (ATR, ṽ): 1664 (m, CO).

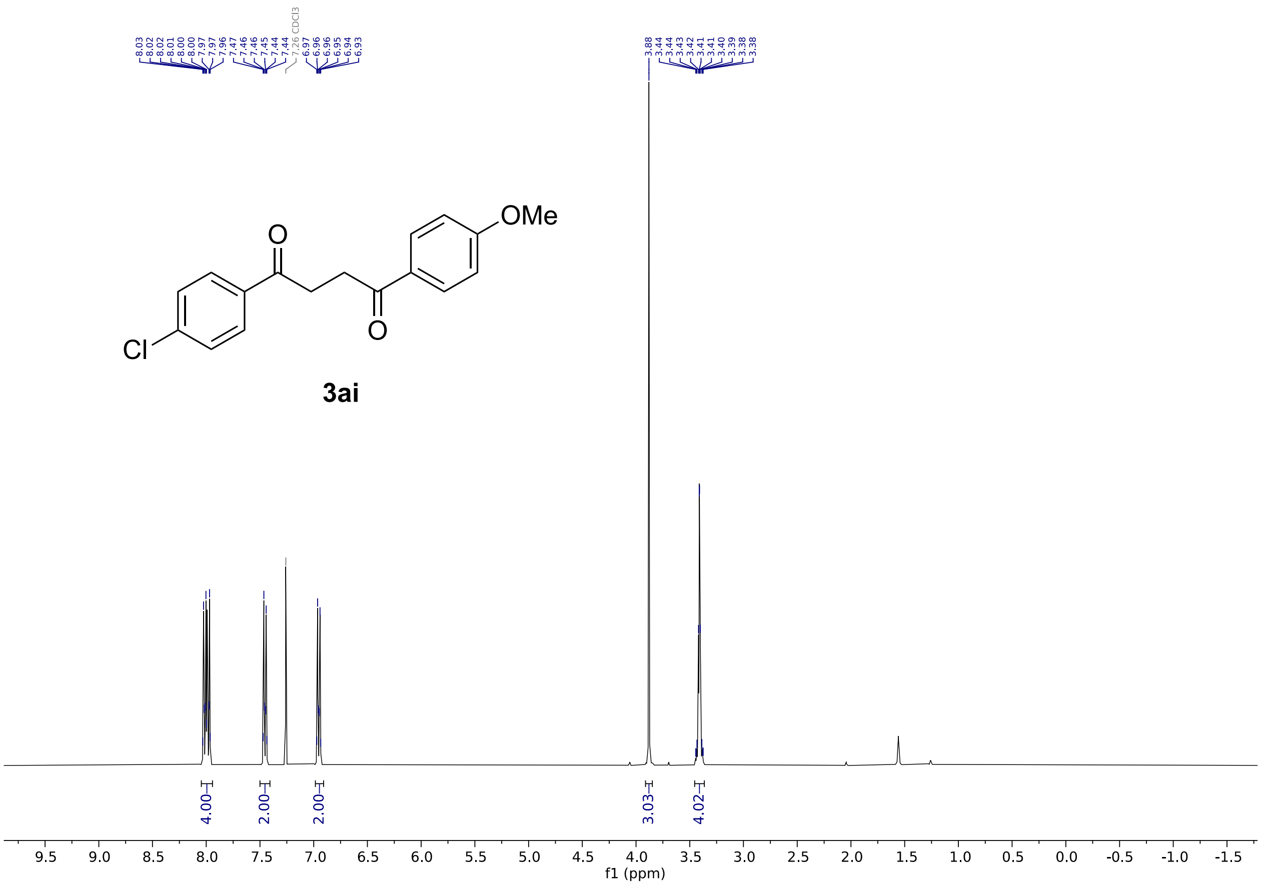

Figure S81. ^{1}H NMR of 1-(4-chlorophenyl)-4-(4-methoxyphenyl)butane-1,4-dione (**3ai**) in $CDCl_3$ measured at 400 MHz.

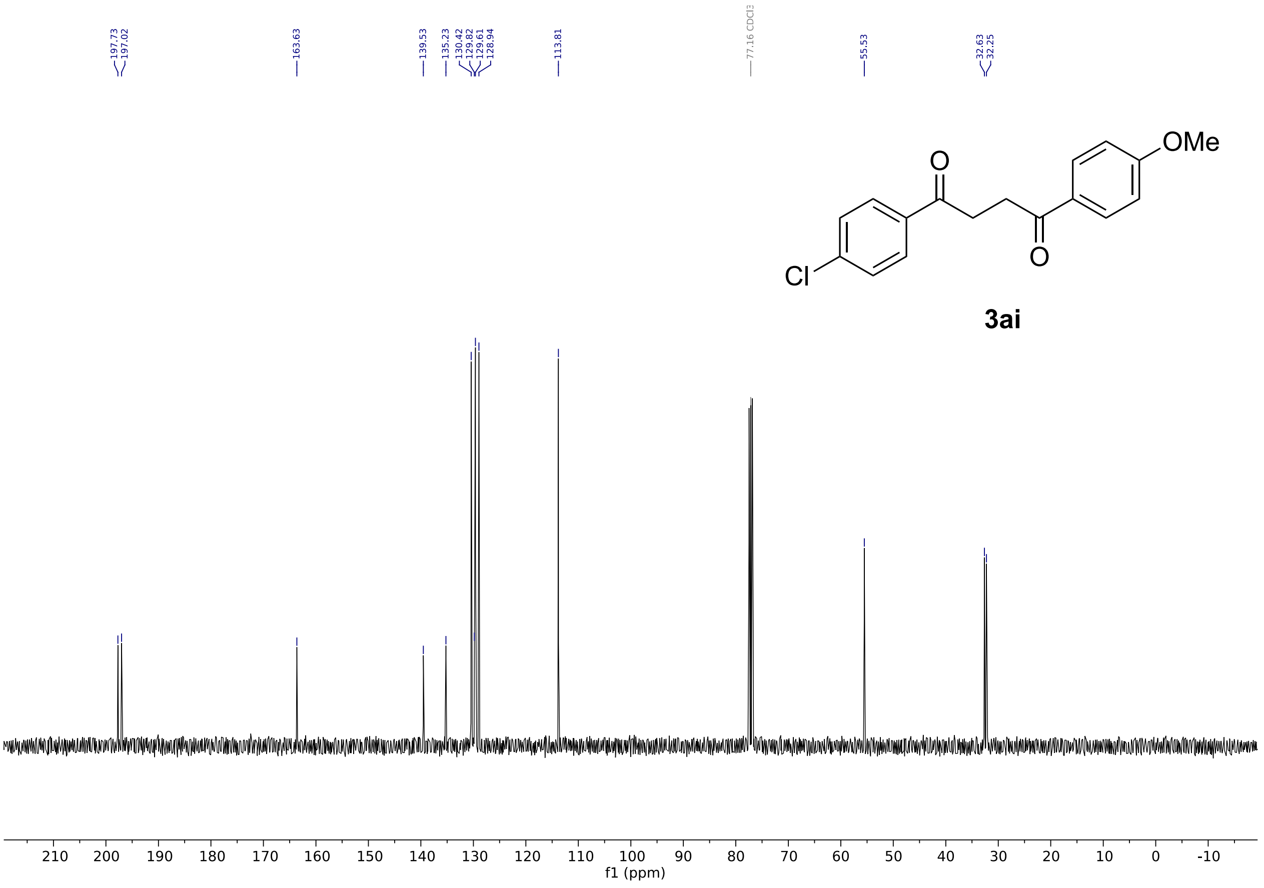

Figure S82. ^{13}C NMR of 1-(4-chlorophenyl)-4-(4-methoxyphenyl)butane-1,4-dione (**3ai**) in $CDCl_3$ measured at 101 MHz.

1-(4-chlorophenyl)-4-cyclopropylbutane-1,4-dione

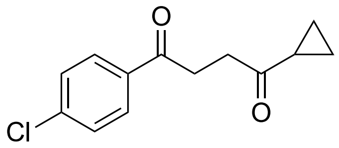

3aj

3aj was synthesized following the general procedure **4**, employing **1a** (169 mg, 1.00 mmol, 1.00 eq), **2j** (**267** mg, 1.50 mmol, 1.50 eq), CuCl (149 mg, 1.50 mmol, 1.50 eq), K_2CO_3 (138 mg, 1.00 mmol, 1.00 eq) at 80 °C and isolated via flash chromatography (23 g SiO_2, gradient from 10:90 to 20:80 EtOAc/*n*-hexane over 10 CV, gradient from 20:80 to 50:50 EtOAc/*n*-hexane over 5 CV) as a yellowish solid (197 mg, 83%).

$C_{13}H_{13}ClO_2$ (236,70 g/mol)

mp: 71-72°C

Rf: 0.18 (EtOAc/n-hexane = 1:10) [UV]

^{1}H NMR (400 MHz, $CDCl_3$): δ = 7.92 (d, *J* = 8.4 Hz, 2H), 7.43 (d, *J* = 8.3 Hz, 2H), 3.23 (t, *J* = 6.3 Hz, 2H), 3.04 (t, *J* = 6.3 Hz, 2H), 2.02 (m, 1H), 1.06 (m, 2H), 0.91 (m, 2H).

^{13}C NMR (101 MHz, $CDCl_3$): δ = 208.9, 197.3, 139.3, 135.0, 129.4, 128.7, 36.6, 32.1, 20.6, 10.7.

HRMS (ESI) m/z: $[M+Na]^+$ Calcd for $C_{13}H_{13}ClO_2Na$ 259.0496; Found 259.0498.

IR (ATR, ṽ): 1671 (s, CO).

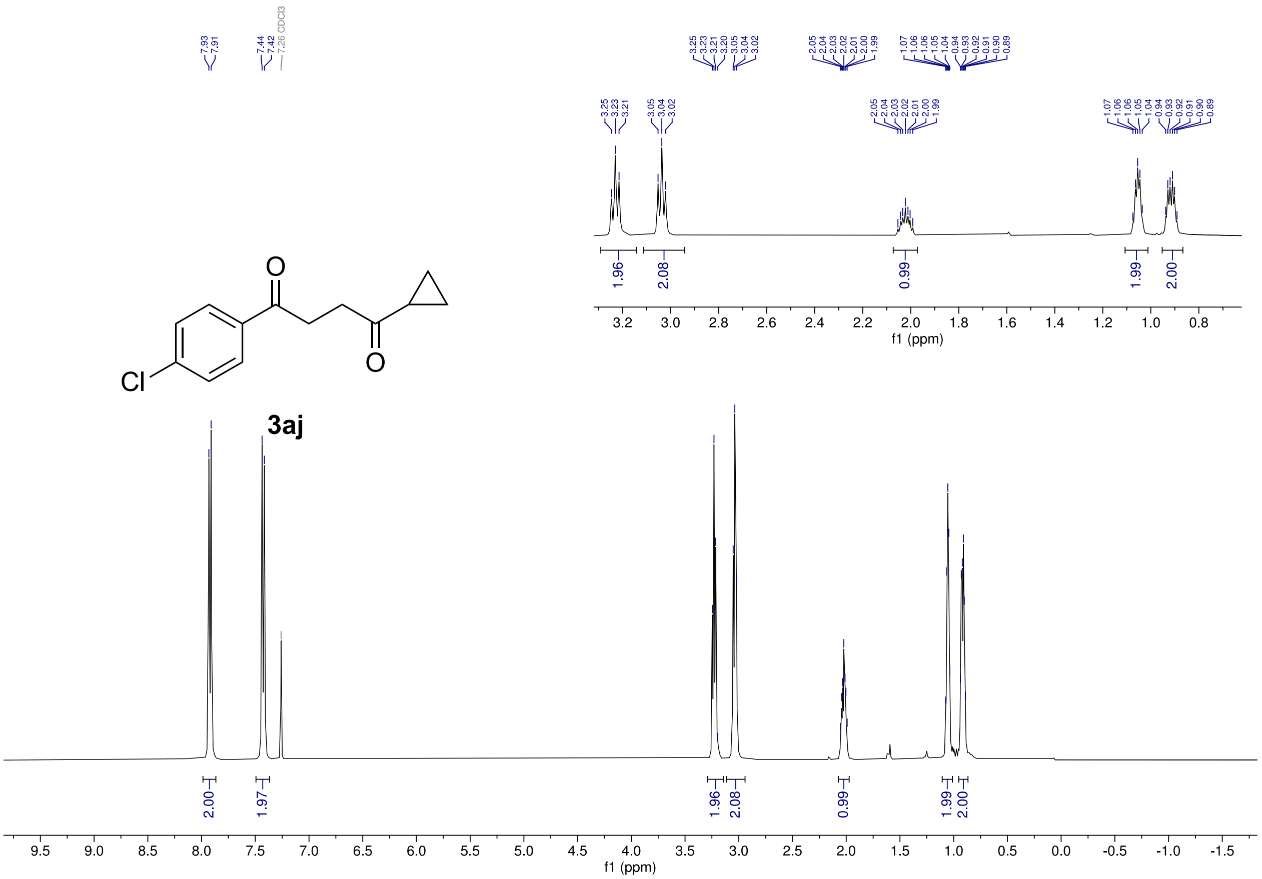

Figure S83. ^{1}H NMR of 1-(4-chlorophenyl)-4-cyclopropylbutane-1,4-dione (**3aj**) in $CDCl_3$ measured at 400 MHz.

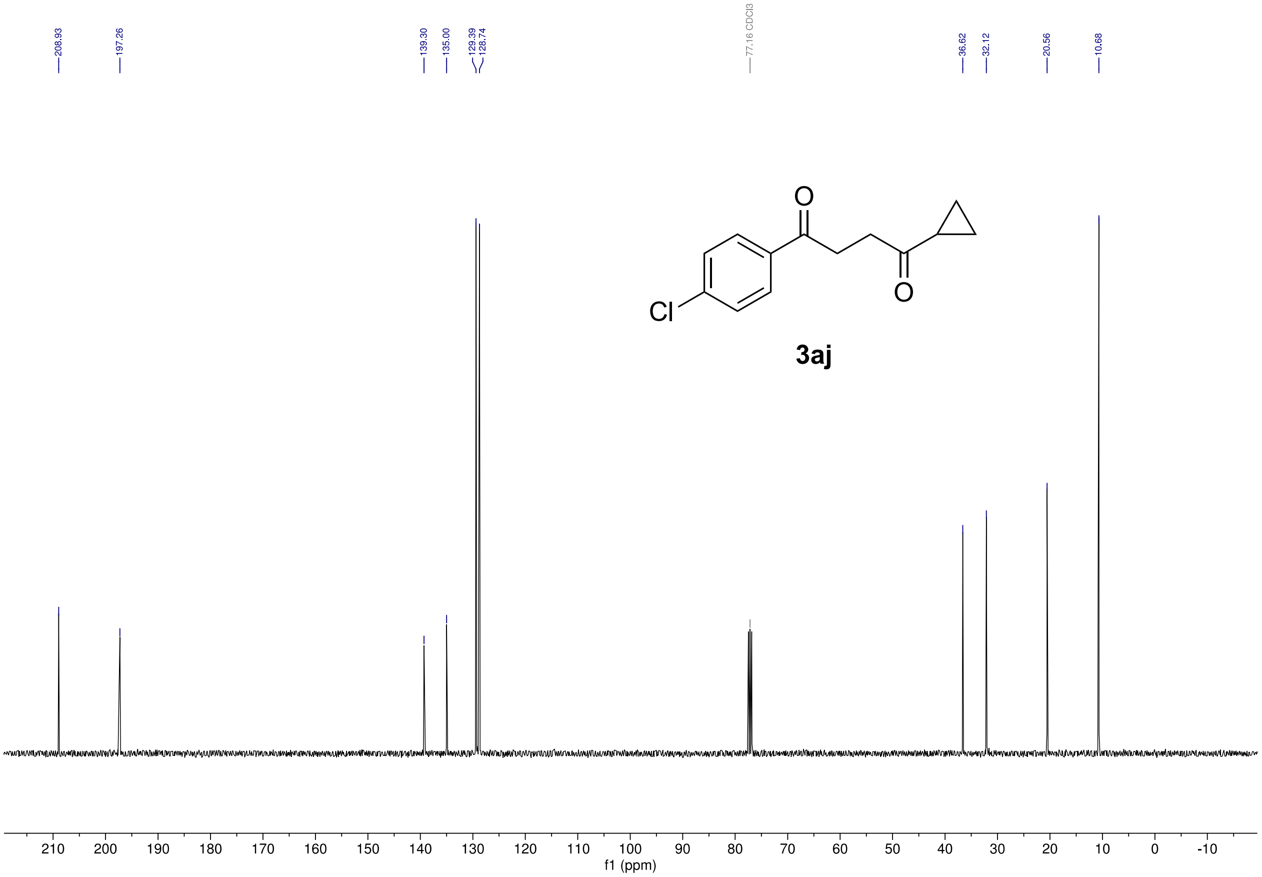

Figure S84. ^{13}C NMR of 1-(4-chlorophenyl)-4-cyclopropylbutane-1,4-dione (**3aj**) in $CDCl_3$ measured at 101 MHz.

1-(4-chlorophenyl)-4-(tetrahydro-2*H*-pyran-4-yl)butane-1,4-dione

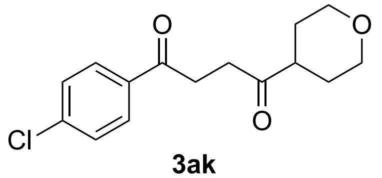

3ak

3ak was synthesized following the general procedure **4**, employing **1a** (169 mg, 1.00 mmol, 1.00 eq), **2k** (**334** mg, 1.50 mmol, 1.50 eq), CuCl (149 mg, 1.50 mmol, 1.50 eq), K_2CO_3 (138 mg, 1.00 mmol, 1.00 eq) at 80 °C and isolated via flash chromatography (23 g SiO_2, gradient from 10:90 to 20:80 EtOAc/*n*-hexane over 10 CV, gradient from 20:80 to 50:50 EtOAc/*n*-hexane over 5 CV) as a pale ochre solid (246 mg, 88%).

$C_{15}H_{17}ClO_3$ (280,75 g/mol)

mp: 88-89°C

Rf: 0.06 (EtOAc/n-hexane = 1:10) [UV]

^{1}H NMR (400 MHz, $CDCl_3$): δ = 7.98 – 7.83 (m, 2H), 7.50 – 7.37 (m, 2H), 4.01 (m, 2H), 3.45 (td, *J* = 11.5, 2.5 Hz, 2H), 3.29 – 3.21 (m, 2H), 2.92 – 2.85 (m, 2H), 2.69 (tt, *J* = 11.3, 4.0 Hz, 1H), 1.84 (m, 2H), 1.74 (m, 2H).

^{13}C NMR (101 MHz, $CDCl_3$): δ = 210.5, 197.4, 139.7, 135.0, 129.6, 129.0, 67.3, 47.7, 33.9, 32.4, 28.3.

HRMS (ESI) m/z: $[M+Na]^+$ Calcd for $C_{15}H_{17}ClO_3Na$ 303.0758; Found 303.0757.

IR (ATR, ṽ): 1679 (m, CO).

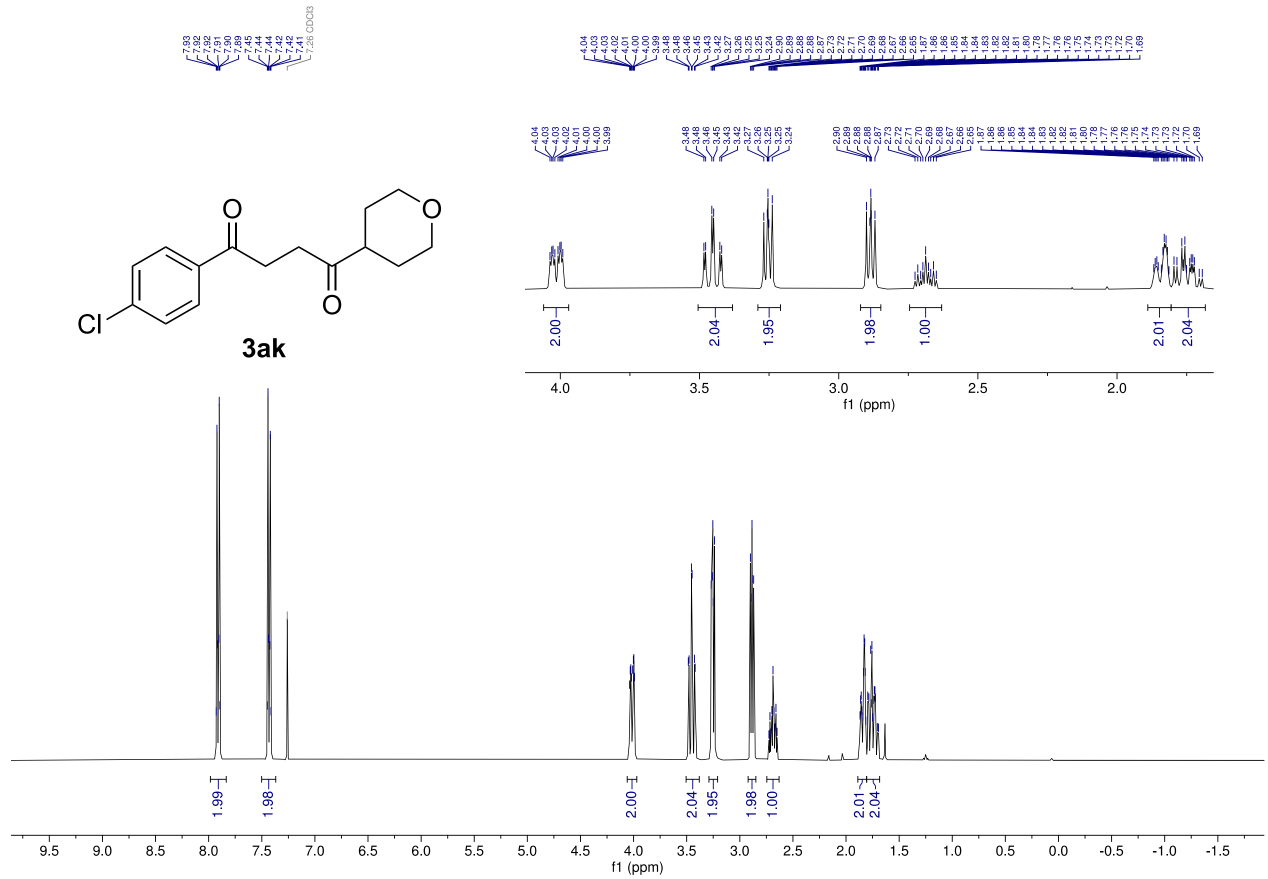

Figure S85. ^{1}H NMR of 1-(4-chlorophenyl)-4-(tetrahydro-2*H*-pyran-4-yl)butane-1,4-dione (**3ak**) in $CDCl_3$ measured at 400 MHz.

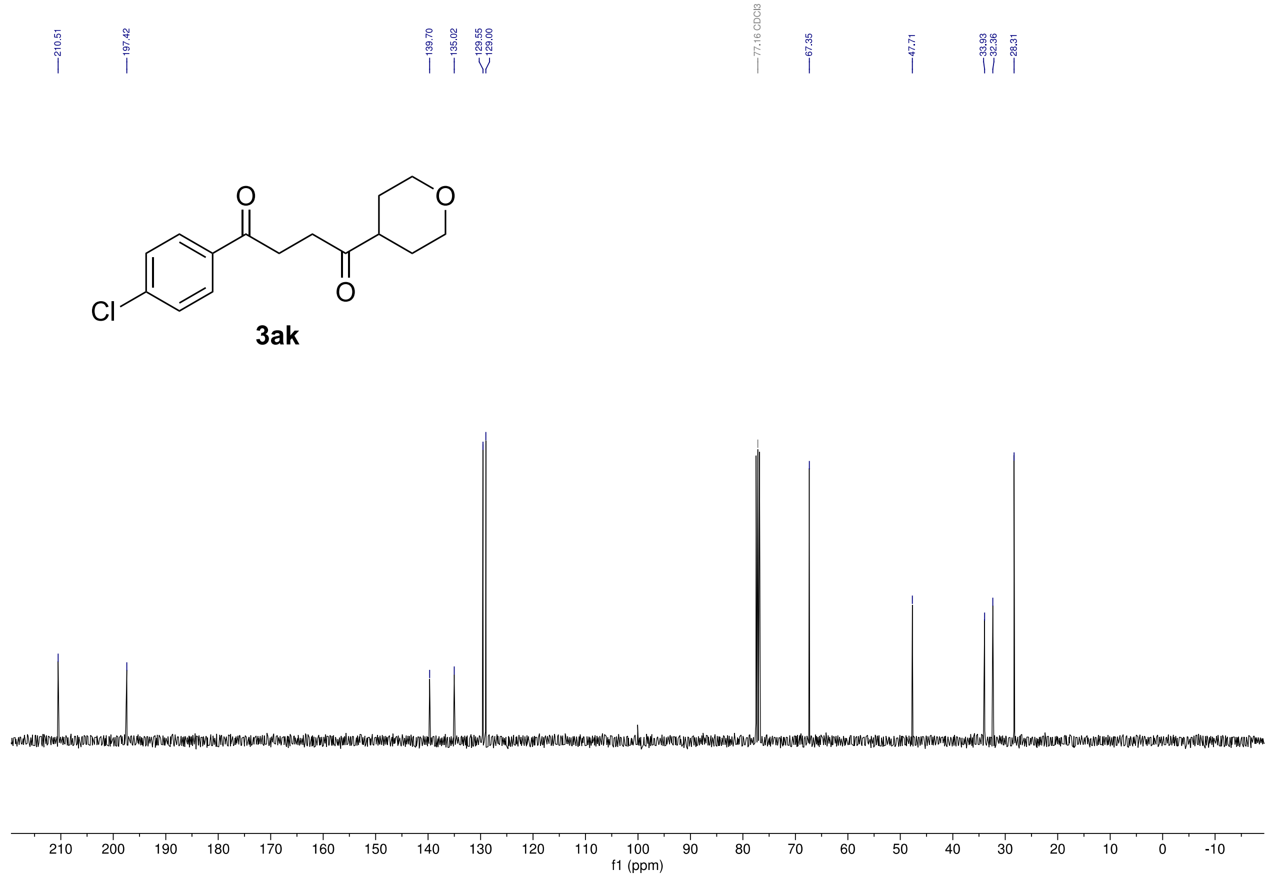

Figure S86. ^{13}C NMR of 1-(4-chlorophenyl)-4-(tetrahydro-2*H*-pyran-4-yl)butane-1,4-dione (**3ak**) in $CDCl_3$ measured at 101 MHz.

(*E*)-1-(4-chlorophenyl)-6-phenylhex-5-ene-1,4-dione

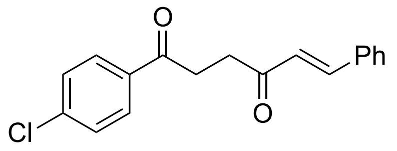

3al

3al was synthesized following the general procedure **4**, employing **1a** (169 mg, 1.00 mmol, 1.00 eq), **2l** (**361** mg, 1.50 mmol, 1.50 eq), CuCl (149 mg, 1.50 mmol, 1.50 eq), K_2CO_3 (138 mg, 1.00 mmol, 1.00 eq) at 80 °C and isolated via flash chromatography (23 g SiO_2, gradient from 10:90 to 20:80 EtOAc/*n*-hexane over 10 CV, gradient from 20:80 to 50:50 EtOAc/*n*-hexane over 5 CV) as a pale ochre solid (223 mg, 75%).

$C_{18}H_{15}ClO_2$ (298,77 g/mol)

mp: 134-135°C

Rf: 0.21 (EtOAc/n-hexane = 1:10) [UV]

^{1}H NMR (400 MHz, $CDCl_3$): δ = 8.00 – 7.93 (m, 2H), 7.64 (d, *J* = 16.2 Hz, 1H), 7.60 – 7.53 (m, 2H), 7.48 – 7.43 (m, 2H), 7.41 (m, 3H), 6.82 (d, *J* = 16.3 Hz, 1H), 3.36 (t, *J* = 6.4 Hz, 2H), 3.16 (t, *J* = 6.4 Hz, 2H).

^{13}C NMR (101 MHz, $CDCl_3$): δ = 198.5, 197.6, 143.1, 139.7, 135.2, 134.6, 130.7, 129.6, 129.1, 129.0, 128.4, 126.1, 34.5, 32.6.

HRMS (ESI) m/z: $[M+Na]^+$ Calcd for $C_{18}H_{15}ClO_2Na$ 321.0653; Found 321.0655.

IR (ATR, ṽ): 1680 (s, CO), 1612 (m, CO).

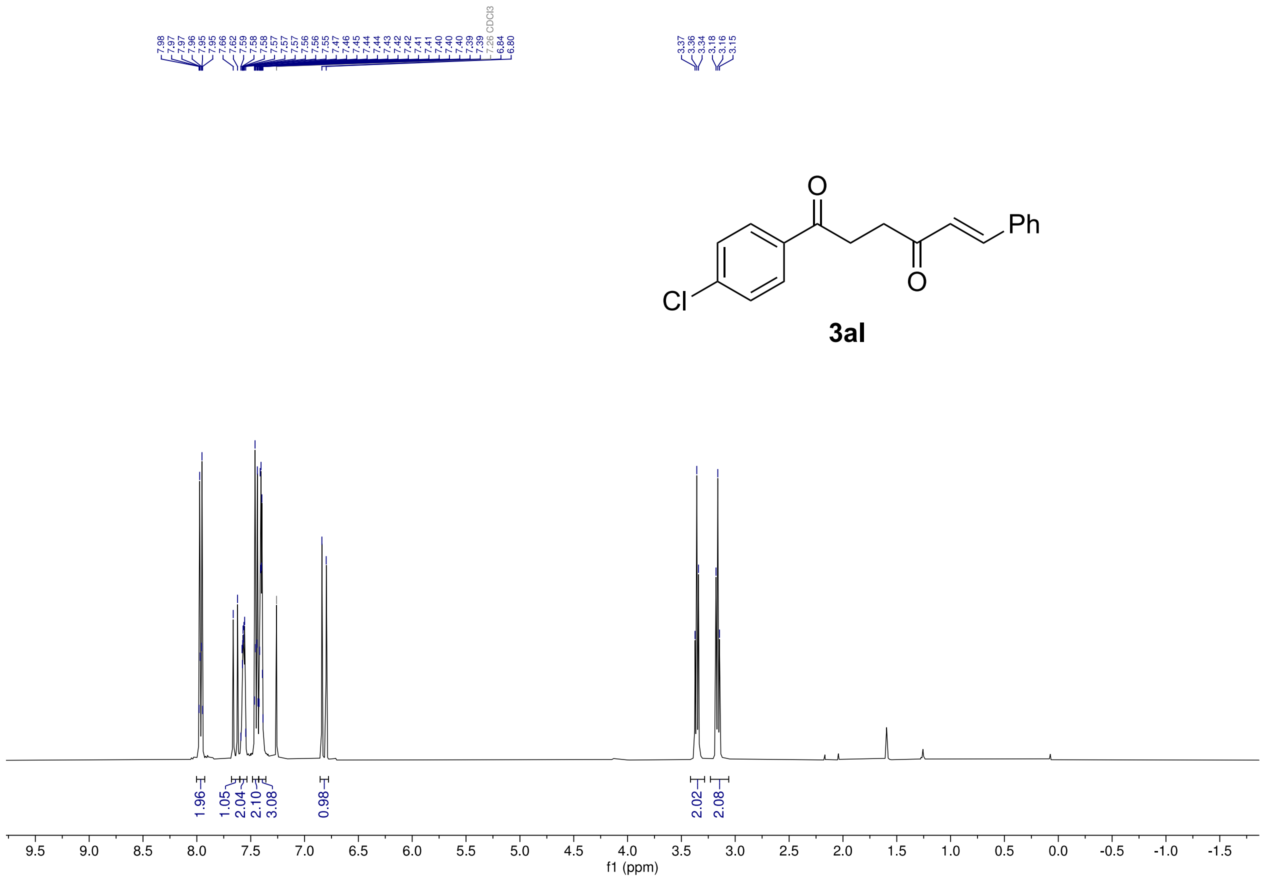

Figure S87. ^{13}C NMR of 1-(4-chlorophenyl)-4-(tetrahydro-2*H*-pyran-4-yl)butane-1,4-dione (**3al**) in $CDCl_3$ measured at 400 MHz.

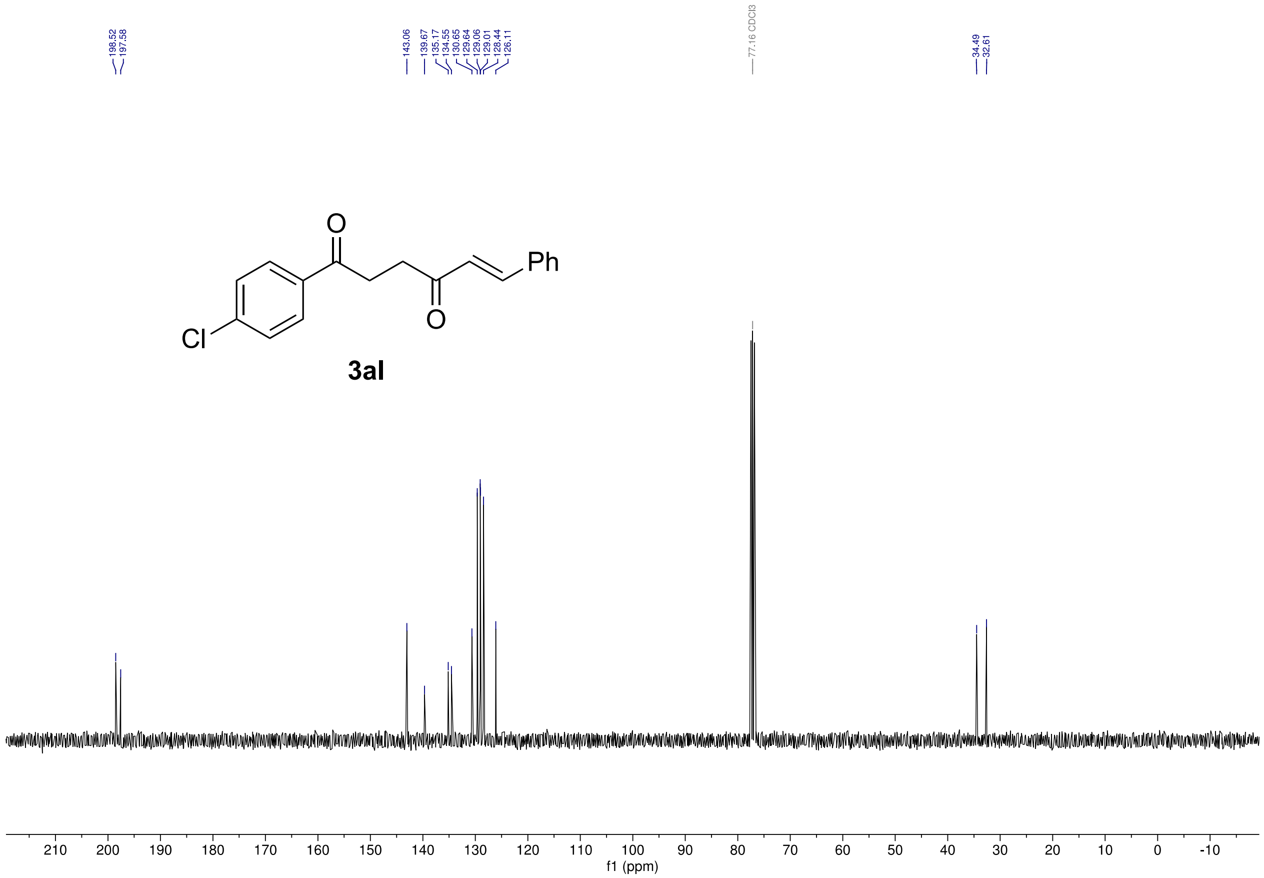

Figure S88. ^{13}C NMR of 1-(4-chlorophenyl)-4-(tetrahydro-2*H*-pyran-4-yl)butane-1,4-dione (**3al**) in $CDCl_3$ measured at 101 MHz.

8. References

(1) Liu, C.; Szostak, M. Decarbonylative Thioetherification by Nickel Catalysis using Air- and Moisture-Stable Nickel Precatalysts. *Chem. Comm.* **2018**, *54*, 2130-2133.

(2) Geiger, V. J.; Lefèvre, G.; Fleischer, I. Iron-Catalyzed Cross-Coupling of Thioesters and Organomanganese Reagents. *Chem. Eur. J.* **2022**, *28*, e202202212.

(3) Zhang, G.; Jia, F.; Gooßen, L. J. Regioselective C−H Alkylation via Carboxylate-Directed Hydroarylation in Water. *Chem. Eur. J.* **2018**, *24*, 4537-4541.

(4) Fang, L.; Fan, S.; Wu, W.; Li, T.; Zhu, J. Ruthenium-Catalyzed Room-Temperature Coupling of α-Keto Sulfoxonium Ylides and Cyclopropanols for δ-Diketone Synthesis. *Chem. Comm.* **2021**, *57*, 7386-7389.

(5) (a) Neese, F. The SHARK Integral Generation and Digestion System. *J. Comput. Chem.* **2023**, *44*, 381-396. (b) Neese, F. Software Update: The ORCA Program System—Version 5.0. *Wiley Interdiscip. Rev. Comput. Mol. Sci.* **2022**, *12*, e1606.

(6) Neese, F. An Improvement of the Resolution of the Identity Approximation for the Formation of the Coulomb Matrix. *J. Comput. Chem.* **2003**, *24*, 1740-1747.

(7) Weigend, F. Accurate Coulomb-Fitting Basis Sets for H to Rn. *Phys. Chem. Chem. Phys.* **2006**, *8*, 1057-1065.

(8) (a) Helmich-Paris, B.; de Souza, B.; Neese, F.; Izsák, R. An Improved Chain of Spheres for Exchange Algorithm. *J. Chem. Phys.* **2021**, *155*, 104109. (b) Neese, F.; Wennmohs, F.; Hansen, A.; Becker, U. Efficient, Approximate and Parallel Hartree–Fock and Hybrid DFT Calculations. A 'Chain-of-Spheres' Algorithm for the Hartree–Fock Exchange. *Chemical Physics* **2009**, *356*, 98-109.

(9) Kästner, J.; Carr, J. M.; Keal, T. W.; Thiel, W.; Wander, A.; Sherwood, P. DL-FIND: An Open-Source Geometry Optimizer for Atomistic Simulations. *J. Phys. Chem. A* **2009**, *113*, 11856-11865.

(10) Sherwood, P.; de Vries, A. H.; Guest, M. F.; Schreckenbach, G.; Catlow, C. R. A.; French, S. A.; Sokol, A. A.; Bromley, S. T.; Thiel, W.; Turner, A. J.; et al. QUASI: A General Purpose Implementation of the QM/MM Approach and its Application to Problems in Catalysis. *J. Mol. Struct. THEOCHEM* **2003**, *632*, 1-28.

(11) (a) Grimme, S. Exploration of Chemical Compound, Conformer, and Reaction Space with Meta-Dynamics Simulations Based on Tight-Binding Quantum Chemical Calculations. *J. Chem. Theory Comput.* **2019**, *15*, 2847-2862. (b) Pracht, P.; Grimme, S.; Bannwarth, C.; Bohle, F.; Ehlert, S.; Feldmann, G.; Gorges, J.; Müller, M.; Neudecker, T.; Plett, C.; et al. CREST—A Program for the Exploration of Low-Energy Molecular Chemical Space. *J. Chem. Phys.* **2024**, *160*, 114110. (c) Pracht, P.; Bohle, F.; Grimme, S. Automated Exploration of the Low-Energy Chemical Space with Fast Quantum Chemical Methods. *Phys. Chem. Chem. Phys.* **2020**, *22*, 7169-7192.

(12) Bannwarth, C.; Ehlert, S.; Grimme, S. GFN2-xTB—An Accurate and Broadly Parametrized Self-Consistent Tight-Binding Quantum Chemical Method with Multipole Electrostatics and Density-Dependent Dispersion Contributions. *J. Chem. Theory Comput.* **2019**, *15*, 1652-1671.

(13) Müller, M.; Hansen, A.; Grimme, S. ωB97X-3c: A Composite Range-Separated Hybrid DFT Method with a Molecule-Optimized Polarized Valence Double-ζ Basis Set. *J. Chem. Phys.* **2023**, *158*, 014103.

(14) Mardirossian, N.; Head-Gordon, M. ωB97M-V: A Combinatorially Optimized, Range-Separated Hybrid, Meta-GGA Density Functional with VV10 Nonlocal Correlation. *J. Chem. Phys.* **2016**, *144*, 214110.

(15) Weigend, F.; Ahlrichs, R. Balanced Basis Sets of Split Valence, Triple Zeta Valence and Quadruple Zeta Valence Quality for H to Rn: Design and Assessment of Accuracy. *Phys. Chem. Chem. Phys.* **2005**, *7*, 3297-3305.

(16) (a) Marenich, A. V.; Cramer, C. J.; Truhlar, D. G. Universal Solvation Model Based on Solute Electron Density and on a Continuum Model of the Solvent Defined by the Bulk Dielectric Constant and Atomic Surface Tensions. *J. Phys. Chem. B* **2009**, *113*, 6378-6396. (b) Garcia-Ratés, M.; Neese, F. Effect of the Solute Cavity on the Solvation Energy and its Derivatives within the Framework of the Gaussian Charge Scheme. *J. Comput. Chem.* **2020**, *41*, 922-939.

(17) He, X.-P.; Shu, Y.-J.; Dai, J.-J.; Zhang, W.-M.; Feng, Y.-S.; Xu, H.-J. Copper-Catalysed Ring-Opening Trifluoromethylation of Cyclopropanols. *Org. Biomol. Chem.* **2015**, *13*, 7159-7163.

(18) Jia, K.; Zhang, F.; Huang, H.; Chen, Y. Visible-Light-Induced Alkoxyl Radical Generation Enables Selective C(sp3)–C(sp3) Bond Cleavage and Functionalizations. *J. Am. Chem. Soc.* **2016**, *138*, 1514-1517.

(19) Wang, S.; Miao, E.; Wang, H.; Song, B.; Huang, W.; Yang, W. Rh-Catalyzed Cascade C–H Activation/C–C Cleavage/Cyclization of Carboxylic Acids with Cyclopropanols. *Chem. Comm.* **2021**, *57*, 5929-5932.

(20) Konik, Y. A.; Kudrjashova, M.; Konrad, N.; Kaabel, S.; Järving, I.; Lopp, M.; Kananovich, D. G. Two-Step Conversion of Carboxylic Esters into Distally Fluorinated Ketones via Ring Cleavage of Cyclopropanol Intermediates: Application of Sulfinate Salts as Fluoroalkylating Reagents. *Org. Biomol. Chem.* **2017**, *15*, 4635-4643.

(21) Ye, Z.; Cai, X.; Li, J.; Dai, M. Catalytic Cyclopropanol Ring Opening for Divergent Syntheses of γ-Butyrolactones and δ-Ketoesters Containing All-Carbon Quaternary Centers. *ACS Catal.* **2018**, *8*, 5907-5914.

(22) Lorenz, J. C.; Long, J.; Yang, Z.; Xue, S.; Xie, Y.; Shi, Y. A Novel Class of Tunable Zinc Reagents (RXZnCH2Y) for Efficient Cyclopropanation of Olefins. *J. Org. Chem.* **2004**, *69*, 327-334.

(23) Tsuchida, H.; Tamura, M.; Hasegawa, E. Cyclization and Ring-Expansion Processes Involving Samarium Diiodide Promoted Reductive Formation and Subsequent Oxidative Ring Opening of Cyclopropanol Derivatives. *J. Org. Chem.* **2009**, *74*, 2467-2475.

(24) Kananovich, D. G.; Konik, Y. A.; Zubrytski, D. M.; Järving, I.; Lopp, M. Simple Access to β-Trifluoromethyl-Substituted Ketones via Copper-Catalyzed Ring-Opening Trifluoromethylation of Substituted Cyclopropanols. *Chem. Comm.* **2015**, *51*, 8349-8352.
(25) Lee, M.; Heo, J.; Kim, D.; Chang, S. On the Origin of Rh-Catalyzed Selective Ring-Opening Amidation of Substituted Cyclopropanols to Access β2-Amino Ketones. *J. Am. Chem. Soc.* **2022**, 3667-3675.
(26) Kulinkovich, O. G.; Shevchuk, T. A.; Isakov, V. E.; Prokhorevich, K. N. Reductive Ethylation of Homoallyl Alcohols with a Disubstituted Double Bond with Ethylmagnesium Bromide in the Presence of Titanium(IV) Isopropoxide. *Russ. J. Org. Chem.* **2006**, *42*, 659-664.
(27) Mills, L. R.; Zhou, C.; Fung, E.; Rousseaux, S. A. L. Ni-Catalyzed β-Alkylation of Cyclopropanol-Derived Homoenolates. *Org. Lett.* **2019**, *21*, 8805-8809.
